# Hydration‐Gated Photoinduced Electron Transfer in BODIPY Dyes

**DOI:** 10.1002/anie.3726851

**Published:** 2026-09-29

**Authors:** Venkata N. K. Mamillapalli, Irina A. Okkelman, Fatima Akhssas, Greta Sambucari, Angela C. Debruyne, Dinesh Beniwal, Christophe Bucher, Mariangela Di Donato, Ruslan I. Dmitriev, Mikhail A. Filatov

**Affiliations:** ^1^ School of Chemical and Biopharmaceutical Sciences Technological University Dublin Dublin Ireland; ^2^ Tissue Engineering and Biomaterials Group, Department of Human Structure and Repair, Faculty of Medicine and Health Sciences Ghent University Ghent Belgium; ^3^ CNRS, ENS De Lyon Lyon France; ^4^ LENS (European Laboratory for Non‐Linear Spectroscopy) Firenze Italy; ^5^ Comprehensive Cancer Centre, School of Cancer and Pharmaceutical Sciences King's College London UK

**Keywords:** BODIPY, charge‐transfer state, fluorescence lifetime imaging microscopy, fluorescence probe, photoinduced electron transfer

## Abstract

Fluorescent probes based on photoinduced electron transfer (PET) are widely used to image intracellular environments. Here we report donor–acceptor dyads based on the BODIPY chromophore in which PET is selectively activated upon hydration. Replacing fluorine groups at boron with triethylene glycol (TEG) chains confers aqueous solubility and modulates the electronic properties of the boron‐coordinated core, suppressing PET in polar organic solvents while enabling efficient charge separation in water. This switching arises from hydrogen bonding at the alkoxy oxygens, which strengthens the effective acceptor character of the BODIPY, supported by electrochemistry, transient absorption spectroscopy, and DFT calculations. The mechanism is general across dyads incorporating various aromatic donors and BODIPY substitution patterns. The lead compound, 5‐TEG, displays hydration‐dependent fluorescence lifetimes spanning a 6 ns range. In fluorescence lifetime imaging microscopy (FLIM) of living cells, tumor spheroids, and intestinal organoids, 5‐TEG provides high brightness and lifetime contrast, enabling segmentation of subcellular compartments and reporting on microenvironmental differences. These results establish hydration‐gated PET as a tunable photophysical mechanism for designing fluorescence probes with lifetime‐encoded readouts in complex biological environments.

## Graphical abstract

Hydration switches photoinduced electron transfer (PET) ON in alkoxy‐coordinated BODIPY dyes via specific hydrogen bonding. This gating yields a fluorescence lifetime span of ∼6 ns, enabling fluorescence lifetime imaging microscopy (FLIM) contrast in live cells and organoids.

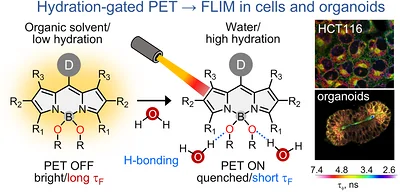

## 1 Introduction

Intracellular processes are governed by dynamic fluctuations in key physicochemical parameters of the cellular milieu, including pH, electric potential, viscosity, local polarity, and temperature [1]. Real‐time monitoring of these parameters inside living cells with high spatial and temporal resolution is therefore essential for mechanistic understanding of cell function and for disease diagnostics [2]. For instance, abnormal changes in the polarity of the membrane lipid environment are closely associated with altered lipid metabolism and are increasingly recognized as a defining characteristic of cancerous cells, which often display a more hydrophobic (lower polarity) membrane interior than normal cells due to elevated de novo lipid synthesis that alters membrane lipid composition [3].

Fluorescence‐based methods are well suited for quantitative imaging of cellular environment parameters, as they combine ease of implementation, minimal invasiveness, single‐molecule sensitivity, and sub‐cellular resolution. Small‐molecule fluorescent sensors, in particular can deliver information on environmental parameters through changes in emission intensity, wavelength, and/or lifetime. Consequently, environment‐sensitive fluorophores have become indispensable tools for dissecting the spatial organization and dynamic behavior of living cells [4].

The key photophysical mechanisms involved in the design of fluorescent probes include photoinduced electron transfer (PET) [5], twisted intramolecular charge transfer (TICT) [6], excited‐state intramolecular proton transfer (ESIPT) [7], and fluorescence resonance energy transfer (FRET) [8]. Notably, charge‐transfer (CT) excited states exhibit strong dependence on local polarity, which modulates the thermodynamic driving force for electron transfer [9]. This sensitivity can be exploited through structural changes of the probe itself or by changes in the environment, making it possible to tune the photophysical response. Consequently, fluorescent sensors involving CT states have enabled mapping of membrane organization and macromolecular crowding at lipid interfaces, ion binding, pH gradients, and other processes in living cells [10].

Yet a fundamental limitation restricts the informative value of the data obtained from such probes: in cellular environments, polarity (quantified by the medium dielectric constant) is intrinsically coupled to other physicochemical parameters, creating cross‐sensitivity. Most directly, polarity and hydration can be strongly coupled in biological environments, as changes in local water accessibility can alter the polarity and hydrogen‐bonding characteristics of the microenvironment [11]. An equally important source of interference exists between polarity and viscosity, since PET‐ and TICT‐based mechanisms involve geometric relaxation of the probe (such as twisting or planarization) as a part of their excited‐state dynamics [12, 13, 14]. This conformational motion renders emission sensitive to microviscosity at the same time as polarity [15]. As a result, a single optical signal often encodes overlapping contributions from multiple environmental variables rather than a single parameter, which can obscure interpretation.

Although this interdependence is widely recognized, design of sensors that selectively respond to a single environmental parameter remains challenging. Polarity‐ and hydration‐sensitive probes illustrate this problem particularly well. Specialized probes, such as Prodan [16], Laurdan [17], and others [18], have been developed to monitor hydration‐dependent lipid packing in live cells. However, these dyes often respond to generic polarity and show pronounced solvatochromism in polar organic solvents, making it difficult to deconvolute hydration‐specific effects. A similar challenge exists for viscosity‐sensitive probes: designing rotors whose response correlates selectively with viscosity while remaining insensitive to changes in local polarity remains difficult [19].

Here we introduce a new approach to PET‐based fluorescent imaging probes that undergo selective activation of intramolecular PET under aqueous hydration conditions. The strategy employs a BODIPY platform in which the fluorine substituents at the boron center are replaced by alkoxy groups, and the *meso*‐aryl group plays the role of an electron donor. We found that a broad range of such O‐BODIPYs are weaker electron acceptors than the corresponding F‐BODIPYs, resulting in thermodynamically unfavorable PET in polar organic solvents. In contrast, O‐BODIPYs undergo efficient PET selectively in water due to the hydrogen‐bond‐driven stabilization of the charge‐transfer states. As a result, these chromophores remain emissive in polar organic solvents and mixed organic–aqueous media but display a pronounced fluorescence quenching in the presence of water. Together, these findings define a distinct regime of excited‐state control in which PET is activated by aqueous hydration rather than by generic solvent polarity (hydration‐gated PET). This mechanism provides a new molecular design strategy to fluorescence‐based optical readout of local water accessibility in complex biological environments.

## 2 Results and Discussion

### 2.1 O‐BODIPYs as Building Blocks for Water‐Selective PET Probes

BODIPY dyes are among the most widely used organic chromophores, with applications ranging from biological imaging [20, 21] to nonlinear optical materials [22, 23]. Their excited‐state reduction potential can be tuned over a wide range [24], providing control over the thermodynamic driving force of PET and enabling the rational design of responsive fluorescent probes [25].

Adequate aqueous solubility is a fundamental requirement for fluorescent probes intended for biological applications, and several approaches for designing water‐soluble BODIPYs have been reported, including conjugation of charged or polar moieties at the meso position, pyrrolic carbons, or boron center [26, 27, 28]. In this context, there has been growing interest in O‐BODIPYs, in which the fluorine substituents at boron are replaced with alkoxy or aryloxy substituents (for representative structures see Table S1). Although F‐BODIPYs and O‐BODIPYs display comparable optical properties, a noticeable difference emerges in their electrochemical behavior. Replacing B–F with B–OR groups makes the BODIPY core harder to reduce [29]. Accordingly, for O‐BODIPYs the first reduction potentials shift to more negative values relative to F‐BODIPYs, by approximately 0.1 V per fluorine substitution [30], while leaving the optical properties essentially unchanged (Figure 1a).

**FIGURE 1:**
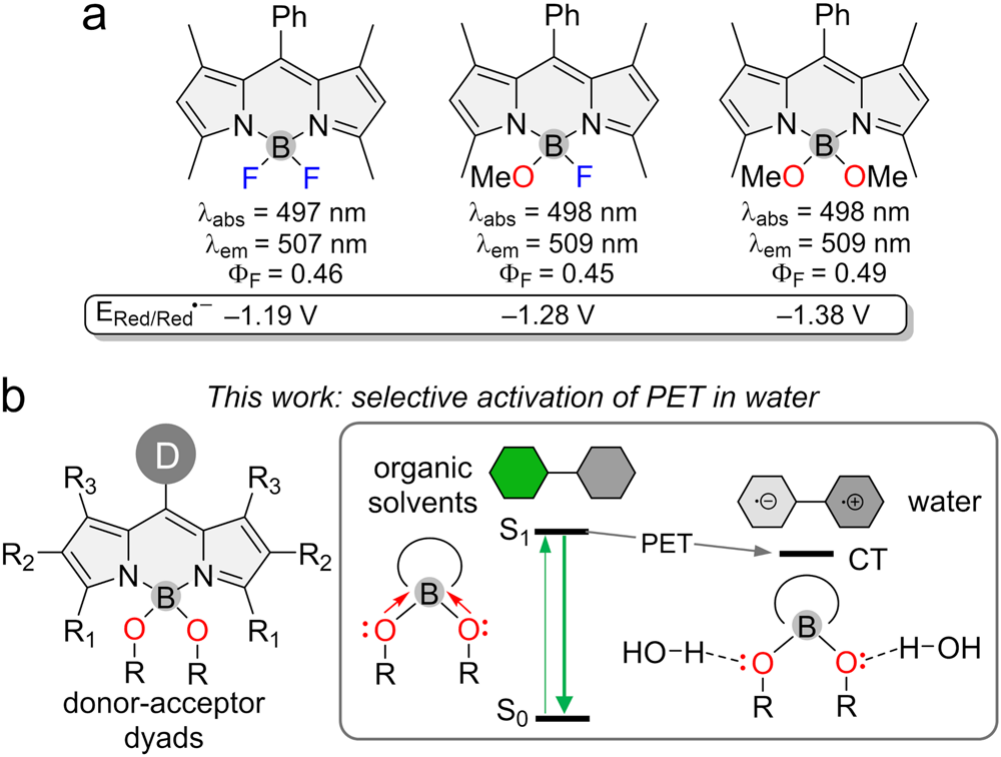
(a) Effect of substitution at the boron center on the first reduction potential (vs SCE) [30]. (b) General structure of the donor–acceptor dyads investigated in this work, and schematic representation of hydration‐gated PET.

In our previous work, we reported donor–acceptor dyads in which PET occurs from a *meso*‐aryl donor to the BODIPY acceptor [31], including water‐soluble derivatives obtained by introducing sulfobetaine fragments at the boron center [32] or by substitution at the pyrrolic positions [33]. In these molecules, fluorescence is quenched both in polar aprotic solvents and in water (Table S2), due to PET being thermodynamically favourable in both media.

Although PET has been reported in individual O‐BODIPY systems [30, 34], the use of boron‐center substitution to tune the electron‐accepting strength of the BODIPY core and thereby control PET energetics in donor–acceptor dyads has not been explored. We therefore selected the anthracene–BODIPY dyad **1** [35] as a model system and prepared its dialkoxy analogues **1‐OMe** and **1‐TEG**, bearing methoxy and triethylene glycol (TEG) groups, respectively (Figure 2a). **1‐TEG** was obtained by substitution of the fluorine atoms in **1** with triethylene glycol monomethyl ether under Lewis‐acid catalysis (AlCl_3_); full synthetic procedures and characterization are provided in the (Section S2). The resulting derivative **1‐TEG** shows excellent solubility in water, with no signs of aggregation at concentrations of 10^−^^6^–10^−^^5^ M (Figure S2).

**FIGURE 2:**
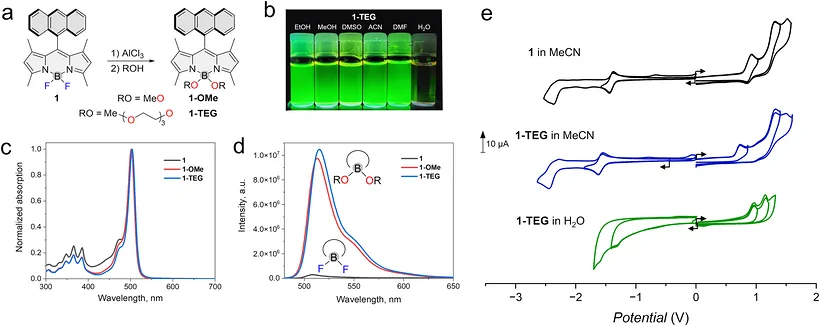
(a) Synthesis of O‐BODIPY derivatives **1‐OMe** and **1‐TEG**. (b) Photograph of **1‐TEG** in selected polar solvents under UV illumination. (c) Absorption spectra of **1**, **1‐OMe**, and **1‐TEG** in acetonitrile. (d) Emission spectra of **1**, **1‐OMe**, and **1‐TEG** in acetonitrile, recorded for samples adjusted to the same absorbance at the excitation wavelength (470 nm). (e) Cyclic voltammograms recorded for **1** and **1‐TEG** in acetonitrile and in water.

Substitution with alkoxy groups at boron has only a minor effect on absorption yet produces a profound change in emission properties (Figure 2c,d). The parent dyad **1** is strongly emissive in non‐polar solvents (Φ_F_ = 0.99 in hexane and 0.92 in toluene), reflecting inefficient PET. In more polar media, fluorescence is progressively quenched: in dichloromethane Φ_F_ decreases to 0.072, and in acetonitrile Φ_F_ drops to 0.006, consistent with efficient PET driven by dielectric stabilization of the formed CT state [34]. By contrast, the dialkoxy analogues **1‐OMe** and **1‐TEG** remain strongly emissive across polar organic solvents (Figure 2c,d; Tables 1 and S3).

**TABLE 1:** Spectroscopic data for compounds **1**, **1‐OMe** and **1‐TEG** in different solvents.

Compound	Solvent^a^	λ_abs_ (nm)	λ_em_ ^b^ (nm)	Φ_F_ ^c^	Lifetime, ns (%)^d^
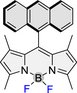 **1**	CYH	504	515	0.99	5.21
	TOL	508	521	0.92	4.74
	DCM	506	517	0.072	2.83 (17), 5.22 (83)
	EtOH	505	513	0.038	1.23 (11), 4.74 (89)
	MeOH	502	510	0.028	2.55 (31), 5.53 (69)
	MeCN	502	512, 621	0.006	2.16 (28), 5.08 (72)
	DMF	505	518, 615	0.011	3.15 (54), 5.5 (46)
	DMSO	507	514, 645	0.010	3.4 (40), 5.25 (60)
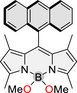 **1‐OMe**	CYH	506	517	0.913	5.28
	TOL	508	518	0.941	4.64
	DCM	506	517	0.890	5.36
	EtOH	504	515	0.391	5.53
	MeOH	504	515	0.314	4.43
	MeCN	503	514	0.398	4.58
	DMF	506	518	0.569	5.03
	DMSO	507	520	0.365	4.57
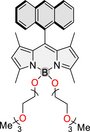 **1‐TEG**	CYH	507	517	0.903	5.41
	TOL	508	519	0.905	4.75
	DCM	507	518	0.847	5.53
	EtOH	505	516	0.374	5.68
	MeOH	505	516	0.381	4.81
	MeCN	504	515	0.444	4.86
	DMF	506	518	0.646	5.06
	DMSO	508	521	0.380	4.61
	H_2_O	502	513	0.005	0.49 (13), 2.09 (44), 5.5 (43)

^a^ CYH—cyclohexane, TOL—toluene, DCM—dichloromethane, EtOH—ethanol, MeOH—methanol, MeCN—acetonitrile, DMF—dimethylformamide, DMSO—dimethylsulfoxide.

^b^ Samples were excited at the vibrational shoulder of the BODIPY absorption (470 nm).

^c^ Measured using Rhodamine 6G as a standard (Φ_F_ = 0.95 in EtOH) [36].

^d^ Relative contributions of components in bi‐ and tri‐exponential decays.

In acetonitrile Φ_F_ = 0.40 for **1‐OMe** and 0.44 for **1‐TEG**, substantially higher than for dyad **1** (Φ_F_ < 0.01). Remarkably, however, fluorescence of **1‐TEG** is strongly quenched in water (Φ_F_ = 0.005) (Figure 2b), with faster fluorescence decays and a prominent sub‐nanosecond component (τ = 0.49 (13%), 2.09 (44%), 5.5 (43%) ns). This contrasts with long‐lived decays in organic solvents, for example in acetonitrile where τ ≈ 4.9 ns (Figure 2d). The fast component in water indicates efficient PET, whereas such components are not observed in organic solvents. Although BODIPYs functionalized with ethylene glycol chains have been reported previously (e.g., entries 6–7 in Table S1) [37, 38], such hydration‐dependent modulation of PET has not been noted.

To understand the role of alkoxy groups in modulating PET, we performed electrochemical measurements comparing **1** and **1‐TEG** in acetonitrile and water (Section S5). The first BODIPY‐centered reduction potential of **1‐TEG** (*E*_1/2_ = −1.687 V vs Fc^+^/Fc, Table S16) is more negative than that of **1** (*E*_1/2_ = −1.495 V vs Fc^+^/Fc; Figure 2e), confirming that the dialkoxyBODIPY core is a weaker electron acceptor. Efficient PET is observed in **1** in polar solvents (Table 1) despite modest endergonicity (ΔG°_PET_ = +0.14 eV), consistent with prior reports [39]. In **1‐TEG**, the driving force for charge separation is strongly endergonic in acetonitrile (ΔG°_PET_ = +0.39 eV), rationalizing the recovery of fluorescence. Going from acetonitrile to water changes the behavior of **1‐TEG**: electrochemical measurements in water reveal an anodic shift of the first irreversible reduction wave to *E*_p_ = −1.549 V versus Fc^+^/Fc, indicating that the O‐BODIPY core becomes easier to reduce under aqueous conditions. Accordingly, ΔG°_PET_ decreases to +0.09 eV in water, rendering charge separation thermodynamically accessible and rationalizing the observed fluorescence quenching. The generality of this mechanism across different donor–acceptor architectures is demonstrated in the following section.

### 2.2 Scope of Hydration‐Gated PET: Donor and Acceptor Variation in BODIPY‐TEGs

We propose that hydration‐activated PET is a general phenomenon for BODIPY donor–acceptor systems bearing dialkoxy substitution at boron. Literature data on water‐soluble O‐BODIPYs (Table S1) corroborate this generality: dialkoxyboron compounds show strongly quenched fluorescence in water, whereas aryloxy‐ or alkoxycarbonyl‐substituted O‐BODIPYs [40, 41] remain fluorescent in aqueous media. Representative examples include 4,4‐dimethoxyBODIPY (Table S1, entry 3), for which Φ_F_ drops from 0.57 in toluene to 0.051 in water [42, 43], an oligoethylene glycol‐appended O‐BODIPY (entry 7) quenched from 0.514 in DCM to 0.089 in water [38], and derivatives reported by the Nagano and Zhao groups (entries 4 and 8) showing similarly low Φ_F_ values [30, 44]. In contrast, aryloxy‐ and alkoxycarbonyl‐substituted O‐BODIPYs (entries 9–13, 16–21) remain highly emissive (Φ_F_ = 0.4–0.9) under aqueous conditions, confirming that the hydration response is specifically associated with two alkoxy substituents at the boron center.

To probe generality across donor–acceptor architectures, we synthesized TEG‐functionalized compounds featuring different aromatic donors (pyrene **2‐TEG**, carbazole **3‐TEG**, 9‐methylanthracene **4‐TEG** and **5‐TEG,** Figure 3) alongside two reference compounds lacking an aromatic donor (**6‐TEG** and **7‐TEG**). To further probe electronic effects, **8‐TEG** and **9‐TEG** bearing 2,5‐dimethoxyphenyl and 4‐pyridyl substituents at the meso position, respectively, were also prepared.

**FIGURE 3:**
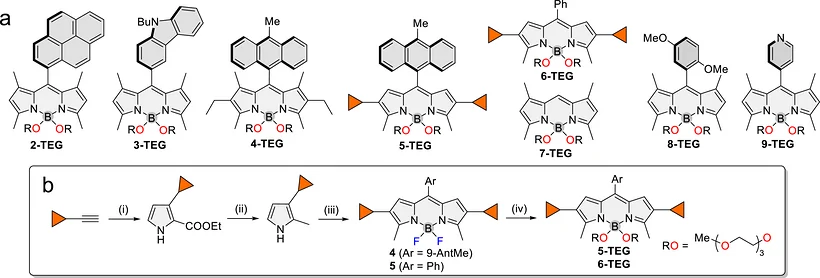
(a) Structures of the studied **BODIPY‐TEG**s. (b) Synthetic route to 2,6‐dicyclopropyl BODIPY derivatives. Conditions: (i) ethyl isocyanoacetate, Ag_2_CO_3_; (ii) LiAlH_4_; (iii) (a) ArCHO, TFA; (b) DDQ; (c) DIPEA, BF_3_•Et_2_O; (iv) (a) AlCl_3_, DCM; (b) triethylene glycol monomethyl ether.

Dyad **5‐TEG** incorporates cyclopropyl substituents at the 2,6‐positions, which induce a bathochromic shift of ∼60 nm relative to the other compounds. This red shift arises from hyperconjugative interaction (σ–π conjugation) between the Walsh orbitals of the strained cyclopropyl C─C bonds and the BODIPY π‐system. DFT calculations show that this interaction raises the BODIPY HOMO while leaving the LUMO comparatively unaffected (Figures S48 and S49), thereby decreasing the HOMO–LUMO gap, consistent with the previous report by the Kuimova group [45].

Table 2 summarizes photophysical properties of the expanded **BODIPY‐TEG** series in MeCN and water. As demonstrated for **1‐TEG**, pyrene‐ and carbazole‐BODIPY dyads remain strongly fluorescent in MeCN (Φ_F_ = 0.48–0.74, τ = 4.6–7.1 ns) yet undergo dramatic quenching in water (Φ_F_ = 0.003–0.07) with faster decay kinetics characteristic of PET. Reference compounds **6‐TEG** and **7‐TEG**, lacking an electron donor, likewise retain high fluorescence in both solvents. **8‐TEG** similarly shows fluorescence quenching and lifetime shortening in water, whereas **9‐TEG** remains largely solvent‐insensitive, consistent with the poor electron‐donating ability of the pyridyl group.

**TABLE 2:** Spectroscopic data for **BODIPY‐TEG** compounds in MeCN and water.

Compound	Solvent	λ_abs_ (nm)	λ_em_ ^a^ (nm)	Φ_F_ ^b^	Lifetime, ns (%)^c^
**2‐TEG**	MeCN	502	515	0.743	5.67
	H_2_O	501	512	0.003	0.30 (26), 1.45 (48), 4.39 (26)
**3‐TEG**	MeCN	499	510	0.573	4.64
	H_2_O	499	508, 742	0.007	0.14 (66), 1.07 (19), 4.97 (15)
**4‐TEG**	MeCN	526	541	0.690	6.41
	H_2_O	528	543	0.07	0.11 (49), 1.37 (39), 6.24 (12)
**5‐TEG**	MeCN	553	585	0.624	6.88
	H_2_O	565	609	0.021	0.13 (21), 1.9 (25), 5.49 (54)
**6‐TEG**	MeCN	547	587	0.481	7.16
	H_2_O	548	590	0.367	5.82
**7‐TEG**	MeCN	503	510	0.880	5.35
	H_2_O	501	508	0.918	5.99
**8‐TEG**	MeCN	502	514	0.75	4.71
	H_2_O	501	515	0.007	0.1 (19), 1.87 (36), 3.99 (45)
**9‐TEG**	MeCN	502	516	0.246	0.82 (81), 2.94 (19)
	H_2_O	501	518	0.275	0.33 (11), 2.71 (89)

^a^ Recorded by exciting the samples at the vibrational shoulder of the BODIPY absorption, 470 nm or 520 nm (for **5‐TEG** and **6‐TEG**).

^b^ Measured using Rhodamine 6G as a standard (Φ_F_ = 0.95 in EtOH) [39].

^c^ Relative contributions of components in bi‐ and tri‐exponential decays.

Characterization across a range of solvents (Table S3), confirm that the hydration‐activated PET quenching is specific to aqueous environments rather than a polarity effect. Formamide is the only organic solvent tested that replicates water's PET‐activating effect, producing similarly strong fluorescence quenching (Φ_F_ in the range of 0.02–0.18, Table S3). This behaviour correlates with its pronounced hydrogen‐bonding ability. The Kamlet–Taft parameters for formamide (α = 0.71, β = 0.48) [46] are considerably closer to those of water (α = 1.17, β = 0.47) than to those of acetonitrile (α = 0.19, β = 0.31). Thus, formamide can form strong hydrogen bonds with the alkoxy oxygens and reproduce the local interactions responsible for PET activation in water.

The cyclopropyl‐substituted dyad **5‐TEG** exhibits red‐shifted emission (λ_em_ = 609 nm in water, Figure 4a), shifting the optical response of hydration‐activated PET toward longer wavelengths advantageous for fluorescence bioimaging. Progressive fluorescence quenching is observed in EtOH/H_2_O and DMSO/H_2_O mixtures (Figures 4b and S4; Tables S4–S9), with water‐content‐dependent lifetime shortening analogous to **1‐TEG**. The fluorescence is partially restored in D_2_O (Φ_F_ = 0.031, ⟨τ⟩_amp_ = 4.01 ns vs. 3.47 ns in H_2_O, Figure S5), consistent with weaker deuterium bonding.

**FIGURE 4:**
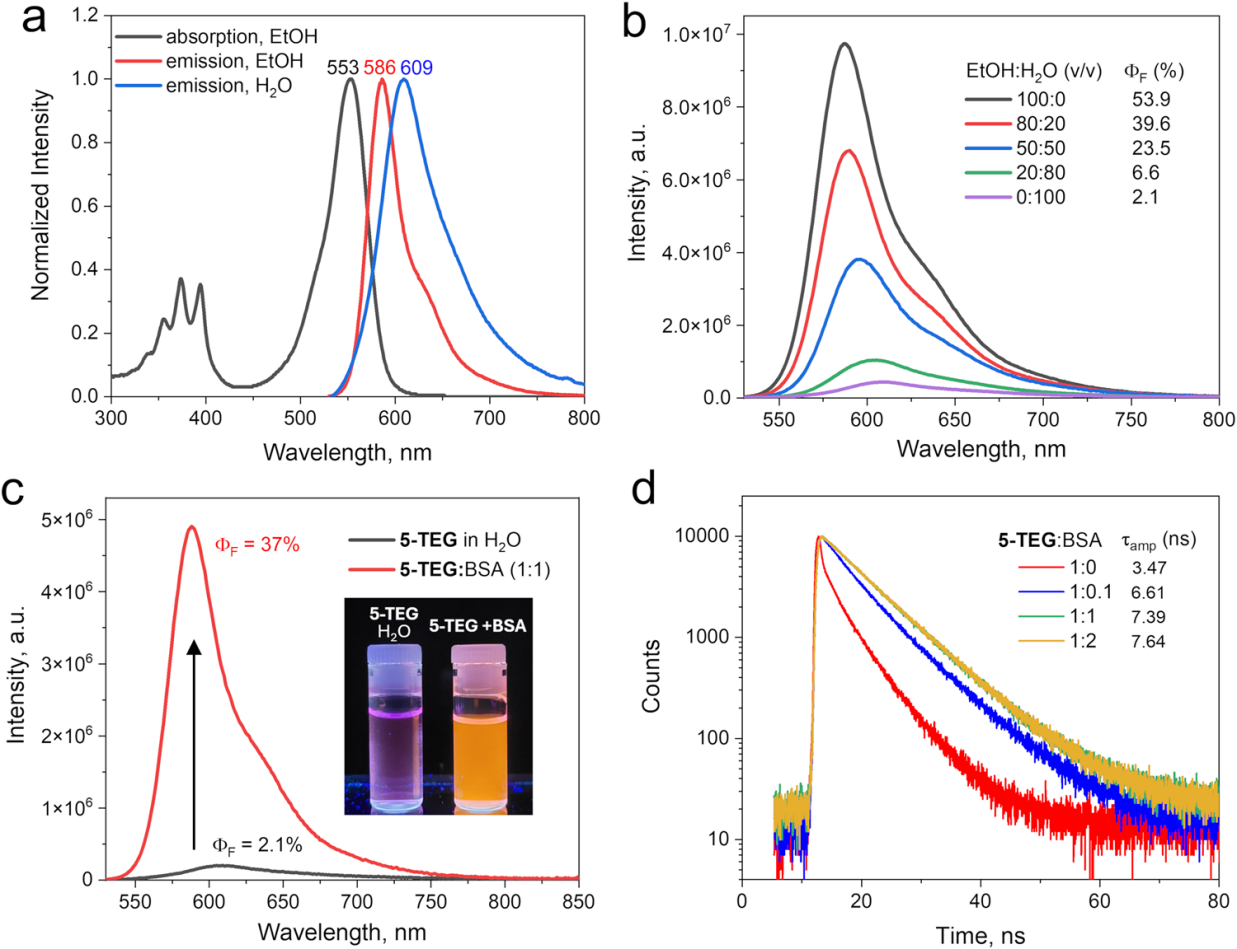
(a) Selected absorption and emission spectra of **5‐TEG**. (b) Emission spectra in EtOH/water mixtures (ratios indicated in the legend). (c) Selected emission spectra of the **5‐TEG** and upon complexation with BSA (50 µM). Inset: photographs of **5‐TEG** solutions under UV illumination showing weak emission in water and bright fluorescence after addition of BSA. (d) Time‐resolved fluorescence decay traces of **5‐TEG** after complexation with BSA.

Importantly, fluorescence quenching of the TEG‐substituted O‐BODIPYs in water is not caused by dye degradation via B–OR hydrolysis. Although slow hydrolysis of alkoxyBODIPYs in aqueous media has been reported previously [47], we found it to be too slow under our conditions to account for the immediate loss of emission. Stability of **7‐TEG** was therefore assessed by ^1^H NMR monitoring in neat D_2_O (10 mM, room temperature): spectra (Figure S6) show slow hydrolysis, with an estimated half‐life of ca. 90 h. Hydrolysis is strongly suppressed at pH > 7: in PBS buffer (Figure S7). We also examined the photostability under continuous illumination, as BODIPYs are generally less photostable in aqueous than in organic media [48]. Under continuous 520 nm irradiation (14 mW cm^−^^2^), the half‐life of **5‐TEG** in PBS is approximately 40 min (Figure S8), indicating sufficient photostability for fluorescence sensing/imaging experiments.

Electrochemical characterization of **5‐TEG** and its F‐BODIPY precursor **5** confirms the hydration‐gated PET activation. Dyad **5** exhibits a first reduction potential of −1.407 V versus Fc^+^/Fc and methylanthracene oxidation at 0.966 V versus Fc^+^/Fc in acetonitrile (ΔG°_PET_ ≈ −0.15 eV). Introducing TEG substituents shifts the reduction potential cathodically to −1.520 V versus Fc^+^/Fc in MeCN, rendering PET in **5‐TEG** thermodynamically unfavorable (ΔG°_PET_ ≈ +0.27 eV). Transfer to water produces an anodic shift to −1.490 V versus Fc^+^/Fc (Table S16), switching the driving force to ΔG°_PET_ ≈ −0.03 eV.

Given that PET is efficient in water, we investigated whether triplet excited states formation occurs in these systems. In the corresponding F‐BODIPY‐based dyads **1**–**4**, charge recombination from the CT state populated the triplet manifold via spin–orbit charge‐transfer intersystem crossing (SOCT‐ISC) [49]. In compound **5**, this process resulted in singlet oxygen (^1^O_2_) generation in organic solvents, with quantum yields of Φ_Δ_ = 0.045 in toluene, 0.25 in dichloromethane, and 0.44 in acetonitrile (Figure S10a). Using anthracene‐9,10‐dipropionic acid disodium salt (ADPA) as a water‐soluble chemical trap and Rose Bengal as a reference photosensitizer, we detected no appreciable singlet oxygen formation upon irradiation of **5‐TEG** in water (Figure S10c). This conclusion was further supported by measurements of ^1^O_2_ phosphorescence at 1276 nm in D_2_O for **1‐5‐TEG** series, which showed negligible ^1^O_2_ generation (Figure S9). The absence of singlet oxygen production across the series supports suitability for fluorescence imaging in living cells.

Complexation of **5‐TEG** with bovine serum albumin (BSA) in water produces a strong “turn‐on” of fluorescence, visible as a large intensity increase upon addition of BSA (Figure 4c). This enhancement is reflected in both the fluorescence quantum yield and the excited‐state lifetimes (Figures 4d and S11; Table S10): Φ_F_ increases 18‐fold relative to water (0.021 → 0.37), accompanied by recovery of a long lifetime (⟨τ⟩_amp_ ≈ 7.6–7.8 ns), approaching values typical of non‐aqueous environments. Addition of 50 mM NaCl reduced the BSA‐enhanced fluorescence by ∼50% (Figure S12) and shortened the mean fluorescence lifetime from 7.6 to 6.7 ns. BSA complexation of the reference compounds **6‐TEG** and **7‐TEG** produced only marginal changes in fluorescence (Φ_F_: 0.367 → 0.422 and 0.918 → 0.921, respectively; Figure S13).

BSA binding likely places **5‐TEG** in a less hydrated microenvironment, reducing interactions of bulk water with the B–OR fragment and thereby suppressing hydration‐gated PET. Consistent with this interpretation, molecular docking identified a low‐energy pose in which the B–OR region is sterically enclosed by the protein (Figure S51), potentially shielding the boron‐bound oxygens from bulk water. The effect of NaCl may reflect ionic‐strength‐dependent changes in dye binding and/or water accessibility within the BSA complex. This may account for the recovery of long lifetimes and high fluorescence efficiency upon BSA binding, although conformational restriction within the protein pocket may also contribute [50].

Addition of sodium dodecyl sulfate (SDS) to **5‐TEG** in PBS produces a pronounced fluorogenic turn‐on in both H_2_O and D_2_O (Figure S14a–d), accompanied by progressive recovery of long fluorescence lifetimes (Tables S11 and S12). At higher SDS concentrations, the decay becomes monoexponential, reaching ∼7.20 ns in H_2_O and ∼7.66 ns in D_2_O, consistent with formation of a predominant SDS‐associated state in which PET is strongly suppressed. Notably, the H_2_O/D_2_O isotope effect observed for free **5‐TEG** persists in the SDS‐associated state (⟨τ⟩amp = 7.20 vs 7.66 ns). This indicates that, although SDS association restricts exposure to bulk water, the dye remains sensitive to the local hydration, consistent with localization within a hydrated interfacial region of the micellar assembly.

By comparison, for **5‐TEG**, changing viscosity in MeOH/glycerol mixtures produces only modest lifetime variations (≈ 6.6 ns in MeOH to ≈4.9 ns in glycerol; Table S13), and variable‐temperature fluorescence (Figures S16 and S17) shows only a limited intensity decrease upon heating. Together with the solvent‐dependent measurements, these observations support hydration as the dominant factor controlling PET, while viscosity and thermal effects are comparatively small.

### 2.3 Excited‐State Dynamics: Transient Absorption Spectroscopy

To clarify the selective activation of PET in water, we performed transient absorption measurements on 5 in MeCN and **5‐TEG** in both MeCN and water.

Immediately after excitation, **5** in MeCN displays an intense negative band centered at ∼550 nm with a tail extending to ∼650 nm, together with a positive band near ∼450 nm (Figure 5a). The negative feature is attributed to overlapping ground‐state bleach (GSB) and stimulated emission (SE), while the positive band corresponds to excited‐state absorption (ESA) of the initially populated BODIPY‐centered S_1_ state. A rapid spectral relaxation occurs within ∼0.5 ps. Within 8.3 ps, the spectrum changes markedly: the SE contribution is replaced by positive signals with maxima at ∼580 and ∼650 nm, assigned to the BODIPY radical‐anion and anthracene radical‐cation [34], respectively, indicating charge separation. Further evolution within 27.2 ps is consistent with relaxation of the CT state. On the nanosecond timescale (2.3 ns), the ESA band in the blue region shifts (≈ 410 → 430 nm) and the structured 580/650 nm features evolve into a broad band spanning ∼585–750 nm. The long‐lived residual signal persists beyond the accessible delay window (>3.5 ns, Figure 5d) and is assigned to a BODIPY‐localized triplet state formed upon charge recombination, corroborated by singlet oxygen generation measurements (Figure S10a).

**FIGURE 5:**
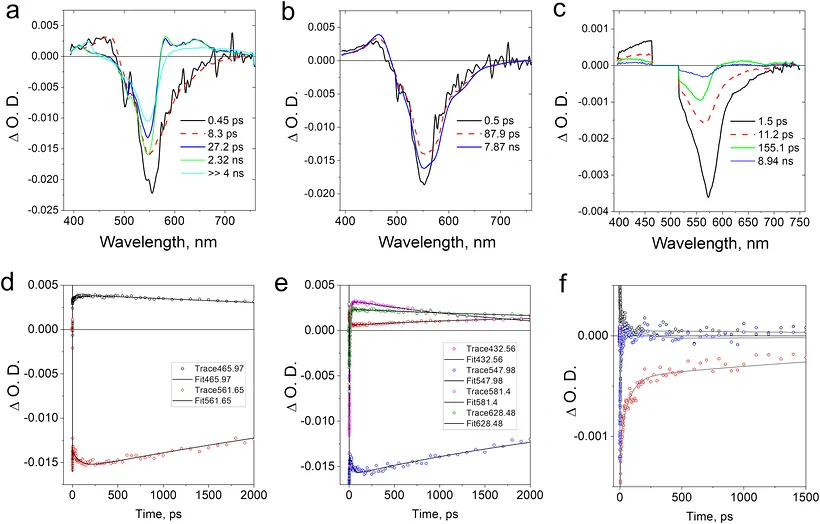
Evolution‐associated difference spectra (EADS) from global analysis of transient absorption data (λ_exc_ = 510 nm) for (a) **5** in MeCN, (b) **5‐TEG** in MeCN, and (c) **5‐TEG** in water. Panels (d–f) show the corresponding selected kinetic traces for (d) **5** (MeCN), (e) **5‐TEG** (MeCN), and (f) **5‐TEG** (H_2_O).

In MeCN, **5‐TEG** shows smaller dynamic changes. The early spectra exhibit the same characteristic GSB/SE feature centered at ∼550 nm with a red tail and a positive ESA band peaking at ∼460 nm (Figure 5b). A minor evolution within ∼0.5 ps, is consistent with rapid solvent‐induced relaxation. A slower component (∼88 ps) produces only subtle intensity changes in both bands, tentatively attributable to conformational and vibrational relaxation. The excited state decays with a lifetime of ∼7.9 ns, returning predominantly to the ground state through radiative pathways, in agreement with the high fluorescence quantum yield.

By contrast, **5‐TEG** in water (Figure 5c) exhibits pronounced quenching dynamics. The initial spectra show a broad negative band around ∼570 nm and an ESA feature spanning ∼380–450 nm. Both signals decrease strongly within ∼3 ps, indicating a fast non‐radiative decay channel for a fraction of the excited‐state population. On a slower timescale (∼64 ps), a broad positive band grows between ∼580 and 680 nm, consistent with charge separation between the BODIPY and anthracene units. Charge recombination occurs within ∼90 ps, as indicated by the disappearance of the positive CT absorption. The remaining signal decays on the ∼3.2 ns timescale.

Taken together, the transient absorption data provide direct, time‐resolved confirmation of the PET switching. In acetonitrile, **5‐TEG** decays predominantly via radiative pathways without pronounced charge‐transfer signatures, consistent with the endergonic ΔG°_PET_ and the high Φ_F_ values in organic media. In water, ultrafast charge separation is clearly evidenced by the growth of radical‐anion/cation absorption bands on the picosecond timescale, followed by rapid charge recombination. This behavior is consistent with the exergonic driving force (ΔG°_PET_ ≈ −0.03 eV) and the strongly quenched fluorescence, confirming selective activation of PET in water.

### 2.4 DFT Modelling of Hydration Effects on PET in the Dialkoxy‐BODIPY Core

To rationalize these observations, we propose that the key factor for PET switching is modulation of O→B electron donation in the tetra‐coordinate N_2_B(OR)_2_ boron center. The alkoxy oxygens can act as strong donors through σ‐donation and hyperconjugation involving the B─O and B─N bonding framework. In polar organic solvents, this effect increases the electron density at boron and, through the B─N bond, contributes to a more electron‐rich dipyrrin core, thereby decreasing the electron‐accepting ability of the BODIPY scaffold. In strongly hydrogen‐bonding media such as water or formamide, the oxygen lone pairs engage in hydrogen bonding, decreasing donation toward boron. This makes the boron center more electron‐deficient and consequently increases the electron‐accepting character of the dipyrrin core.

To gain further mechanistic insight, DFT calculations were performed for anthracenylBODIPY **5‐OMe** with two explicit water molecules hydrogen‐bonded to the methoxy oxygens, and the reference F‐BODIPY **5**. The calculations confirm a measurable redistribution of electron density upon hydrogen bonding, consistent with attenuated alkoxy→boron donation.

Analysis of Mayer bond orders provides direct evidence for this effect. As shown in Table 3, the B─O bond orders decrease markedly upon hydrogen bonding: in vacuum, the B─O bond orders in **5‐OMe** are ∼1.12, but drop to ∼0.89–0.93 in **5‐OMe·2H_2_O**. Concomitantly, the B─N bond orders increase from ∼0.80 to ∼0.85–0.86. The same trend is preserved in MeCN: B─O bond orders decrease from ∼1.116 to ∼0.88–0.92, while B─N bond orders rise from ∼0.798 to ∼0.852–0.857. The reduction in B─O bond order reflects diminished O→B donation when the oxygen lone pairs engage in hydrogen bonding, while the increase in B─N bond order indicates strengthening of the B–N interaction as boron draws electron density from the dipyrrin nitrogen atoms. The slight asymmetry in bond orders for **5‐OMe·2H_2_O** reflects non‐equivalent hydrogen‐bonding geometries at the two methoxy oxygens.

**TABLE 3:** Parameters for the model BODIPY systems computed at the B3LYP/6‐31+G(d,p) level. Mayer bond orders quantify the covalent character of B─O and B─N bonds.

		Mayer bond orders^a^						
Compound	Conditions	B–O^1^	B–O^2^	B–N^1^	B–N^2^	HOMO‐1^b^ (eV)	HOMO (eV)	LUMO (eV)
**5**	Vacuum	n/a	n/a	n/a	n/a	−5.58 (Ant)	−5.51 (BDP)	−2.70 (BDP)
**5**	PCM‐MeCN	n/a	n/a	n/a	n/a	−5.66 (BDP)	−5.62 (Ant)	−2.90 (BDP)
**5‐OMe**	Vacuum	1.1230	1.1229	0.7956	0.7956	−5.55 (Ant)	−5.36 (BDP)	−2.53 (BDP)
**5‐OMe**	PCM‐MeCN	1.1161	1.1162	0.7982	0.7982	−5.60 (Ant)	−5.49 (BDP)	−2.69 (BDP)
**5‐OMe·2H_2_O**	Vacuum	0.8913	0.9265	0.8587	0.8360	−5.70 (Ant)	−5.62 (BDP)	−2.81 (BDP)
**5‐OMe·2H_2_O**	PCM‐MeCN	0.8816	0.9225	0.8567	0.8522	−5.61 (Ant)	−5.58 (BDP)	−2.80 (BDP)

^a^ Superscripts 1, 2 label the two symmetry‐equivalent oxygen (O) or nitrogen (N) atoms.

^b^ BDP and Ant stand for BODIPY and 9‐methylanthracene units, respectively.

Orbital energies are presented in Table 3 and the frontier molecular orbitals of **5‐OMe·2H_2_O** are shown in Figure 6a. According to TDDFT calculations, excitation is centered on the BODIPY moiety, and the S_1_ state in all cases involves predominantly (>90%) a single configuration, either HOMO‐1→LUMO or HOMO→LUMO depending on the compound and solvation. For compound **5** in vacuum (Figure 6b), HOMO is on the BODIPY and HOMO‐1 is on the anthracene moiety, and, therefore, PET corresponds to the HOMO‐1→HOMO uphill jump by 0.07 eV, making it inefficient and preserving fluorescence. In MeCN (Figure 6b), the orbital order is inverted: HOMO‐1 is now localized on BODIPY (−5.66 eV) and HOMO on anthracene (−5.62 eV). Excitation of BODIPY creates a vacancy on HOMO‐1, and the ensuing PET is exergonic and fast.

**FIGURE 6:**
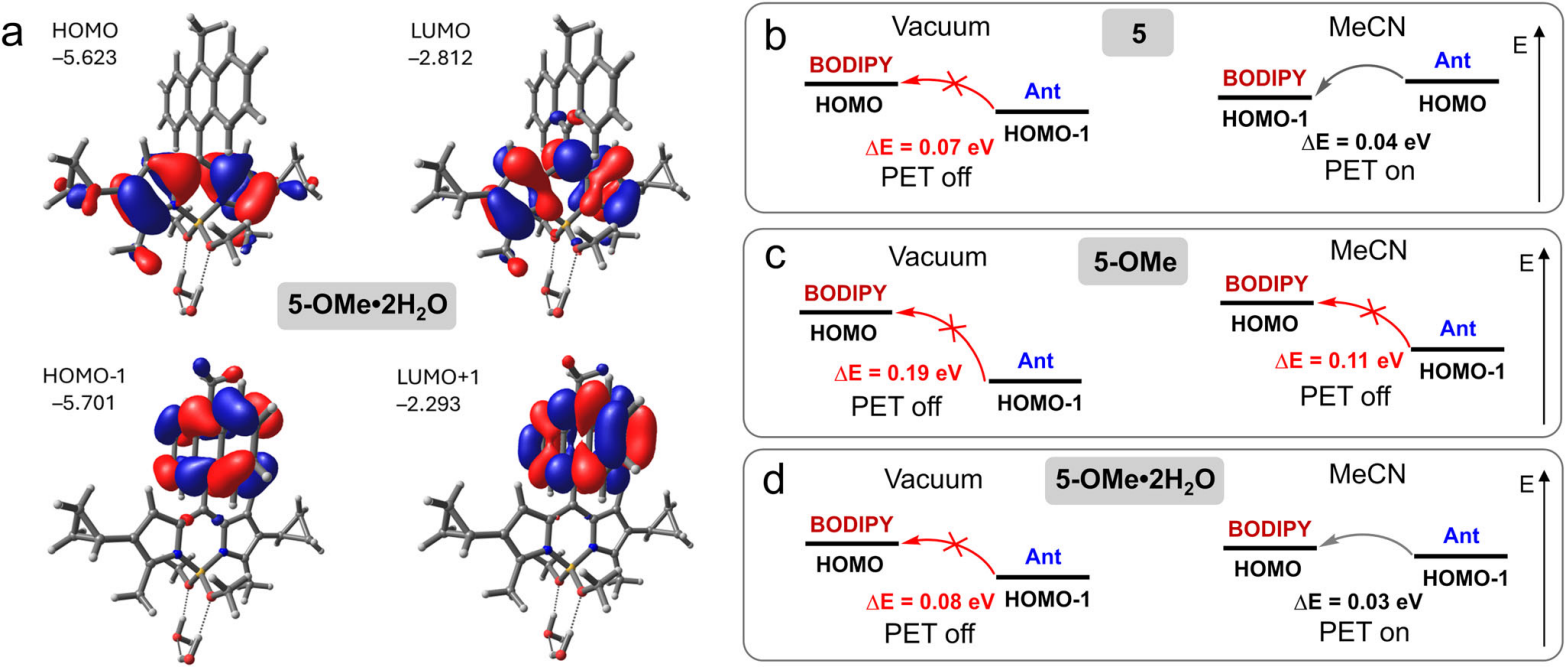
(a) Frontier molecular orbitals (energies given in eV) of **5‐OMe·2H_2_O** (two explicit water molecules hydrogen‐bonded to the alkoxy oxygens) in vacuum (B3LYP/6‐31+G(d,p)). (b–d) Energy‐level diagrams illustrating the HOMO–HOMO‐1 arrangements and PET activation for compounds **5**, **5‐OMe** and **5‐OMe·2H_2_O** in vacuum and in acetonitrile (SCRF model in Gaussian 16).

For **5‐OMe** in vacuum, HOMO‐1 remains on anthracene and HOMO on BODIPY (Figure 6c). PET from BODIPY to anthracene is uphill (0.19 eV) and hence inefficient. Therefore, the compound is expected to retain its fluorescence. In MeCN, the gap narrows to 0.11 eV, but PET remains unfavorable, consistent with experiment.

The critical change occurs upon hydration (Figure 6d). In **5‐OMe·2H_2_O** in vacuum, hydrogen bonding reduces the HOMO−HOMO‐1 gap to 0.08 eV, while in MeCN, the gap becomes even smaller, 0.03 eV. Given the proximity of the BODIPY and anthracene units and their respective orbitals, PET is likely to become fast in spite of remaining slightly endergonic, leading to fluorescence quenching.

Taken together, these computational results support the proposed mechanism: hydrogen bonding at the dialkoxyboron fragment reduce O→B donation, as evidenced by decreased B─O bond orders and reduced oxygen charge density, making the BODIPY core more electron‐accepting and facilitating PET under aqueous conditions.

### 2.5 5‐TEG as Fluorescence Lifetime Imaging Probe in Living Cells

Fluorescence lifetime imaging microscopy (FLIM) enables quantitative mapping of the intracellular microenvironment through changes in excited‐state lifetime, with applications ranging from metabolic imaging via NAD(P)H autofluorescence to biosensing and super‐resolution microscopy [51, 52]. Imaging intracellular hydrophobicity by FLIM is of particular interest for studying lipid organization, macromolecular crowding, and the biophysical determinants of cell function [53, 54, 55]. Small‐molecule FLIM probes are advancing rapidly [56], but hydration‐sensitive probes remain scarce despite growing interest in intracellular water organization [57]. Given that **5‐TEG** fluorescence with lifetimes spanning a ∼6 ns range, we evaluated its performance as a FLIM probe in live cells.

With human colorectal carcinoma HCT116 cells stained with concentrations 0.5–9 µM, we found exceptionally high intracellular brightness of **5‐TEG**, enabling for high‐resolution live FLIM. Two‐exponential fitting of the **5‐TEG‐**stained cells (9 µM) global decays resulted in mean and intensity‐weighted lifetimes of ⟨τ⟩ = 3.37 ns and 2.78 ns, respectively (Table S14). Across the full tested concentration range, lifetime decreased with increasing dye concentration, with the longest value (⟨τ⟩ = 6.1 ns) observed at 0.5 µM (Figure 7a–c and Table S14s). Interestingly, higher staining concentrations (9, 4.5 µM), revealed pronounced intracellular lifetime heterogeneity (Figure 7a,e), resulting in visualising distinct intracellular structures having median lifetime populations of 1.9, 2.6, and 4 ns; these could be assigned, based on morphology, to different subcellular compartments including plasma membrane, Golgi, and ER (Figure S18a–c). Observed bright intracellular staining with long fluorescence lifetimes indicate that **5‐TEG** potentially binds to lipophilic structures such as lipids, intrinsically disordered proteins and macromolecular condensates. This is consistent with the BSA and SDS‐micelle experiments, where association of **5‐TEG** with these environments enhances fluorescence and prolongs the lifetime.

**FIGURE 7:**
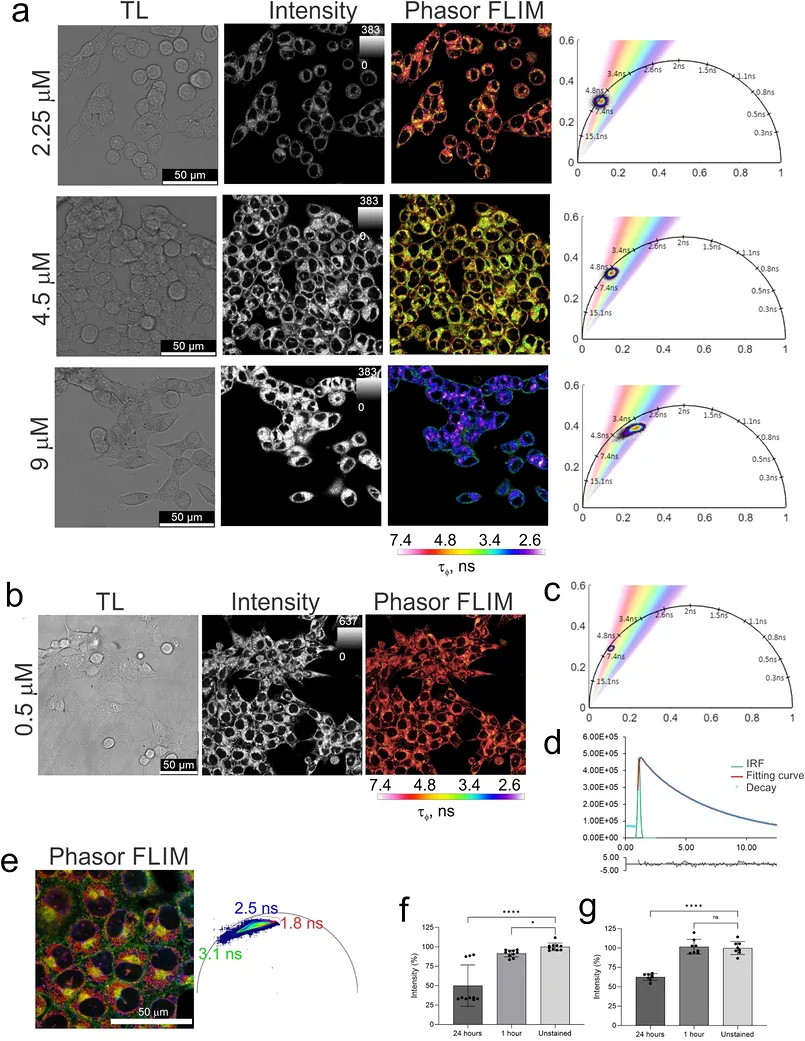
Microscopy of human carcinoma HCT116 adherent cells stained with **5‐TEG**. (a, b) Effect of staining concentration on the intracellular **5‐TEG** fluorescence lifetimes. (a) Cell monolayers were labeled with 9, 4.5 and 2.25 µM **5‐TEG** for 1 h using identical microscopy imaging parameters. Image sections from left to right correspond to TL (transmission light image), intensity image and Phasor‐FLIM τ_φ_‐color coded images with corresponding phasor plots (GS‐Lab). (bd) Example of cell monolayer staining with 0.5 µM **5‐TEG** (1 h). From left to right: transmission light, intensity, Phasor‐FLIM τ_φ_‐color coded images, (c) corresponding phasor plot with τ_φ_—projection (GS‐Lab) and (d) an example of fluorescence decay collected for the cell monolayers loaded with 0.5 µM **5‐TEG**. (e) Intracellular **5‐TEG** shows heterogeneity of lifetime values (from left to right: transmission light, Phasor‐FLIM τ_φ_‐color coded images with corresponding phasor plot. The triangle color coding mask with lifetime fixed at 3.1, 2.5, and 1.8 ns applied in LAS X software (median filter 11, threshold 10, harmonic 1). Scale bar is 50 µm. (f–g) Concentration‐ and loading time effects of **5‐TEG** on total cellular ATP levels in HCT116 cells. (f) 9 µM of **5‐TEG**; (g) 0.5 µM of **5‐TEG**.

Sequential imaging with multiphoton NAD(P)H autofluorescence FLIM also demonstrated compatibility of **5‐TEG** with optical metabolic imaging (Figure S18d) [58]. **5‐TEG** also displayed minor or no toxicity reflected in cell ATP production (Figure 7f,g). We also tested storage stability of **5‐TEG** by repeating the staining experiment using a stock solution stored in DMSO (−20°C, 2 months). Lifetime values were marginally lower compared to the fresh sample (mean difference of 0.7 ns at 9–2.25 µM; 0.16 ns at 0.5 µM; Table S15 and Figure S19), likely reflecting a combination of storage effects, variability in cell loading, and residual pile‐up contributions. Importantly, the concentration‐dependent lifetime heterogeneity and subcellular structure resolution were fully preserved (Figure S19a,b), confirming no effect of the storage.

### 2.6 Fluorescence Lifetime Microscopy of 3D Multicellular Spheroid and Organoid Cultures With 5‐TEG

Conventional 2D cell cultures poorly recapitulate ‘niche‐like’ cell biochemical and biophysical microenvironment features of a real tumour or stem cell niche. Various 3D cell cultures, produced either via bioengineering approaches or through the intrinsic capacity of cells to self‐organize can help establishing more tractable in vitro models minimising the need for animal or in vivo experiments [59, 60, 61]. However, imaging of 3D cell cultures remains technically challenging due to the limited brightness, light penetration depth and scattering, and the difficulty of delivering fluorescent probes uniformly throughout a dense cell mass [62]. We tested if **5‐TEG** could help address such challenges using two widely used 3D cell models: multicellular spheroids, established for studying colorectal cancer metabolism [63, 64] and stem cell‐derived small intestinal organoids grown in Matrigel hydrogel (Figure 8) [65, 66]. Surprisingly, **5‐TEG** penetrated throughout the full depth of the dense cell mass of HCT116 spheroids, with strong signal down to at least 50–75 µm and no visible attenuation of intensity (Figure 8a–d), demonstrating exceptional in‐depth staining efficacy well‐suited for true live 3D imaging. FLIM revealed a gradient‐like distribution through the spheroid volume, with longer lifetimes resolved in the core. Such gradients may reflect differences of cell compactness, lipophilicity, oxygenation [61], cell death, metabolism [64, 65] or osmotic pressure gradients and prompt for further in‐depth study [67].

**FIGURE 8:**
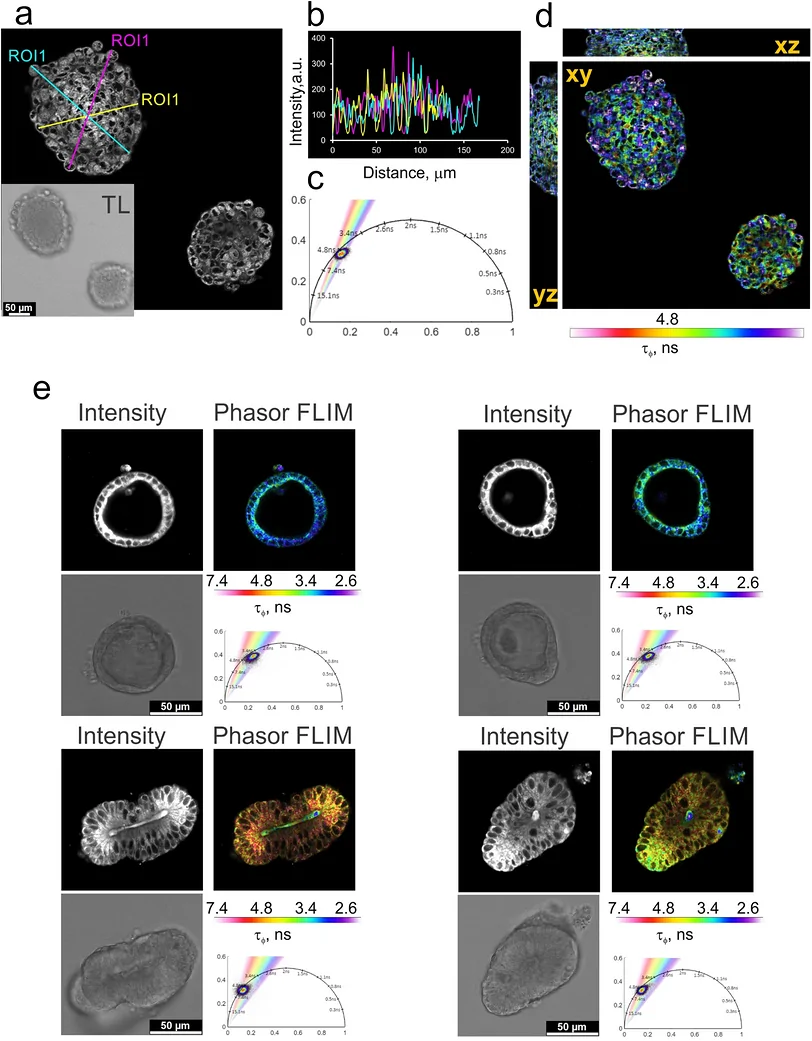
Staining of 3D cultures (HCT116 multicellular spheroids and small intestinal organoids) with 5‐TEG. (a, b) Intensity profile (cross‐section at 57 µm depth from the top of the spheroid) of 5‐TEG within stained multicellular spheroid (a). ROI 1‐3 reflect 3 individual line profiles (indicated with magenta, cyan and yellow), which correspond to the intensity profiles on the graph (b). The intensity profiles display elevated intensity in the centre of spheroid section. TL: transmission light image, scale bar is 50 µm. (c, d) Phasor plot with τ_φ_—projection (GS‐Lab; (c) with corresponding 3D Phasor‐FLIM reconstruction of xy, xz, and yz axes slice view (d) of the multicellular spheroid (from section a). (e) 5‐TEG staining of mouse small intestinal organoids demonstrates heterogeneity of fluorescence lifetime values. Four examples of organoids with different 5‐TEG lifetime values are shown with corresponding intensity, Phasor‐FLIM, phasors with τ_φ_ projections and transmission light images (GS‐Lab). Scale bar is 50 µm.

**5‐TEG** also efficiently accumulated in Matrigel‐grown small intestinal organoids, revealing a heterogeneous lifetime distribution, invisible in conventional intensity imaging (Figure 8e), reminiscent to previously reported BODIPY diacids [34]. Notably, organoid‐to‐organoid lifetime differences were more pronounced than those observed between spheroids, potentially reflecting functional heterogeneity in cell‐type composition along the crypt‐villus axis and their differences in metabolism [68]. Within individual organoids, lifetime distributions ranged from uniform to clear intercellular gradients. The observed lifetime differences may in part reflect variations in local hydration, lipid organization, molecular binding, and other microenvironmental factors within the organoid.

Collectively, **5‐TEG** displayed high performance as a FLIM probe for 3D biological imaging, combining exceptional brightness, deep tissue penetration, and a broad lifetime range. Such lifetime‐based segmentation of subcellular compartments can potentially help visualising microenvironmental differences in complex 3D cultures. These capabilities, which are difficult to achieve simultaneously with conventional fluorescent probes [69, 70], open avenues for cell‐specific labeling [51], multiplexing with other solvatochromic dyes [71] and validation with other imaging modalities [72]. **5‐TEG** also allows for ultrafast staining of live cell cultures with subsequent lifetime‐specific segmentation of different subcellular structures (Figure S18b). These features enable unbiased investigation of dynamic intracellular processes including inter‐organelle communication, while minimizing light exposure and resulting in high‐quality, noise‐free imaging data for intensity and lifetime analysis [73, 74].

## 3 Conclusion

In summary, we identified dialkoxy‐substituted O‐BODIPYs as a platform for hydration‐gated excited‐state charge transfer, providing a rare example of PET modulation dictated by aqueous hydration rather than by general solvent polarity. Replacing B–F with B–OR groups attenuates the reduction potentials of the BODIPY core, suppressing PET in polar organic solvents while enabling efficient charge separation in water. This behavior arises from hydrogen bonding at the alkoxy oxygens, which weakens O→B electron donation and strengthens the acceptor character of the BODIPY, switching the PET driving force, consistent with electrochemical measurements, transient absorption spectroscopy, and DFT calculations. The mechanism is general across TEG‐functionalized dyads spanning anthracene, pyrene, and carbazole donors and can be combined with BODIPY scaffold substitution to access red‐shifted emitters. The lead compound, **5‐TEG**, exhibits a hydration‐dependent lifetime span (≈6 ns) with negligible reactive oxygen species generation and functions as a bright FLIM probe. In 2D and 3D biological models, the lifetime contrast supports compartment‐level segmentation and mapping of microenvironmental heterogeneity with depth penetration beyond conventional probes. Overall, hydration‐gated PET in dialkoxy O‐BODIPYs enables lifetime‐encoded fluorescence probes for complex biological environments and provides a new design principle for dyes with pronounced sensitivity to local hydration.

## Author Contributions

**Venkata N. K. Mamillapalli**: investigation, visualization, writing – original draft. **Irina A. Okkelman**: writing – review and editing, visualization, investigation, writing – original draft. **Fatima Akhssas**: investigation, visualization, writing – review and editing. **Greta Sambucari**: investigation. **Angela C. Debruyne**: investigation. **Dinesh Beniwal**: investigation. **Christophe Bucher**: investigation, writing – review and editing, supervision, validation. **Mariangela Di Donato**: investigation, writing – original draft, visualization, supervision. **Ruslan I. Dmitriev**: funding acquisition, writing – review and editing, supervision. **Mikhail A. Filatov**: conceptualization, investigation, funding acquisition, writing – original draft, visualization, project administration, supervision.

## Conflicts of Interest

The authors declare no conflicts of interest.

## Supporting information



Synthetic procedures, NMR and mass spectra for new compounds, optical, electrochemical and computational data, materials and methods for FLIM and data analysis.**Supporting File**: anie75061‐sup‐0001‐SuppMat.pdf.

## Data Availability

The data that support the findings of this study are openly available in ChemRxiv at https://chemrxiv.org/, reference number 10.26434/chemrxiv.15004275/v2.
