# Supplementary material for: Hydration‐Gated Photoinduced Electron Transfer in BODIPY Dyes
Source: Angew Chem Int Ed Engl. 2026 Sep 29;65(46):e3726851. doi: 10.1002/anie.3726851 (PMC13749329; doi:10.1002/anie.3726851)
Supplement: Supplementary file 1 [file ANIE-65-e3726851-s001.pdf]

Supporting Information for:

Hydration-Gated Photoinduced Electron Transfer in BODIPY Dyes

Contents

1. General Procedures 2

2. Synthetic Procedures and Characterization Details 4

3. Optical Properties 9

4. Fluorescence Lifetime Imaging Microscopy 34

5. Electrochemical Data 40

6. NMR and High-Resolution Mass Spectra 53

7. Computational Data 70

8. References 82

1. General Procedures

^{1}H and ^{13}C NMR spectra were recorded on a Bruker Avance III 400 MHz spectrometer. Chemical shifts are reported as ppm relative to TMS and referenced to residual solvent peaks of $CDCl_3$ (δ 7.26 ppm), DMSO-d^6 (δ 2.50 ppm) and D_2O (δ 4.79 ppm). Multiplicity assignments are abbreviated as follows: s = singlet, d = doublet, t = triplet, q = quartet, dd = doublet of doublets, ddd = doublet of doublet of doublets, dt = doublet of triplets, m = multiplet.

UV-vis absorption spectra were recorded in solutions using Shimadzu UV-1900i and Perkin Elmer Lambda 1050 UV/VIS/NIR spectrometers (1 cm path length quartz cell). Fluorescence spectra were measured using FluoroMax Plus-C spectrometer. Emission quantum yields of the compounds were measured relative to the fluorescence of Rhodamine 6G as a standard (Φ_F = 0.95 in ethanol). Sample concentrations were chosen to obtain an absorbance of 0.03-0.07 at the excitation wavelength; at least three measurements were performed for each sample.

Excited state lifetimes were determined by time correlated single photon counting (TCSPC) measurements using Horiba Scientific DeltaHub DH-HT High throughput TCSPC mo dule equipped with a DeltaDiode-510L pulsed laser diode with peak wavelength 510nm +/-10nm. Datastation v2.4 software was used for data collection, and analysis was conducted on Eztime software. Decay curves were fitted with mono-, bi- and triexponential functions, and the quality was determined by the χ^2 values. Sample concentrations ranged from 0.1 to 0.2 µM depending on the compound.

Steady state solution phosphorescence emission of $^{1}O_2$ was acquired on a FluoroMax™ Plus fluorimeter from HORIBA, equipped with 150W ozone-free xenon source and DSS-IGA020L liquid nitrogen cooled InGaAs detector (800-1550nm at liquid nitrogen temperature). Liquid samples of BODIPY were prepared in carbon disulfide as a solvent. Absorbance at the excitation wavelength was adjusted to 0.05-0.2. Solutions were transferred into quartz cuvettes and placed in the instrument's sample holder. Samples were excited at 520 nm. Excitation and emission slits were set to 2 and 29 nm, respectively. Diffuse light, scattering and harmonic phenomena were filtered off from with a longpass filter (830 nm) from Edmund Optics placed before the emission monochromator.

Transient absorption spectra were acquired using an apparatus based on a Ti:sapphire regenerative amplifier (Coherent Legend Elite), providing 40 fs pulses centred at 800 nm, with an average power of 3.2 W and 1 kHz repetition rate. The system was pumped by a commercial Ti:sapphire oscillator (Coherent Micra). The output of the amplifier was split to generate the pump and probe pulses. Pump pulses at 510 nm were produced by mixing the signal output of a commercial optical parametric amplifier (TOPAS, Light Commercial) with the 800 nm fundamental output in a BBO crystal. The probe and reference pulses were obtained by focusing a small portion of the laser output on a 3 mm thick CaF_2 plate. The polarization angle between the pump and probe beam was set to the magic angle by rotating a $\lambda/2$ plate. The measurements were performed in a quartz cell (2 mm optical path length), mounted on a movable stage to avoid photodegradation and multiple excitations of the sample. The data were analyzed with a global fitting procedure using the GLOTARAN package and applying a linear sequential model.[1]

Electrochemical measurements were performed using an acetonitrile (Acros Organics, extra-dry with molecular sieves), passed on an activated alumina column and conserved with molecular sieves. The electrolyte tetra-n-butylammonium bis(trifluoromethanesulfonyl)imide (TBATFSI) was synthesized, recrystallized and dried using standard procedures. Electrochemical measurements in organic medium were performed inside a glovebox under a nitrogen atmosphere, and in aqueous medium under argon. Cyclic voltammetry (CV), voltammetry with rotating disc electrodes (RDE), and Differential pulse voltammetry (DPV) were carried out in a standard three-electrode cell, using

Biologic SP-300 potentiostat, connected to an interfaced computer that employed EC-Lab (v.11.60) software. The reference electrodes were, in organic medium: $Ag/AgNO_3$ (10^{-2} M + TBATFSI 0.1 M in CH_3CN, and in aqueous medium: Ag/AgCl, separated from the analyzed solution by a sintered glass frit filled with the background solution. The auxiliary electrode was platinum wire separated from the analyzed solution by a sintered glass frit filled with the background solution. All curves were systematically recalibrated using Ferrocene as an internal reference. An automatic ohmic drop compensation procedure was systematically performed when using cyclic voltammetry. Glassy carbon electrode (Ø = 3 mm) working electrodes, were polished with 1 µm diamond paste before each recording. Voltamperometry with a rotating disk electrode (RDE), was carried out with a radiometer (CTV101 radiometer analytical) equipment at a rotation rate of 500 rpm using a glassy carbon RDE tip (Ø= 5 mm).

Mass spectrometry analysis (HRMS) was performed with a Q-Tof Premier Waters MALDI quadrupole time-of-flight (Q-TOF) mass spectrometer equipped with Z-spray electrospray ionization (ESI) and matrix assisted laser desorption ionization (MALDI) sources in positive mode with trans-2-[3-(4-tertbutylphenyl)-2-methyl-2-propenylidene]malononitrile as the matrix. ESI mass spectra were acquired in positive modes as required, using a Micromass TOF mass spectrometer interfaced to a Waters 2960 HPLC or a Bruker microTOF-Q III spectrometer interfaced to a Dionex UltiMate 3000 LC. Atmospheric pressure chemical ionization (APCI) experiments were performed on a Bruker microTOFQ III spectrometer interfaced to a Dionex UltiMate 3000 LC.

2. Synthetic Procedures and Characterization Details

The handling of all air/water sensitive materials was carried out using standard high vacuum techniques. Tetrahydrofuran and dichloromethane were distilled from $LiAlH_4$ and CaH_2, respectively. All other solvents were used as commercially supplied. Analytical thin layer chromatography was performed using silica gel 60 (fluorescence indicator F254, pre-coated sheets, 0.2 mm thick, 20 cm × 20 cm; Merck) plates and visualized by UV irradiation (λ = 254 nm). Column chromatography was carried out using silica gel 60 (0.063-0.2 mm) and activated neutral alumina.

Compounds **1**,[2] **2**,[3] **3**,[4] **4**,[2] **7**,[5] **8**[6] and **9**[7] were synthesized as described previously.

Procedure for the synthesis of pyrrole P1

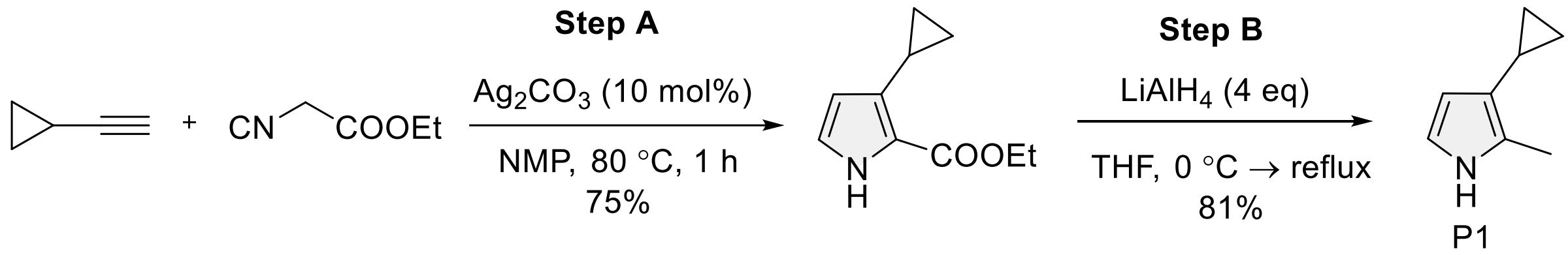

Step A. A modified literature procedure was used.[8] Ag_2CO_3 (0.14 g, 0.5 mmol) and NMP (25 mL) were added to a 100 mL round-bottom flask equipped with a nitrogen inlet and reflux condenser. Cyclopropylacetylene (0.43 mL, 5 mmol) was added to the mixture, which was heated to 80 °C. Ethyl isocyanoacetate (0.82 mL, 7.5 mmol) was then added, and heating was continued for 1 h. After completion of the reaction, the mixture was filtered and washed with DCM. The combined organic layers were washed with water, dried over sodium sulfate, concentrated under reduced pressure, and purified by silica gel column chromatography using 20% ethyl acetate/hexane to obtain the ethoxycarbonylpyrrole derivative in 75% yield (0.67 g). The ^1H NMR spectrum matched the literature data.

Step B. A modified literature procedure was used.[9] To an oven-dried two-neck round-bottom flask equipped with a nitrogen inlet and reflux condenser, $LiAlH_4$ (0.46 g, 12 mmol) and dry THF (30 mL) were added, and the mixture was cooled to 0 °C. The ester derivative prepared in Step A (0.54 g, 3 mmol) dissolved in dry THF (10 mL) was added dropwise to the reaction mixture, and the contents were refluxed overnight. After complete conversion, the reaction mixture was cooled to 0 °C and quenched with 10% aqueous NaOH solution. The reaction mixture was partitioned between water and ethyl acetate. The organic layer was dried over sodium sulfate, concentrated under reduced pressure, and used directly in the next step. A colorless liquid was obtained (0.29 g, 81% yield). ^1H NMR (400 MHz, $CDCl_3$) δ 7.70 (s, 1H), 6.54 (t, J = 2.7 Hz, 1H), 5.79 (t, J = 2.8 Hz, 1H), 2.28 (s, 3H), 1.65 (ddd, J = 13.5, 8.4, 5.1 Hz, 1H), 0.91–0.83 (m, 1H), 0.81–0.76 (m, 2H), 0.52–0.47 (m, 2H). ^{13}C NMR (101 MHz, $CDCl_3$) δ 124.56, 121.33, 115.07, 106.07, 29.85, 11.30, 7.10.

General procedure for the synthesis of 2,6-dicyclopropylBODIPYs

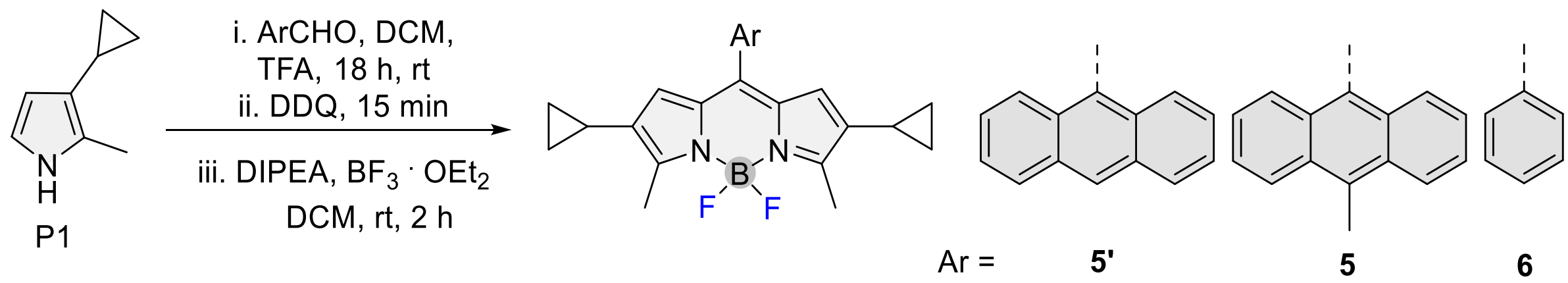

3-Cyclopropyl-2-methyl-pyrrole (P1) (0.178 g, 1.47 mmol) and the corresponding aldehyde (0.73 mmol) were dissolved in 20 mL of dry DCM and the solution was degassed by bubbling argon for 10

min. Trifluoroacetic acid (5 μL) was added and the reaction mixture was stirred for 18 h under argon. 2,3-Dichloro-5,6-dicyano-1,4-benzoquinone (DDQ) (0.083 g, 0.5 mmol) was added and the mixture was stirred for another 15 min. Subsequently the reaction mixture was transferred into a separation funnel and washed with water (2 × 20 mL). The aqueous phases were combined and extracted with 50 mL DCM. All organic phases were combined, dried over Na_2SO_4, filtered and evaporated in vacuum. The resulting solid was dissolved in 20 mL DCM and stirred under argon atmosphere. DIPEA (0.66 mL, 3.8 mmol) was added, and the mixture was stirred for another 5 min. Then boron trifluoride diethyl etherate (0.65 mL, 5.2 mmol) was added, the reaction flask was wrapped with aluminium foil, and the solution was stirred for 2 h. The reaction mixture was transferred into a separation funnel and washed with water (2 × 50 mL). The combined aqueous phases were extracted with 50 mL DCM. The organic phases were combined, dried over Na_2SO_4, filtered and evaporated in vacuum. The crude products were purified by silica gel column chromatography (EtOAc/hexane 1:5) to afford analytically pure compounds.

Compound **5′**: pink solid, 114 mg (33% yield). ^{1}H NMR (400 MHz, $CDCl_3$) δ 8.57 (s, 1H), 8.04 (d, *J* = 8.5 Hz, 2H), 7.84 (d, *J* = 8.8 Hz, 2H), 7.50 – 7.44 (m, 2H), 7.40 – 7.33 (m, 2H), 5.61 (s, 2H), 2.71 (s, 6H), 1.46 (dd, *J* = 9.0, 4.1 Hz, 2H), 0.70 – 0.63 (m, 4H), 0.25 – 0.18 (m, 4H). ^{13}C NMR (101 MHz, $CDCl_3$) δ 156.90, 137.28, 135.82, 134.69, 131.00, 128.42, 128.32, 127.79, 126.65, 126.45, 125.61, 124.01, 13.12, 7.24, 6.95. UV−vis (MeCN): λ_{max} [nm] (log ε) = 553 (4.58), 348 (3.92), 365 (4.11), 385 (4.11). HRMS (APCI): m/z found 476.2236, calcd for (M+) 476.2236.

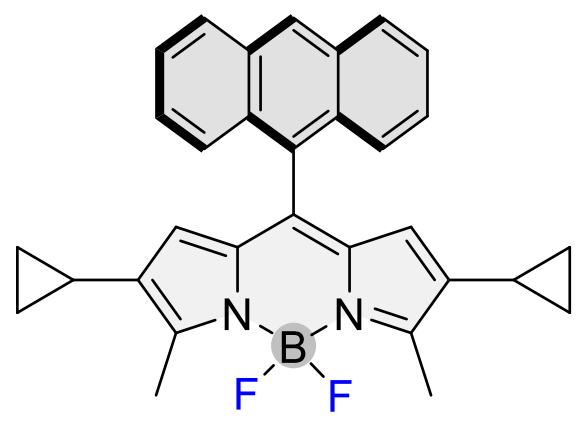

Compound **5**: pink solid, 129 mg (36% yield). ^{1}H NMR (400 MHz, $CDCl_3$) δ 8.35 (d, *J* = 8.9 Hz, 2H), 7.86 (d, *J* = 8.8 Hz, 2H), 7.54 – 7.48 (m, 2H), 7.40 – 7.34 (m, 2H), 5.61 (s, 2H), 3.20 (s, 3H), 2.71 (s, 6H), 1.46 (dt, *J* = 13.3, 5.1 Hz, 2H), 0.69 – 0.62 (m, 4H), 0.22 – 0.17 (m, 4H). ^{13}C NMR (101 MHz, $CDCl_3$) δ 156.75, 138.05, 135.75, 134.96, 132.42, 130.70, 129.50, 127.54, 126.43, 125.87, 125.48, 124.72, 124.08, 14.51, 13.10, 7.23, 6.95. UV−vis (MeCN): λ_{max} [nm] (log ε) = 553 (4.60), 356 (4.14), 374 (4.26), 394 (4.22). HRMS (APCI): m/z found 513.2278, calcd for (M+Na) $C_{32}H_{29}BF_2N_2Na$ 513.2284.

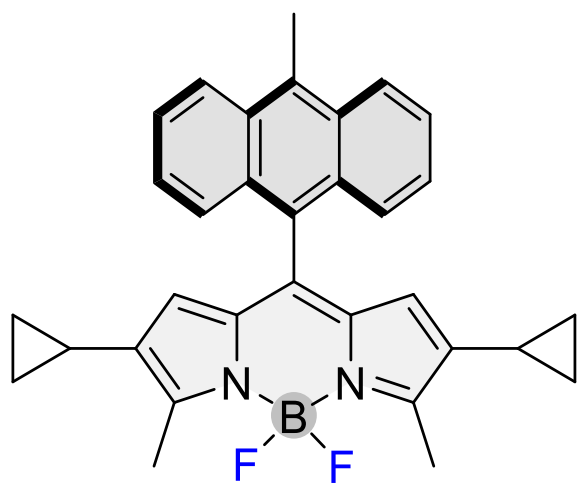

Compound **6**: pink solid, 77 mg (28% yield). ^{1}H NMR (400 MHz, $CDCl_3$) δ 7.46 (dt, *J* = 14.6, 6.8 Hz, 5H), 6.19 (s, 2H), 2.67 (s, 6H), 1.57 (dd, *J* = 11.6, 6.6 Hz, 2H), 0.83 (q, *J* = 6.1 Hz, 4H), 0.48 – 0.42 (m, 4H). ^{13}C NMR (101 MHz, $CDCl_3$) δ 156.50, 140.15, 135.38, 134.66, 132.92, 130.37, 129.66, 128.26, 124.56, 13.00, 7.33, 7.00. UV−vis (MeCN): λ_{max} [nm] (log ε) = 547 (4.51), 360 (3.92). HRMS (APCI): m/z found 399.1808, calcd for (M+Na) $C_{23}H_{23}BF_2N_2Na$ 399.1815.

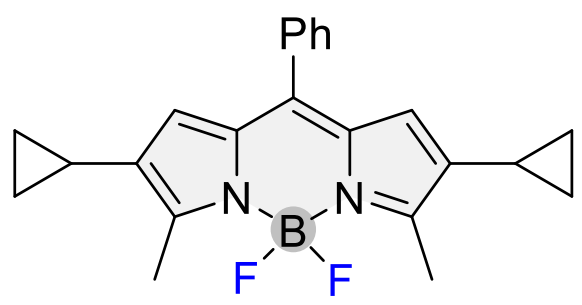

General procedure for the synthesis of TEG-substituted BODIPYs

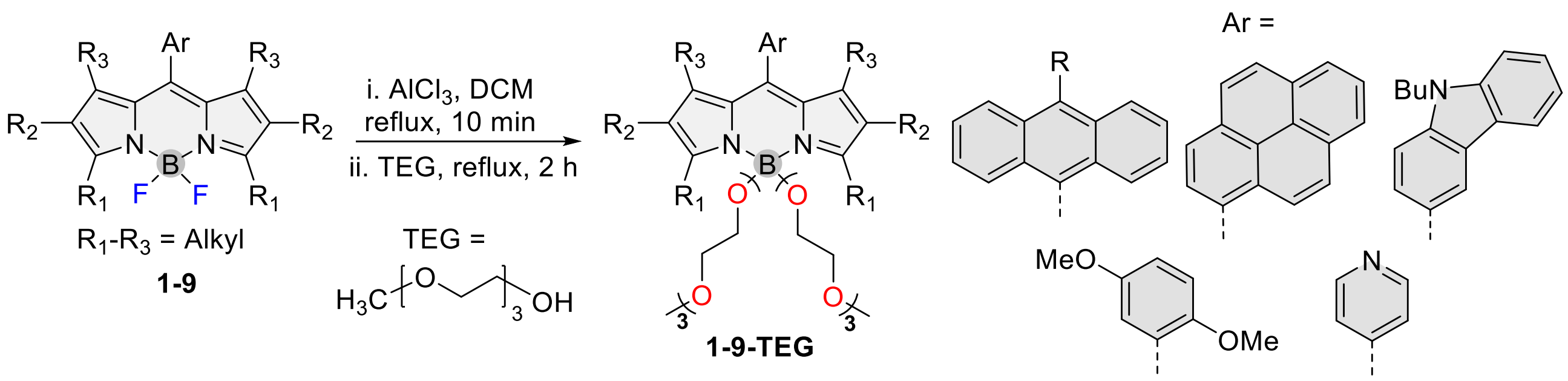

BDPs 1-9 (0.1 mmol) were dissolved in 10 mL of dry DCM, and the solution was degassed by bubbling argon for 10 min. Aluminium chloride (31 mg, 0.23 mmol) was then added and the solution refluxed

for 10 min, after which the triethylene glycol monomethyl ether (164 mg, 1 mmol) was added and refluxed further for 2 h under argon. Subsequently the reaction mixture was quenched with water, diluted with 20 mL of DCM and transferred into a separation funnel, washed with water (2 × 40 mL). The aqueous phases were combined and extracted with 30 mL DCM. All organic phases were combined, dried over Na_2SO_4, filtered and evaporated in vacuum. The crude products were purified by activated neutral alumina column chromatography (EtOAc to EtOAc/MeOH 10:1) to afford analytically pure compounds.

Compound **1-TEG**: orange solid, 37 mg (52% yield). ^{1}H NMR (400 MHz, DMSO) δ 8.83 (s, 1H), 8.19 (d, *J* = 8.3 Hz, 2H), 7.71 (d, *J* = 8.5 Hz, 2H), 7.54 (dt, *J* = 14.1, 6.7 Hz, 4H), 6.01 (s, 2H), 3.52 – 3.47 (m, 12H), 3.42 (dt, *J* = 9.3, 4.8 Hz, 8H), 3.23 (d, *J* = 4.4 Hz, 10H), 2.53 (s, 6H), 0.53 (s, 6H). ^{13}C NMR (101 MHz, DMSO) δ 155.83, 140.06, 137.74, 133.10, 130.97, 129.16, 128.74, 128.46, 128.09, 127.47, 125.91, 123.95, 121.12, 71.70, 71.31, 69.91, 69.80, 69.68, 60.63, 58.08, 14.43, 12.85. UV−vis (MeCN): λ_{max} [nm] (log ε) = 504 (4.90), 348 (4.02), 366 (4.15), 385 (4.10). HRMS (APCI): m/z found 735.3782, calcd for (M+Na) $C_{41}H_{53}BN_2NaO_8$ 735.3787.

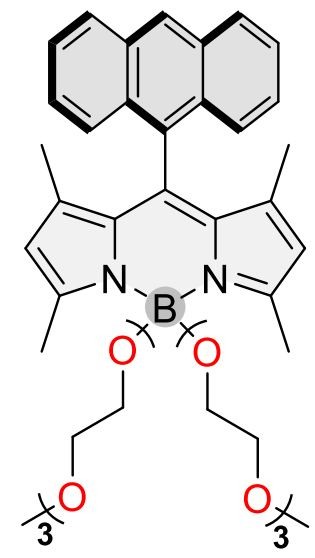

Compound **2-TEG**: orange semi-solid, 43 mg (58% yield). ^{1}H NMR (400 MHz, DMSO) δ 8.47 (d, *J* = 7.8 Hz, 1H), 8.40 (d, *J* = 6.9 Hz, 1H), 8.32 (d, *J* = 8.4 Hz, 3H), 8.21 (d, *J* = 9.3 Hz, 1H), 8.13 (t, *J* = 7.7 Hz, 1H), 7.99 (d, *J* = 7.8 Hz, 1H), 7.81 (d, *J* = 9.1 Hz, 1H), 6.06 (s, 2H), 3.54 – 3.38 (m, 22H), 3.24 (s, 3H), 3.22 (s, 3H), 3.14 (t, *J* = 5.3 Hz, 2H), 2.53 (s, 6H), 0.79 (s, 6H). ^{13}C NMR (101 MHz, DMSO) δ 155.67, 140.49, 139.63, 132.95, 131.23, 130.82, 130.43, 129.33, 129.14, 128.62, 128.20, 127.39, 126.83, 126.27, 126.07, 125.84, 125.69, 123.90, 123.72, 123.13, 121.18, 71.73, 71.71, 71.33, 69.92, 69.89, 69.81, 69.75, 69.69, 69.67, 60.60, 60.52, 58.10, 58.08, 14.43, 13.45. UV−vis (MeCN): λ_{max} [nm] (log ε) = 502 (4.80), 325 (4.46), 340 (4.58). HRMS (APCI): m/z found 759.3772, calcd for (M+Na) $C_{43}H_{53}BN_2NaO_8$ 759.3787.

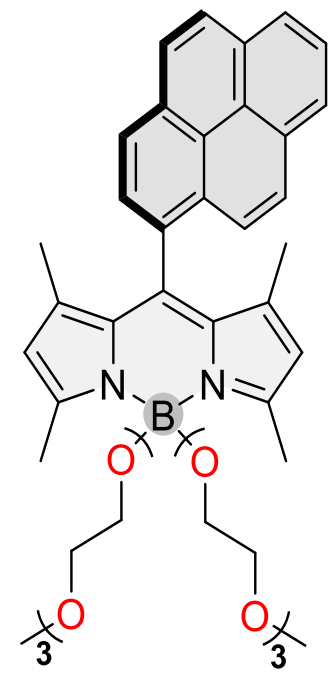

Compound **3-TEG**: yellowish-brown semi-solid, 42 mg (56% yield). ^{1}H NMR (400 MHz, DMSO) δ 8.26 (d, *J* = 7.7 Hz, 1H), 8.14 (d, *J* = 1.4 Hz, 1H), 7.78 (d, *J* = 8.4 Hz, 1H), 7.65 (d, *J* = 8.3 Hz, 1H), 7.52 – 7.46 (m, 1H), 7.33 (dd, *J* = 8.4, 1.7 Hz, 1H), 7.20 (t, *J* = 7.5 Hz, 1H), 6.07 (s, 2H), 4.46 (t, *J* = 7.1 Hz, 2H), 3.52 – 3.40 (m, 18H), 3.38 (d, *J* = 5.5 Hz, 2H), 3.23 (d, *J* = 1.4 Hz, 6H), 3.04 (dt, *J* = 12.9, 5.4 Hz, 4H), 2.48 (s, 6H), 1.79 (dt, *J* = 14.8, 7.2 Hz, 2H), 1.35 (dd, *J* = 15.1, 7.4 Hz, 2H), 1.25 (s, 6H), 0.90 (t, *J* = 7.4 Hz, 3H). ^{13}C NMR (101 MHz, DMSO) δ 154.85, 142.89, 140.72, 140.36, 140.09, 132.99, 126.19, 125.33, 124.87, 122.55, 122.08, 120.96, 120.83, 120.09, 119.08, 109.95, 109.56, 71.71, 71.65, 71.31, 69.89, 69.86, 69.76, 69.71, 69.66, 60.47, 60.37, 58.08, 42.27, 30.71, 19.84, 14.36, 14.26, 13.78. UV−vis (MeCN): λ_{max} [nm] (log ε) = 499 (4.87), 334 (3.95), 348 (3.95). HRMS (APCI): m/z found 780.4364, calcd for (M+Na) $C_{43}H_{60}BN_3NaO_8$ 780.4366.

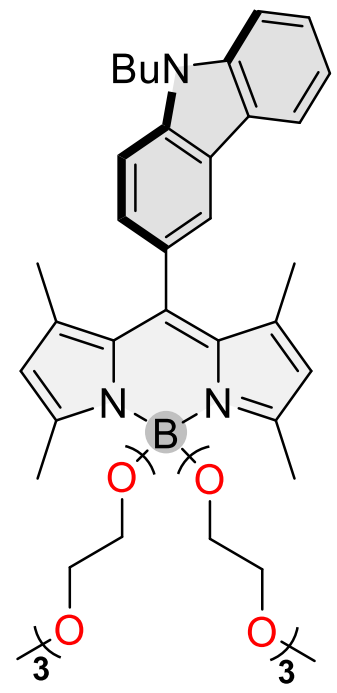

Compound **4-TEG**: green semi-solid, 38 mg (49% yield). ^{1}H NMR (400 MHz, DMSO) δ 8.46 (d, *J* = 8.9 Hz, 2H), 7.75 (d, *J* = 8.6 Hz, 2H), 7.62 – 7.56 (m, 2H), 7.53 – 7.47 (m, 2H), 3.52 – 3.45 (m, 12H), 3.43 – 3.38 (m, 8H), 3.22 (s, 6H), 3.19 (s, 4H), 2.52 (s, 6H), 2.15 (q, *J* = 7.4 Hz, 4H), 0.82 (t, *J* = 7.5 Hz, 6H), 0.45 (s, 6H). ^{13}C NMR (101 MHz, DMSO) δ 154.04, 136.79, 135.29, 132.59, 132.32, 131.89, 129.45, 129.12, 127.53, 126.79, 125.78, 125.32, 124.99, 71.65, 71.31, 69.91, 69.76, 69.68, 60.60, 58.08, 16.47, 14.72, 14.16, 12.19, 10.19. UV−vis (MeCN): λ_{max} [nm] (log ε) = 526 (4.63), 358 (3.89), 375 (4.03), 395 (4.00). HRMS (APCI): m/z found 805.4582, calcd for (M+Na) $C_{46}H_{63}BN_2NaO_8$ 805.4570.

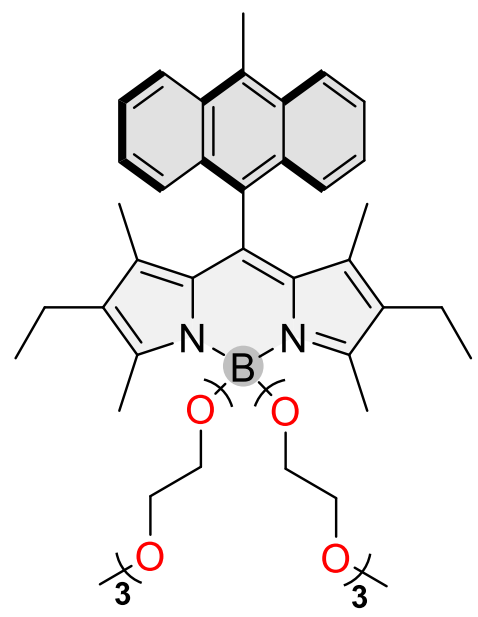

Compound **5-TEG**: pink semi-solid, 38 mg (49% yield). ^{1}H NMR (400 MHz, DMSO) δ 8.43 (d, *J* = 8.9 Hz, 2H), 7.65 (d, *J* = 8.6 Hz, 2H), 7.59 – 7.51 (m, 2H), 7.48 – 7.40 (m, 2H), 5.48 (s, 2H), 3.53 – 3.39 (m, 24H), 3.22 (s, 6H), 3.16 (s, 3H), 2.62 (s, 6H), 1.54 (td, *J* = 8.2, 4.1 Hz, 2H), 0.66 – 0.60 (m, 4H), 0.19 – 0.10 (m, 4H). ^{13}C NMR (101 MHz, DMSO) δ 156.31, 136.56, 135.59, 135.12, 132.41, 129.97, 129.00, 126.24, 126.16, 126.13, 125.46, 125.17, 121.17, 71.63, 71.32, 69.92, 69.78, 69.69, 60.74, 58.09, 14.16, 12.48, 7.61, 6.73. UV−vis (MeCN): λ_{max} [nm] (log ε) = 553 (4.60), 356 (3.99), 374 (4.17), 395 (4.14). HRMS (APCI): m/z found 801.4251, calcd for (M+Na) $C_{46}H_{59}BN_2NaO_8$ 801.4257.

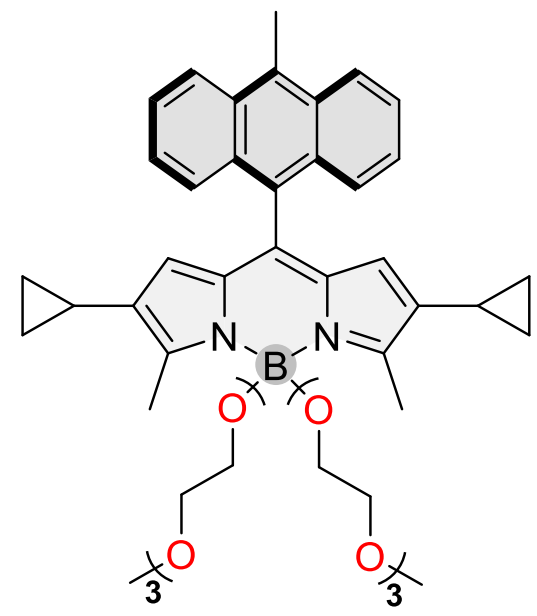

Compound **6-TEG**: pink semi-solid, 31 mg (46% yield). ^{1}H NMR (400 MHz, DMSO) δ 7.51 (dd, *J* = 12.7, 5.3 Hz, 3H), 7.44 (dd, *J* = 8.0, 1.5 Hz, 2H), 6.12 (s, 2H), 3.46 (ddd, *J* = 11.1, 8.1, 5.0 Hz, 16H), 3.30 (t, *J* = 5.4 Hz, 4H), 3.21 (s, 6H), 2.96 (t, *J* = 5.3 Hz, 4H), 2.57 (s, 6H), 1.68 – 1.62 (m, 2H), 0.81 (dd, *J* = 8.3, 2.1 Hz, 4H), 0.46 – 0.41 (m, 4H). ^{13}C NMR (101 MHz, DMSO) δ 155.88, 139.29, 134.65, 134.20, 133.38, 130.26, 129.68, 128.32, 121.97, 71.51, 71.30, 69.85, 69.81, 69.67, 69.64, 60.44, 58.07, 12.41, 7.79, 6.79. UV−vis (MeCN): λ_{max} [nm] (log ε) = 547 (4.56), 347 (3.87). HRMS (APCI): m/z found 687.3773, calcd for (M+Na) $C_{37}H_{53}BN_2NaO_8$ 687.3787.

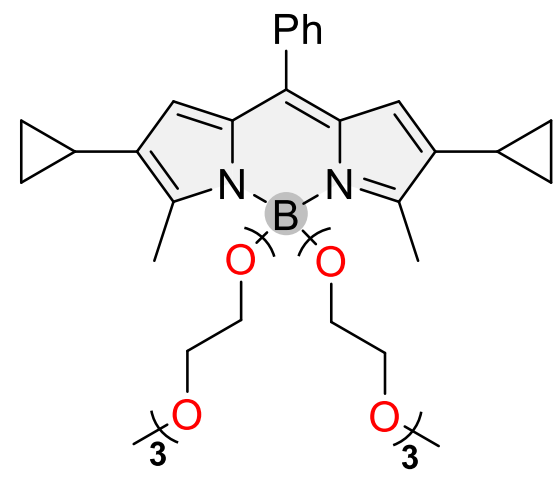

Compound **7-TEG**: greenish-brown semi-solid, 37 mg (69% yield). ^{1}H NMR (400 MHz, DMSO) δ 7.48 (s, 1H), 6.12 (s, 2H), 3.48 – 3.40 (m, 16H), 3.25 (t, *J* = 5.5 Hz, 4H), 3.22 (s, 6H), 2.85 (t, *J* = 5.4 Hz, 4H), 2.41 (s, 6H), 2.24 (s, 6H). ^{13}C NMR (101 MHz, DMSO) δ 156.15, 139.83, 134.41, 121.51, 118.58, 71.55, 71.32, 69.86, 69.70, 69.66, 60.37, 58.11, 14.36, 11.04. UV−vis (MeCN): λ_{max} [nm] (log ε) = 503 (4.58), 364 (3.47). HRMS (APCI): m/z found 559.3163, calcd for (M+Na) $C_{27}H_{45}BN_2NaO_8$ 559.3161.

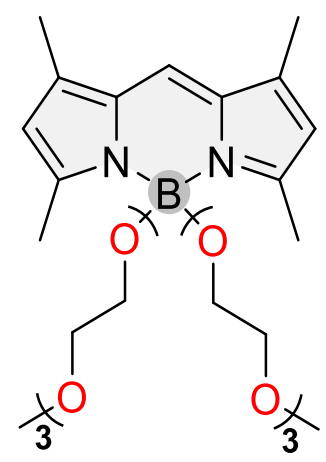

Compound **8-TEG**: yellowish-brown semi-soild, 32 mg (48% yield). ^{1}H NMR (400 MHz, DMSO) δ 7.11 (d, *J* = 9.1 Hz, 1H), 7.05 (dd, *J* = 9.0, 3.0 Hz, 1H), 6.73 (s, 1H), 6.04 (s, 2H), 3.73 (s, 3H), 3.65 (s, 3H), 3.50 – 3.38 (m, 18H), 3.34 – 3.24 (m, 6H), 3.22 (s, 6H), 3.03 – 2.95 (m, 4H), 2.44 (s, 6H), 1.42 (s, 6H). ^{13}C NMR (101 MHz, DMSO) δ 154.72, 154.08, 149.98, 140.18, 132.19, 120.64, 71.55, 71.30, 69.87, 69.72, 69.65, 69.60, 60.17, 58.08, 56.31, 55.75, 40.15, 39.94, 39.73, 39.52, 39.31, 39.10, 38.89, 14.35, 13.58. UV−vis (MeCN): λ_{max} [nm] (log ε) = 502 (4.69), 364 (3.49). HRMS (APCI): m/z found 673.3866, calcd for (M+H) $C_{35}H_{54}BN_2O_{10}$ 673.3867.

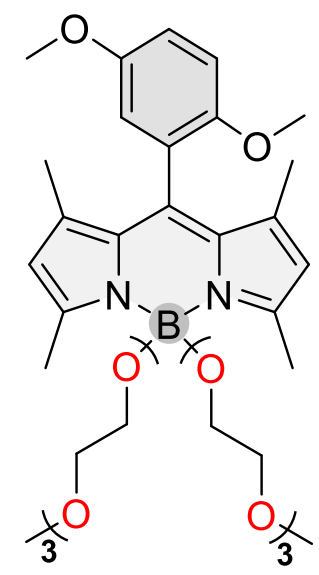

Compound **9-TEG**: yellowish-brown semi-solid, 34 mg (56% yield). ^{1}H NMR (400 MHz, DMSO) δ 8.75 (dd, *J* = 4.3, 1.6 Hz, 2H), 7.45 (dd, *J* = 4.3, 1.6 Hz, 2H), 6.12 (s, 2H), 3.49 – 3.44 (m, 8H), 3.42 – 3.39 (m, 10H, overlapping with solvent), 3.32 (t, *J* = 5.4 Hz, 4H, , overlapping with solvent), 3.22 (s, 3H), 2.98 (t, *J* = 5.4 Hz, 6H), 2.46 (s, 3H), 1.34 (s, 6H). ^{13}C NMR (101 MHz, DMSO) δ 156.01, 150.50, 142.97, 140.33, 137.91, 131.14, 123.53, 121.48, 71.61, 71.32, 69.86, 69.71, 69.65, 60.40, 58.09, 14.39, 14.26. UV−vis (MeCN): λ_{max} [nm] (log ε) = 502 (4.44), 364 (3.23). HRMS (APCI): m/z found 614.3607, calcd for (M+H) $C_{32}H_{49}BN_3O_8$ 614.3601.

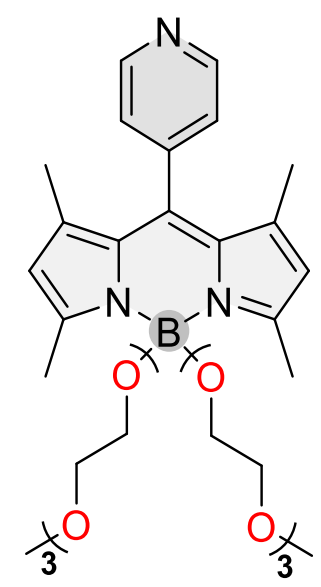

Compound **1-OMe** was prepared following the same procedure as above and methanol (32 mg, 1 mmol) was used instead of triethylene glycol monomethyl ether.

Compound **1-Ome:** orange solid, 23 mg (52% yield). ^{1}H NMR (400 MHz, DMSO) δ 8.83 (s, 1H), 8.19 (d, *J* = 8.0 Hz, 2H), 7.72 (d, *J* = 8.5 Hz, 2H), 7.54 (ddt, *J* = 14.9, 7.9, 3.4 Hz, 4H), 6.03 (s, 2H), 3.00 (s, 6H), 2.49 (s, 6H), 0.54 (s, 6H). ^{13}C NMR (101 MHz, DMSO) δ 155.25, 140.02, 137.99, 133.36, 130.98, 129.15, 128.73, 128.45, 128.11, 127.51, 125.92, 123.95, 121.05, 48.84, 14.32, 12.88. UV−vis (MeCN): λ_{max} [nm] (log ε) = 503 (5.03), 348 (4.12), 365 (4.26), 385 (4.20). HRMS (APCI): m/z found 471.2210, calcd for (M+Na) $C_{29}H_{29}BN_2NaO_2$ 471.2214.

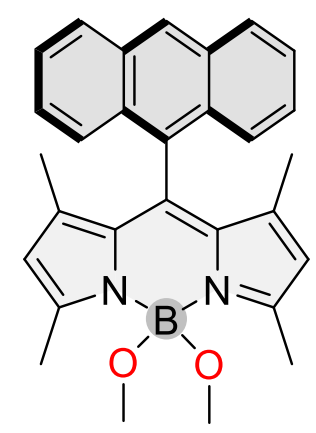

3. Optical Properties

Table S1. Fluorescence quantum yields (Φ_F) and lifetimes (τ) of selected reported O-BODIPY dyes in different solvents. Colour code: organic solvent, aqueous media. THF – Tetrahydrofuran, TOL – Toluene, PBS – Phosphate buffered saline, DCM – dichloromethane, SPB – Sodium phosphate buffer.

Entry	Structure	Solvent	Φ_F	τ_F, ns	Reference
**4,4-Dialkoxy-BODIPY**					
1	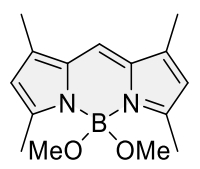	H_2O	0.933±0.140	–	10
2	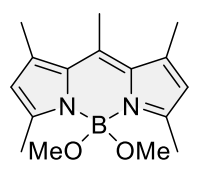	H_2O	0.35	–	11
3	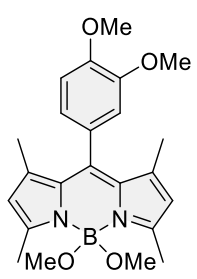	THF	0.42	–	12
		TOL	0.57	–	
		H_2O	0.051±0.008	–	3
4	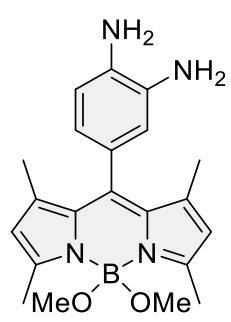	SPB	0.012	–	13
5	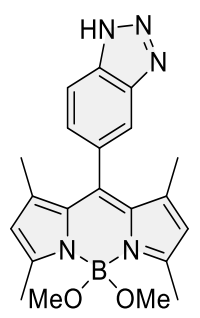	SPB	0.496	–	13
6	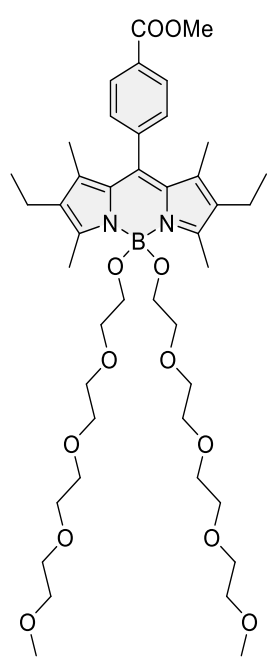	H_2O	0.49	4.68	14
7	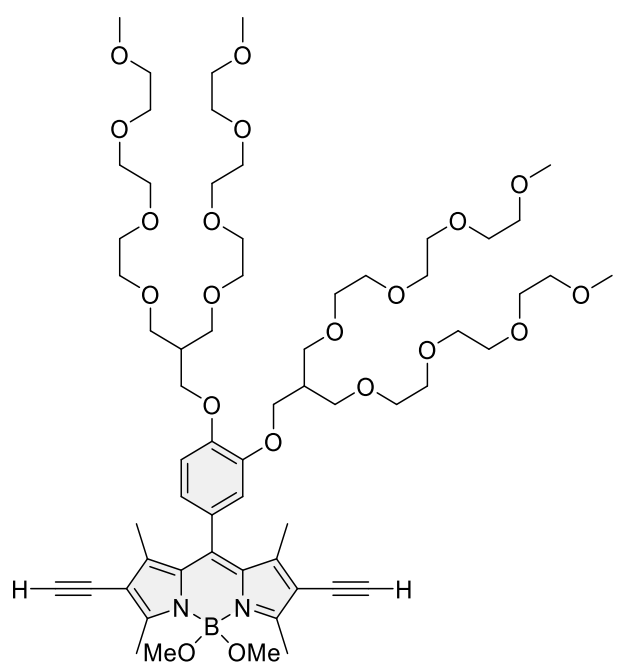	DCM	0.514	–	15
		H_2O	0.089	–	

**4,4-Diaryloxy-BODIPY**					
8	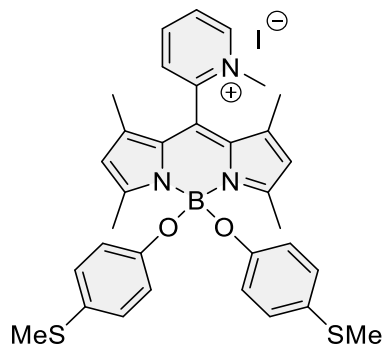	PBS	0.0013	–	16
**4,4-Bis(alkoxycarbonyl)-BODIPY and mixed systems**					
9	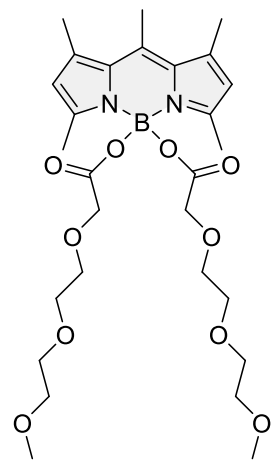	MeOH	0.91	6.23	17
		H_2O	0.73	6.20	
10	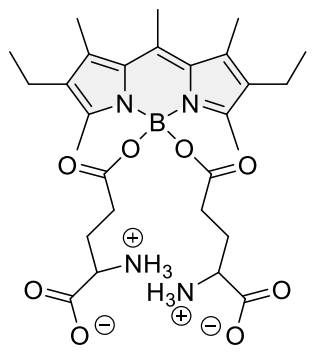	MeOH	0.83	6.67	
		H_2O	0.80	6.96	
11	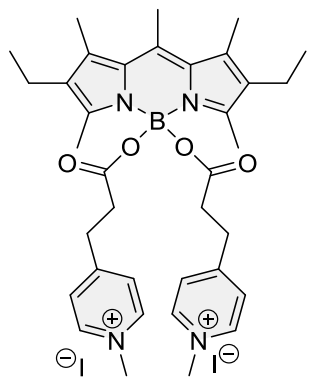	MeOH	0.61	5.44	
		H_2O	0.63	5.55	
12	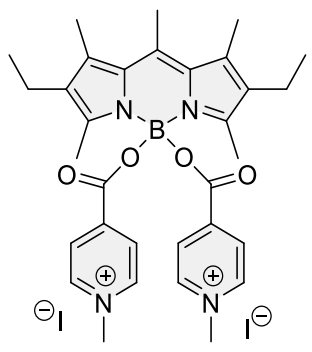	MeOH	0.67	6.29	
		H_2O	0.41	6.14	
13	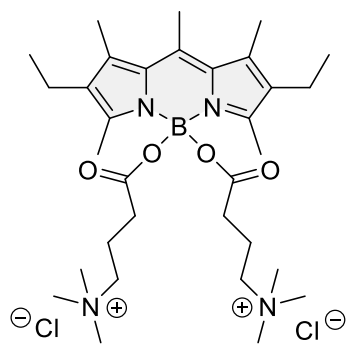	MeOH	0.74	6.87	
		H_2O	0.71	6.89	
14	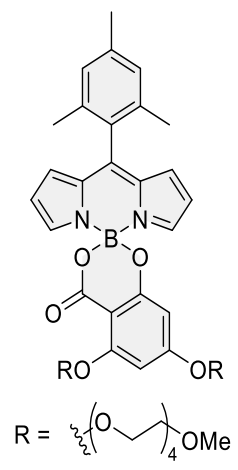	$CHCl_3$	0.17	0.45	18
		EtOH	0.03	<0.15	
		H_2O	<0.02	<0.15	
15	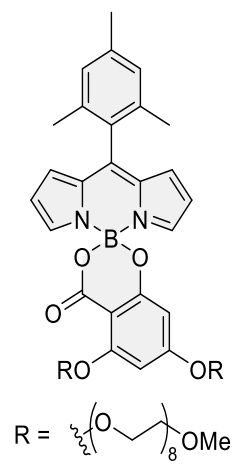	$CHCl_3$	0.06	0.49	
		EtOH	0.02	0.11	
		H_2O	<0.01	<0.15	

16	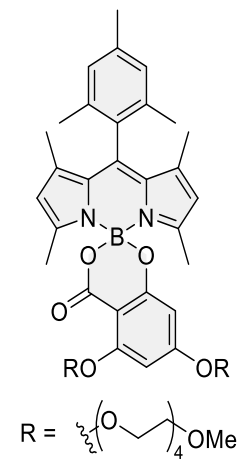	$CHCl_3$	0.88	6.04	
		EtOH	0.85	6.58	
		H_2O	0.66	6.86	
17	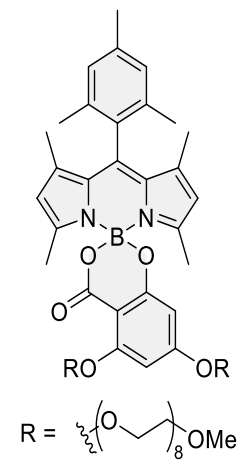	$CHCl_3$	0.86	5.82	
		EtOH	0.79	6.6	
		H_2O	0.78	6.4	
18	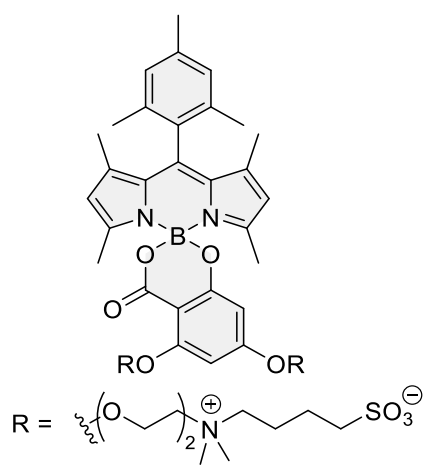	$CHCl_3$	0.71	5.72	
		EtOH	0.84	5.99	
		H_2O	0.85	6.31	
19	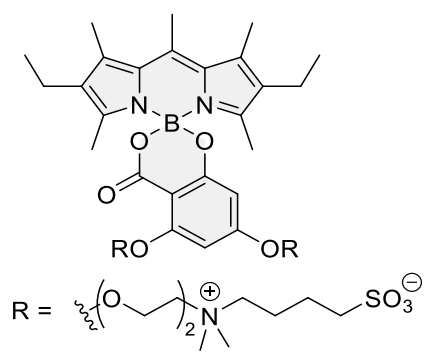	$CHCl_3$	0.72	6.87	
		EtOH	0.76	7.39	
		H_2O	0.78	7.5	
20	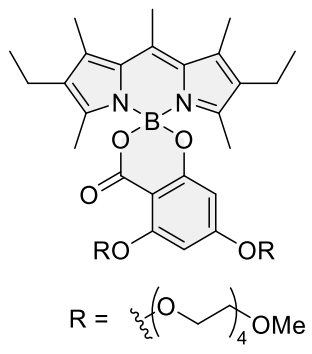	$CHCl_3$	0.72	6.99	
		EtOH	0.75	7.42	
		H_2O	0.71	7.55	
21	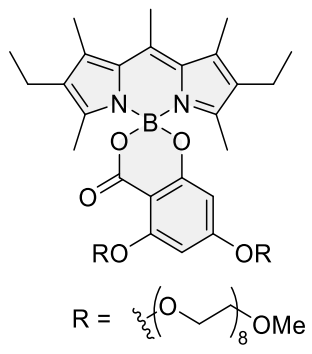	$CHCl_3$	0.73	6.85	
		EtOH	0.72	7.28	
		H_2O	0.71	7.77	
**4-Fluoro-4-alkoxy-BODIPY**					
22	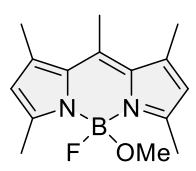	H_2O	0.33	–	11
23	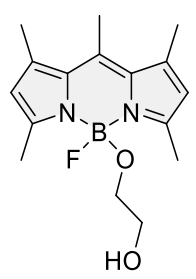	H_2O	0.47	–	
24	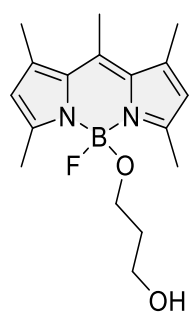	H_2O	0.35	–	

25	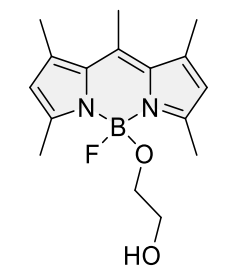	H_2O	0.47	–	
26	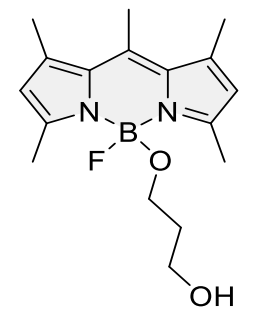	H_2O	0.35	–	
27	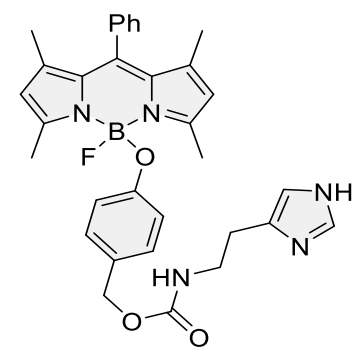	SPB	0.13	–	19
28	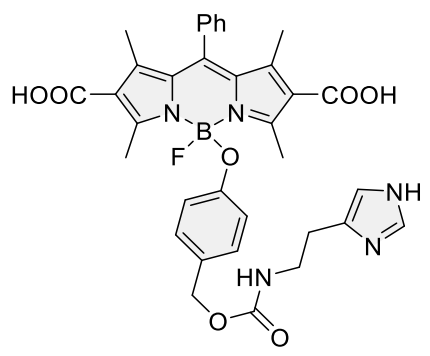	SPB	0.11	–	

Table S2. Chemical structures, fluorescence quantum yields (Φ_F) and lifetimes (τ) of representative polarity-sensitive dyes and previously reported water-soluble BODIPYs.

Entry	Structure	Solvent	Φ_F	τ_F, ns (χ^2)	Reference
1	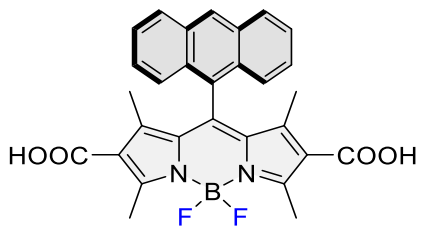	EtOH	0.045	2.99, 4.91 (1.04)	20
		MeOH	0.042	2.43, 4.65 (1.15)	
		MeCN	0.028	1.82, 4.15 (1.16)	
		DMF	0.027	2.06, 4.06 (1.16)	
		DMSO	0.013	1.11, 3.97 (1.36)	
		H_2O	0.019	1.709	
2	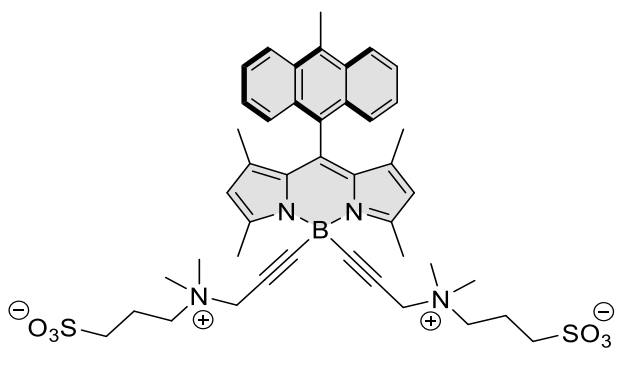	EtOH	0.025	0.10, 2.39, 5.58 (1.12)	21
		MeOH	0.014	2.05, 5.82 (1.09)	
		MeCN	0.009	2.41, 5.96 (1.2)	
		DMF	0.018	2.61, 5.56 (1.22)	
		DMSO	0.015	2.86, 4.77 (1.07)	
		H_2O	0.01	0.29, 2.7, 6.34 (1.14)	

Table S3. Optical data of TEG-BODIPYs compared to their parent F-BODIPYs.

Compound	Solvent[a]	λ_{abs}	λ_{em}[b]	Φ_F[b]	Lifetime (χ^2)
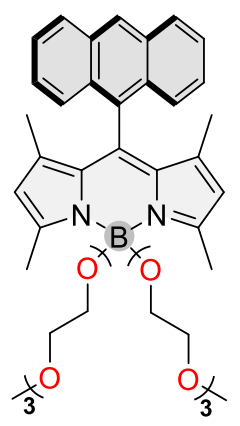 1- TEG	CYH	507	517	0.903	5.41 (1.1)
	TOL	508	519	0.905	4.75 (1.04)
	DCM	507	518	0.847	5.53 (1.05)
	EtOH	505	517	0.374	5.68 (1.18)
	MeOH	505	516	0.381	4.81 (1.16)
	MeCN	504	515	0.444	4.86 (1.1)
	DMF	506	518	0.646	5.06 (1.18)
	DMSO	507	520	0.380	4.61 (1.13)
	Formamide	506	519, 620	0.026	2.74, 5.23 (1.22)
	H_2O	502	513	0.005	0.49, 2.09, 5.5 (1.04)
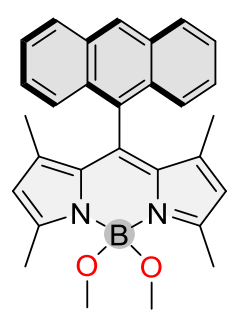 1-OMe	CYH	507	517	0.913	5.28 (1.11)
	TOL	508	518	0.941	4.64 (1.06)
	DCM	506	518	0.890	5.36 (1.05)
	EtOH	504	515	0.391	5.53 (1.04)
	MeOH	504	515	0.314	4.43 (1.05)
	MeCN	503	514	0.398	4.58 (1.2)
	DMF	506	518	0.569	5.03 (1.15)
	DMSO	507	520	0.365	4.57 (1.1)
	Formamide	505	517, 634	0.022	2.56, 5.25 (1.18)
	H_2O	501	512	0.005	0.31, 2.19, 5.08 (1.03)

Compound	Solvent	λ_{abs}	λ_{em}	Φ_F	Lifetime (χ^2)
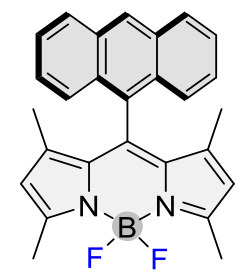 1	HEX	504	515	0.99	5.21 (1.06)
	TOL	508	521	0.92	4.74 (1.07)
	DCM	506	517	0.072	2.83, 5.22 (1.1)
	EtOH	505	513	0.038	1.23, 4.74 (1.35)
	MeOH	502	510	0.028	2.55, 5.53 (1.16)
	MeCN	502	512, 621	0.006	2.16, 5.08 (1.19)
	DMF	505	518, 615	0.011	3.15, 5.5 (1.08)
	DMSO	507	514, 645	0.010	3.4, 5.25 (1.03)

Compound	Solvent[a]	λ_{abs}	λ_{em}[b]	Φ_F[b]	Lifetime (χ^2)	Compound	Solvent	λ_{abs}	λ_{em}	Φ_F	Lifetime (χ^2)
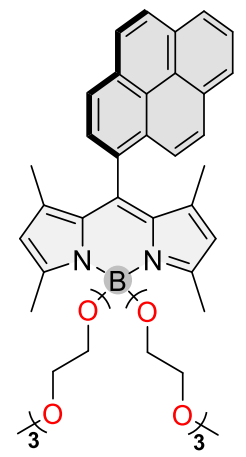 **2-TEG**	CYH	505	517	0.849	5.25 (1.09)	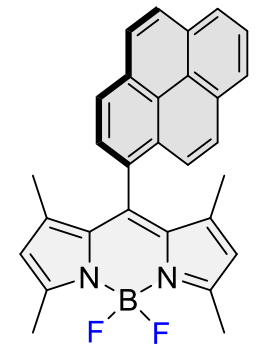 **2**	HEX	503	513	0.967	5.02 (1.13)
	TOL	506	518	0.862	4.68 (1.11)		TOL	507	516	0.926	4.62 (1.14)
	DCM	505	518	0.905	5.28 (1.04)		DCM	505	514	0.805	5.14 (1.07)
	EtOH	503	514	0.538	5.93 (1.13)		EtOH	502	511	0.651	4.94 (1.12)
	MeOH	503	514	0.683	6.01 (1.15)		MeOH	500	512	0.282	3.89 (1.16)
	MeCN	502	515	0.743	5.67 (1.31)		MeCN	501	509	0.126	4.06 (1.21)
	DMF	504	517	0.763	5.22 (1.17)		DMF	504	514	0.165	4.32 (1.45)
	DMSO	506	519	0.860	4.97 (1.26)		DMSO	505	517, 616	0.133	4.51 (1.16)
	Formamide	504	517	0.304	3.83 (1.34)						
	H_2O	501	512	0.003	0.30, 1.45, 4.39 (1.05)						
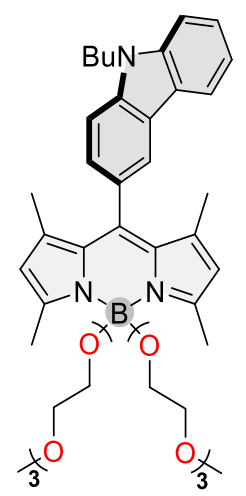 **3-TEG**	CYH	503	513	0.873	4.79 (1.13)	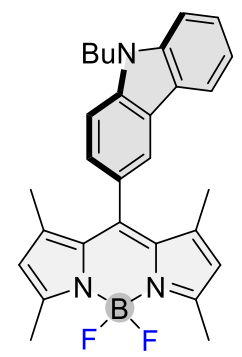 **3**	CYH	502	513	0.651[22]	3.97 (1.15)
	TOL	504	515	0.879	4.31 (1.11)		TOL	503	513	0.773	3.92 (1.14)
	DCM	502	513	0.894	4.81 (1.16)		DCM	500	512	0.633	4.32 (1.13)
	EtOH	500	511	0.498	5.33 (1.27)		EtOH	498	509	0.366	2.33, 4.43 (1.17)
	MeOH	499	511	0.590	4.94 (1.21)		MeOH	497	508	0.087	2.11, 5.41 (1.21)
	MeCN	499	510	0.573	4.64 (1.28)		MeCN	496	509	0.010[16]	3.34, 5.49 (0.99)
	DMF	501	513	0.688	4.65 (1.36)		DMF	500	511	0.071	3.25, 5.0 (1.15)
	DMSO	502	514	0.735	4.82 (1.32)		DMSO	501	512	0.049	2.41, 4.56 (1.15)
	Formamide	501	512	0.079	1.91, 5.5 (1.14)						
	H_2O	499	508, 742	0.007	0.14, 1.07, 4.97 (1.03)						

Compound	Solvent[a]	λ_{abs}	λ_{em}[b]	Φ_F[b]	Lifetime (χ^2)	Compound	Solvent	λ_{abs}	λ_{em}	Φ_F	Lifetime (χ^2)
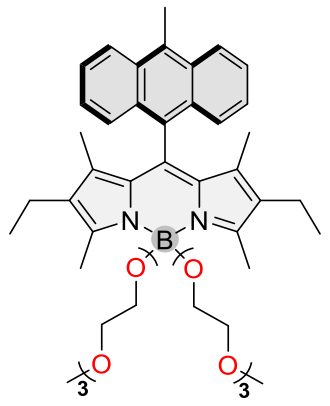 **4-TEG**	CYH	529	543	0.752	5.78 (1.13)	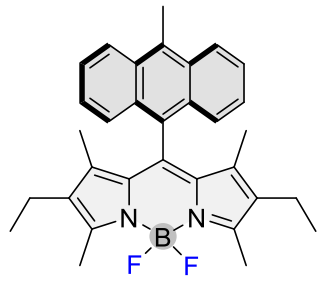 **4**	CYH	529	542	0.845	5.63 (1.17)
	TOL	530	544	0.671	5.17 (1.13)		TOL	531	544	0.898	5.16 (1.14)
	DCM	530	544	0.677	6.01 (1.11)		DCM	530	544	0.609	5.89 (1.11)
	EtOH	528	542	0.741	6.52 (1.19)		EtOH	527	539	0.261	2.43, 4.6 (1.15)
	MeOH	527	542	0.662	6.61 (1.14)		MeOH	526	540	0.076	2.1, 3.68 (1.12)
	MeCN	526	541	0.690	6.41 (1.11)		MeCN	526	539	0.031	0.14, 3.07, 6.02 (1.08)
	DMF	528	542	0.776	5.94 (1.16)		DMF	529	542	0.099	3.61, 5.69 (1.03)
	DMSO	529	545	0.744	5.48 (1.19)		DMSO	530	543	0.061	1.87, 4.59 (1.2)
	Formamide	529	544	0.190	3.72 (1.09)						
	H_2O	528	543	0.07	0.11, 1.9, 5.49 (1.11)						
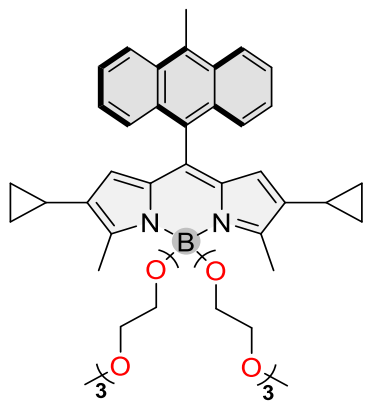 **5-TEG**	CYH	549	581	0.707	6.51 (1.24)	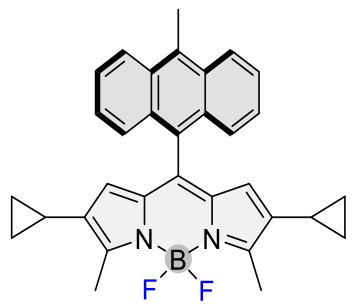 **5**	CYH	551	584	0.777	6.31 (1.2)
	TOL	555	587	0.750	5.92 (1.22)		TOL	556	589	0.711	5.78 (1.17)
	DCM	555	587	0.728	6.11 (1.3)		DCM	555	589	0.456	5.54 (1.21)
	EtOH	553	586	0.539	6.96 (1.3)		EtOH	551	586	0.188	2.68, 4.97 (1.14)
	MeOH	553	585	0.433	6.64 (1.16)		MeOH	551	583	0.062	1.49, 5.8 (1.18)
	MeCN	553	585	0.624	6.88 (1.37)		MeCN	553	583	0.032	1.89, 6.07 (1.13)
	DMF	555	588	0.553	6.38 (1.42)		DMF	557	587	0.107	2.5, 6.14 (0.98)
	Formamide	559	601	0.183	1.89, 6.81 (1.38)		DMSO	559	588	0.082	2.0, 5.71 (1.09)
	H_2O	565	609	0.021	0.13, 1.9, 5.49 (0.99)						

Compound	Solvent[a]	λ_{abs}	λ_{em}[b]	Φ_F[b]	Lifetime (χ^2)	Compound	Solvent	λ_{abs}	λ_{em}	Φ_F	Lifetime (χ^2)
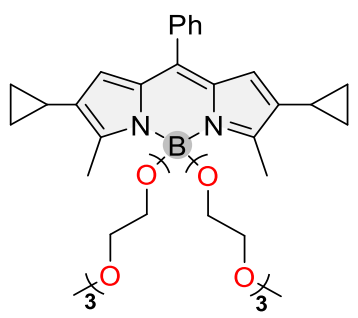 **6-TEG**	CYH	545	582	0.641	6.63 (1.34)	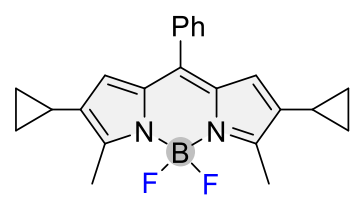 **6**	CYH	546	582	0.551	5.38 (1.16)
	TOL	549	590	0.660	6.13 (1.22)		TOL	551	589	0.616	5.33 (1.22)
	DCM	549	588	0.513	6.9 (1.42)		DCM	550	591	0.554	5.81 (1.18)
	EtOH	546	585	0.480	7.15 (1.19)		EtOH	545	586	0.465	5.64 (1.26)
	MeOH	546	586	0.524	7.4 (1.21)		MeOH	545	587	0.469	5.43 (1.09)
	MeCN	547	587	0.481	7.16 (1.16)		MeCN	547	589	0.410	5.1 (1.18)
	DMF	549	590	0.519	6.45 (1.08)		DMF	551	594	0.433	4.89 (1.22)
	DMSO	551	591	0.546	6.04 (1.49)		DMSO	552	596	0.431	4.82 (1.04)
	Formamide	551	591	0.527	6.31 (1.27)						
	H_2O	548	590	0.367	5.82 (1.29)						
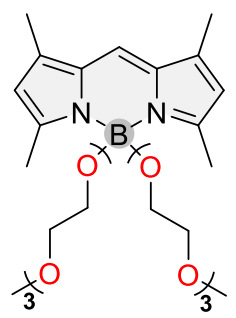 **7-TEG**	CYH	510	514	0.928	5.0 (1.1)	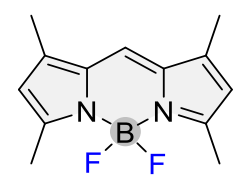 **7**	CYH	508	513	0.974	4.93 (1.04)
	TOL	510	516	0.976	4.48 (1.28)		TOL	510	518	0.968	4.58 (1.09)
	DCM	508	515	0.962	5.23 (1.23)		DCM	507	513	0.972	5.08 (1.03)
	EtOH	506	512	0.946	5.61 (1.12)		EtOH	504	511	0.952	5.28 (1.15)
	MeOH	505	512	0.969	5.86 (1.06)		MeOH	502	509	0.975	5.48 (1.05)
	MeCN	503	510	0.880	5.35 (1.18)		MeCN	501	508	0.942	5.3 (1.04)
	DMF	505	513	0.937	5.07 (1.18)		DMF	503	511	0.940	4.95 (1.24)
	DMSO	506	515	0.921	4.84 (1.17)		DMSO	504	513	0.933	4.67 (1.02)
	Formamide	506	514	0.933	5.35 (1.15)		H_2O	496	504	0.881	5.81 (1.04)
	H_2O	501	508	0.918	5.99 (1.03)						

Compound	Solvent[a]	λ_{abs}	λ_{em}[b]	Φ_F[b]	Lifetime (χ^2)	Compound	Solvent	λ_{abs}	λ_{em}	Φ_F	Lifetime (χ^2)
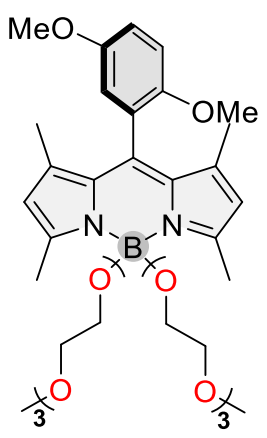<br>**8-TEG**	CYH	506	519	0.898	4.41 (1.19)	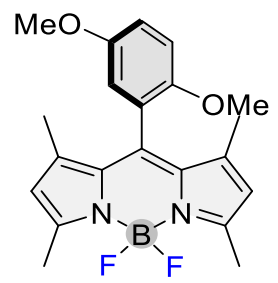<br>**8**	CYH	505	516	0.917	4.92 (1.26)
	TOL	507	521	0.964	4.45 (1.14)		TOL	506	519	0.967	4.54 (1.17)
	DCM	504	518	0.849	4.93 (1.22)		DCM	504	515	0.791	4.86 (1.25)
	EtOH	504	517	0.851	2.45, 5.68 (1.0)		EtOH	502	513	0.264	1.85, 3.62 (0.98)
	MeOH	503	516	0.678	5.02 (1.26)		MeOH	501	512	0.037	0.43, 3.92 (1.22)
	MeCN	502	514	0.750	4.71 (1.18)		MeCN	499	510	0.007	0.24, 1.37, 4.48 (1.11)
	DMF	505	518	0.725	4.75 (1.14)		DMF	503	515	0.011	0.28, 2.99 (1.15)
	DMSO	506	520	0.581	3.2 (1.18)		DMSO	504	516	0.005	0.04, 1, 4.3 (1.09)
	Formamide	504	518	0.052	0.33, 0.75, 4.27 (1.15)						
	H_2O	501	515	0.007	0.1, 1.87, 3.99 (1.02)						
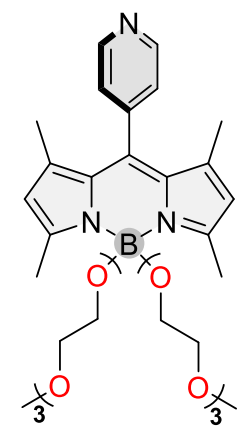<br>**9-TEG**	CYH	506	521	0.273	0.81, 3.03 (1.06)	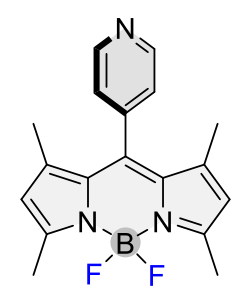<br>**9**	CYH	505	518	0.182	1.16 (1.32)
	TOL	507	522	0.341	1.24, 2.58 (1.05)		TOL	506	521	0.352	1.76 (1.2)
	DCM	506	522	0.243	1.37, 2.95 (1.08)		DCM	504	518	0.277	1.75 (1.19)
	EtOH	504	522	0.254	1.01, 3.3 (1.09)		EtOH	503	517	0.187	1.34, 3.98 (1.06)
	MeOH	504	519	0.220	0.82, 3.64 (1.15)		MeOH	502	517	0.163	1.08, 4.34 (1.01)
	MeCN	502	516	0.246	0.82, 2.94 (1.2)		MeCN	500	514	0.243	1.72 (1.17)
	DMF	504	520	0.329	0.91, 2.55 (1.05)		DMF	503	518	0.344	2.07 (1.02)
	DMSO	505	521	0.436	2.6 (1.24)		DMSO	504	518	0.445	2.57 (1.16)
	Formamide	505	522	0.490	3.19 (1.09)						
	H_2O	501	518	0.275	0.33, 2.71 (1.03)						

[a] CYH – cyclohexane, TOL – toluene, DCM – dichloromethane, EtOH – ethanol, MeOH – methanol, MeCN – acetonitrile, DMF – dimethylformamide, DMSO – dimethylsulfoxide. [b] Samples were excited at the vibrational shoulder of the BODIPY absorption at 470, 480 or 520 nm. [c] Fluorescence quantum yields were measured using Rhodamine 6G as a standard (Φ_F = 0.95 in EtOH).[23]

Compound	Ethanol	Water	Excitation spectra

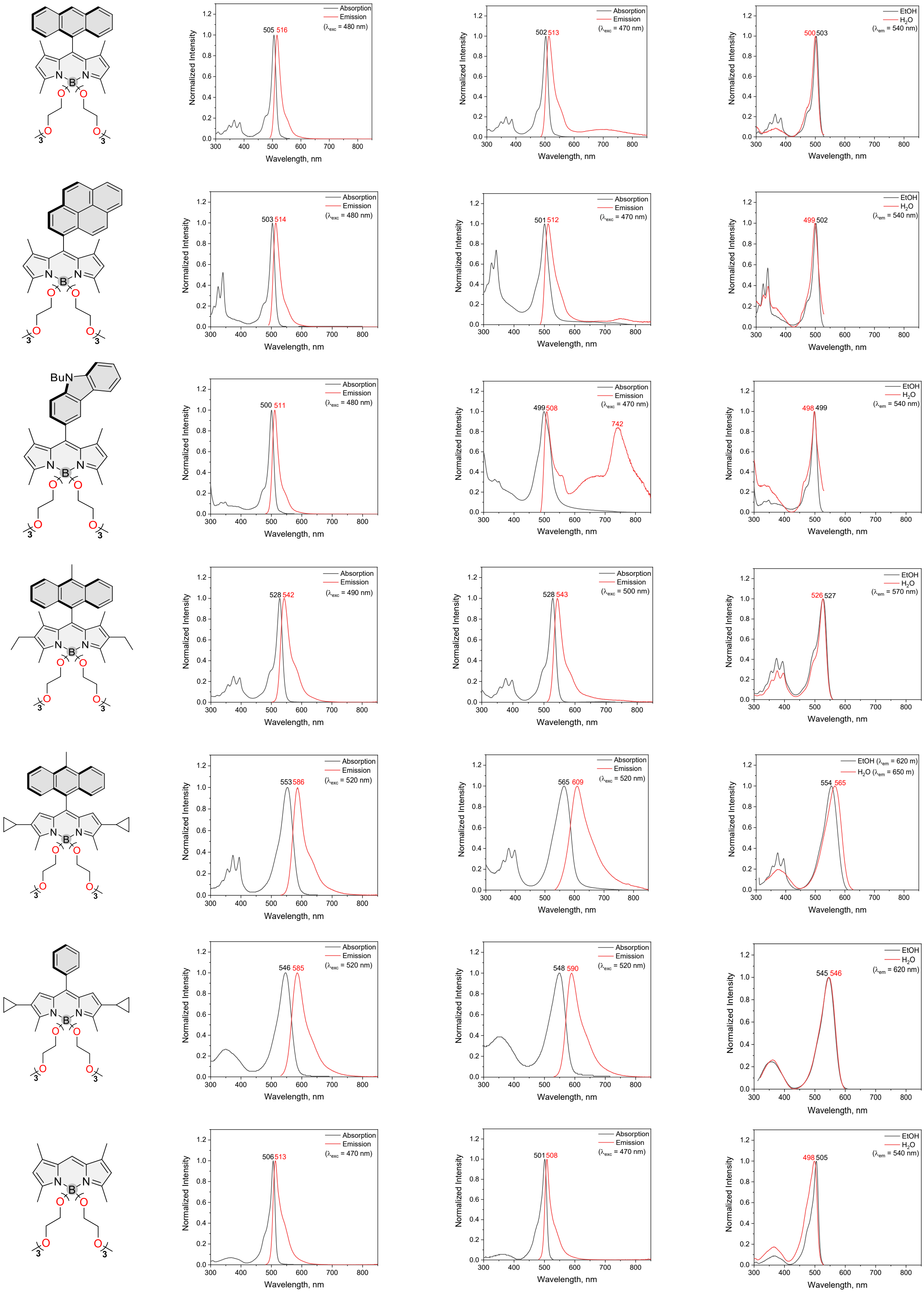

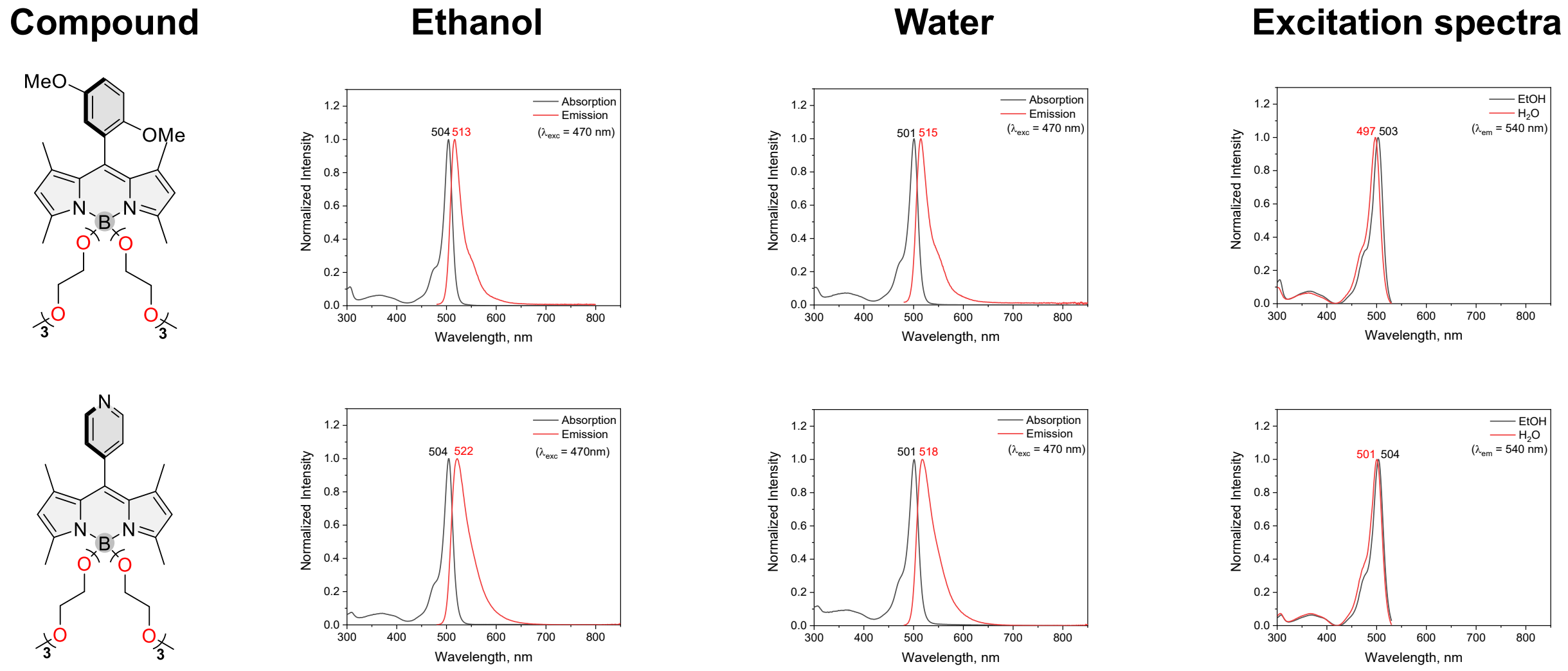

Figure S1. Absorption, emission and excitation spectra of **BODIPY-TEG**s in ethanol and water.

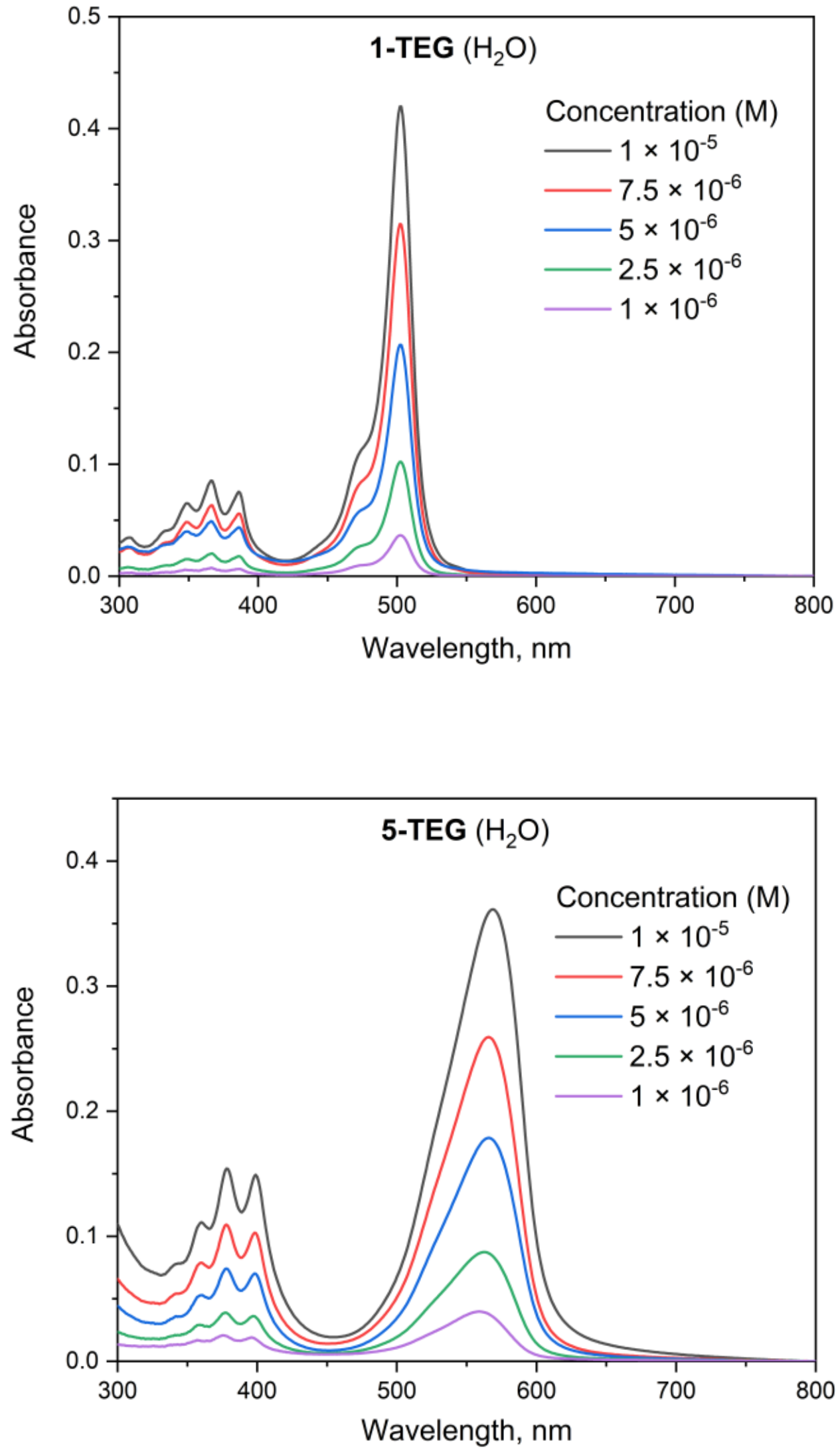

Figure S2. Concentration-dependent spectra of compounds **1-TEG** and **5-TEG** in water.

Table S4. Fluorescence quantum yields of **5-TEG** in EtOH–water mixtures.

EtOH:H_2O (v/v)	Φ_F	EtOH:H_2O (v/v)	Φ_F
100:0	0.539	50:50	0.235
90:10	0.442	40:60	0.166
80:20	0.396	30:70	0.122
70:30	0.345	20:80	0.0658
60:40	0.290	10:90	0.0479

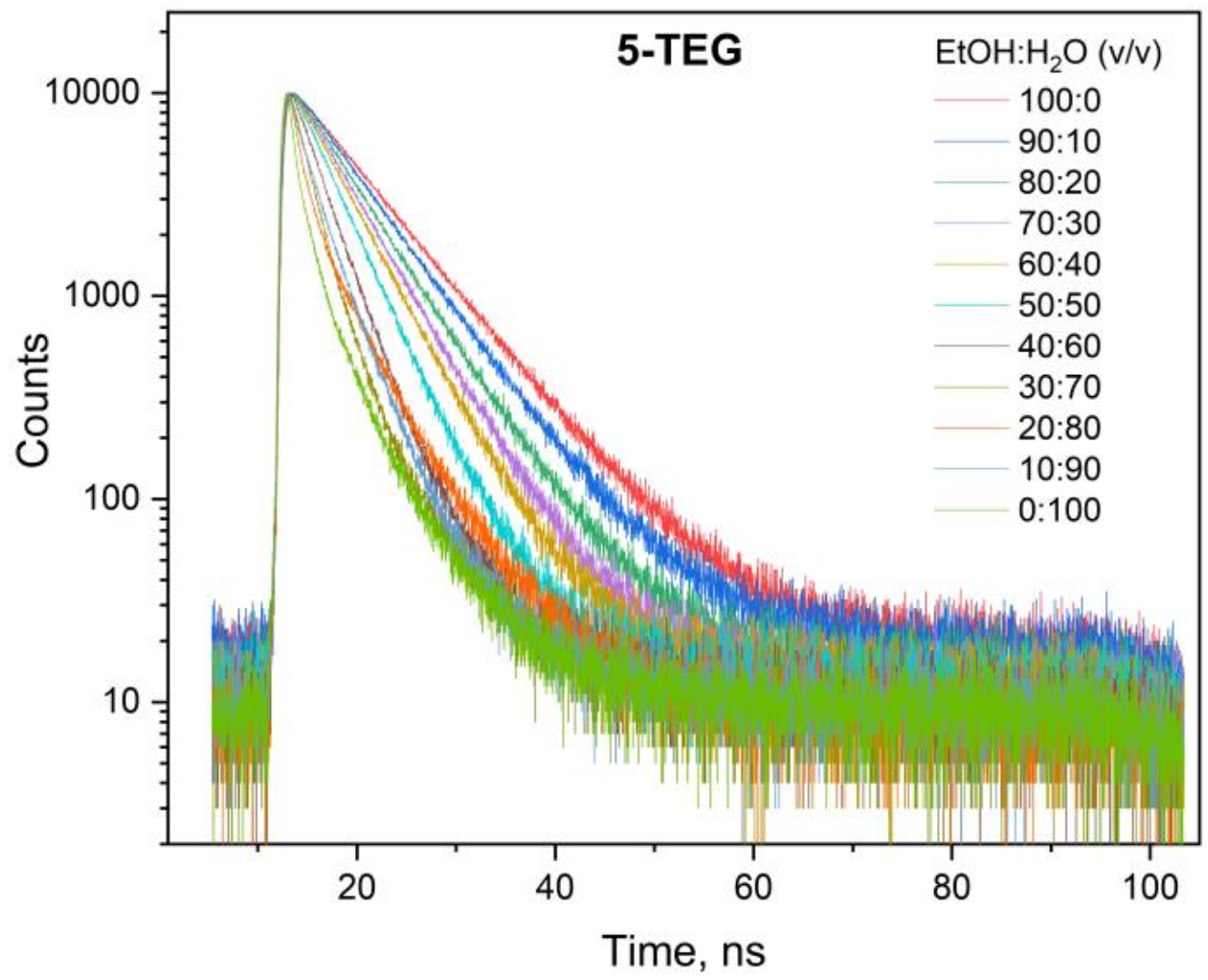

Figure S3. Time-resolved fluorescence decay traces of **5-TEG** recorded in ethanol/water mixtures of varying composition (v/v, as indicated in the legend).

Table S5. Fluorescence lifetimes of **5-TEG** in EtOH/H_2O mixtures (v/v).

EtOH:H_2O (v/v)	Fit model	τ_1 (A1%)	τ_2 (A2%)	τ_3 (A3%)	$\langle\tau\rangle$amp (ns)	χ^2
100:0	mono	6.96 (100)	–	–	6.96	1.30
90:10	mono	6.19 (100)	–	–	6.19	1.22
80:20	mono	5.42 (100)	–	–	5.42	1.14
70:30	mono	4.88 (100)	–	–	4.88	1.16
60:40	mono	4.47 (100)	–	–	4.47	1.14
50:50	mono	3.76 (100)	–	–	3.76	1.26
40:60	bi	2.46 (82)	4.92 (18)	–	2.90	1.05
30:70	bi	1.67 (82)	5.19 (18)	–	2.30	1.11
20:80	bi	0.91 (48)	4.65 (52)	–	2.85	1.23
10:90	bi	1.35 (38)	3.39 (62)	–	2.61	1.27
0:100	tri	0.13 (21)	1.9 (25)	5.49 (54)	3.47	0.99

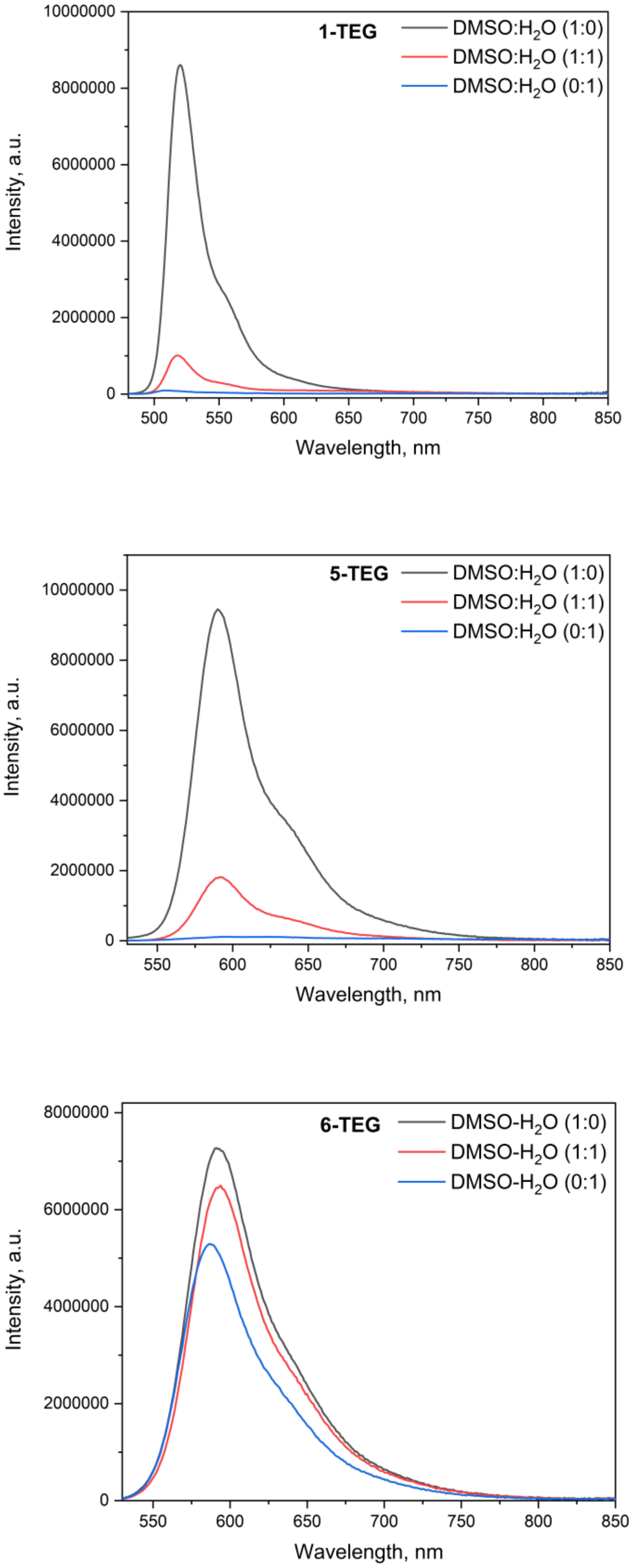

Figure S4. Selected emission spectra of the **BDP-TEG**s in DMSO/H_2O mixtures of varying composition (v/v, as indicated in the legend). Fluorescence was recorded with excitation at 470 nm (**1-TEG**) or 520 nm (**5-TEG**).

Table S6. Fluorescence quantum yields in DMSO/H_2O mixtures of selected **BDP-TEG**s.

DMSO/H_2O ratio	Φ_F		
	**1-TEG**	**5-TEG**	**6-TEG**
1:0	0.380	0.590	0.546
5:1	0.233	0.534	0.495
2:1	0.111	0.348	0.468
1:1	0.043	0.182	0.446
1:2	0.019	0.054	0.429
1:5	0.007	0.025	0.386
0:1	0.005	0.021	0.367

Table S7. Fluorescence lifetimes of **1-TEG** in DMSO/H_2O mixtures (v/v).

DMSO:H_2O (v/v)	Fit model	τ_1 (A1%)	τ_2 (A2%)	τ_3 (A3%)	$\langle\tau\rangle$amp (ns)	χ^2
100:0	mono	4.48 (100)	–	–	4.48	1.12
90:10	mono	4.37 (100)	–	–	4.37	1.09
80:20	mono	4.22 (100)	–	–	4.22	1.15
70:30	bi	2.24 (10)	4.08 (90)	–	3.90	1.19
60:40	bi	0.19 (7)	3.74 (93)	–	3.49	1.31
50:50	tri	0.08 (10)	2.87 (51)	5.55 (39)	3.64	1.08
40:60	tri	0.09 (12)	2.62 (46)	5.99 (42)	3.74	1.13
30:70	tri	0.12 (12)	2.18 (29)	5.98 (59)	4.17	1.05
20:80	tri	0.15 (10)	1.93 (22)	5.99 (67)	4.45	1.04
10:90	tri	0.26 (11)	1.78 (24)	5.88 (65)	4.28	1.03
0:100	tri	0.2 (30)	1.33 (15)	6.55 (55)	3.86	1.11

Table S8. Fluorescence lifetimes of 5-**TEG** in DMSO/H_2O mixtures (v/v).

DMSO:H_2O (v/v)	Fit model	τ_1 (A1%)	τ_2 (A2%)	τ_3 (A3%)	⟨τ⟩amp (ns)	χ^2
100:0	mono	5.85 (100)	–	–	5.84	1.12
90:10	mono	5.76 (100)	–	–	5.76	1.04
80:20	mono	5.51 (100)	–	–	5.51	1.16
70:30	mono	4.9 (100)	–	–	4.9	1.0
60:40	mono	3.99 (100)	–	–	3.99	1.15
50:50	bi	2.44 (81)	4.96 (19)	–	2.92	1.19
40:60	bi	1.44 (76)	5.42 (24)	–	2.39	1.21
30:70	tri	0.25 (22)	1.33 (38)	5.19 (40)	2.64	1.13
20:80	tri	0.19 (24)	1.96 (43)	5.3 (33)	2.58	1.11
10:90	tri	0.14 (28)	1.83 (29)	5.12 (43)	2.78	1.15
0:100	tri	0.13 (21)	1.9 (25)	5.49 (54)	3.47	0.99

Table S9. Fluorescence lifetimes of **6-TEG** in DMSO/H_2O mixtures (v/v).

DMSO:H_2O (v/v)	Fit model	τ_1 (A1%)	τ_2 (A2%)	τ_3 (A3%)	⟨τ⟩amp (ns)	χ^2
100:0	mono	6.08 (100)	–	–	6.08	1.16
90:10	mono	6.17 (100)	–	–	6.17	1.05
80:20	mono	6.2 (100)	–	–	6.2	1.12
70:30	mono	6.32 (100)	–	–	6.32	1.13
60:40	mono	6.35 (100)	–	–	6.35	1.15
50:50	mono	6.3 (100)	–	–	6.3	1.03
40:60	mono	6.33 (100)	–	–	6.33	1.2
30:70	mono	6.29 (100)	–	–	6.29	1.18
20:80	mono	6.19 (100)	–	–	6.19	1.29
10:90	mono	6.07 (100)	–	–	6.07	1.36
0:100	mono	5.78 (100)	–	–	5.78	1.37

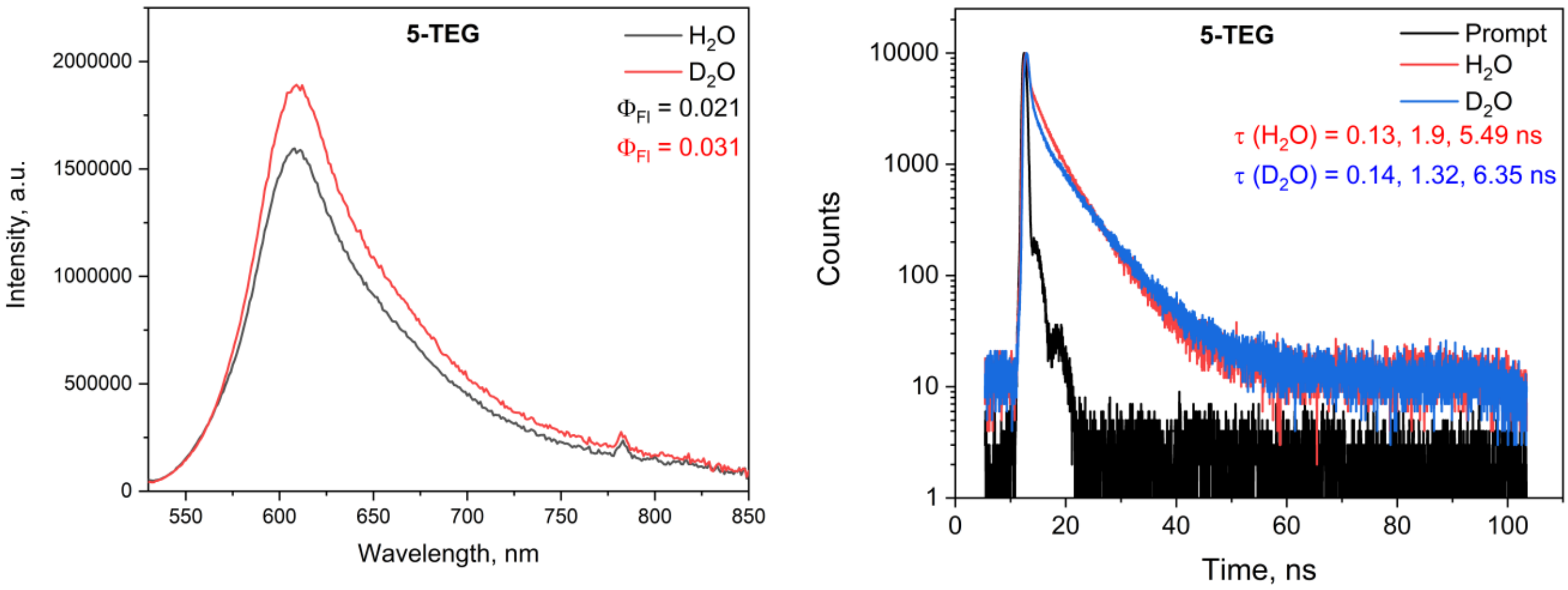

Figure S5. Fluorescence spectra and time-resolved fluorescence decay traces of **5-TEG** recorded in H_2O and D_2O. Fluorescence was recorded with excitation at 520 nm.

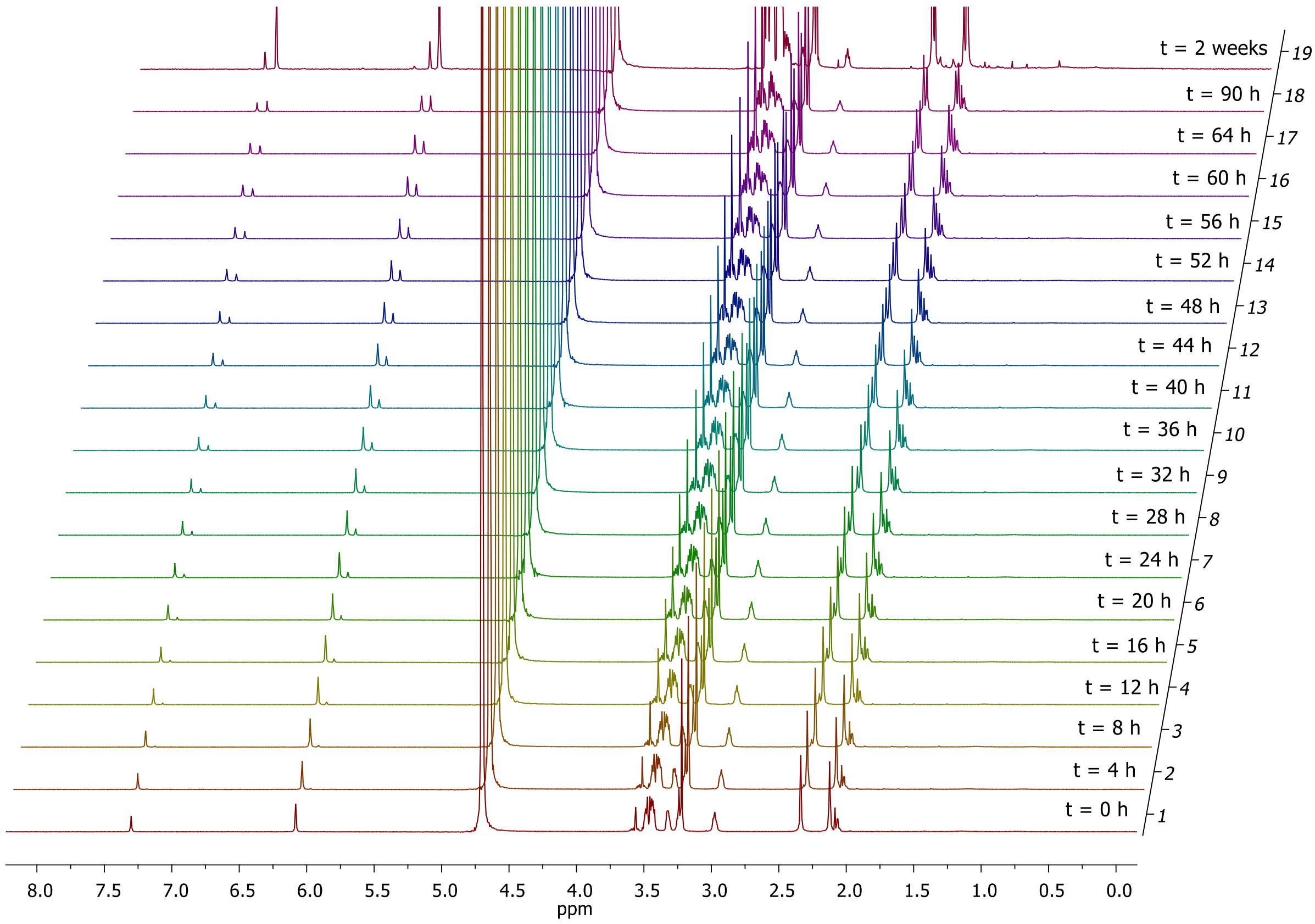

Figure S6. Temporal evolution of the ^1H NMR spectra of **7-TEG** in D_2O (concentration: 10 mM).

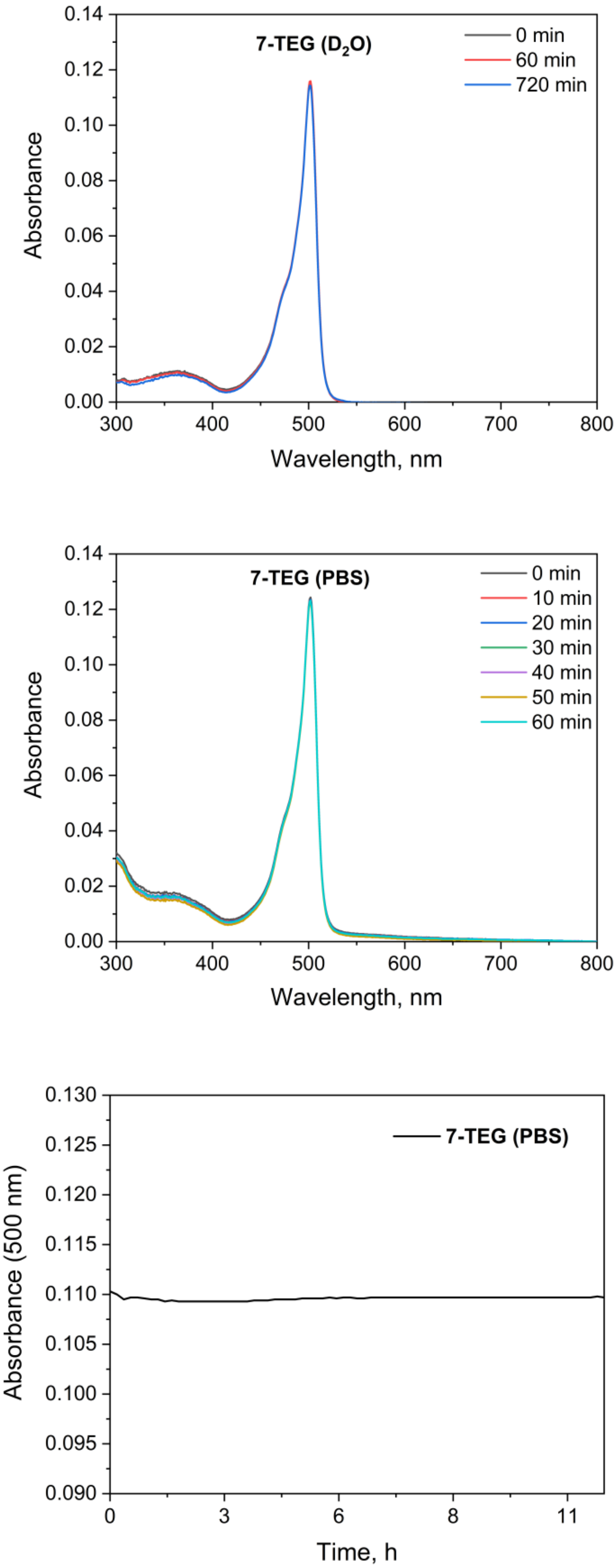

Figure S7. Stability of **7-TEG** in aqueous media, monitored by UV-Vis absorption spectra in D_2O and PBS over time, and by absorbance at 500 nm in PBS over 12 h.

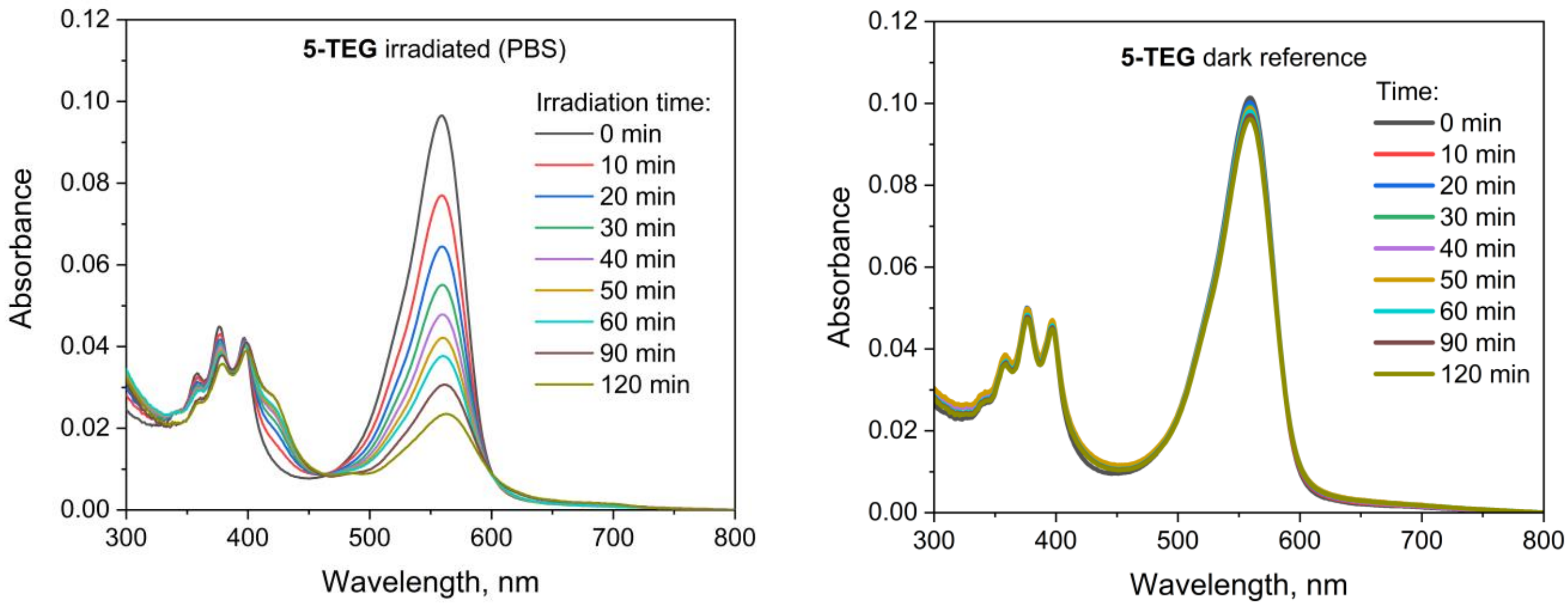

Figure S8. Time-dependent UV–Vis absorption spectra of **5-TEG** in an air-saturated PBS solutions under continuous 520 nm irradiation (14 mW cm^{-2}) (left) and kept in the dark (right).

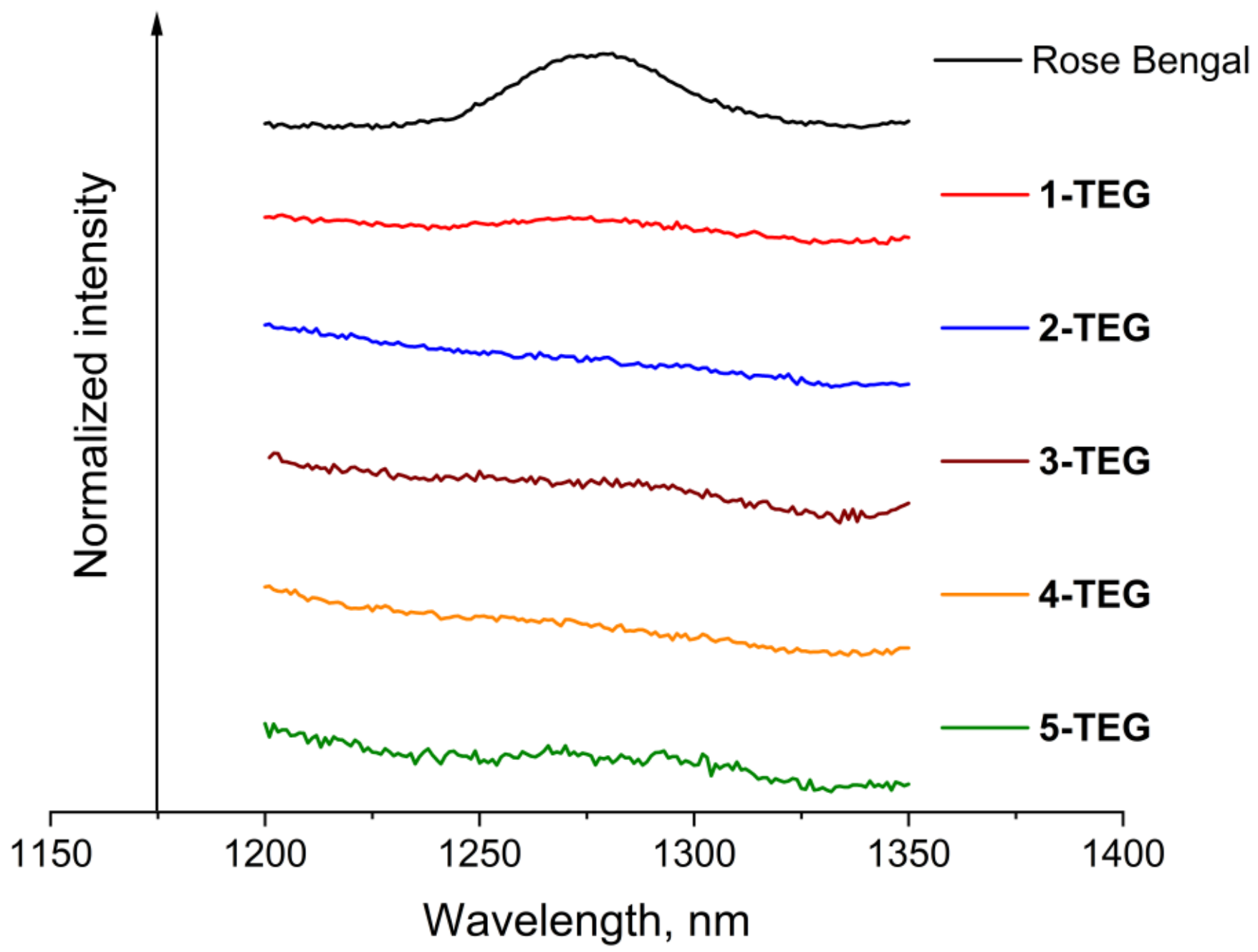

Figure S9. Singlet oxygen phosphorescence emission of **BDP-TEG**s in D_2O compared with Rose Bengal.

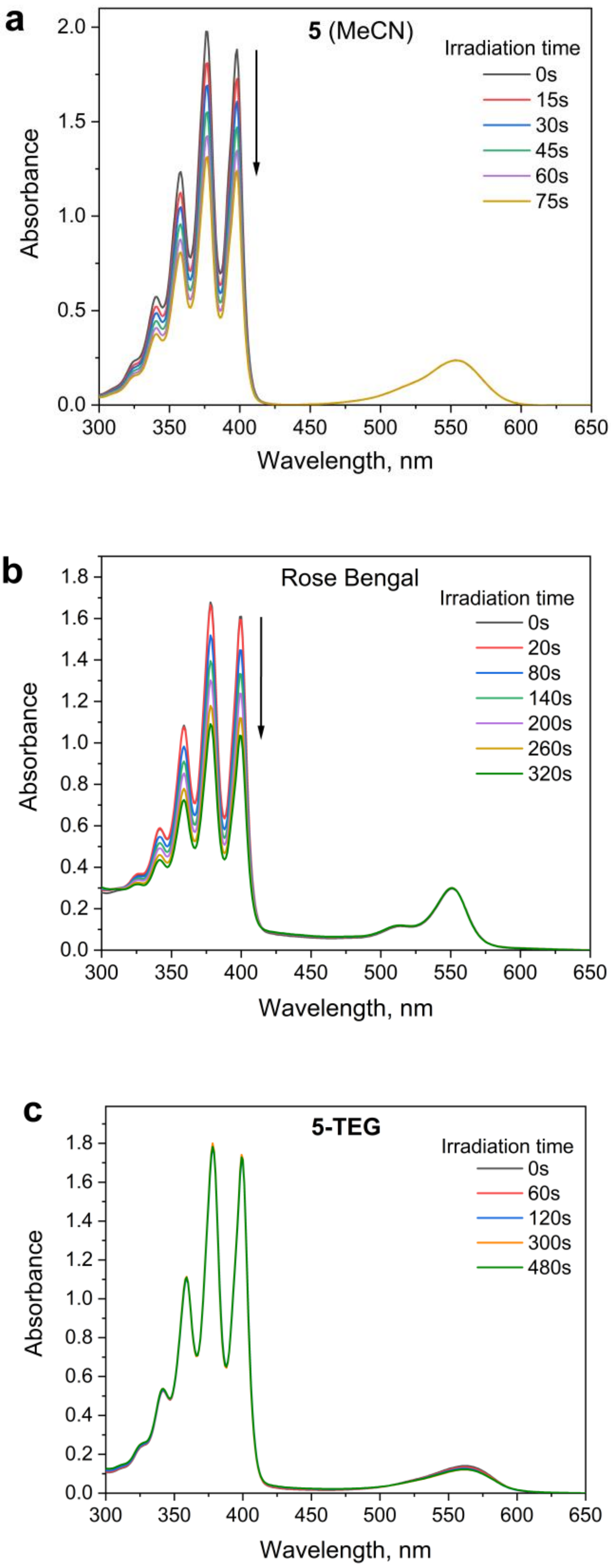

Figure S10. Photooxidation of 9,10-dimethylanthracene in air-saturated MeCN in the presence of BODIPY **5**. (b,c) Photooxidation of 1,9-dimethylanthracenedipropionate in air-saturated MeOH/H_2O (1:4) in the presence of Rose Bengal (b) and **5-TEG** (c). In all cases, irradiation was performed with a 520 nm laser at a constant power density of 14 mW cm^{-2}.

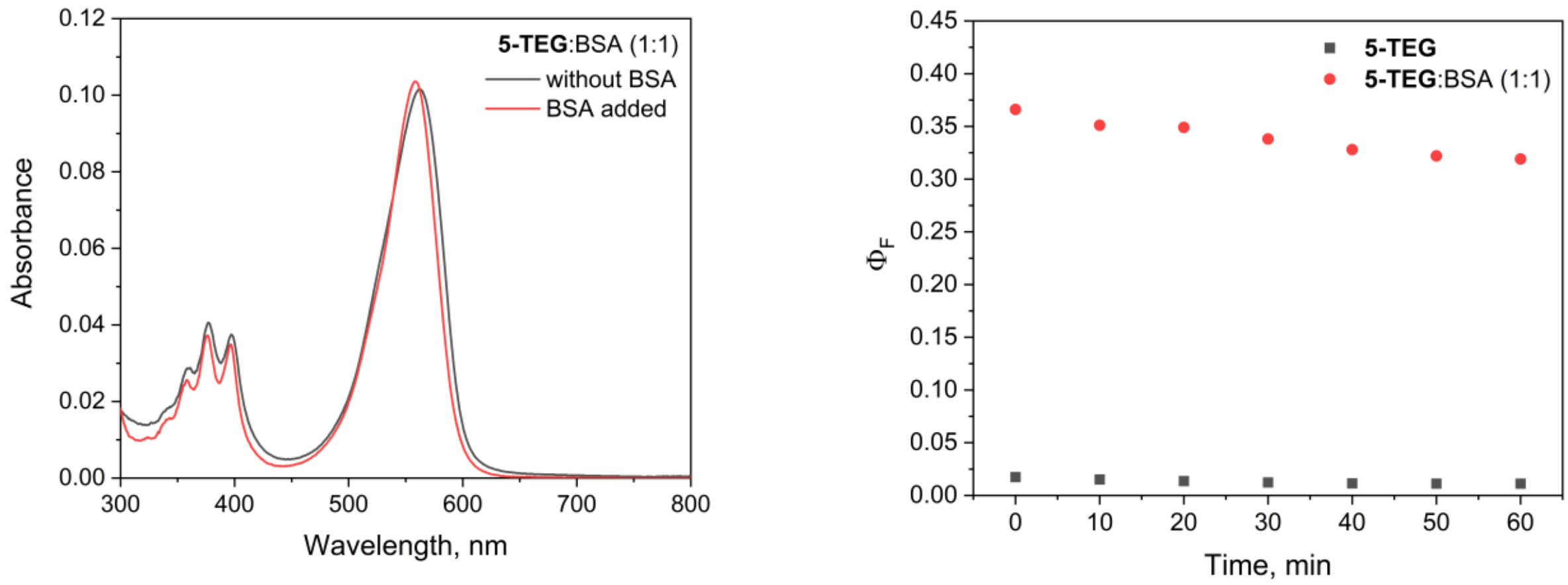

Figure S11. Change in absorption of **5-TEG** upon complexation with BSA (3 μM) (left). Changes in the fluorescence quantum yield of the solutions under observation for a period of 60 min. Fluorescence was recorded with excitation at 520 nm.

Table S10. Fluorescence lifetime decay and quantum yield of **5-TEG** on complexation with BSA.

5-TEG:BSA ratio	Fit model	τ_1 (A1%)	τ_2 (A2%)	τ_2 (A2%)	⟨τ⟩amp (ns)	χ^2	Φ_F
1:0	tri	0.13 (21)	1.9 (25)	5.49 (54)	3.47	1.03	0.021
1:0.1	bi	3.74 (33)	8.03 (67)	–	6.61	1.04	0.092
1:0.5	bi	4.58 (22)	8.44 (78)	–	7.58	1.15	0.255
1:1	bi	3.48 (15)	8.08 (85)	–	7.39	1.04	0.357
1:2	bi	3.94 (14)	8.24 (86)	–	7.64	1.15	0.398

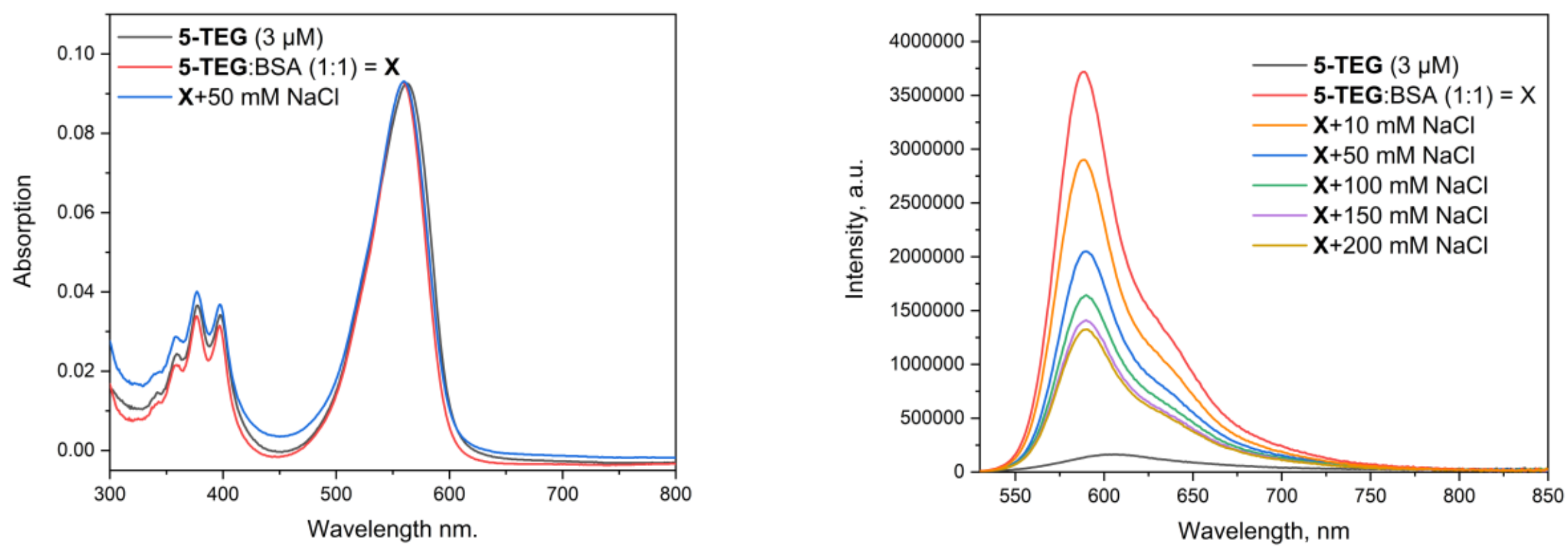

Figure S12. Absorption (left) and fluorescence emission (right) spectra of **5-TEG** (3 μM) in water and in the presence of BSA and NaCl. The effect of NaCl on the BSA-bound dye is shown for 10–200 mM NaCl. Fluorescence was recorded with excitation at 520 nm.

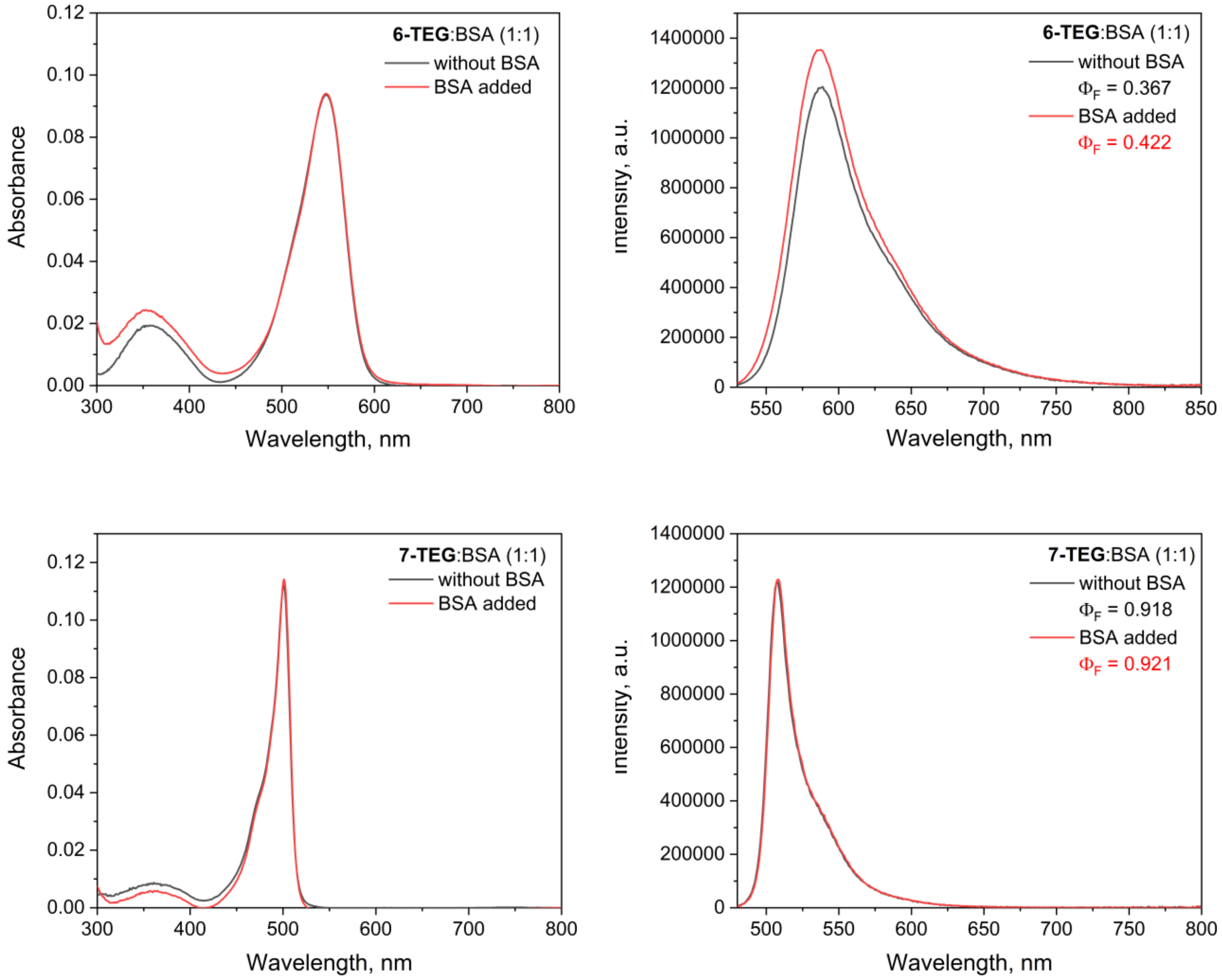

Figure S13. Changes in absorption and emission of reference compounds **6-TEG** and **7-TEG** upon complexation with BSA (3 μM). Fluorescence was recorded with excitation at 520 nm (**6-TEG**) or 470 nm (**7-TEG**).

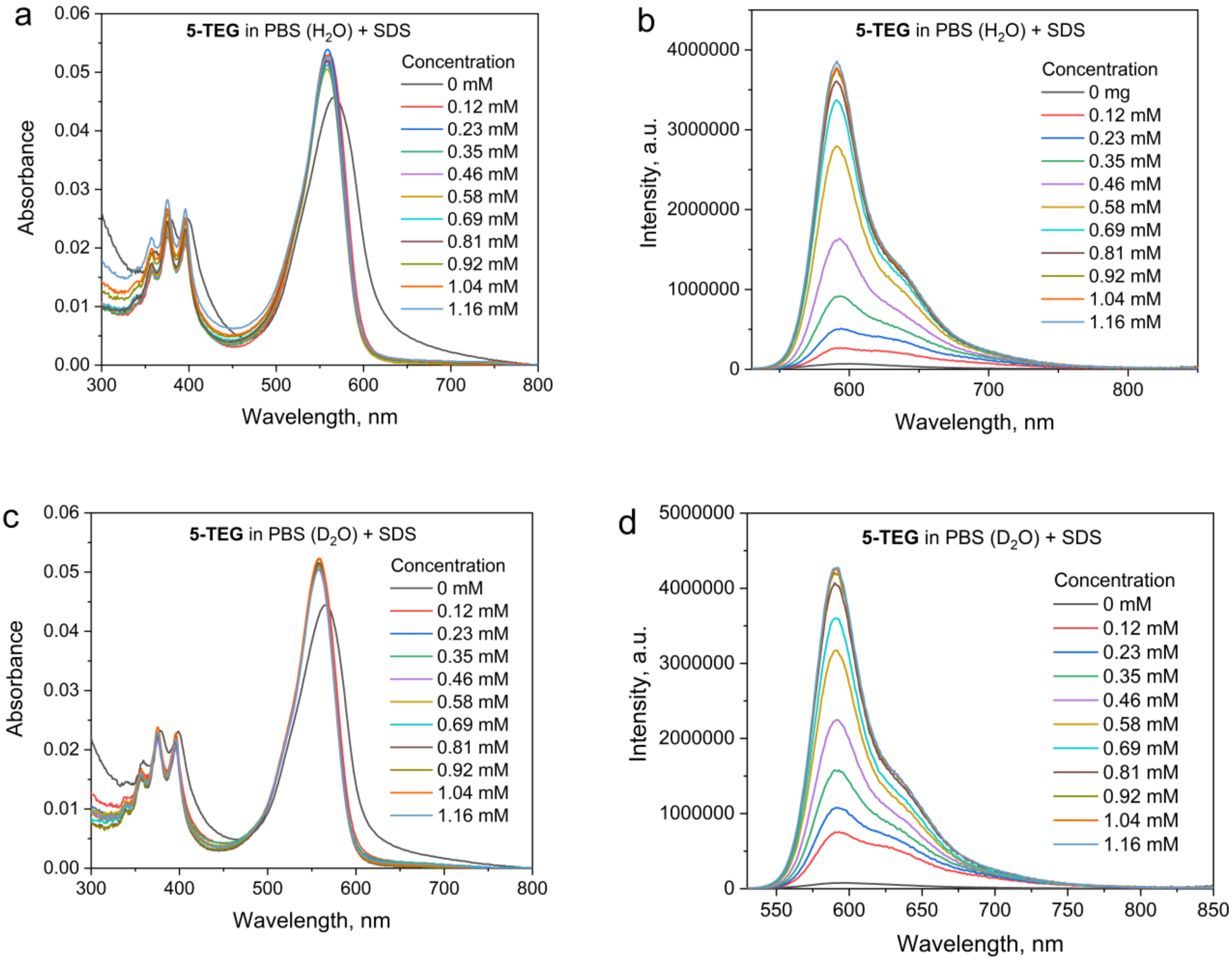

Figure S14. Effect of SDS on the photophysical properties of **5-TEG** (1.5 μM) in PBS prepared with H_2O and D_2O. Absorption (a,c) and fluorescence emission spectra (b,d) recorded upon increasing SDS concentration in H_2O (a,b) and D_2O (c,d). Fluorescence was recorded with excitation at 520 nm.

Table S11. Time-resolved fluorescence parameters of **5-TEG** in PBS (H_2O) at different SDS concentrations.

SDS (mM)	Fit model	τ_1 (A1%)	τ_2 (A2%)	τ_3 (A3%)	⟨τ⟩amp (ns)	χ^2
0	tri	0.14 (18)	2.54 (29)	5.58 (53)	3.72	1.08
0.12	tri	0.28 (31)	1.34 (59)	4.94 (10)	1.37	1.09
0.23	tri	0.24 (17)	1.7 (62)	3.9 (21)	1.91	1.09
0.35	bi	2.11 (27)	4.8 (73)	–	4.07	1.17
0.46	bi	2.09 (12)	5.99 (88)	–	5.52	1.15
0.58	bi	3.44 (19)	6.68 (81)	–	6.07	1.2
0.69	mono	6.64 (100)	–	–	6.64	1.28
0.81	mono	7.08 (100)	–	–	7.08	1.19
0.92	mono	7.16 (100)	–	–	7.16	1.13
1.04	mono	7.20 (100)	–	–	7.20	1.09
1.16	mono	7.20 (100)	–	–	7.20	1.07

Table S12. Time-resolved fluorescence parameters of **5-TEG** in PBS (D_2O) at different SDS concentrations.

SDS (mM)	Fit model	τ_1 (A1%)	τ_2 (A2%)	τ_3 (A3%)	⟨τ⟩amp (ns)	χ^2
0	tri	0.19 (21)	2.0 (21)	6.27 (58)	4.10	1.08
0.12	tri	0.21 (28)	1.43 (56)	5.65 (16)	1.76	1.04
0.23	tri	0.3 (10)	2.17 (58)	4.45 (32)	2.71	1.11
0.35	bi	2.21 (30)	4.78 (70)	–	4.0	1.22
0.46	bi	2.54 (23)	5.73 (77)	–	5.0	1.12
0.58	bi	3.59 (20)	6.84 (80)	–	6.19	1.22
0.69	mono	6.84 (100)	–	–	6.84	1.36
0.81	mono	7.29 (100)	–	–	7.29	1.17
0.92	mono	7.58 (100)	–	–	7.58	1.1
1.04	mono	7.64 (100)	–	–	7.64	1.17
1.16	mono	7.66 (100)	–	–	7.66	1.11

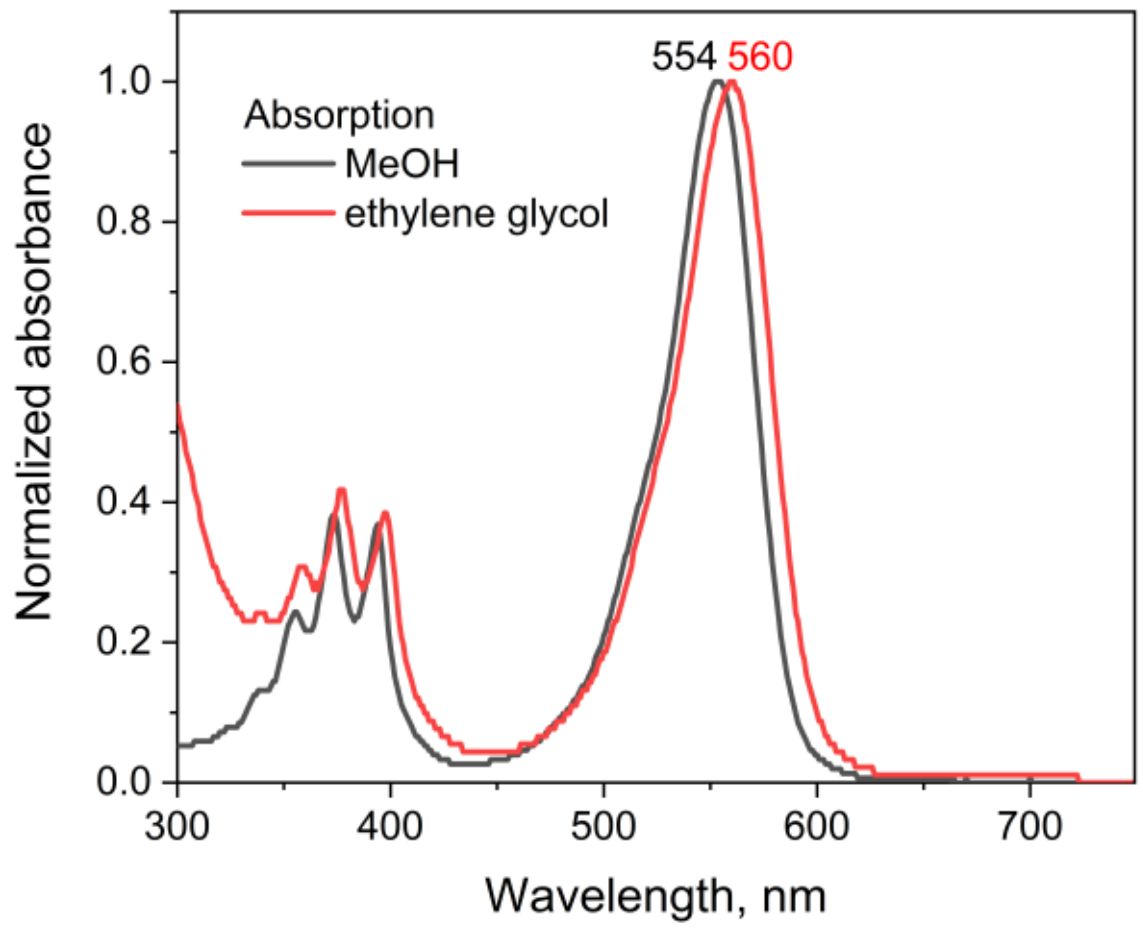

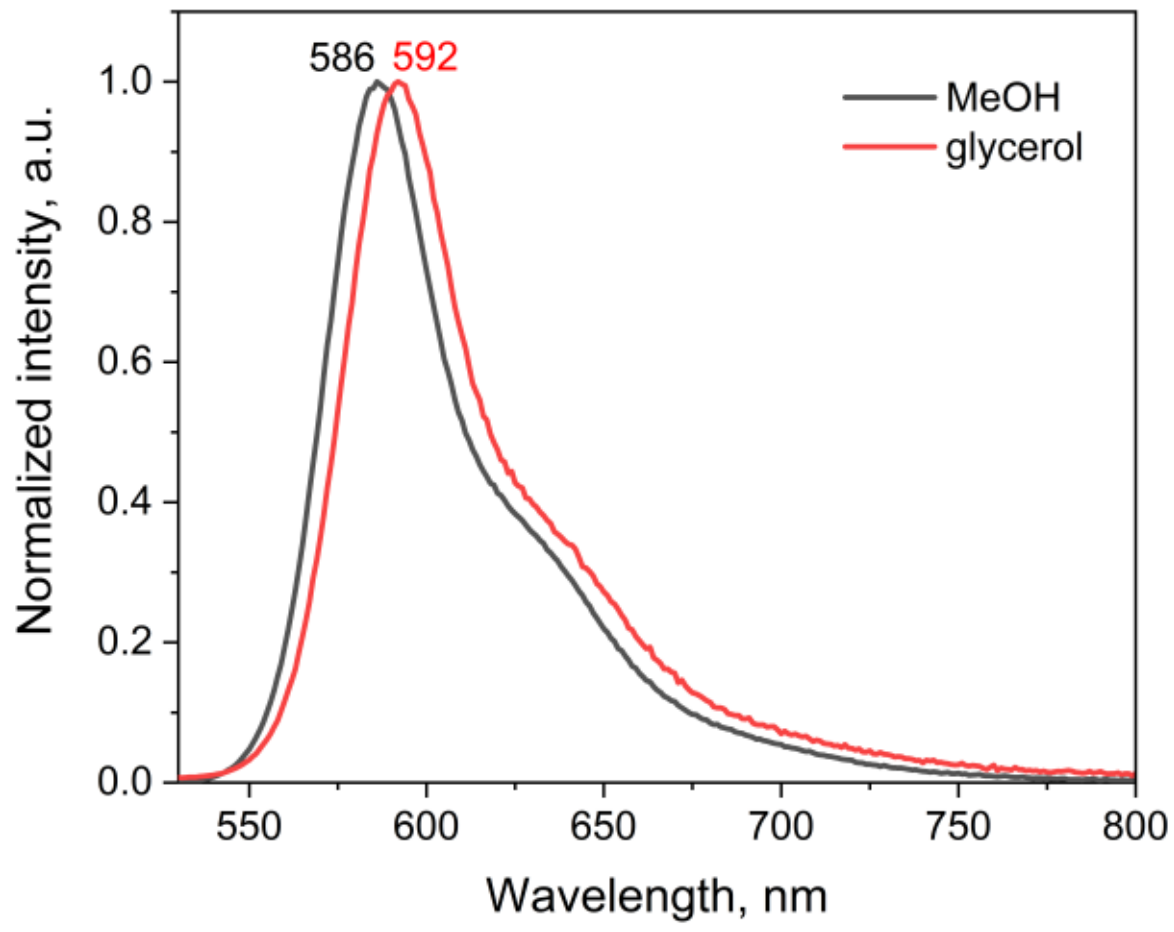

Figure S15. Normalized absorption (left) and fluorescence (right) spectra of **5-TEG** in methanol and glycerol. Fluorescence was recorded with excitation at 520 nm.

Table S13. Fluorescence lifetimes of **5-TEG** in methanol/glycerol mixtures (v/v).

MeOH:glycerol (v/v)	Fit model	τ_1 (A1%)	τ_2 (A2%)	χ^2
100:0	mono	6.64 (100)	—	1.16
90:10	mono	6.14 (100)	—	1.14
80:20	mono	5.99 (100)	—	1.19
70:30	mono	5.68 (100)	—	1.13
60:40	mono	5.72 (100)	—	1.11
50:50	mono	5.56 (100)	—	1.07
40:60	mono	5.48 (100)	—	1.69
30:70	mono	5.39 (100)	—	1.22
20:80	mono	5.21 (100)	—	0.99
10:90	mono	4.92 (100)	—	1.23
0:100	mono	4.88 (100)	—	1.38

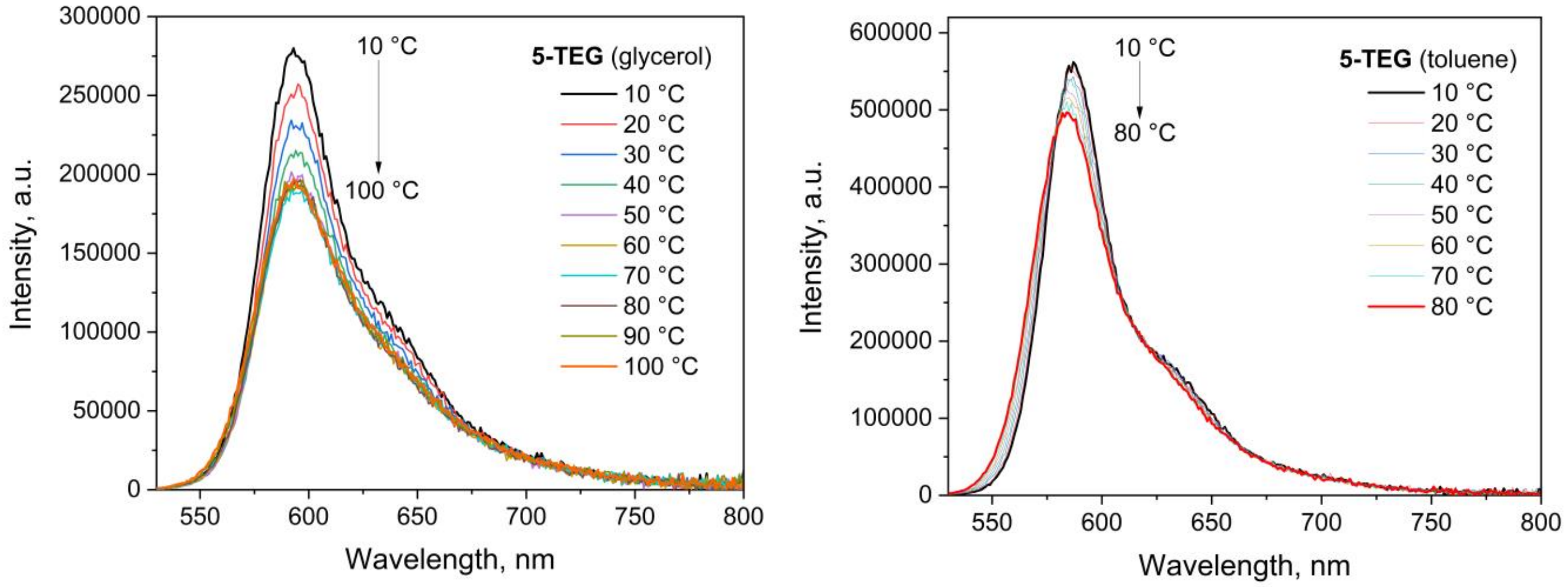

Figure S16. Variable-temperature fluorescence emission spectra of **5-TEG** recorded in glycerol (left, 10–100 °C) and toluene (right, 10–80 °C). Fluorescence was recorded with excitation at 520 nm.

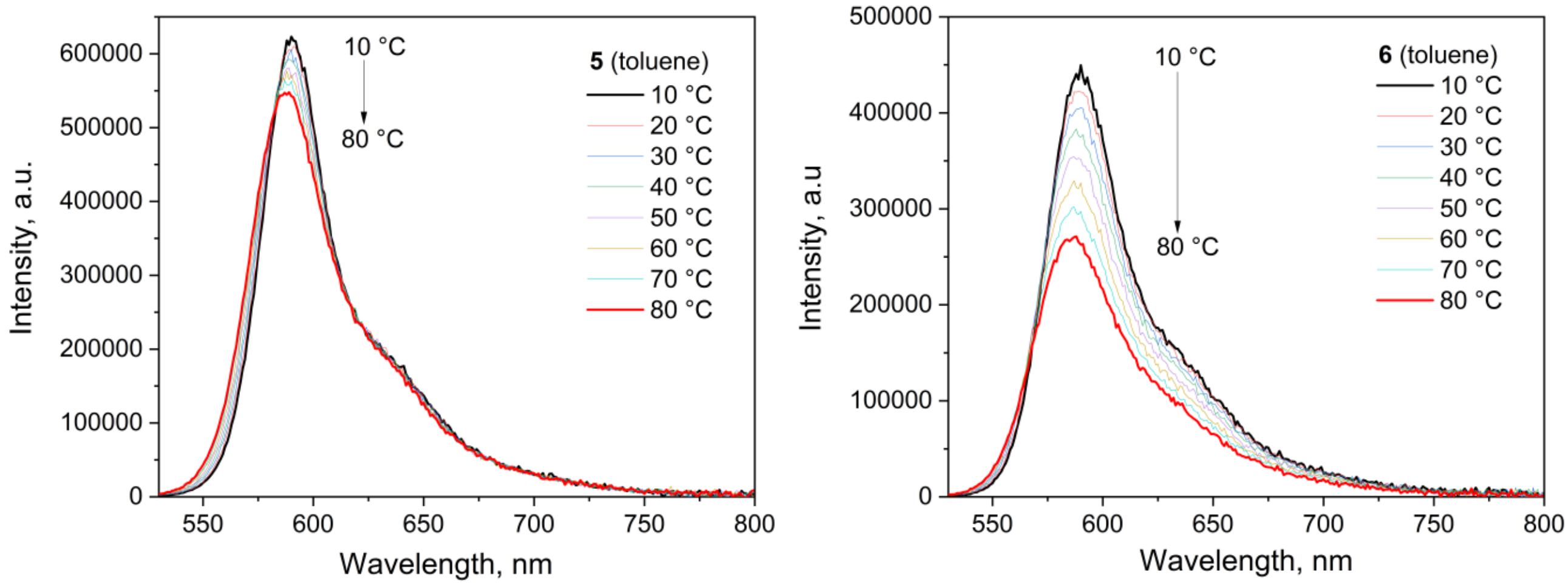

Figure S17. Variable-temperature fluorescence emission spectra of compounds **5** (left) and **6** (right) recorded in toluene over 10–80°C. Fluorescence was recorded with excitation at 520 nm.

4. Fluorescence Lifetime Imaging Microscopy

Small intestinal organoid culture materials

Cell and organoid culture materials

McCoy's 5A media (392-0420, VWR, Belgium), L-glutamine (25030081, Gibco, Belgium), HEPES (15630080, Thermo Fisher Scientific, Belgium), sodium pyruvate (11360039, Thermo Fisher Scientific, Belgium), FBS (A5256701, Gibco, Belgium), Trypsin-EDTA solution (T4049, Sigma-Aldrich, Belgium), 3D Petri Dish® micromolds (Z764019, Sigma-Aldrich, Belgium), IntestiCult™ Organoid Growth Medium (Mouse) (STEMCELL technologies, 06005, Belgium), 200 mM GlutaMax, Matrigel growth factor-reduced (734-0269, VWR, Belgium), mouse intestinal organoid growth medium (STEMCELL technologies, 06005, Belgium), DMEM high glucose GlutaMax^{TM} Supplement media (61965026, Gibco, Belgium), 24-well Tissue Culture plates (734-2325, VWR, Belgium).

Cell culture

Human colon cancer HCT116 cell line (ATCC) was cultured using McCoy's 5A culture media supplemented with 10% heat-inactivated FBS, 1 mM sodium pyruvate, 2 mM L-glutamine in a humidified atmosphere at 37 °C, ambient O_2, and 5% CO_2. At 90% confluency, cells were detached with 0.25% trypsin EDTA solution (1 mM) and passaged. Cells were cultured in antibiotic-free conditions and regularly tested for mycoplasma and bacterial contamination.

For microscopy of adherent (2D) cell culture 10,000 of HCT116 cells were seeded in the microwells of 12-well silicon-multiwell microscopy dishes (Ibidi GmbH, 81201) with the bottom cover glass (no. 1.5 thickness) coated with 0.07 mg/mL collagen IV/0.015 mg/mL d-poly lysine. Cells were grown for 2 days in a culture media and stained for 1 h with 9, 4.5, 2.25 and 0.5 µM **5-TEG** prior imaging analysis. After rinsing 3 times with imaging media (phenol-free and sodium bicarbonate-free DMEM media supplemented with 10 mM D-glucose, 2 mM GlutaMax, 10 mM HEPES, 1 mM sodium pyruvate; referred as imaging media) cells were left in imaging media for a live imaging analysis.

HCT116 spheroids (initial 500 cells per spheroid) were formed using a high-throughput method, according to a previously published protocol.[24] Briefly, 2% agarose molds were made using the 3D Petri Dish® micromolds hereby producing 81 spheroids per mold. After 3 days of formation, spheroids were transferred to a 0.07 mg/mL collagen IV/0.015 mg poly-D-lysine coated microwells of 12-well silicon microscopy dishes (ibidi), stained for 1 h with 4.5 µM **5-TEG** in imaging media, washed 5 times with imaging media and left in imaging media for the live imaging analysis.

Organoid culture

Production of C57BL/6N mouse (2 month old) small intestinal organoid culture was done using the method described method [25] Lgr5-GFP (*Lgr5-EGFP-ires-CreERT2*) mouse intestinal organoid culture[26] were generously provided by Prof. H. Clevers Lab (Hubrecht Institute, The Netherlands). Then mouse small intestinal organoids were cultured at density 150–300 organoids per 50 µL Matrigel dome in 500 µL of IntestiCult™ Organoid Growth Medium (Mouse) per well of 24-well plate. The culture were regularly passaged (every 3-4 days) by mechanical disruption of Matrigel domes, as described previously.[27] Organoids were cultured in antibiotic-free conditions and regularly checked for mycoplasma and bacterial contamination (CTC2 Core, UGent).

For microscopy, organoids were embedded in 2 µl Matrigel domes inside the µ-Slide 18 well glass bottom ibidi microscopy dishes (81817, ibidi) at a density of 3 - 10 organoids per dome and grown for one day in organoid culture media prior the analysis. Organoids were pre-stained for 1 h with 4.5 µM

of 4-TEG in culture media, washed once with imaging media and left in imaging media for live microscopy.

Microscopy

Confocal FLIM microscopy was performed on a Stellaris 8 Falcon/Dive microscope, (Leica Microsystems, Ghent Light Microscopy Core, Ghent University), equipped with a white-light laser (440 − 790 nm), multiphoton MaiTai HP laser (690 – 1040 nm; Spectra-Physics), HC PL Apo 10×/0.4 air, HC Fluotar 25×/0.95 W, HC PL Apo 40×/1.25 GLYC corr, HC PL Apo 63×/1.4 oil objectives, HyD X, HyD R and HyD S internal detectors and HyD NDD and PMT NDD external detectors for two-photon imaging, temperature/CO_2-controlled incubator, and dedicated LAS X acquisition and analysis software (ver. 4.8.2). For routine microscopy, a 40×/1.25 GLYC corr. objective was used, with typical settings: 80 or 40 MHz white-light laser frequency pulse, scan speed 200 - 600 Hz, pixel dwell time 0.36 – 4.1 µs, pinhole 1.0 airy units, 1 – 3 frame repetition rate, 512 × 512, 1024 × 1024 or 1128 × 1128 pixels resolution. The following general excitation and emission settings were used for fluorescence imaging: **5-TEG** excited at 555 nm (laser power 0.1 – 1.2 %, emission 578 – 615 or 565 - 605 nm), NAD(P)H excited at 740 nm (laser power 5%, emission 410-470 nm). 3D reconstruction of HCT116 spheroids was done with 1 µm step size and 114 frames (512 × 512 resolution); 3D reconstruction of HCT116 cell monolayer stained with 9 µM **5-TEG** for 1 h was done with 0.36 µm step size and 14 frames (1128 × 1128 resolution).

ATP analysis

Cytotoxicity effects of **5-TEG** on HCT116 cells were investigated using the CellTiter cell ATP assay (Promega, G7571) as described previously.[28] Briefly, 15,000 HCT116 cells per well were seeded on 96-well plates pre-coated with 0.07 mg/mL collagen IV. After one day in culture cells were incubated with 9 or 0.5 µM **5-TEG** (diluted in McCoy's 5A cell culture media) for 18 h and 1 h, followed by washing with imaging media. CellTiter-Glo reagent was added to each well with imaging media in a 1:1 proportion by volume, followed by 2 minutes on an orbital shaker to induce cell lysis. After stabilizing the luminescent signal for 10 min, the signal was read using the multimode microplate reader Varioscan LUX (Thermo Scientific) with an integration time of 1 s per well. Luminescence intensity signal was normalized by total protein concentration. Protein cell extracts were treated in the same manner on the same dish and prepared as described previously.[28]

Data analysis

Phasor plot analysis of cell monolayers stained with 0.5 – 9 µM **5-TEG**, as well as spheroids and organoids was done in an open source GS-Lab software[29] with applied median filter 10, harmonic 1, relative intensity threshold 0.3–1, tau phase (τ_φ) color-coded projection. 3D reconstruction of spheroids was done in ImageJ Fiji from τ_φ-color coded intensity image series produced in GS-Lab. 3D reconstruction of cell monolayers stained with 9 µM **5-TEG** was done from a seria of intensity images in LAS_X software (ver. 4.8.2). Fluorescence lifetime Phasor-based fraction unmixing analysis and τ_φ-color coded images for high resolution HCT116 monolayer stained with 9 µM **5-TEG** was done in LAS_X software (ver. 4.8.2). Unmixing was done with a 3 fixed τ_φ components equal to 4.0, 2.6 and 1.9 ns, radius 12. Fluorescence lifetime global fitting-based analysis of fluorescence decays was done in LAS_X software (ver. 4.8.2). For fitting-based analysis of probes fluorescence lifetime the n-Exponential reconvolution fitting model was applied with n=2, fitting range 1.294 – 12.609 ns. The quality of fitting decays was estimated based on χ2 of a global decay fitting and the residuals fluctuation ±10 photons along the fitted decay.

Table S14. Two-exponential global fitting analysis of **5-TEG** fluorescence lifetime in cell and organoid model. Lifetime values of organoid culture correspond to individual organoids on Fig. 7d (in order of left to right from top to bottom).

Sample type	[5-TEG], μM	A1, %	A2, %	τ_1, ns	τ_2, ns	Mean τ (Intensity Weighted), ns	Mean τ_2 (Amplitude Weighted), ns	X^2
HCT116 2D culture, 80 MHz frequency of the laser pulse	9	54.06	45.94	1.58	4.17	3.37	2.77	4.67
HCT116 2D culture, 40 MHz frequency of the laser pulse	9	39.97	60.03	0.79	3.12	2.78	2.19	2.44
HCT116 2D culture, 80 MHz frequency of the laser pulse	9	27.52	72.48	0.86	3.97	3.72	3.11	0.75
HCT116 2D culture, 80 MHz frequency of the laser pulse	4.5	17.24	82.76	1.32	5.60	5.37	4.86	0.73
HCT116 2D culture, 80 MHz frequency of the laser pulse	2.25	12.53	87.47	1.31	6.28	6.10	5.65	0.85
HCT116 2D culture, 80 MHz frequency of the laser pulse	0.5	10.06	89.94	1.04	6.22	6.10	5.70	1.13
3D Organoid cullture, 80 MHz	4.5	22.21	77.79	0.90	3.91	3.71	3.24	0.71
3D Organoid cullture, 80 MHz	4.5	22.64	77.36	0.99	4.16	3.94	3.44	0.76
3D Organoid cullture, 80 MHz	4.5	11.40	88.60	0.99	5.67	5.55	5.13	0.96
3D Organoid cullture, 80 MHz	4.5	16.46	83.54	1.12	5.21	5.02	4.54	0.75

Table S15. Two-exponential global fitting analysis of **5-TEG** fluorescence lifetime in cell monolayers after the storage of DMSO stock solution (2 months, -20 °C, dark).

Sample type	[5-TEG], µM	A1, %	A2, %	τ_1, ns	τ_2, ns	Mean τ (Intensity Weighted), ns	Mean τ_2 (Amplitude Weighted), ns	X^2
HCT116 2D culture, 80 MHz frequency of the laser pulse	9	34.87	65.13	0.8	3.32	3.02	2.44	2.53
HCT116 2D culture, 80 MHz frequency of the laser pulse	4.5	18.02	81.98	1.06	4.93	4.74	4.24	0.94
HCT116 2D culture, 80 MHz frequency of the laser pulse	2.25	15.35	84.65	1.14	5.52	5.34	4.85	0.74
HCT116 2D culture, 80 MHz frequency of the laser pulse	0.5	10.81	89.19	1.18	6.08	5.95	5.55	0.77

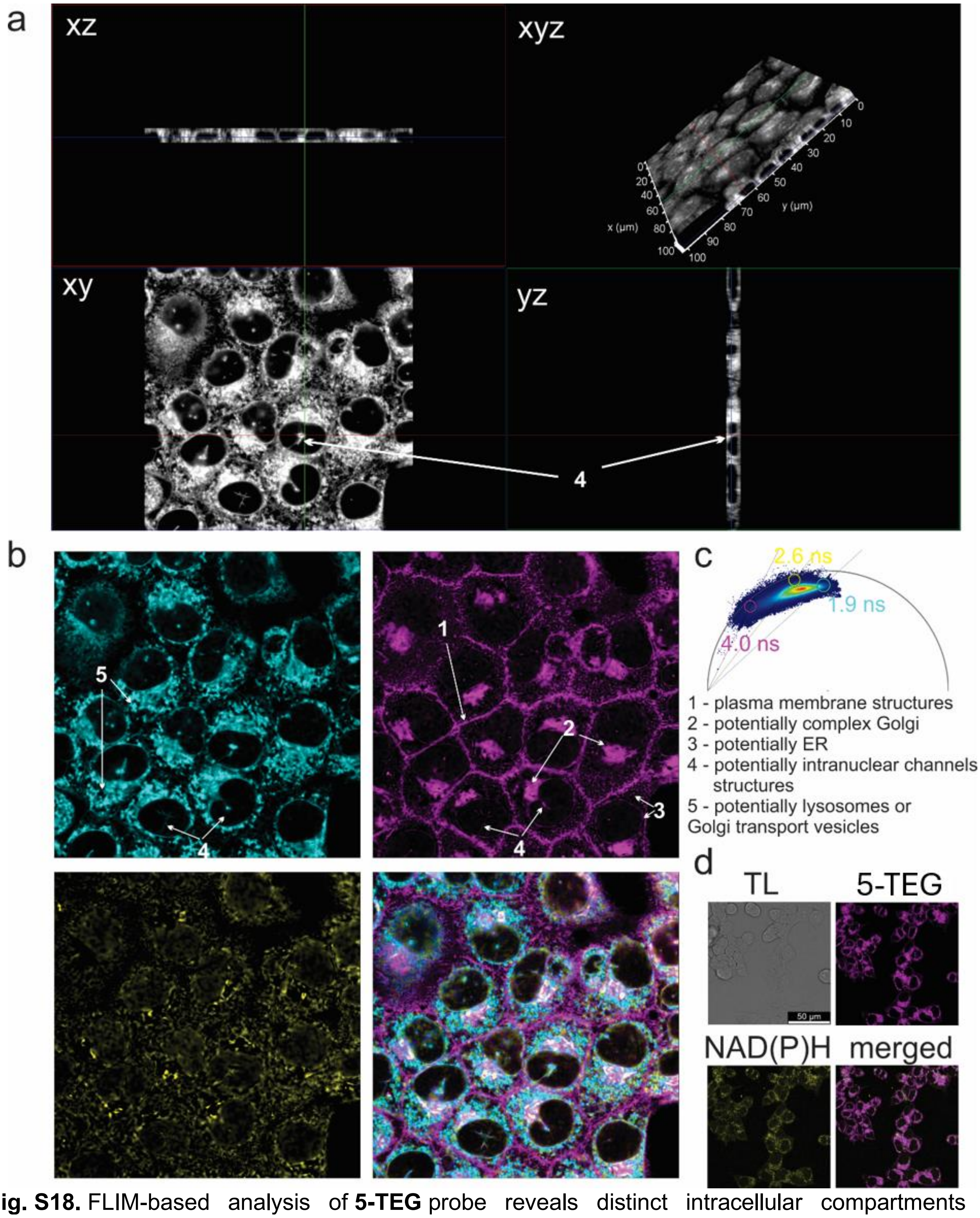

Fig. S18. FLIM-based analysis of **5-TEG** probe reveals distinct intracellular compartments in HCT116 cells. (a): Intensity-based 3D reconstruction of cell monolayer stained with 9 µM **5-TEG**. (b, c): Phasor-based lifetime fractions separation reveals different intracellular compartments labelled with **5-TEG** (b). Cyan, magenta and yellow color coding corresponds to individual lifetime fractions applied as shown on the respective phasor plot in (c). Numbers indicate different intracellular structures based on typical morphology of organelles. (d)**:** Imaging of 9 µM **5-TEG** staining combined with multiphoton imaging of NAD(P)H autofluorescence in HCT116 cells. Sections correspond to transmission light, **5-TEG** fluorescence intensity (magenta), NAD(P)H autofluorescence intensity (yellow) and merged fluorescence intensity images. Scale bar is 50 µm.

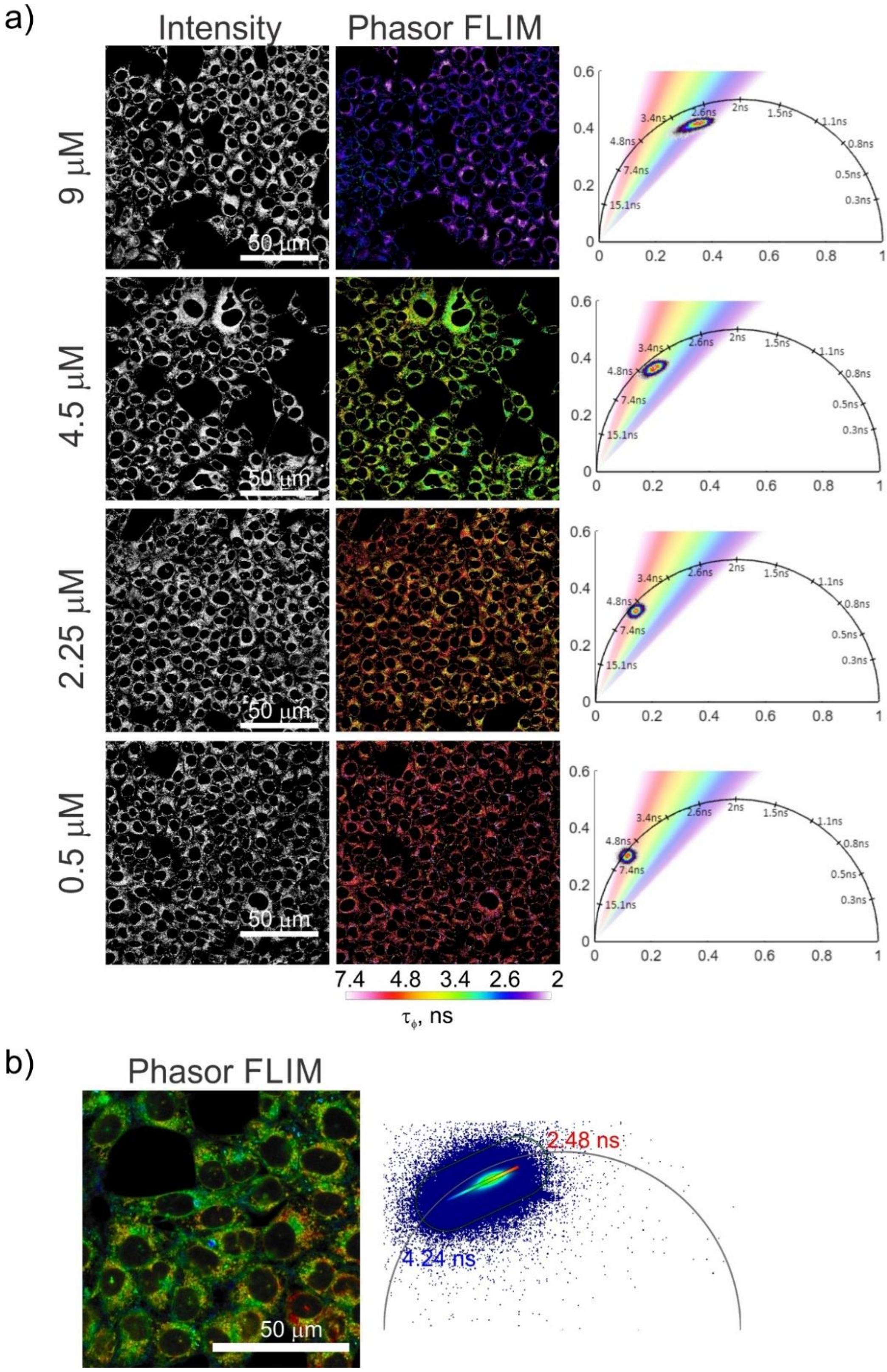

Fig. S19. Microscopy of **5-TEG** -stained human carcinoma HCT116 adherent cells after 2 month of **5-TEG** stock solution storage. (a): Effect of staining concentration (0.5–9 μM) on the intracellular **5-TEG** fluorescence lifetimes. Intensity image and Phasor-FLIM t_{φ}-color coded images with corresponding phasor plots (GS-Lab). (b): Phasor-FLIM hight resolution image of 9 μM **5-TEG** stained cell monolayer with the corresponding phasor plot (LAS X software).

5. Electrochemical Data

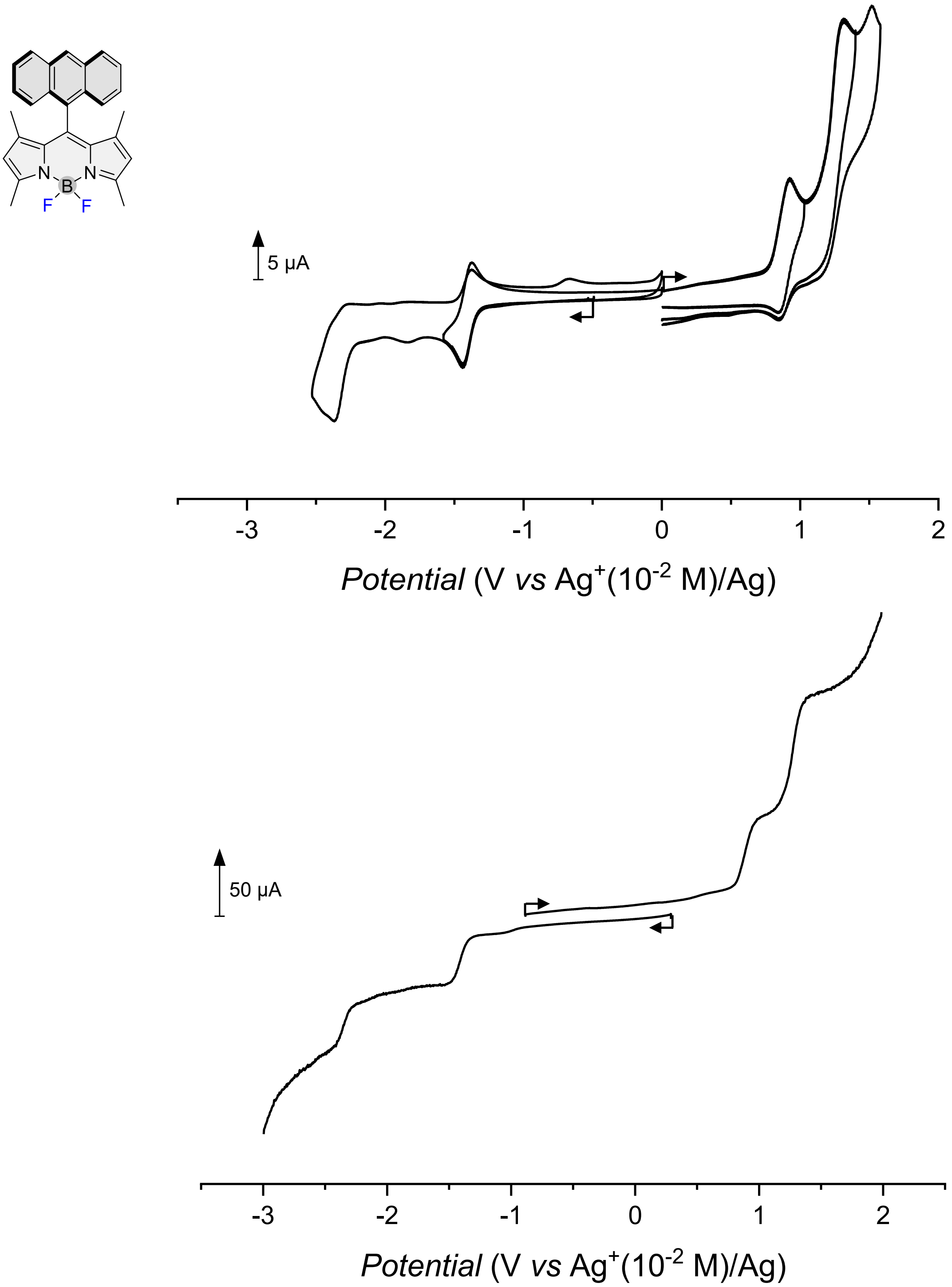

Figure S20. Voltammograms curves of compound **1** (0.5 mM) in 0.1 M TBATFSI/MeCN, recorded at a static glassy carbon working electrode (top) (Ø = 3 mm, v = 100 mV s^{-1}), and at a rotating disk electrode (bottom) (Ø = 5 mm, v = 10 mV s^{-1}, 500 rd min^{-1}). Counter electrode: Pt wire. reference: Ag^{+} (10^{-2} M)/Ag.

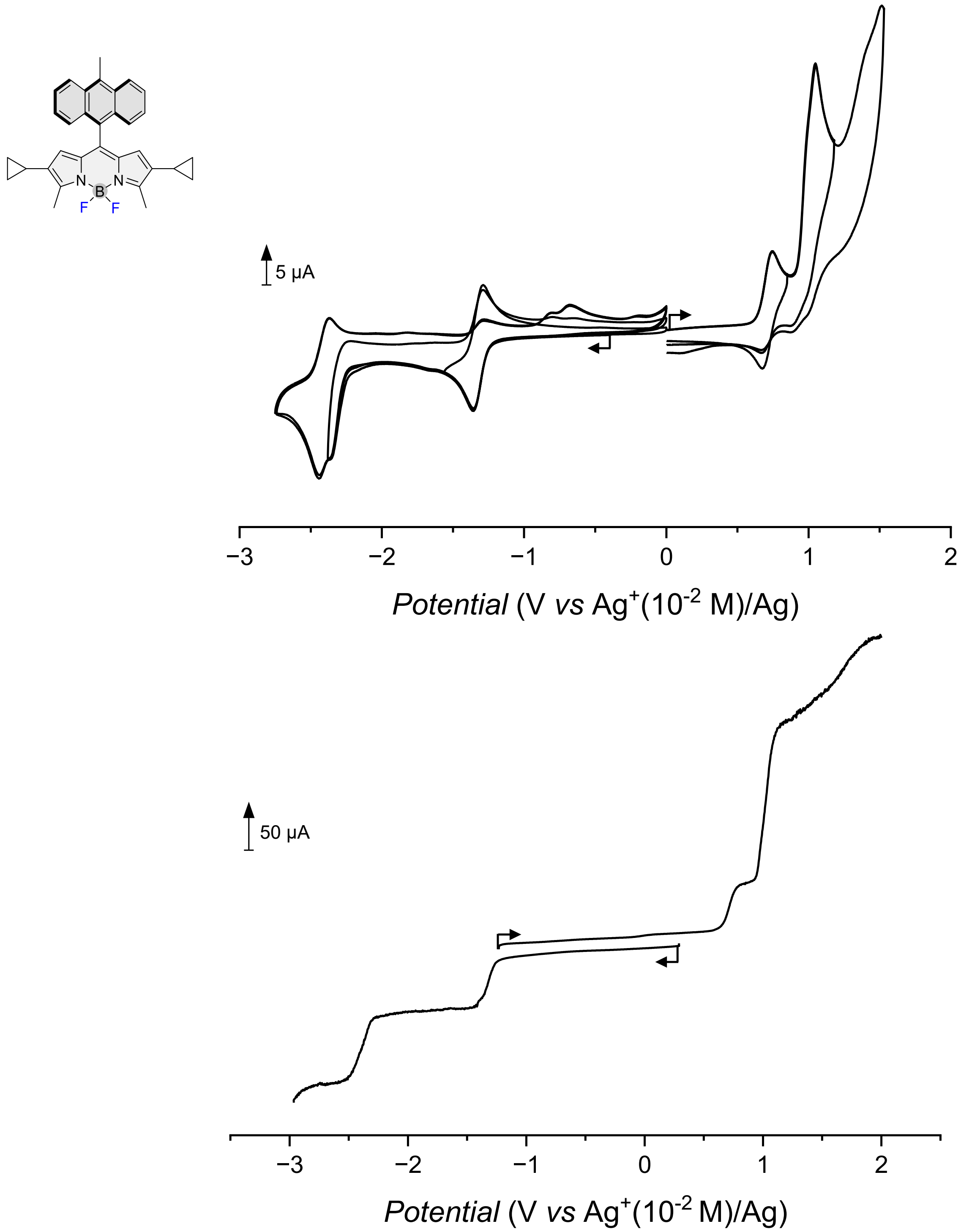

Figure S21. Voltammograms curves of **5** (0.5 mM) in 0.1 M TBATFSI/MeCN, recorded at a static glassy carbon working electrode (top) (Ø = 3 mm, v = 100 mV s^{-1}), and at a rotating disk electrode (bottom) (Ø = 5 mm, v = 10 mV s^{-1}, 500 rd min^{-1}). Counter electrode: Pt wire. reference: Ag^+ (10^{-2} M)/Ag.

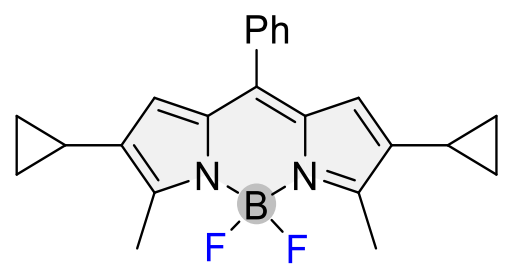

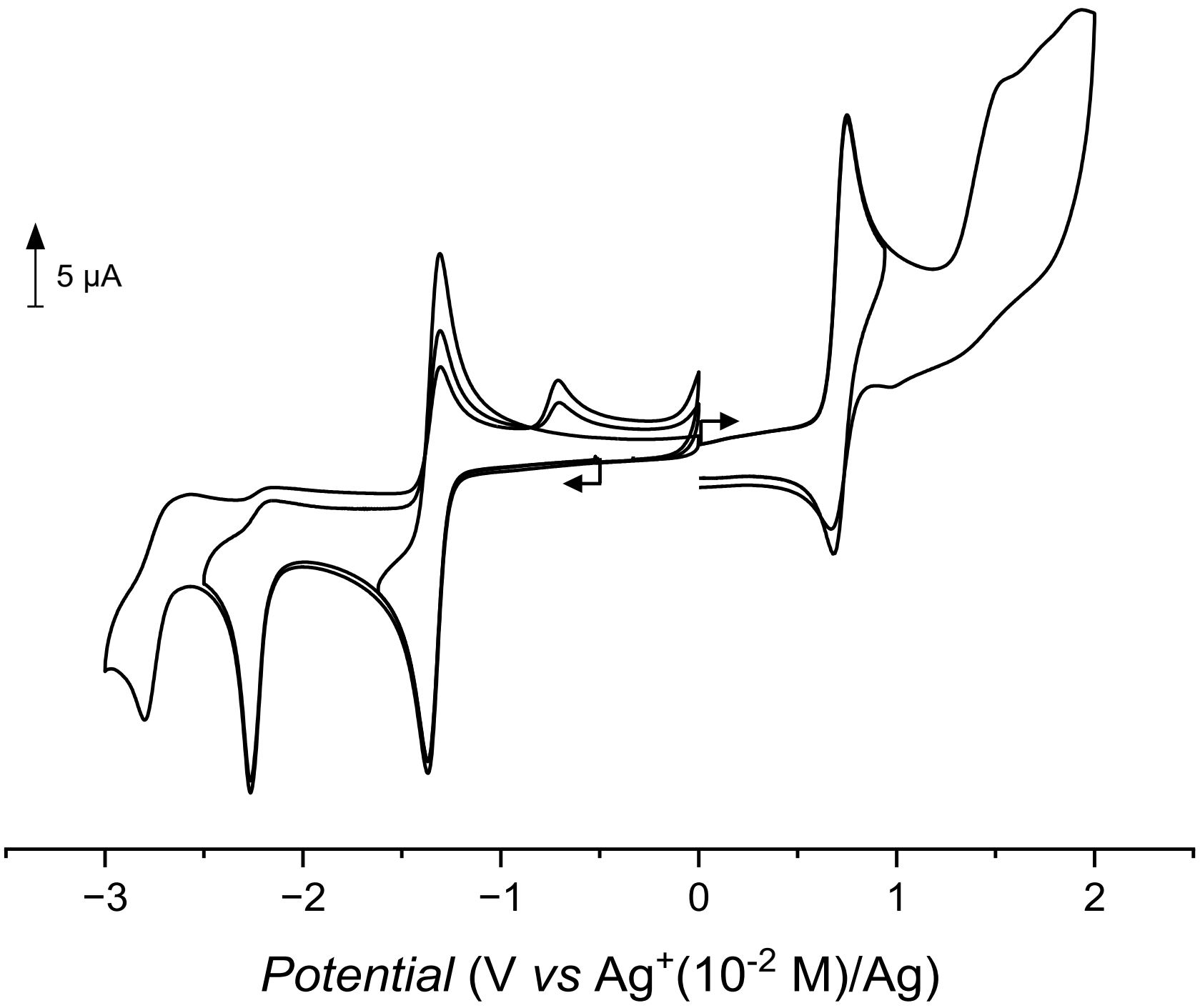

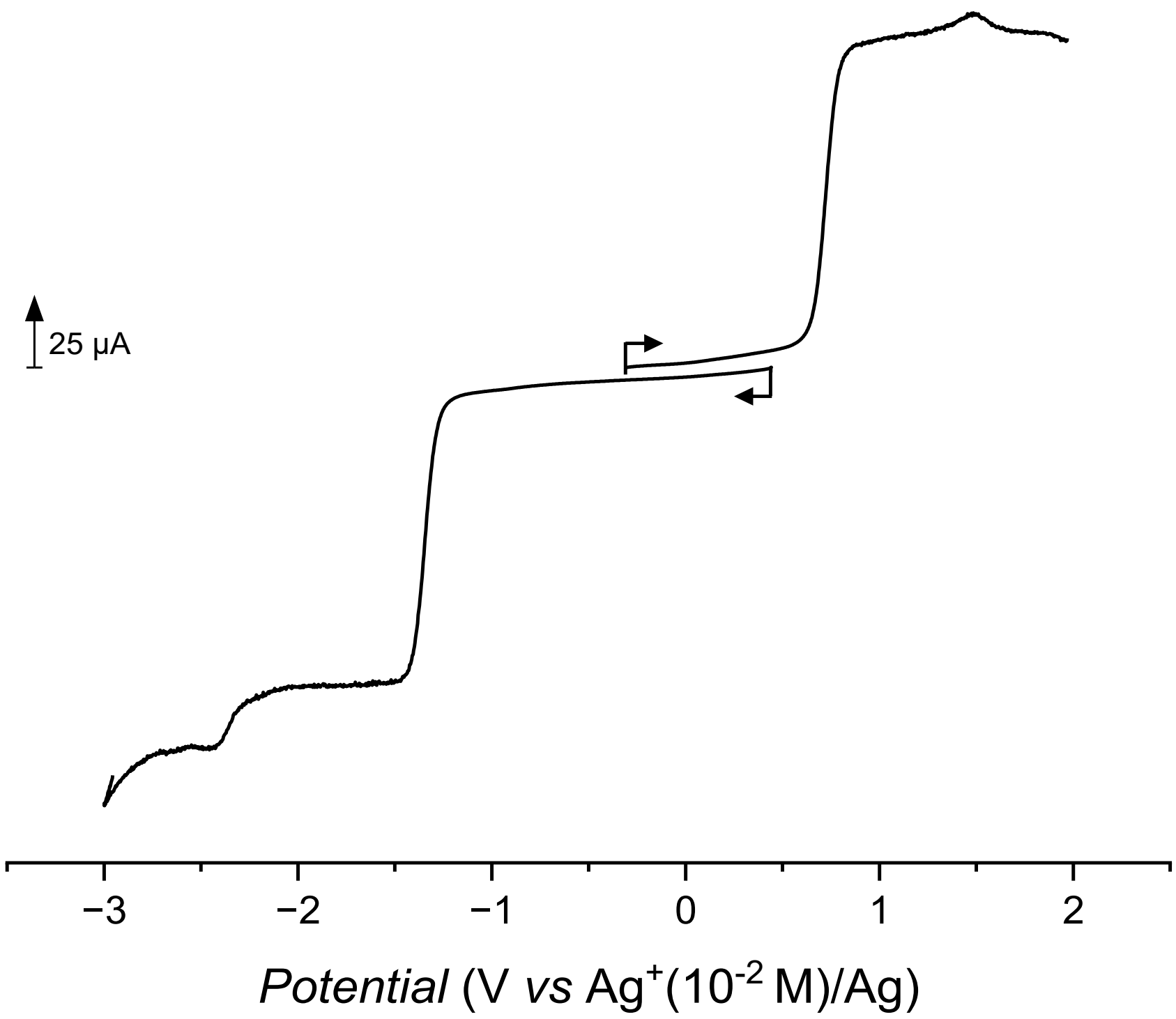

Figure S22. Voltammograms curves of **6** (1.0 mM), in 0.1 M TBATFSI/CH_3CN, recorded at a static glassy carbon working electrode (top) (Ø = 3 mm, *v* = 100 mV s^{-1}), and at a rotating disk electrode (bottom) (Ø = 5 mm, *v* = 10 mV s^{-1}, 500 rd min^{-1}). Counter electrode: Pt wire. reference: Ag^+ (10^{-2} M)/Ag.

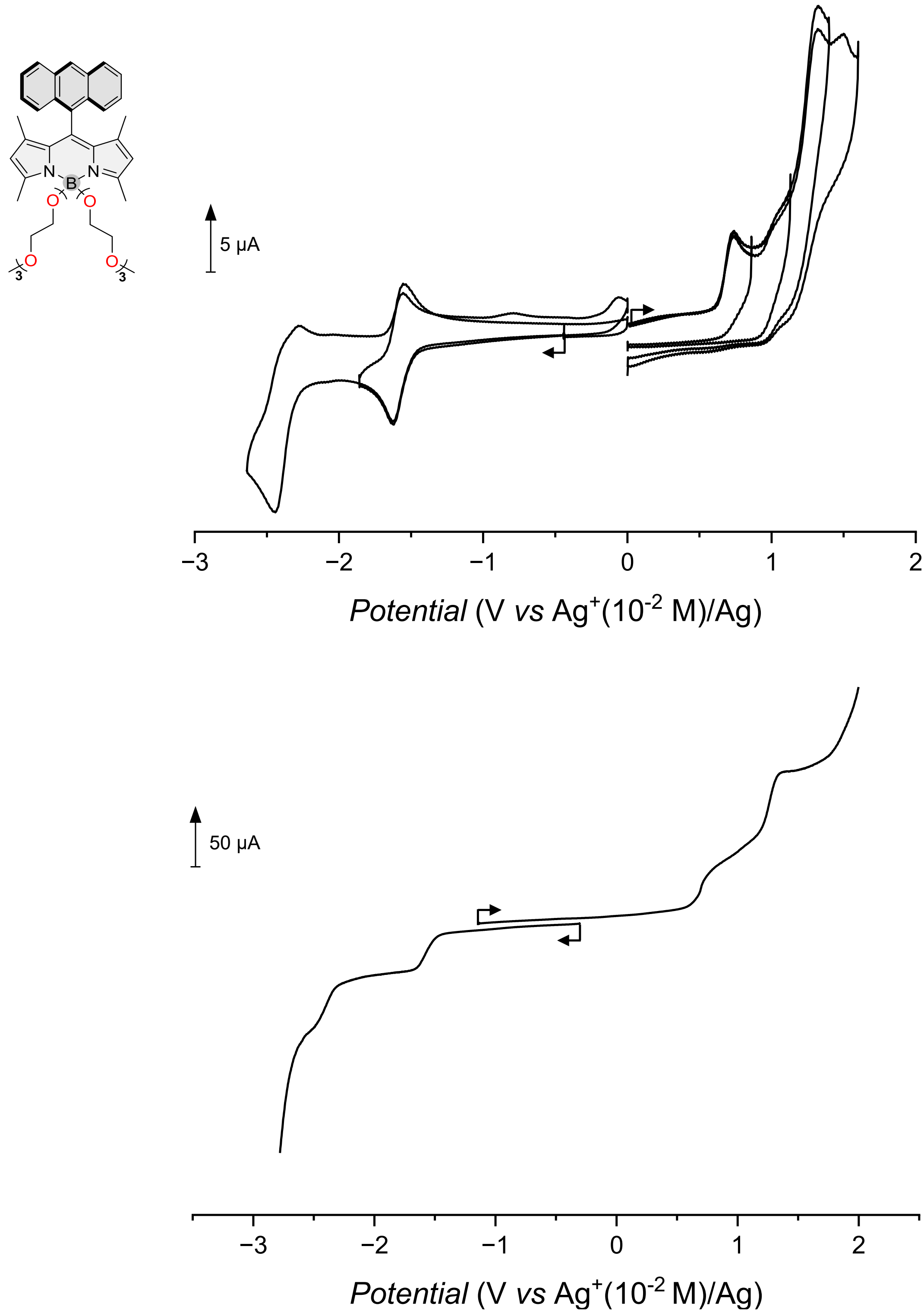

Figure S23. Voltammograms curves of **1-TEG** (0.5 mM), in 0.1 M TBATFSI/MeCN, recorded at a static glassy carbon working electrode (top) (Ø = 3 mm, v = 100 mV s^{-1}), and at a rotating disk electrode (bottom) (Ø = 5 mm, v = 10 mV s^{-1}, 500 rd min^{-1}). Counter electrode: Pt wire. reference: Ag^+ (10^{-2} M)/Ag.

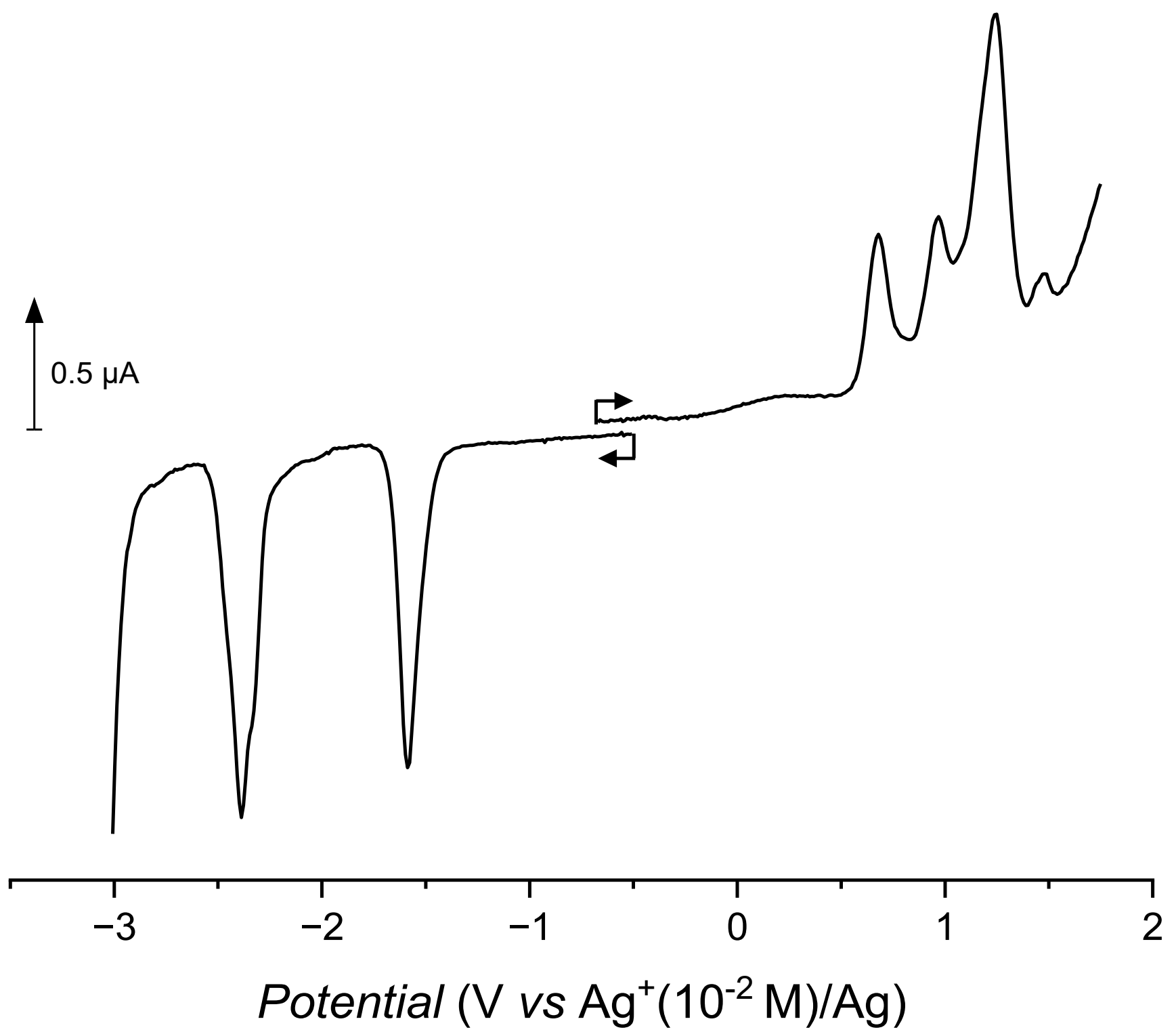

Figure S24. DPV curves of **1-TEG** (0.5 mM), in 0.1 M TBATFSI/MeCN, recorded at a glassy carbon working electrode (Ø = 3 mm, *v* = 20 mV s^{-1}), Counter electrode: Pt wire, reference: Ag^+ (10^{-2} M)/Ag.

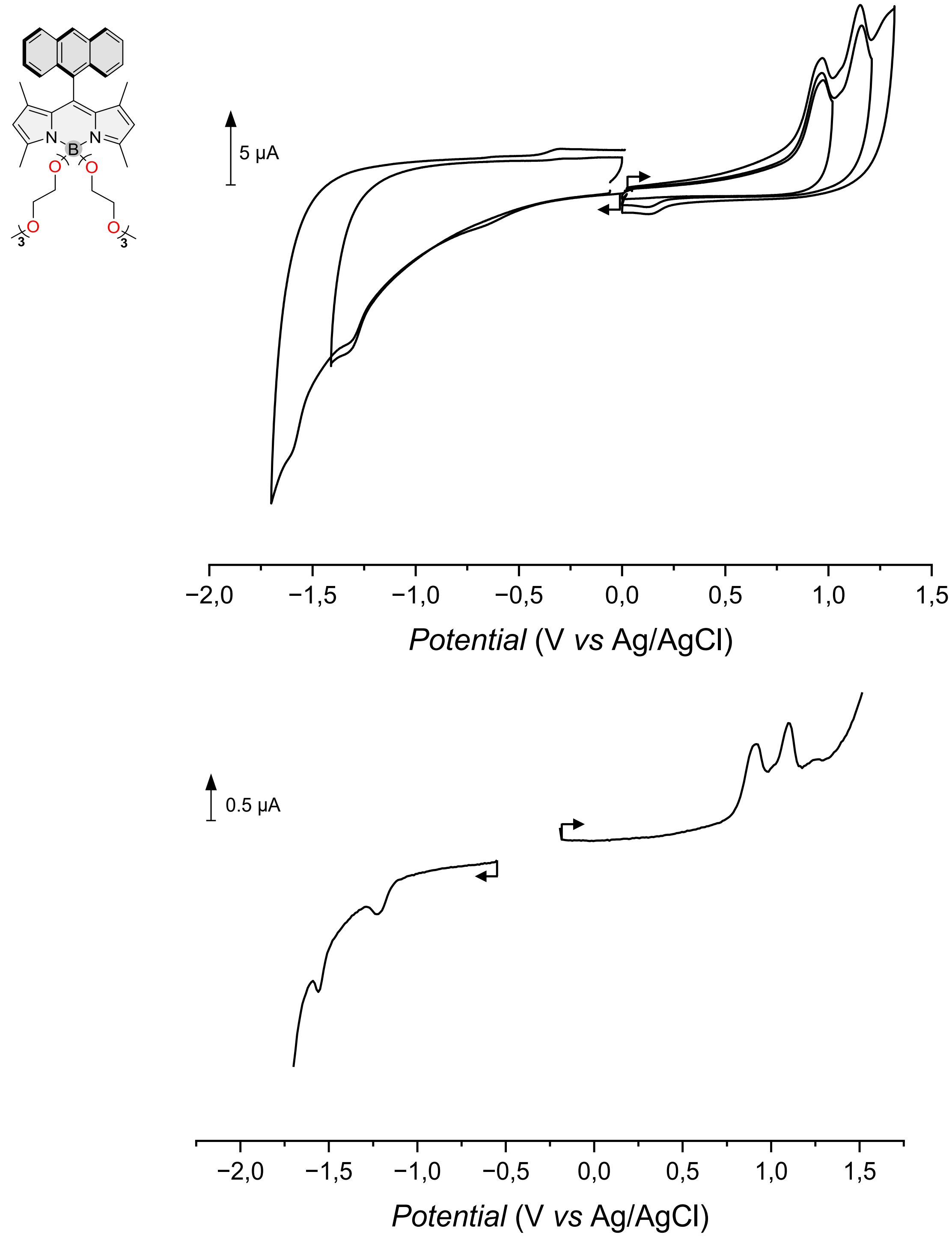

Figure S25. Cyclic voltammograms curves of **1-TEG** (0.5 mM), in 0.1 M LiTFSI/H_2O, recorded at a static glassy carbon working electrode (top), and DPV curves (bottom) (Ø = 3 mm, v = 100 mV s^{-1}). Counter electrode: Pt wire. reference: (Ag^+/AgCl).

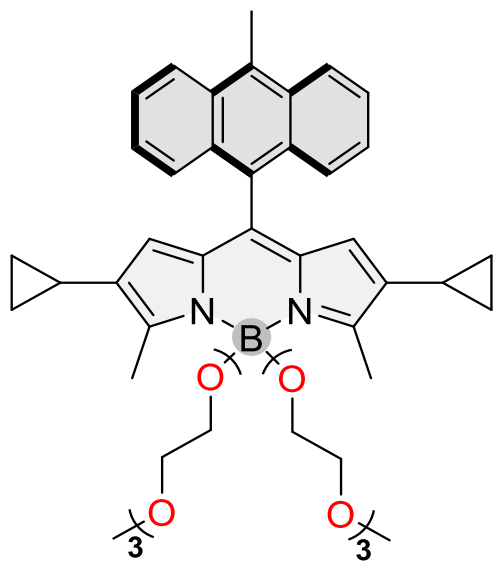

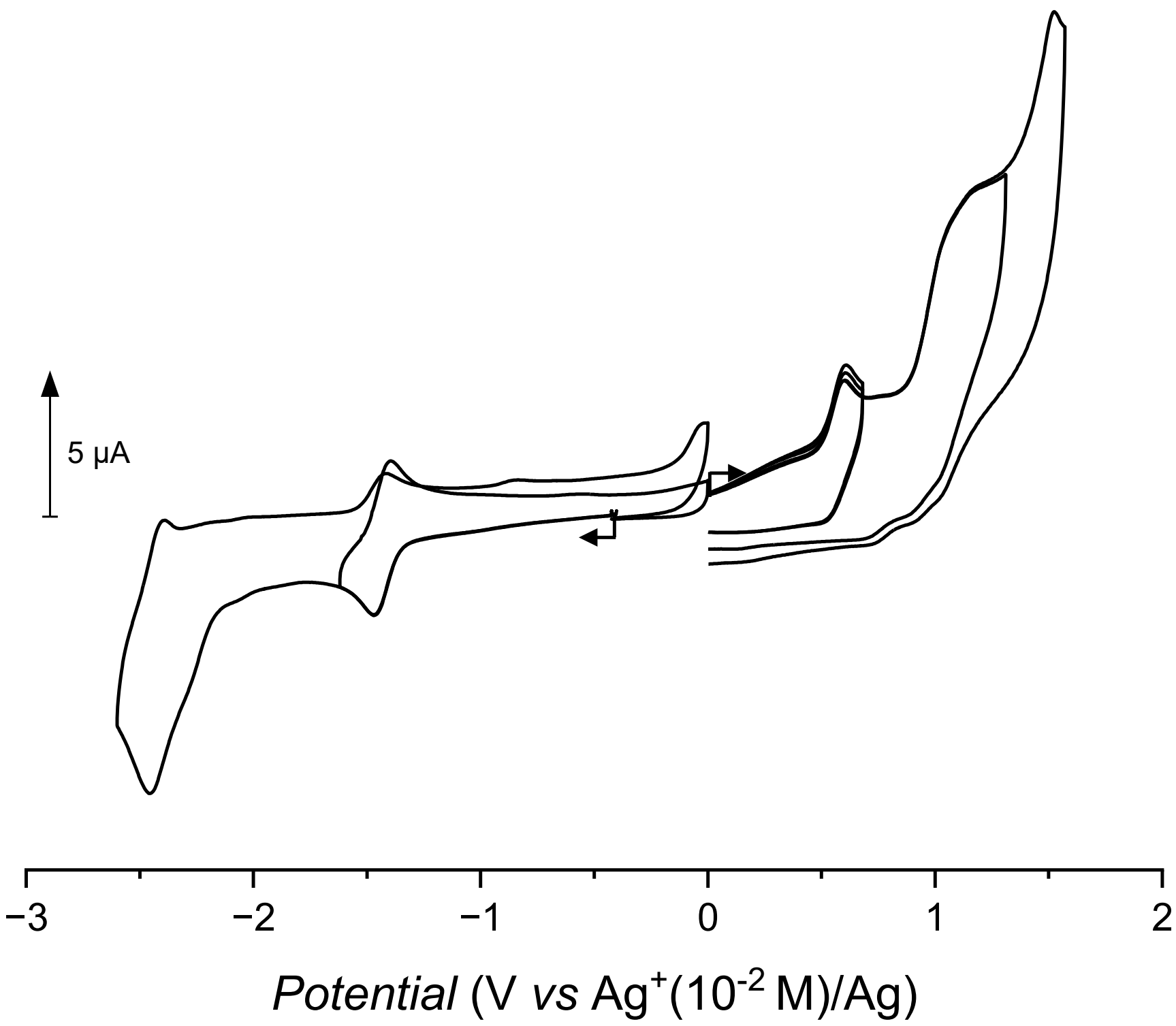

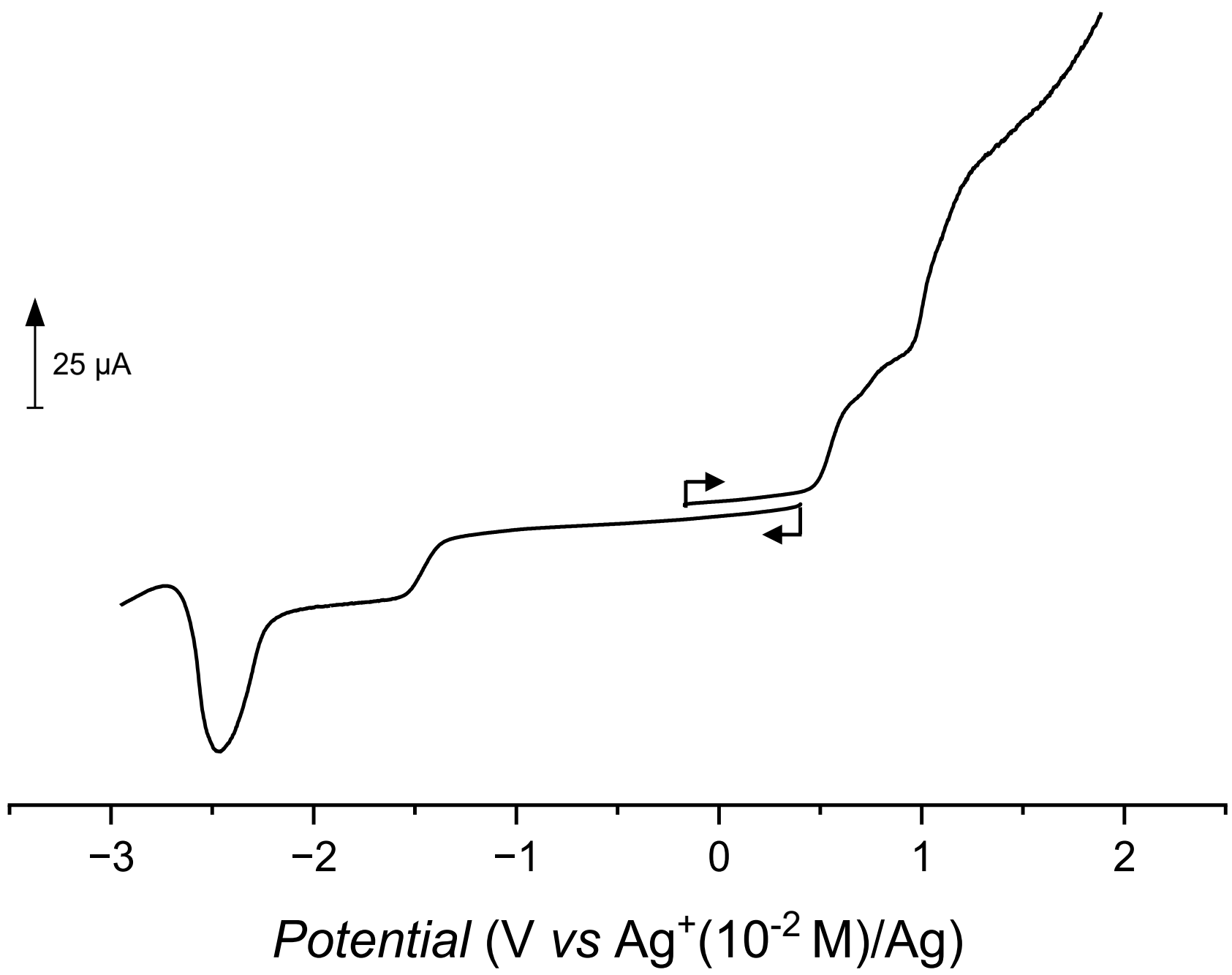

Figure S26. Voltammograms curves of **5-TEG** (0.2 mM), in 0.1 M TBATFSI/MeCN, recorded at a static glassy carbon working electrode (top) (Ø = 3 mm, v = 100 mV s^{-1}), and at a rotating disk electrode (bottom) (Ø = 5 mm, v = 10 mV s^{-1}, 500 rd min^{-1}). Counter electrode: Pt wire. reference: Ag^{+} (10^{-2} M)/Ag.

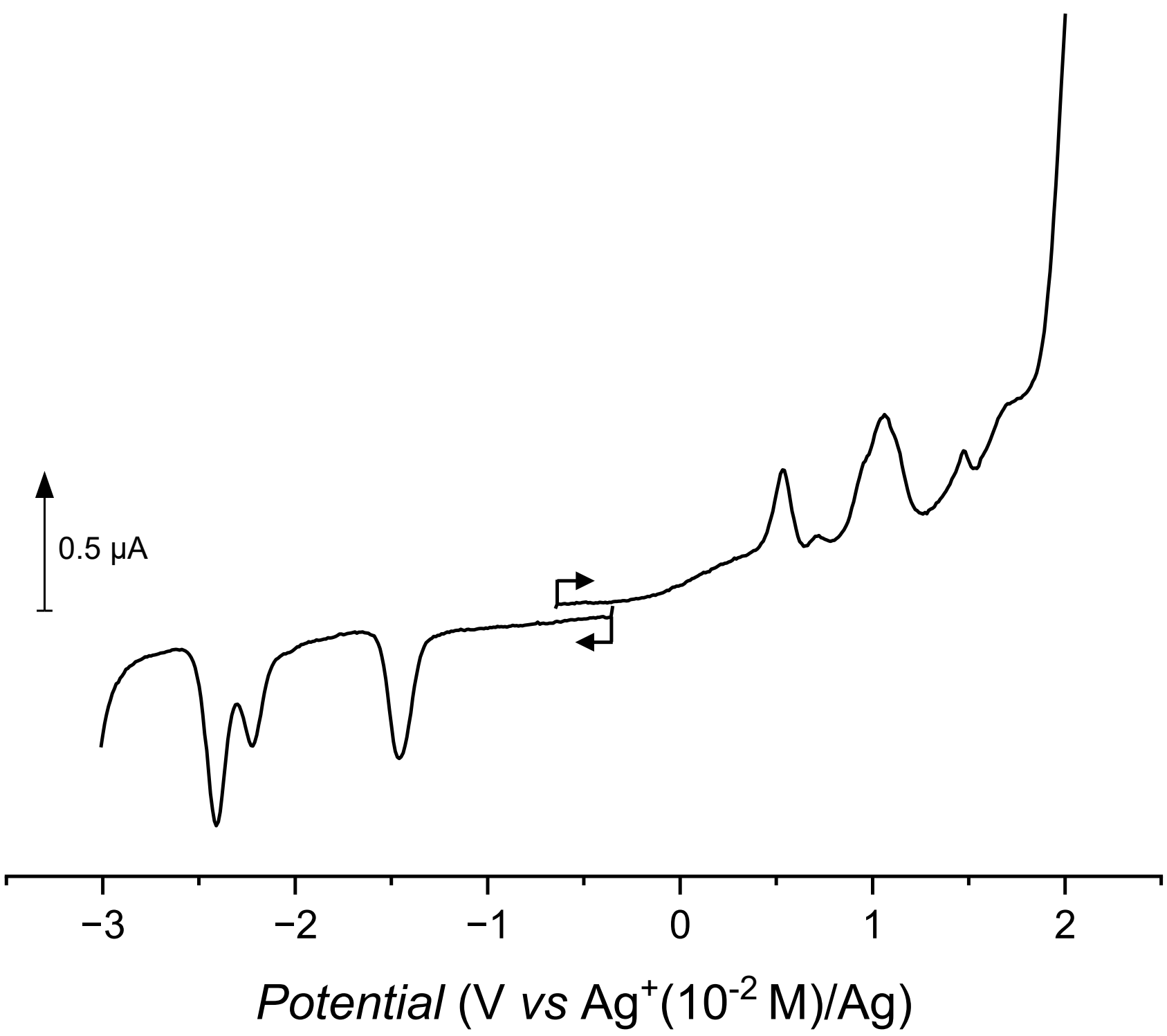

Figure S27. DPV curves of 5**-TEG** (0.2 mM), in 0.1 M TBATFSI/MeCN, recorded at a glassy carbon working electrode (Ø = 3 mm, v = 20 mV s^{-1}), Counter electrode: Pt wire, reference: Ag^{+} (10^{-2} M)/Ag.

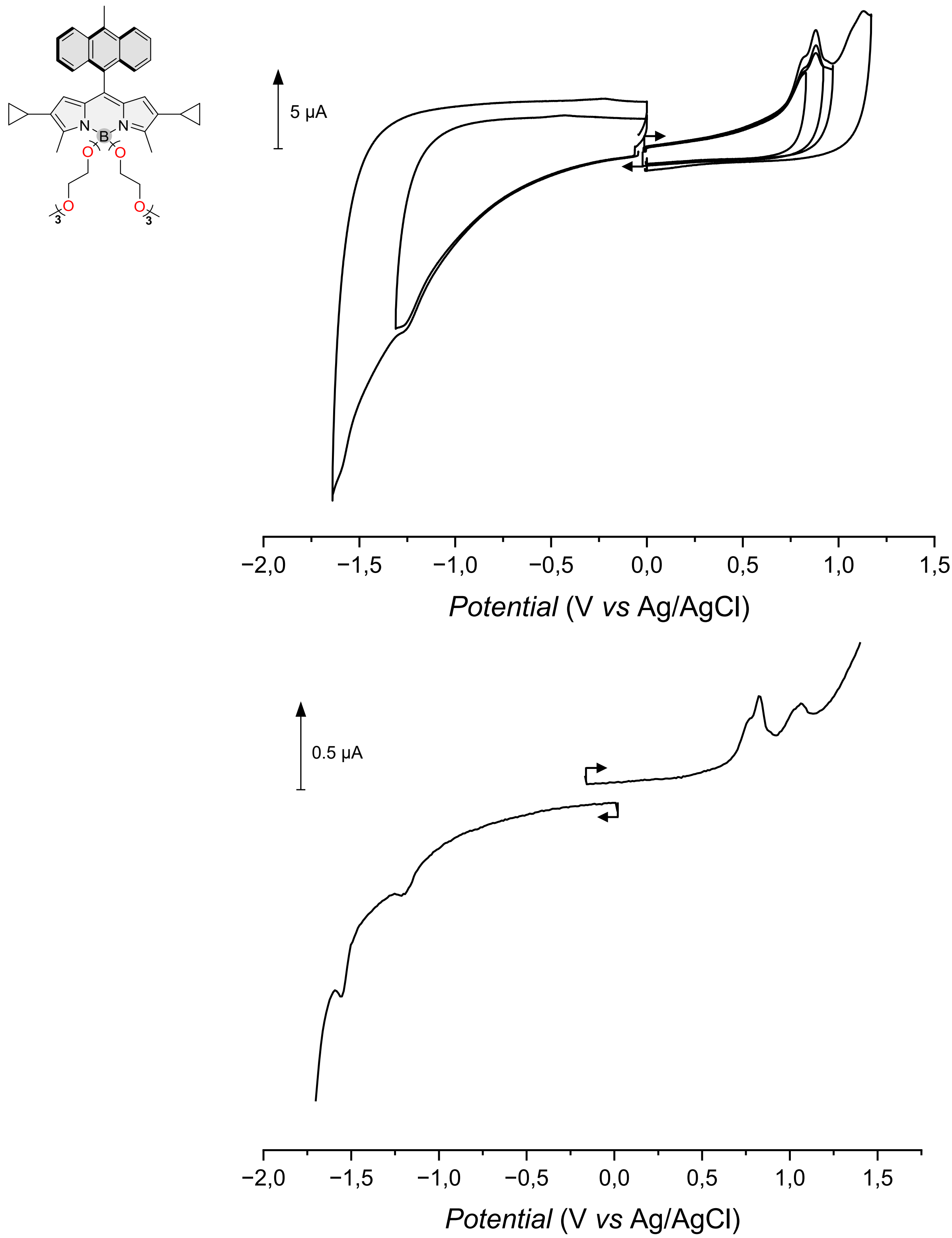

Figure S28. Cyclic voltammograms curves of **5-TEG** (0.2 mM), in 0.1 M LiTFSI/H_2O, recorded at a static glassy carbon working electrode (top) (Ø = 3 mm, v = 100 mV s^{-1}), and DPV curves (bottom) (Ø = 3 mm, v = 20 mV s^{-1}). Counter electrode: Pt wire. reference: (Ag^+/AgCl).

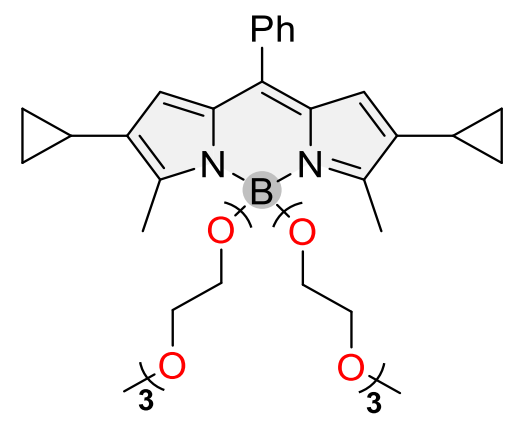

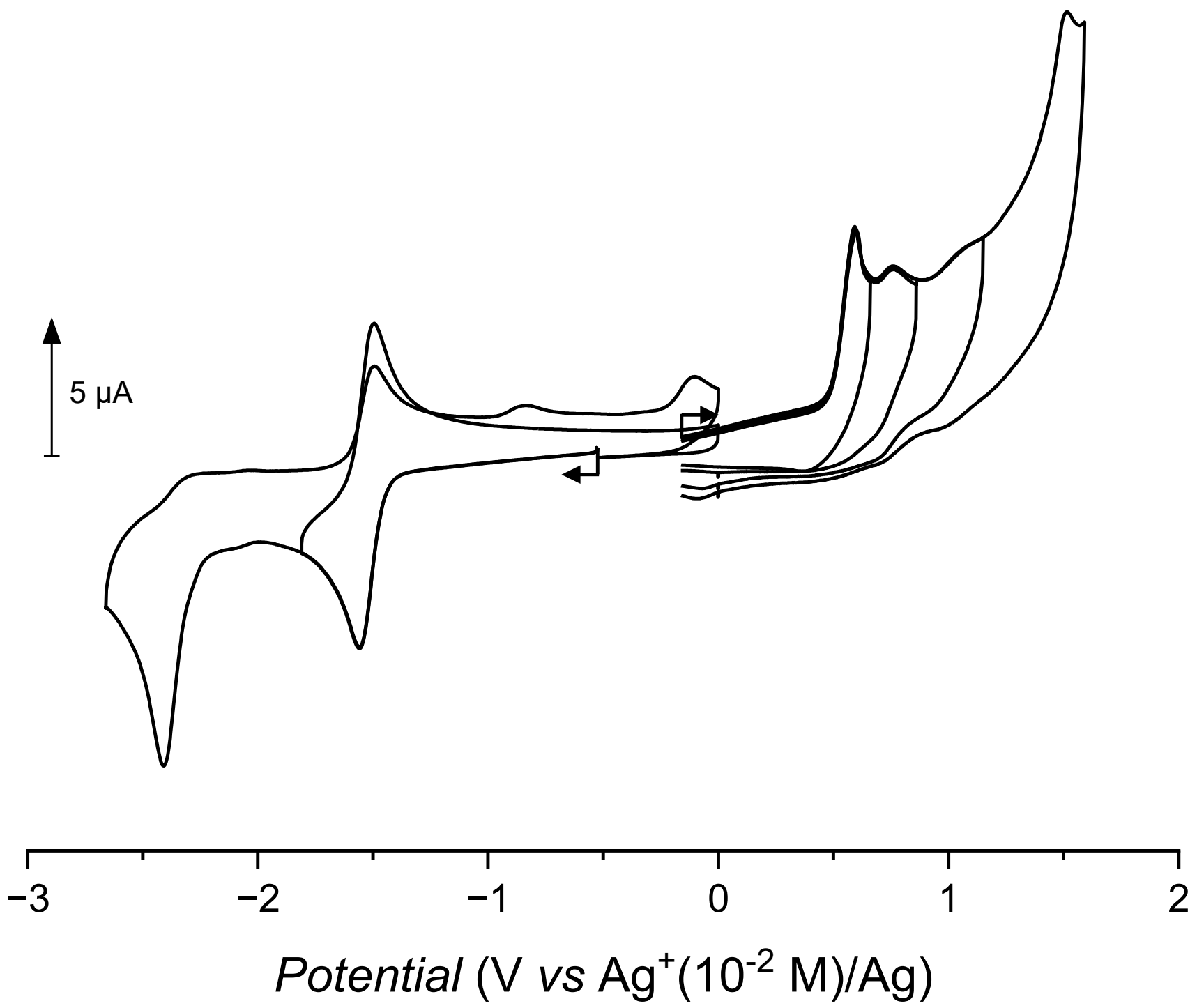

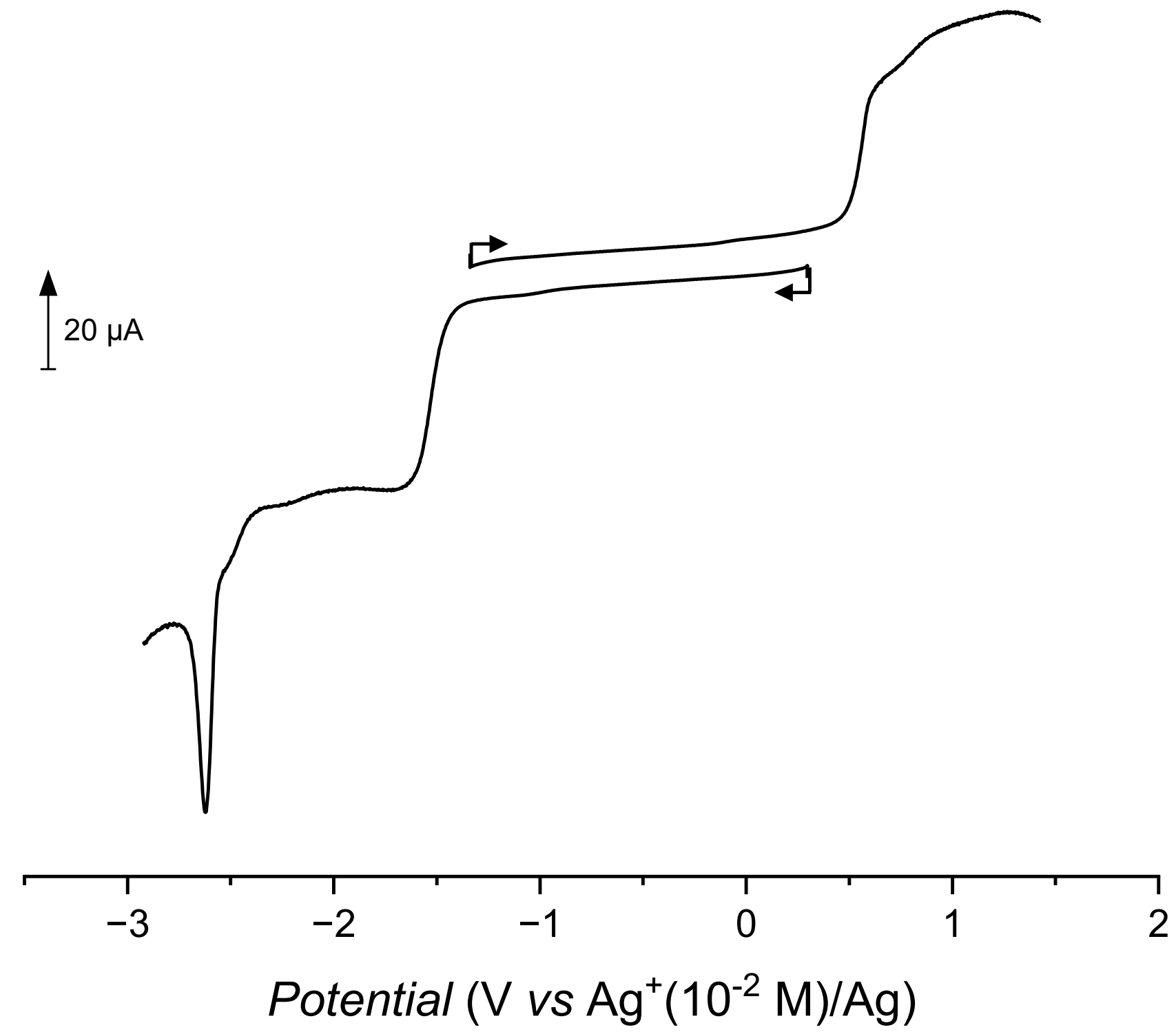

Figure S29. Voltammograms curves of **6-TEG** (0.2 mM), in 0.1 M TBATFSI/MeCN, recorded at a static glassy carbon working electrode (**top**) (Ø = 3 mm, *v* = 100 mV s^{-1}), and at a rotating disk electrode (bottom) (Ø = 5 mm, *v* = 10 mV s^{-1}, 500 rd min^{-1}). Counter electrode: Pt wire. reference: Ag^{+} (10^{-2} M)/Ag.

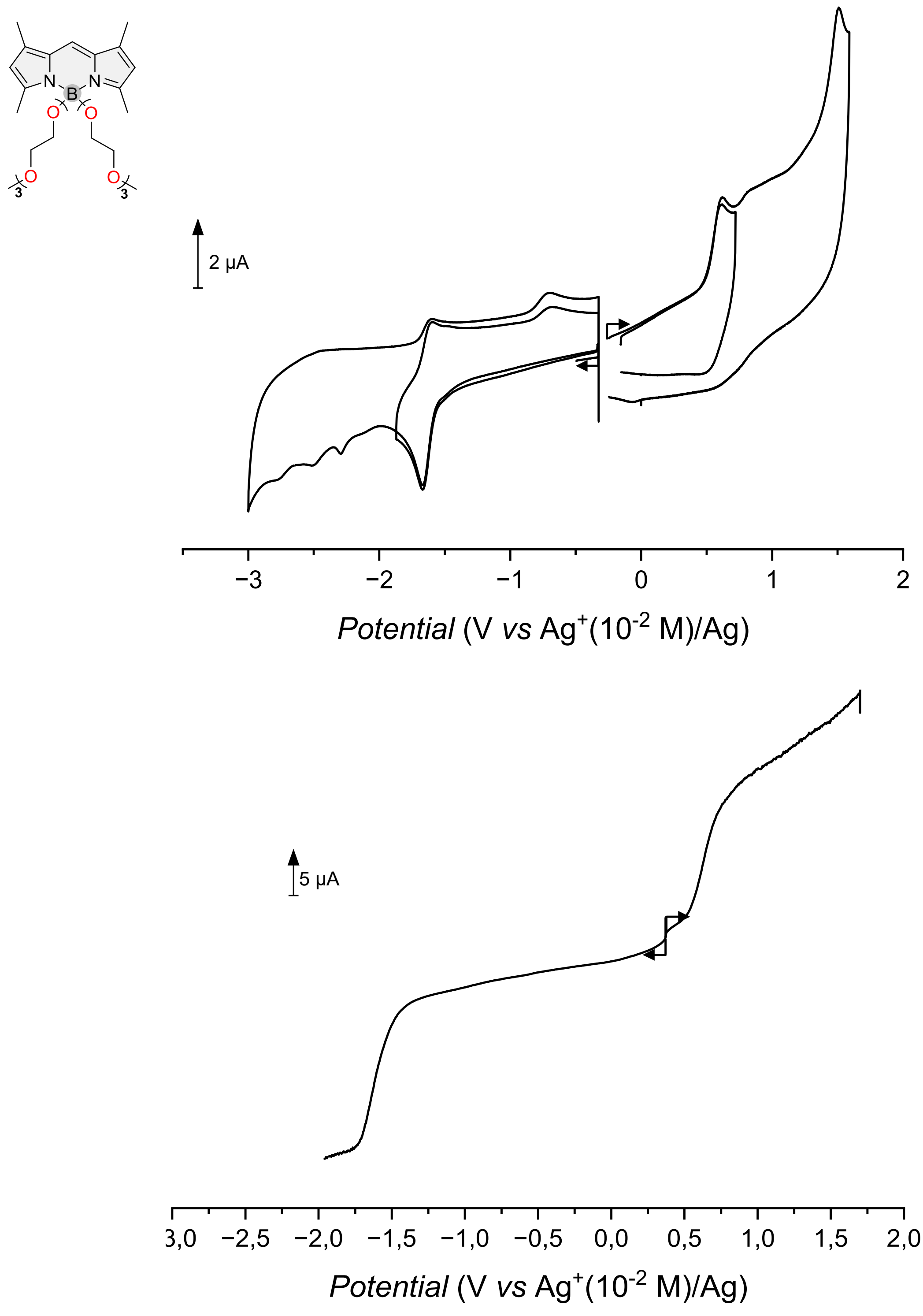

Figure S30. Voltammograms curves of **7-TEG** (0.2 mM), in 0.1 M TBATFSI/MeCN, recorded at a static glassy carbon working electrode (top) (Ø = 3 mm, v = 100 mV s^{-1}), and at a rotating disk electrode (**bottom**) (Ø = 5 mm, v = 10 mV s^{-1}, 500 rd min^{-1}). Counter electrode: Pt wire. reference: Ag^+ (10^{-2} M)/Ag.

Table S16. Electrochemical data (peak potentials[a] or half-wave potentials[b]) of the investigated BODIPYs in acetonitrile and water. The half-wave of the reduction potential values given in volts ($E_{1/2}$), the associated peak to peak potential shifts ΔEp=|Epa - Epc| are given in mV. [a] Peak potential value (E_{pa} or E_{pc} in V) reported for irreversible waves. The redox potentiels are reported in Volts (V) *vs* E_{ref}[Fc^+/Fc] used as an internal standard(IS) in CH_3CN. (In aqueous medium, Ferrocene-methanol was used as an internal standard, ΔE (Fc-methanol − Fc) = 5 mV).

**BODIPY**	**Solvent**	**Reduction (V *vs* Fc^+/Fc)**			**Oxidation (V *vs* Fc^+/Fc)**				**ΔE**
		$E_{1/2}$(red1)[b], ΔE_p (mV)	$E_{1/2}$(red2)[b], ΔE_p (mV)	$E_{1/2}$(red3), ΔE_p (mV)	E_{pa}(ox1)[a], ΔE_p (mV)	E_{pa}(ox2)[a]	E_{pa}(ox 3)[a]	E_{pa}(ox 4)	
**1**	MeCN	–1.495 (67)	–2.456[a]	–	0.832 (79)[b]	1.215	1.438	–	2.327
**1-TEG**	MeCN	–1.687 (72)	–2.529[a]	–	0.645	0.971	1.224	1.404	2.332
**1-TEG**	H_2O	–1.554[a]	–1.840[a]	–	0.722	0.907	1.027	–	2.276
**5**	MeCN	–1.407 (70)	–2.440[a]	–2.491(68)	0.659 (71)[b]	0.966	1.365	–	2.066
**5-TEG**	MeCN	–1.520 (73)	–2.453[a]	–	0.511	1.000	1.437	–	2.031
**5-TEG**	H_2O	–1.495[a]	–1.833[a]	–	0.562	0.640	0.888	–	2.057
**6**	MeCN	−1.430 (62)	−2.35	−2.89	0.627 (62)	−	−	−	2.057
**6-TEG**	MeCN	−1.614 (69)	−2.495	−	0.506	0.685	−	−	2.120
**7-TEG**	MeCN	−1.727 (58)	−	−	0.521	1.417	−	−	2.248

Estimation of the driving force for photoinduced electron transfer

The standard free energy change for photoinduced charge separation, $\Delta G^{\circ}_{\mathrm{PET}}$, was estimated using a Rehm–Weller-type relationship.[30]

$$\Delta G^{\circ}_{\mathrm{PET}} = E_{\mathrm{ox}}(D/D^{\cdot+}) - E_{\mathrm{red}}(A/A^{\cdot-}) + w - E_{00}$$

where $E_{\mathrm{ox}}(D/D^{\cdot+})$ is the oxidation potential of the donor fragment and $E_{\mathrm{red}}(A/A^{\cdot-})$ is the reduction potential of the acceptor fragment (both in volts vs Fc^+/Fc), $E_{00}$is the 0–0 excitation energy (in eV), and $w$is the Coulombic work term (in eV). The Coulomb term was approximated as:

$$w = -\frac{e^2}{4\pi\varepsilon_0\varepsilon_r r_{DA}} = -\frac{14.40}{\varepsilon_r\, r_{DA}(\text{Å})}\ \text{eV}$$

with ε_r the dielectric constant of the solvent and r_{DA} the donor–acceptor separation distance. The constant 14.40 corresponds to $e^2/(4\pi\varepsilon_0)$expressed in eV·Å.

Excited-state energy. E_{00} was obtained from the intersection (“cross-point”) of the normalized absorption and fluorescence spectra and converted using:

$$E_{00}(\text{eV}) = \frac{1240}{\lambda_{00}(\text{nm})}$$

Electrochemical potentials. Oxidation and reduction potentials were taken from cyclic voltammetry in the relevant solvent and are reported vs Fc^+/Fc. For donor-acceptor dyads, donor- and BODIPY-centred oxidation waves were assigned by comparison to the corresponding reference BODIPYs lacking the donor unit (compounds **6**, **6-TEG**, **7** and **7-TEG**).

Donor–acceptor distances (r_{DA}). Center-to-center distance were estimated from the computed DFT geometries. The following r_{DA}values were used in the present work:

- BODIPY–anthracene dyads **1** and **1-TEG**: $r_{DA} = 4.40$ Å.
- Where relevant for comparison, literature values based on electron–hole separation from transition-density analysis (e.g., $= 3.34$ Å for dyad **1**) were used.[31]
- MeAnt dyads: $r_{DA} = 4.607$ Å for compound **5** and $r_{DA} = 4.95$ Å for **5-TEG**.

$\varepsilon_r$values of 37.5 (MECN) and 78.4 (water) were used.

6. NMR and High-Resolution Mass Spectra

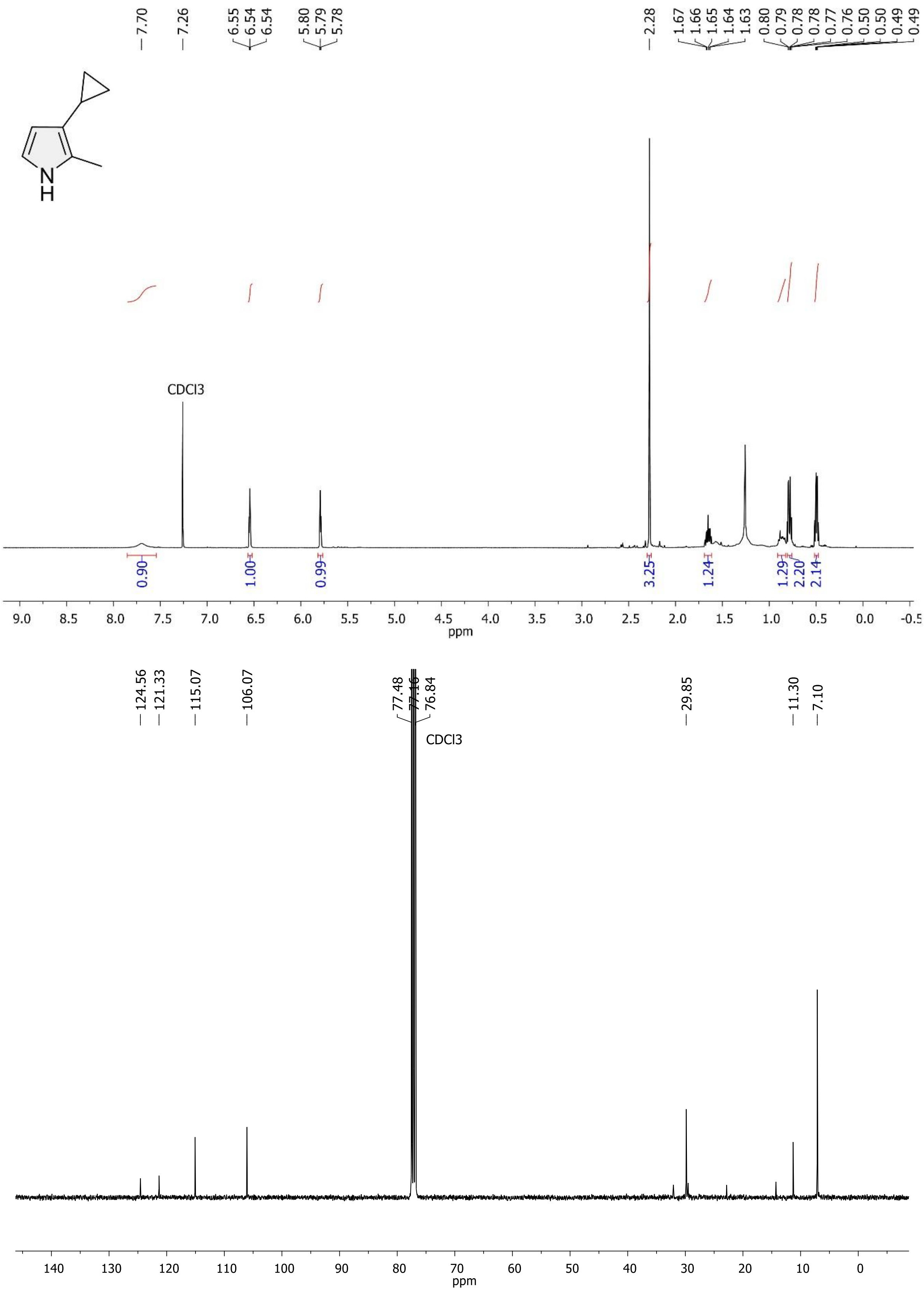

Figure S31. ^{1}H and ^{13}C NMR spectra of compound **P1**.

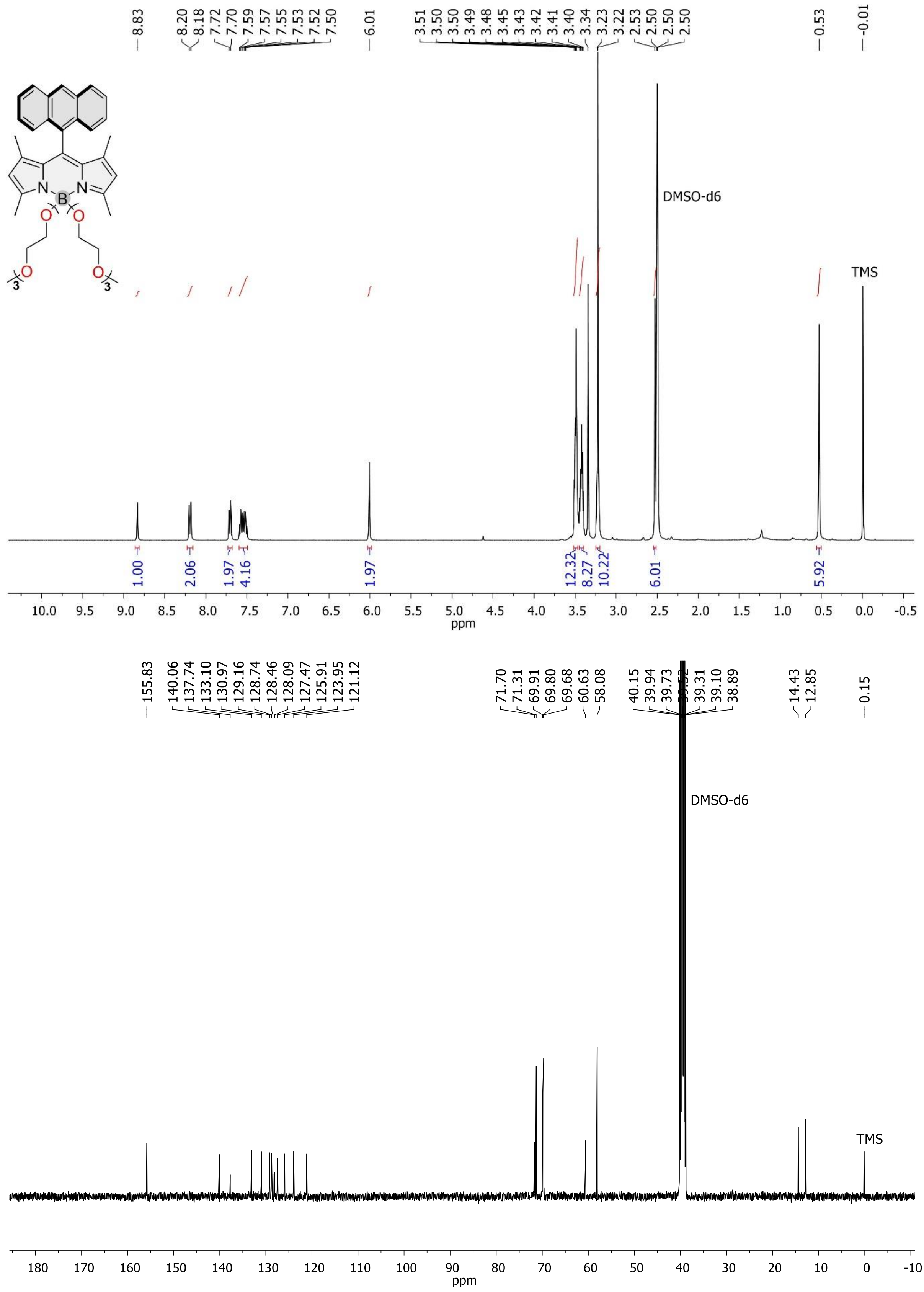

Figure S32. ^1H and ^{13}C NMR spectra of compound **1-TEG**.

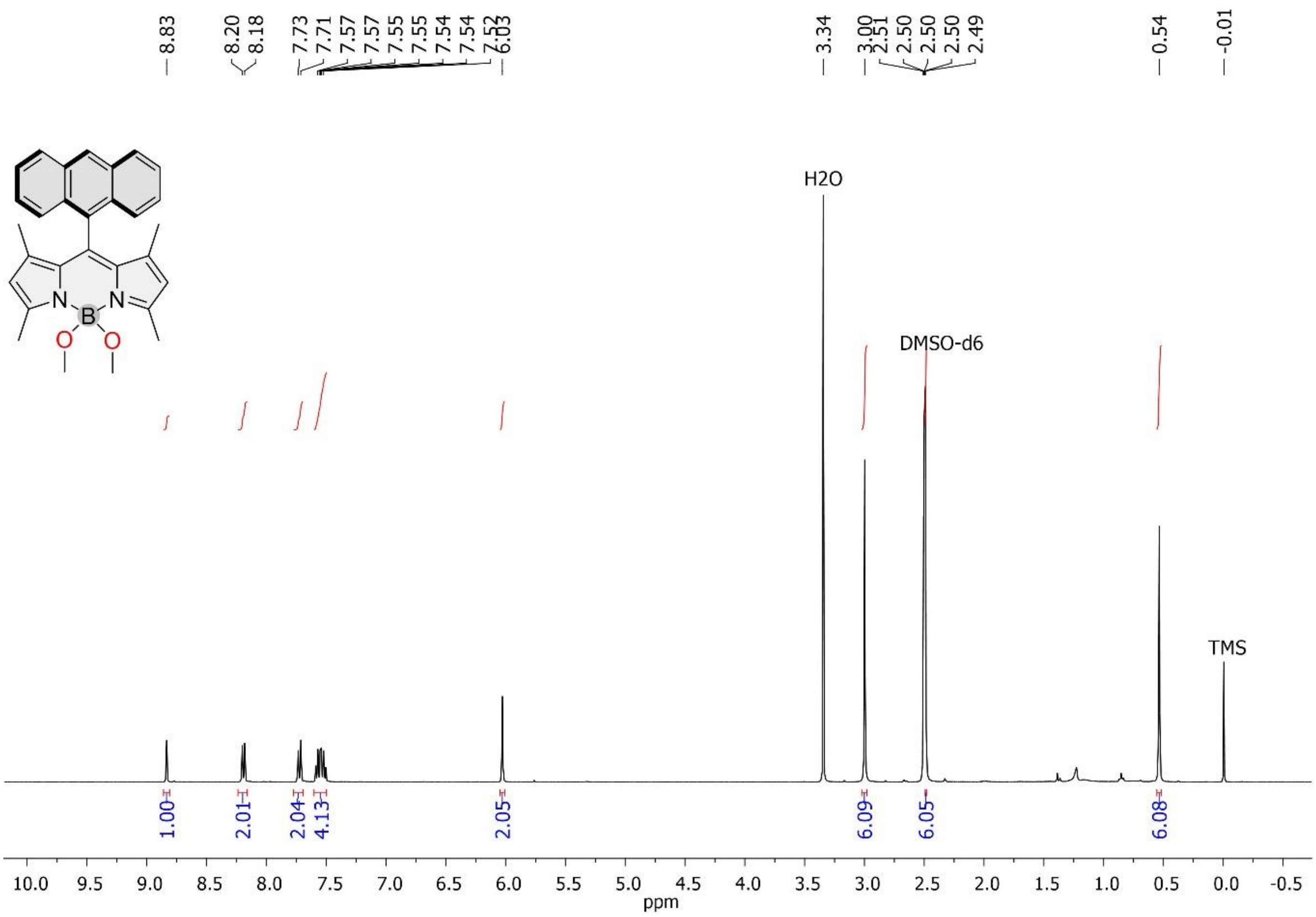

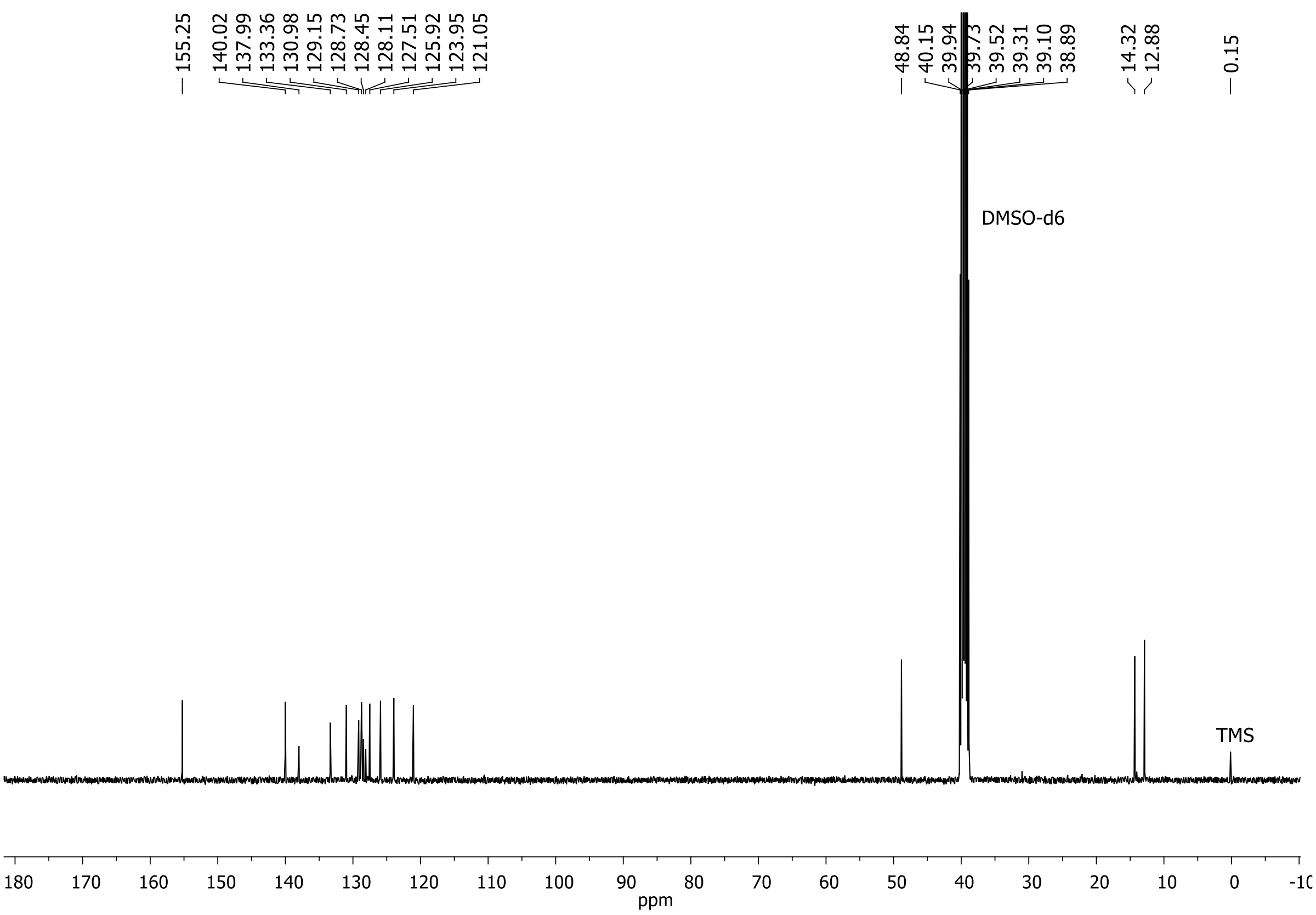

Figure S33. ^{1}H and ^{13}C NMR spectra of compound **1-OMe**.

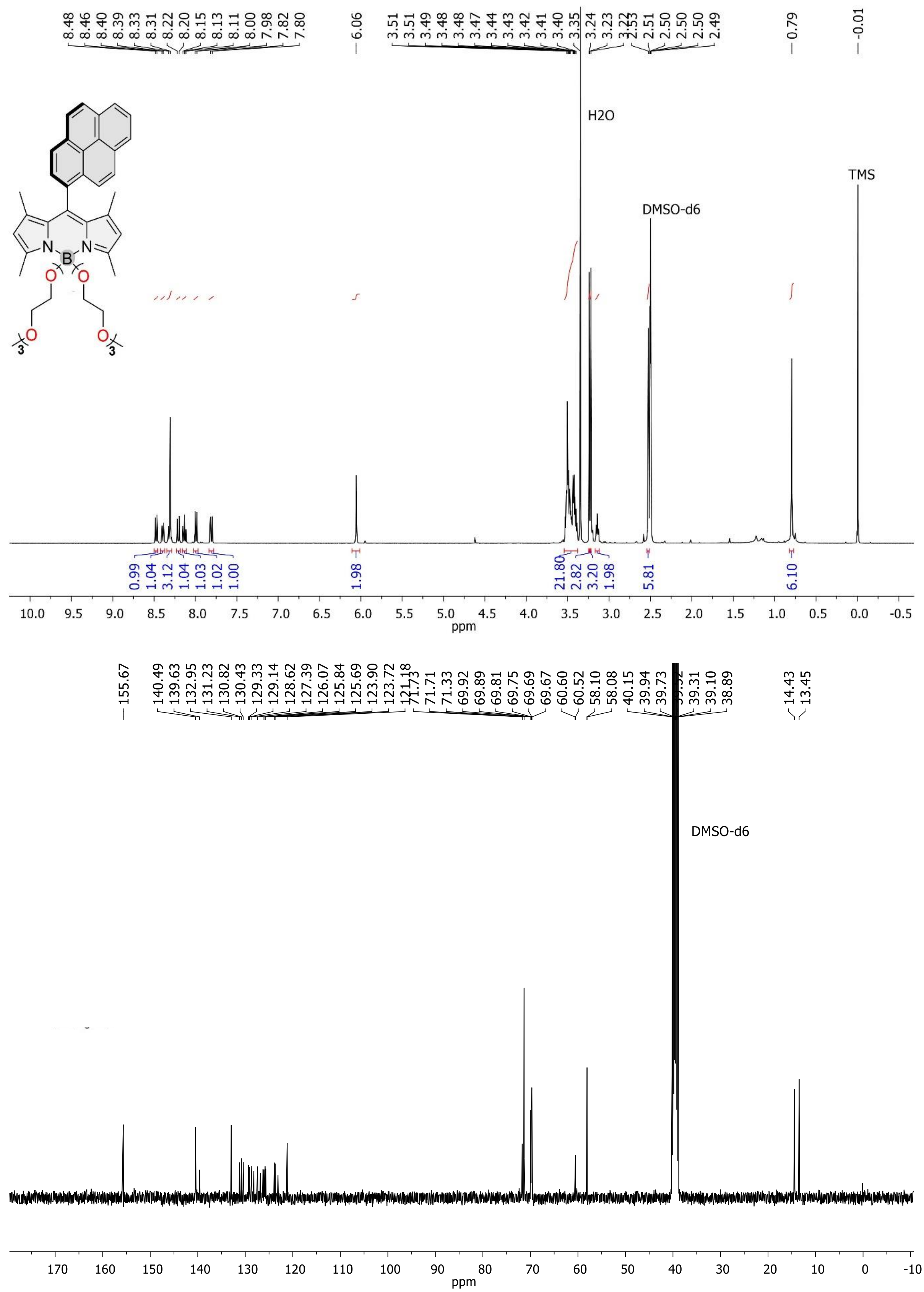

Figure S34. ^{1}H and ^{13}C NMR spectra of compound **2-TEG**.

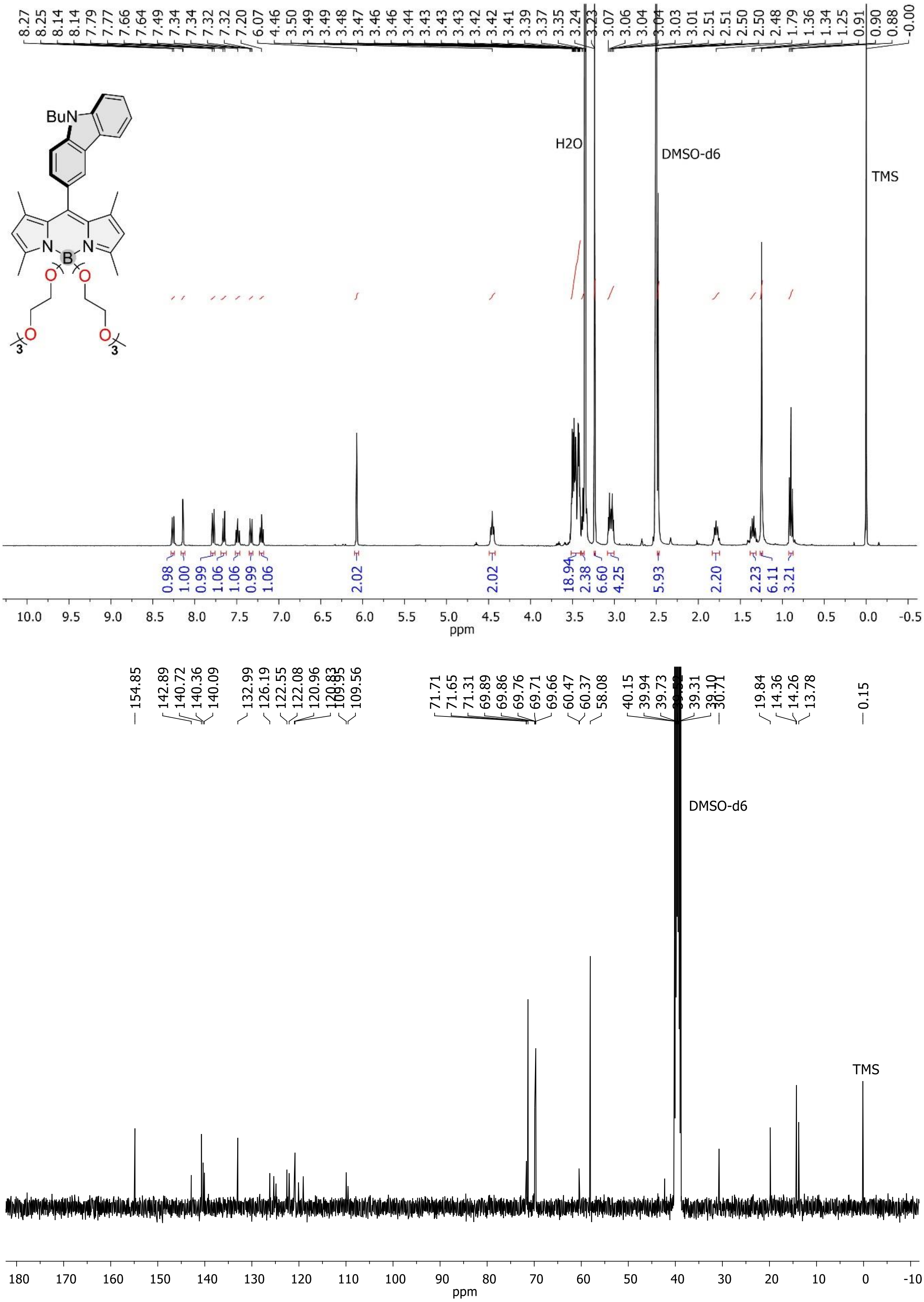

Figure S35. ^{1}H and ^{13}C NMR spectra of compound **3-TEG**.

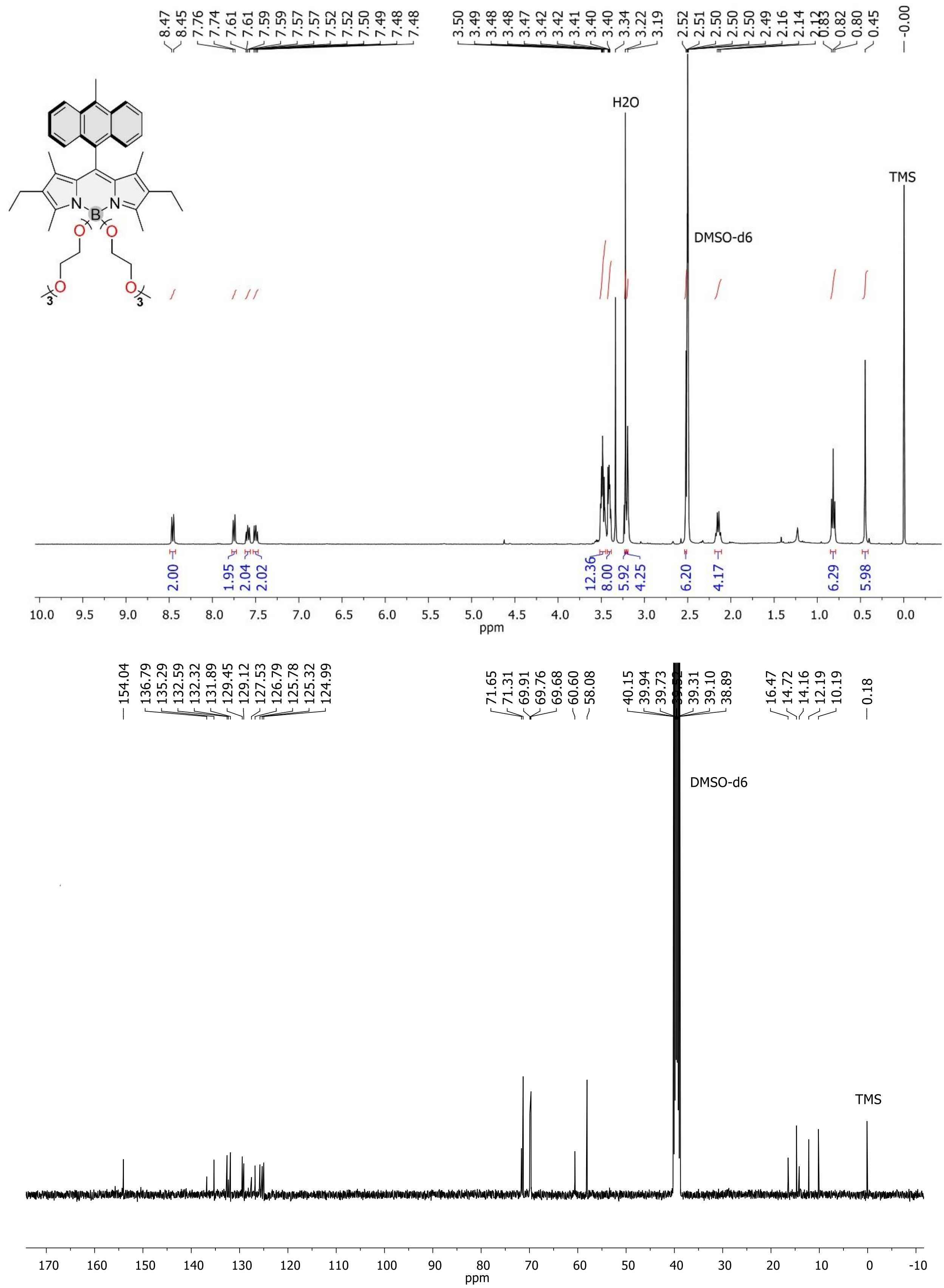

Figure S36. ^{1}H and ^{13}C NMR spectra of compound **4-TEG**.

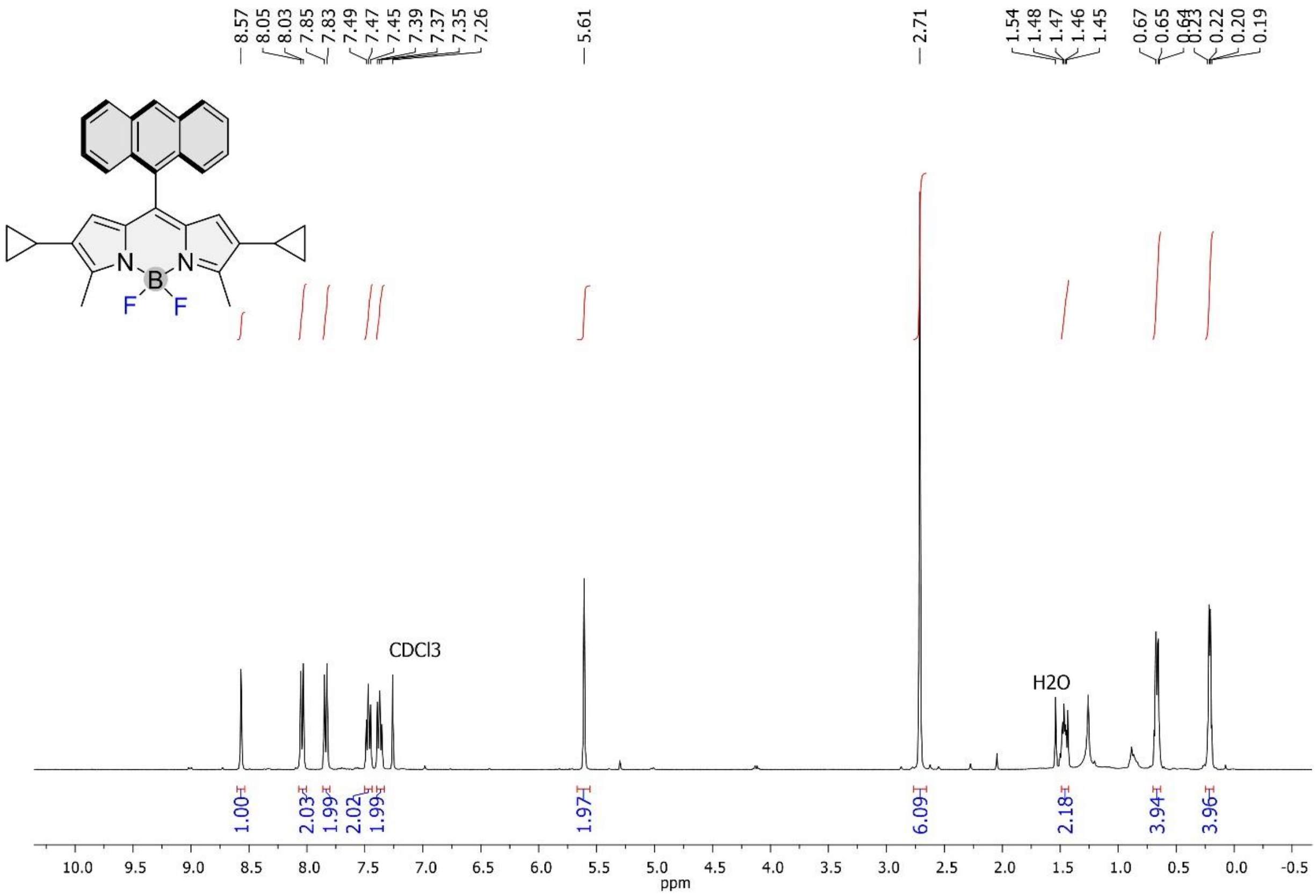

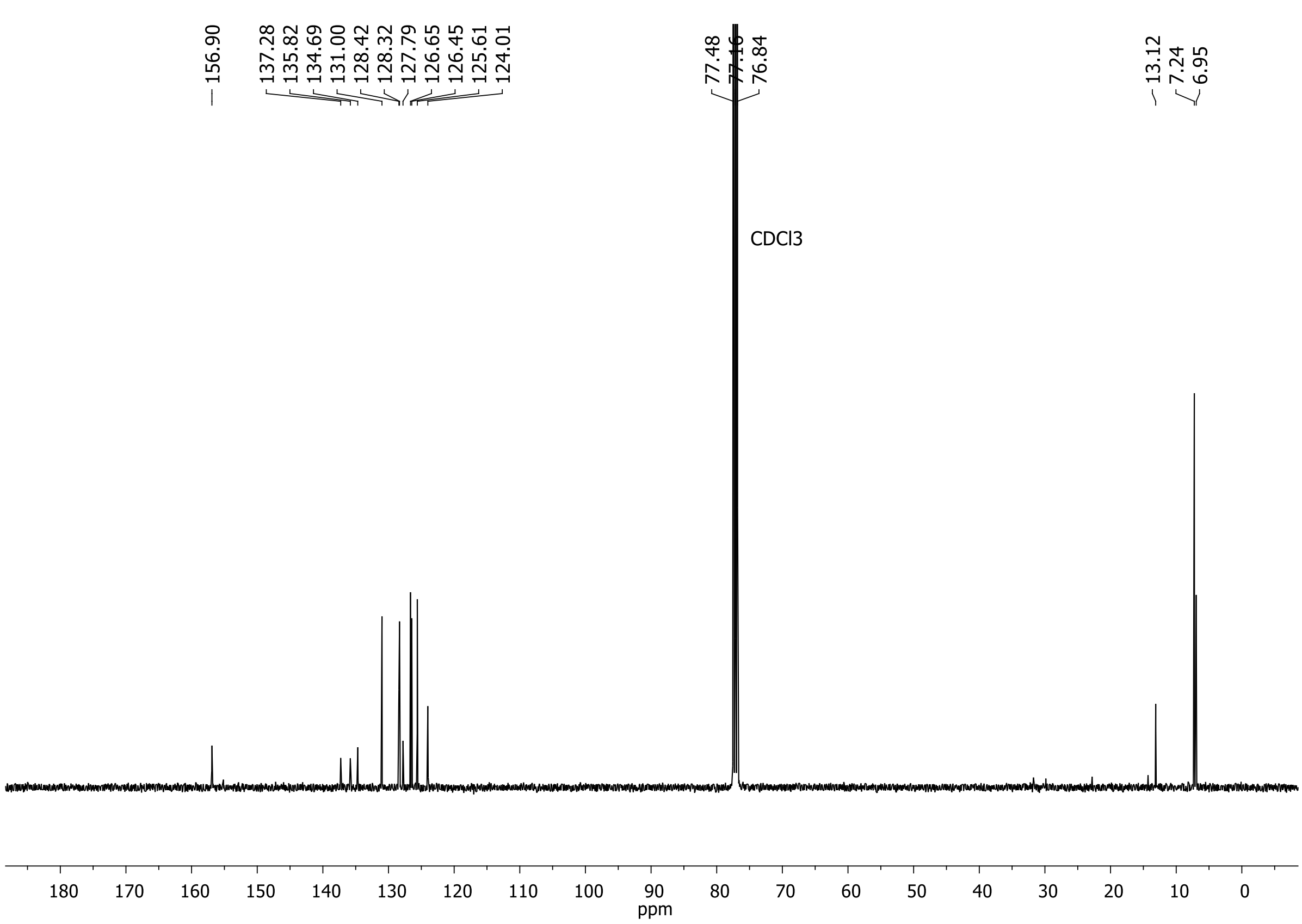

Figure S37. ^{1}H and ^{13}C NMR spectra of compound **5′**.

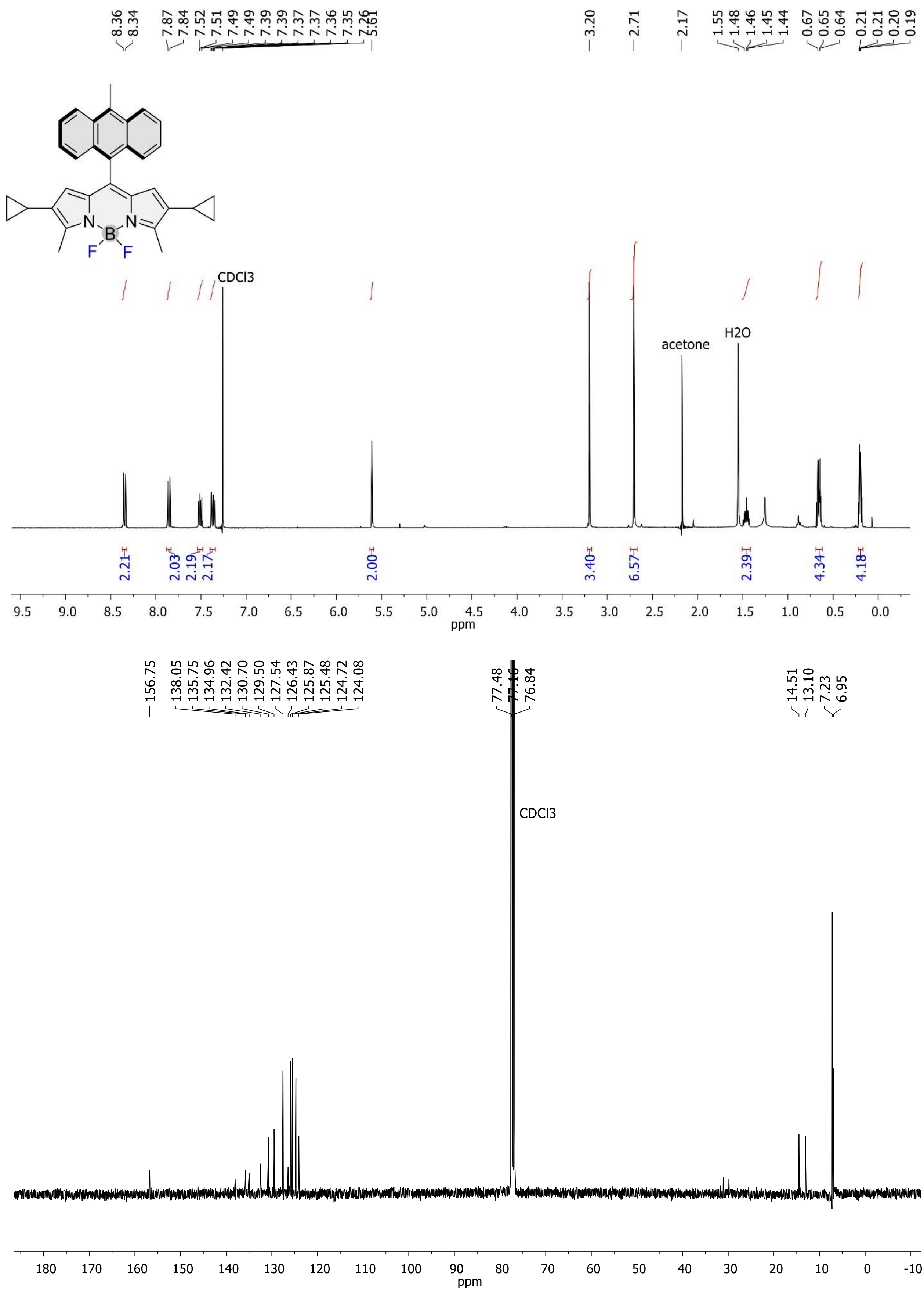

Figure S38. ^{1}H and ^{13}C NMR spectra of compound **5**.

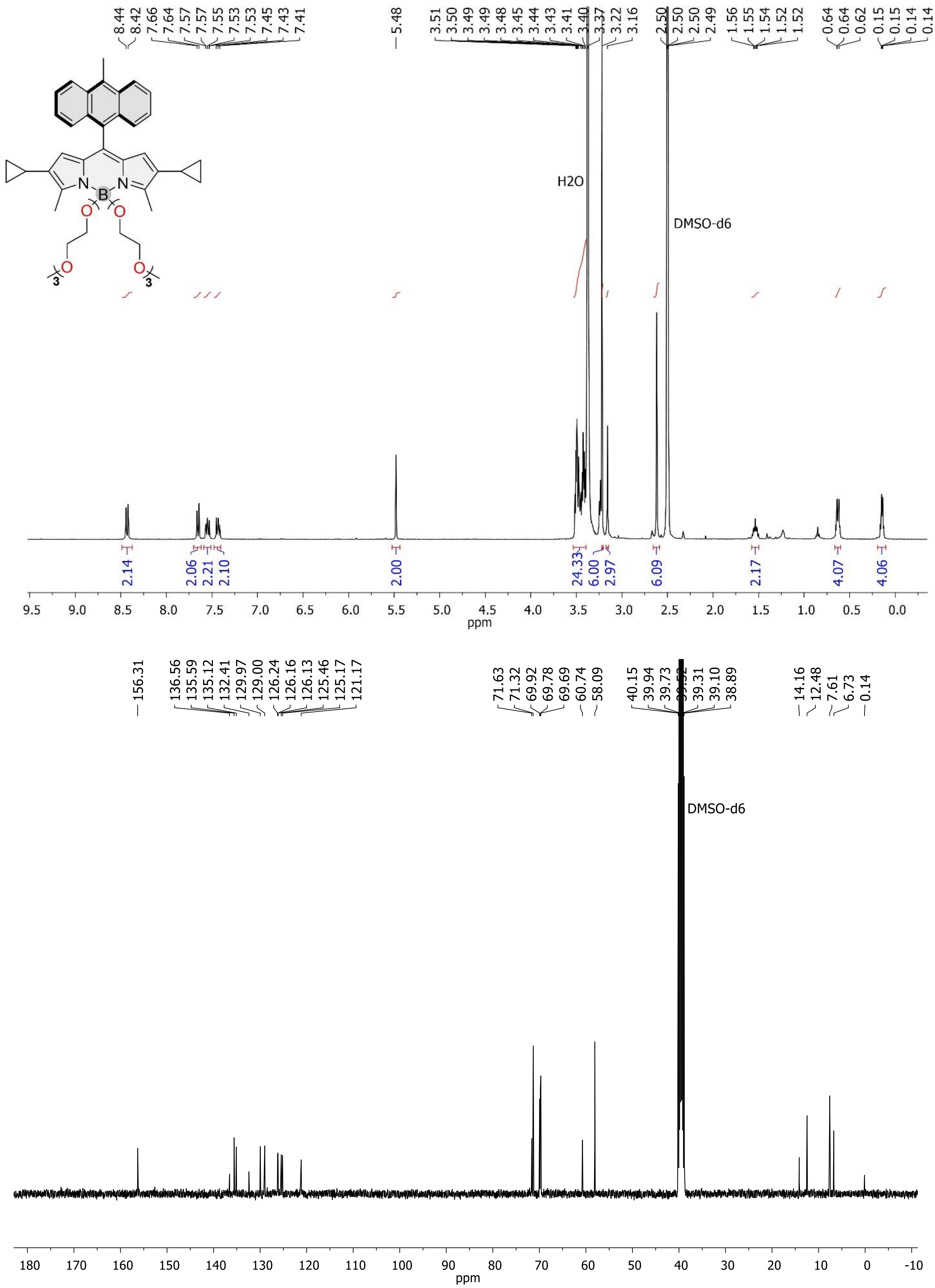

Figure S39. ^{1}H and ^{13}C NMR spectra of compound **5-TEG**.

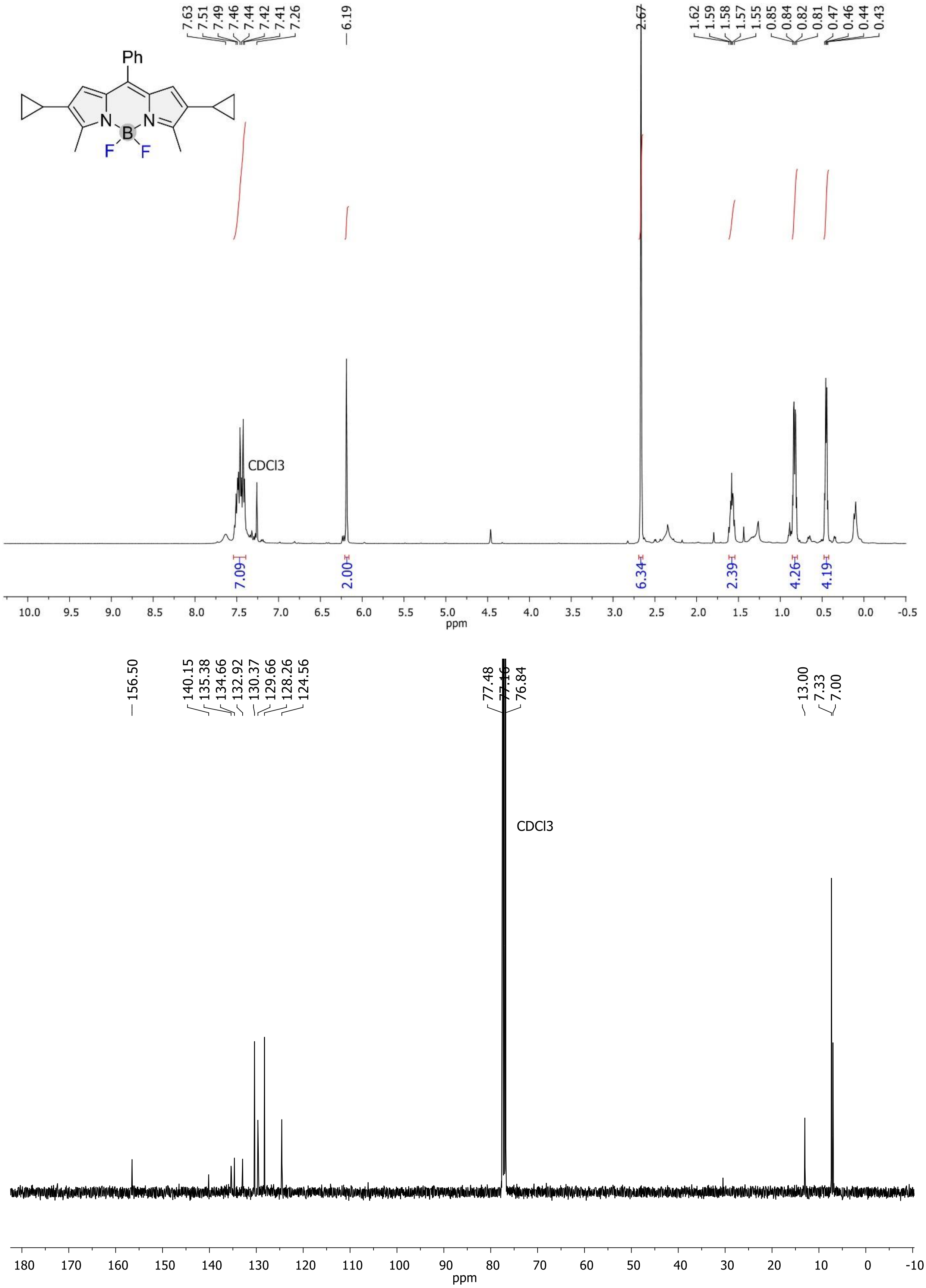

Figure S40. ^{1}H and ^{13}C NMR spectra of compound **6**.

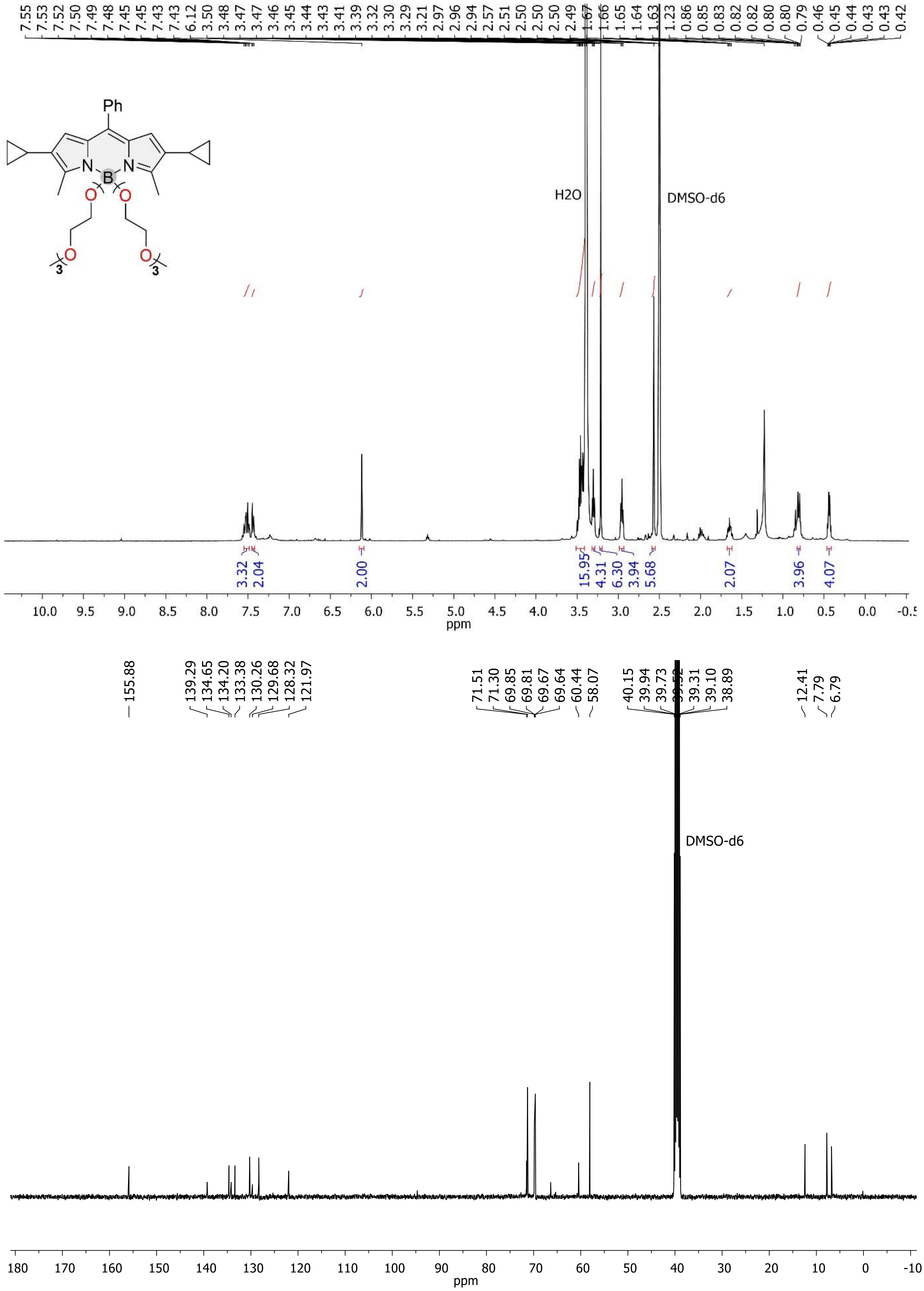

Figure S41. ^{1}H and ^{13}C NMR spectra of compound **6-TEG**.

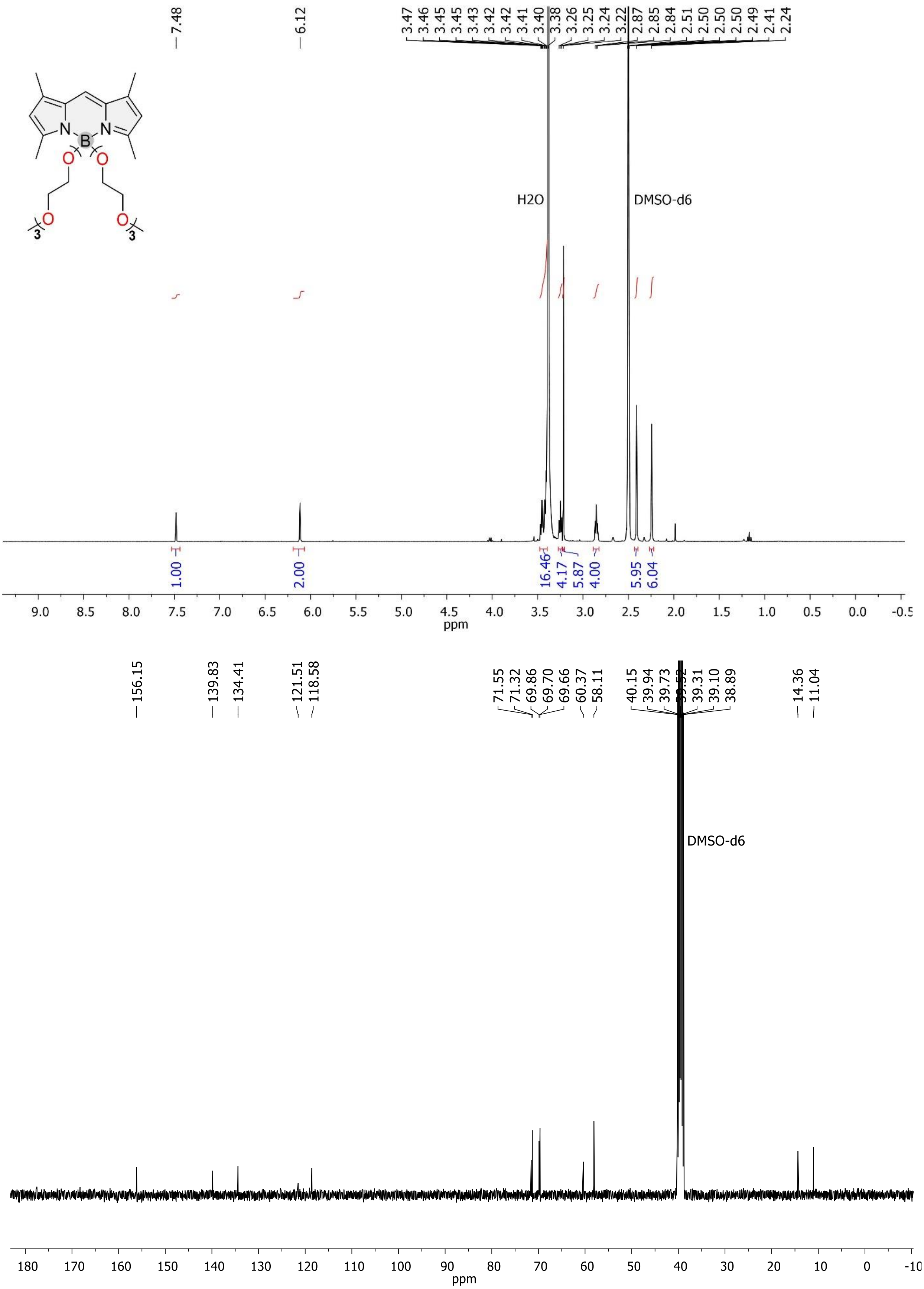

Figure S42. ^{1}H and ^{13}C NMR spectra of compound **7-TEG**.

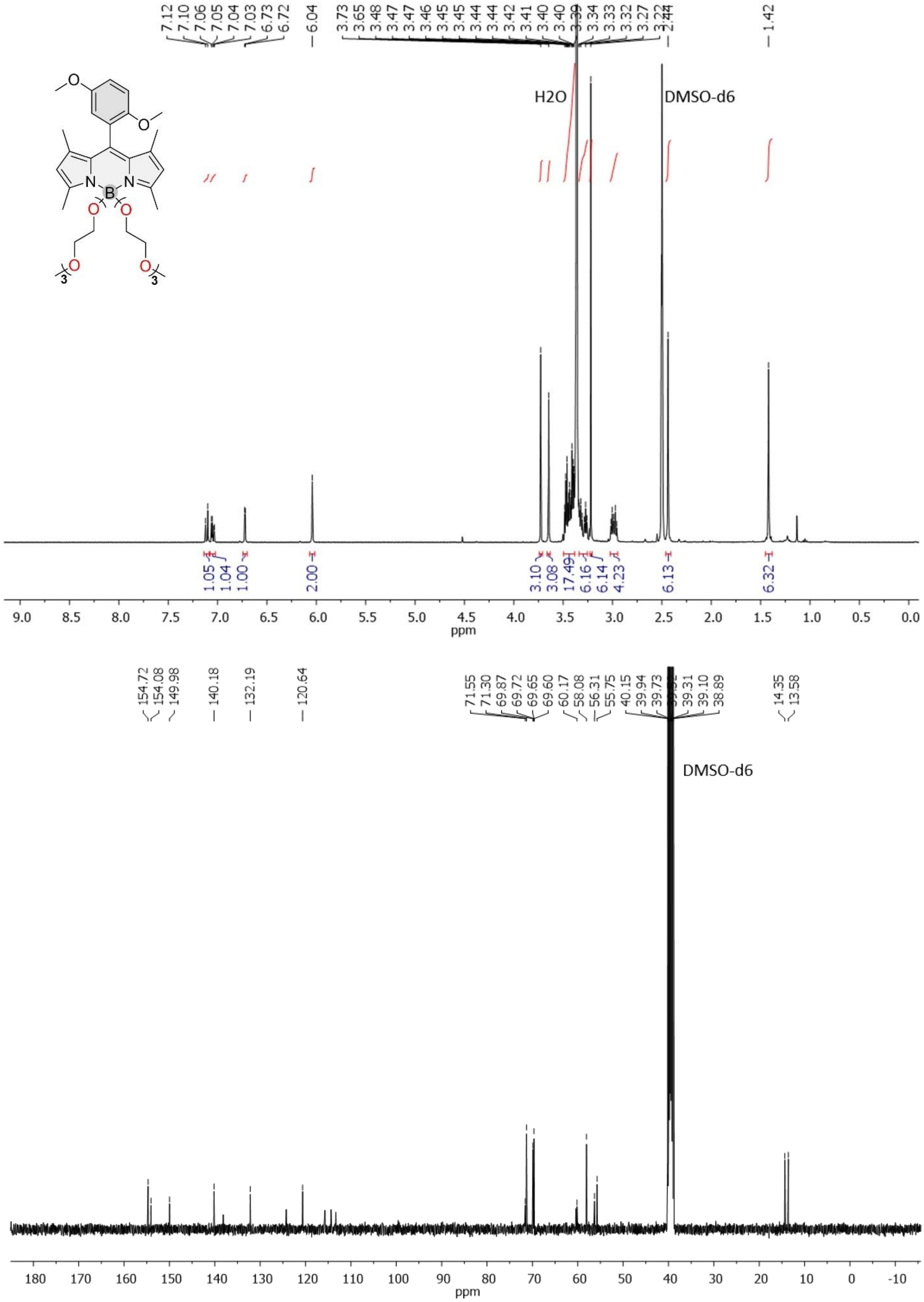

Figure S43. ^{1}H and ^{13}C NMR spectra of compound **8-TEG**.

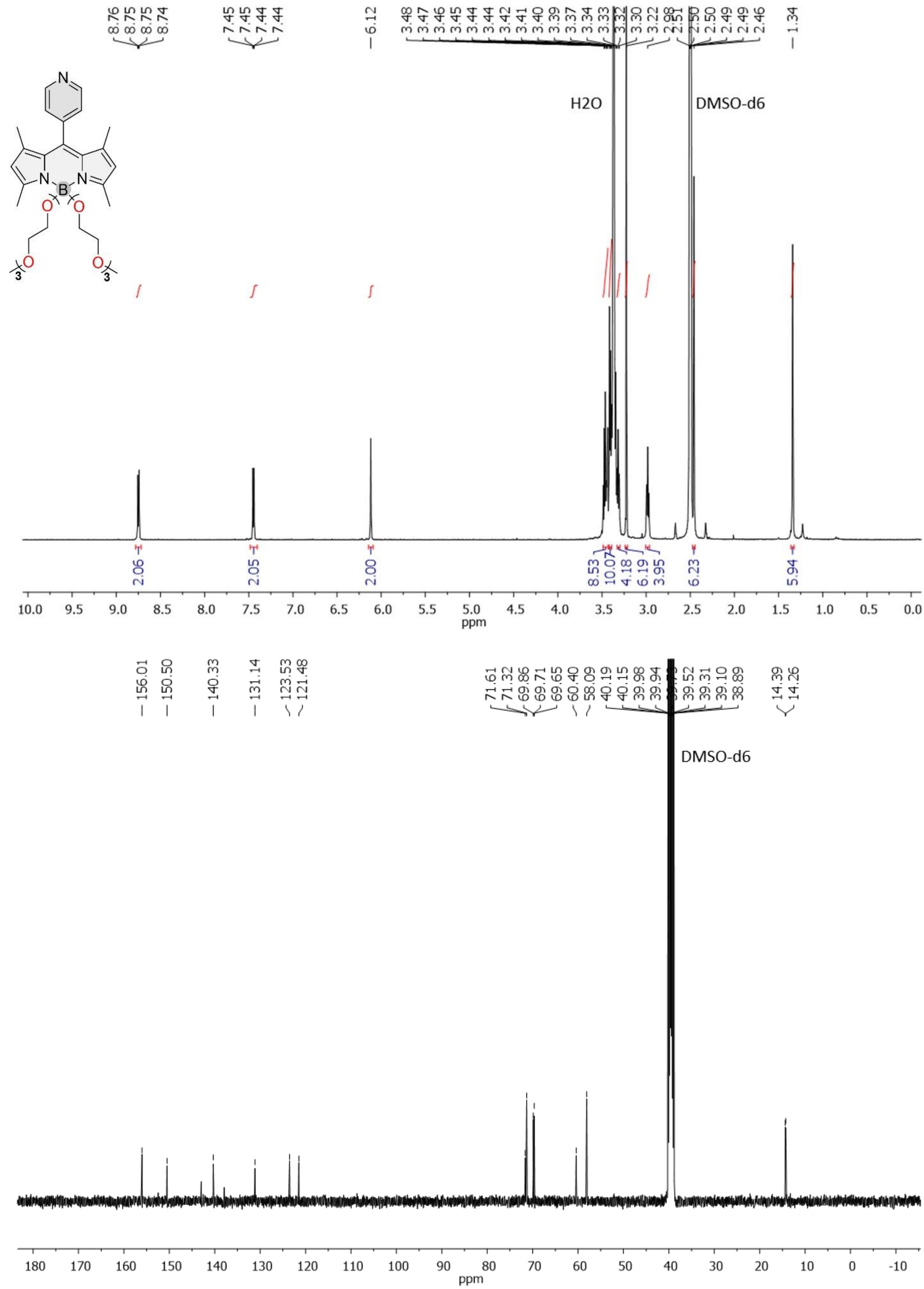

Figure S44. ^{1}H and ^{13}C NMR spectra of compound **9-TEG**.

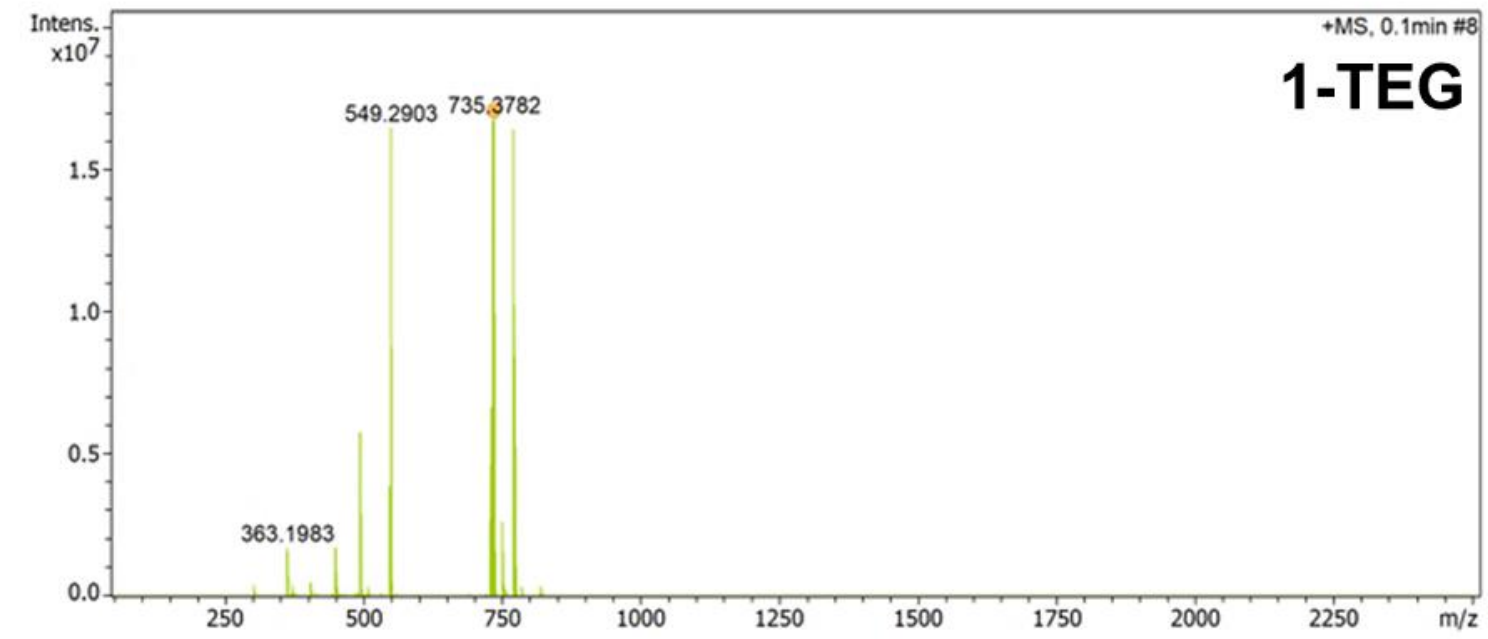

Meas. m/z	#	Ion Formula	m/z	err [ppm]	mSigma	# mSigma	Score	rdb	e⁻ Conf	N-Rule
735.3782	1	C41H53BN2NaO8	735.3787	1.7	43.1	1	100.00	17.0	even	ok

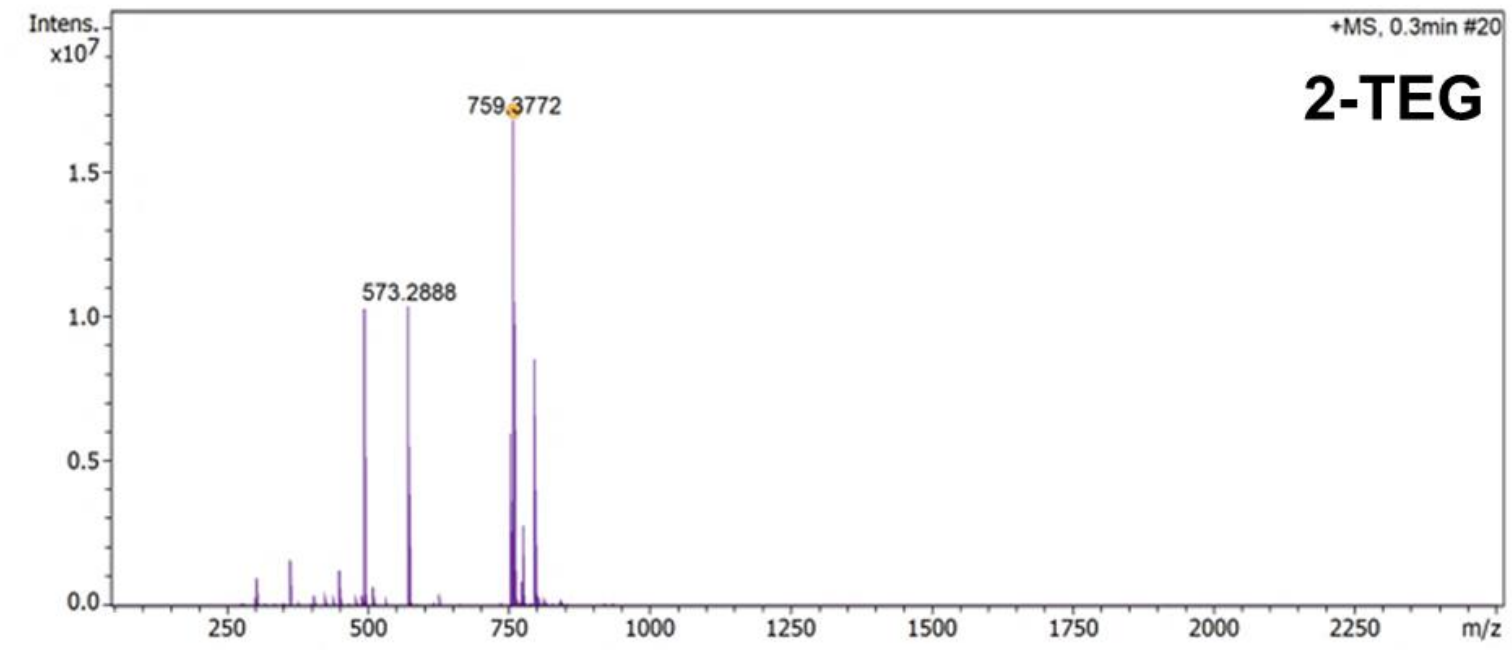

Meas. m/z	#	Ion Formula	m/z	err [ppm]	mSigma	# mSigma	Score	rdb	e⁻ Conf	N-Rule
759.3772	1	C43H53BN2NaO8	759.3787	3.0	56.8	1	100.00	19.0	even	ok

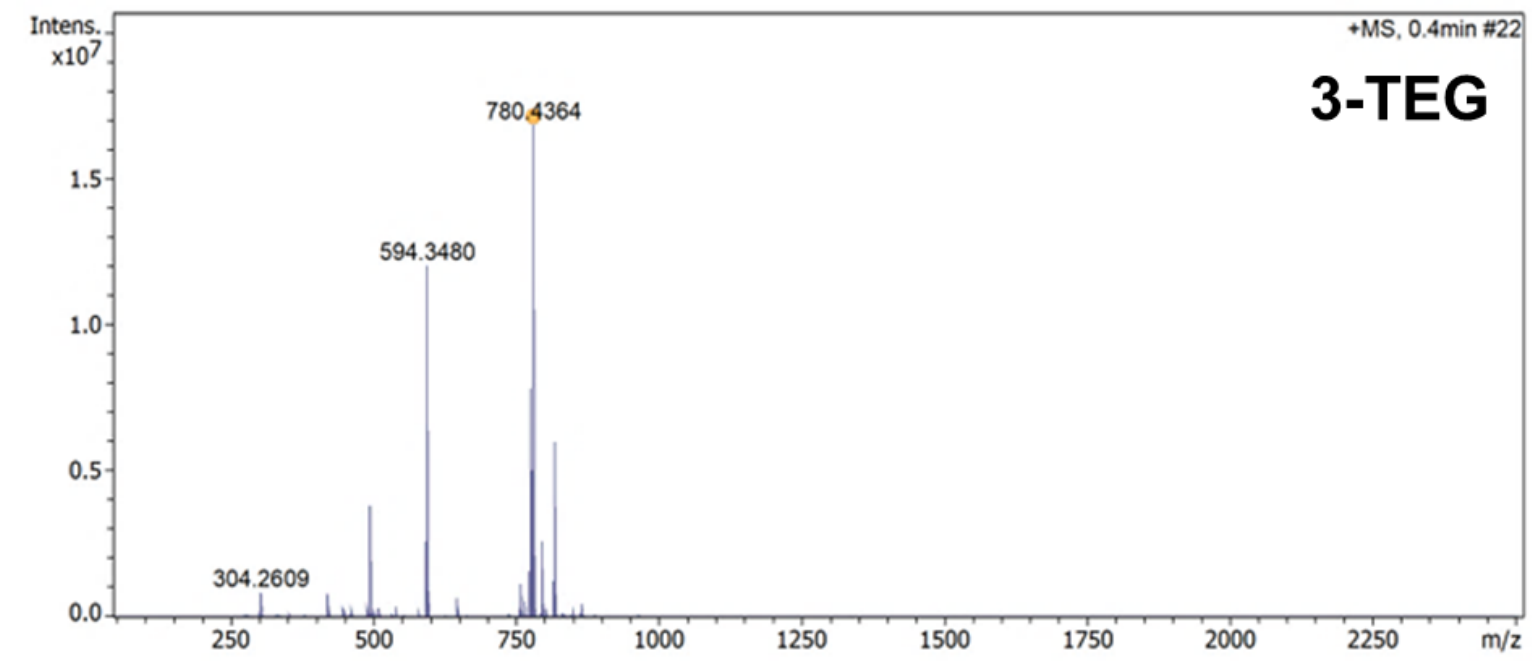

Meas. m/z	#	Ion Formula	m/z	err [ppm]	mSigma	# mSigma	Score	rdb	e⁻ Conf	N-Rule
780.4364	1	C43H60BN3NaO8	780.4366	1.2	89.1	1	100.00	16.0	even	ok

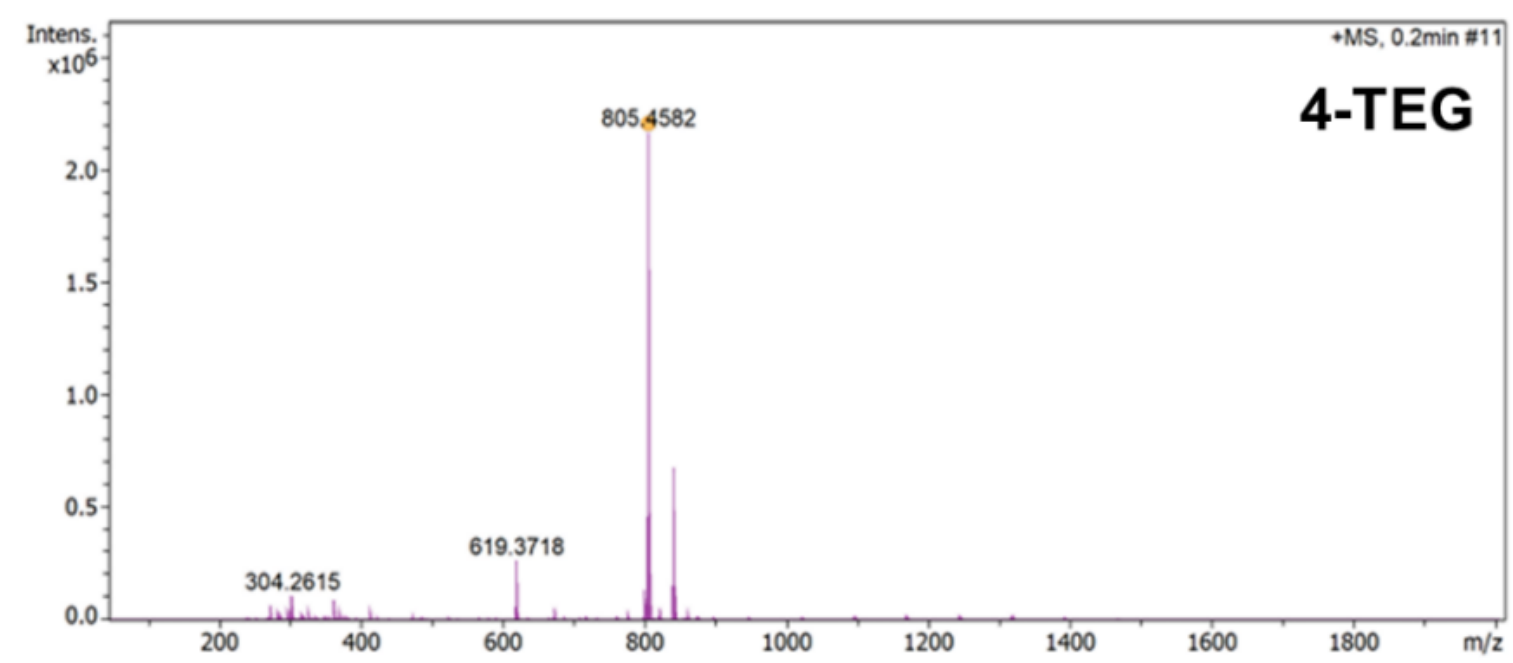

Meas. m/z	#	Ion Formula	m/z	err [ppm]	mSigma	# mSigma	Score	rdb	e⁻ Conf	N-Rule
805.4582	1	C46H63BN2NaO8	805.4570	-0.6	5.3	1	100.00	17.0	even	ok

Figure S45. High-resolution mass spectra of compounds **1-TEG**, **2-TEG**, **3-TEG** and **4-TEG**.

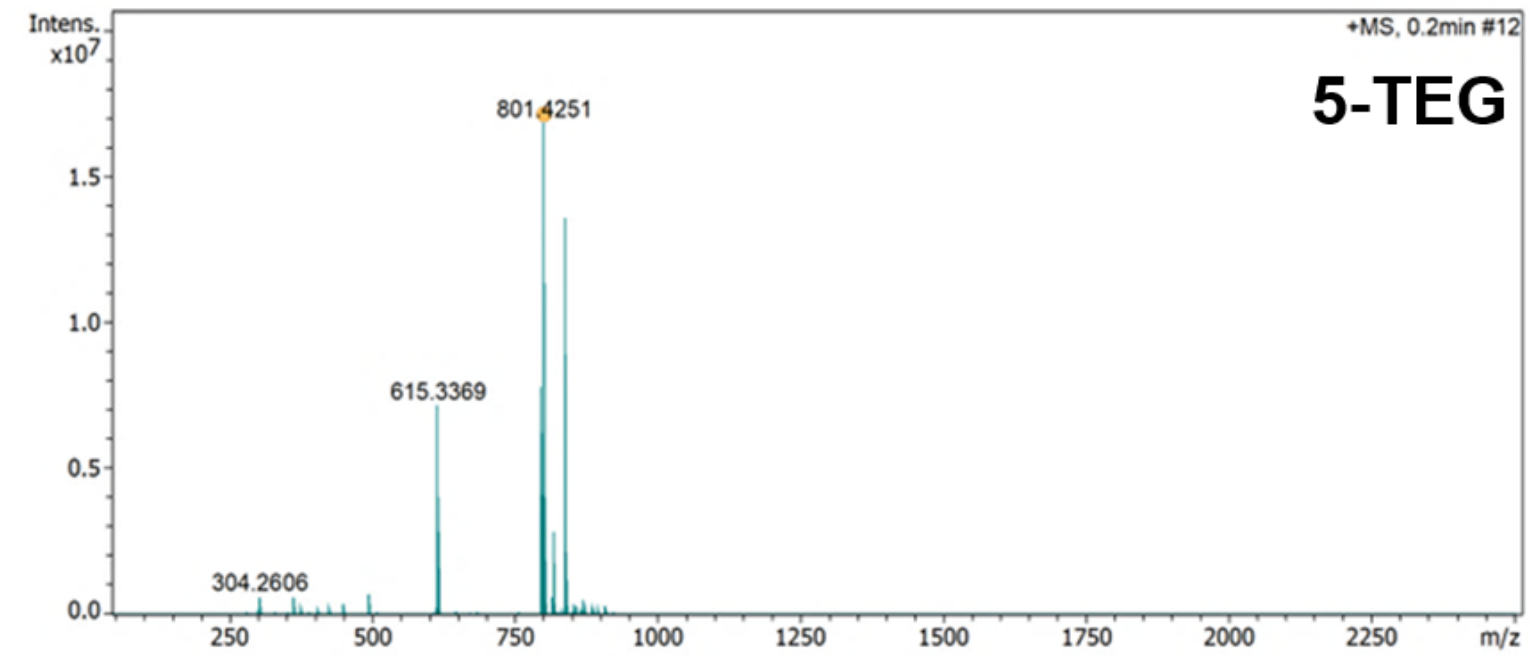

Meas. m/z	#	Ion Formula	m/z	err [ppm]	mSigma	# mSigma	Score	rdb	e⁻ Conf	N-Rule
801.4251	1	C46H59BN2NaO8	801.4257	1.7	87.1	1	100.00	19.0	even	ok

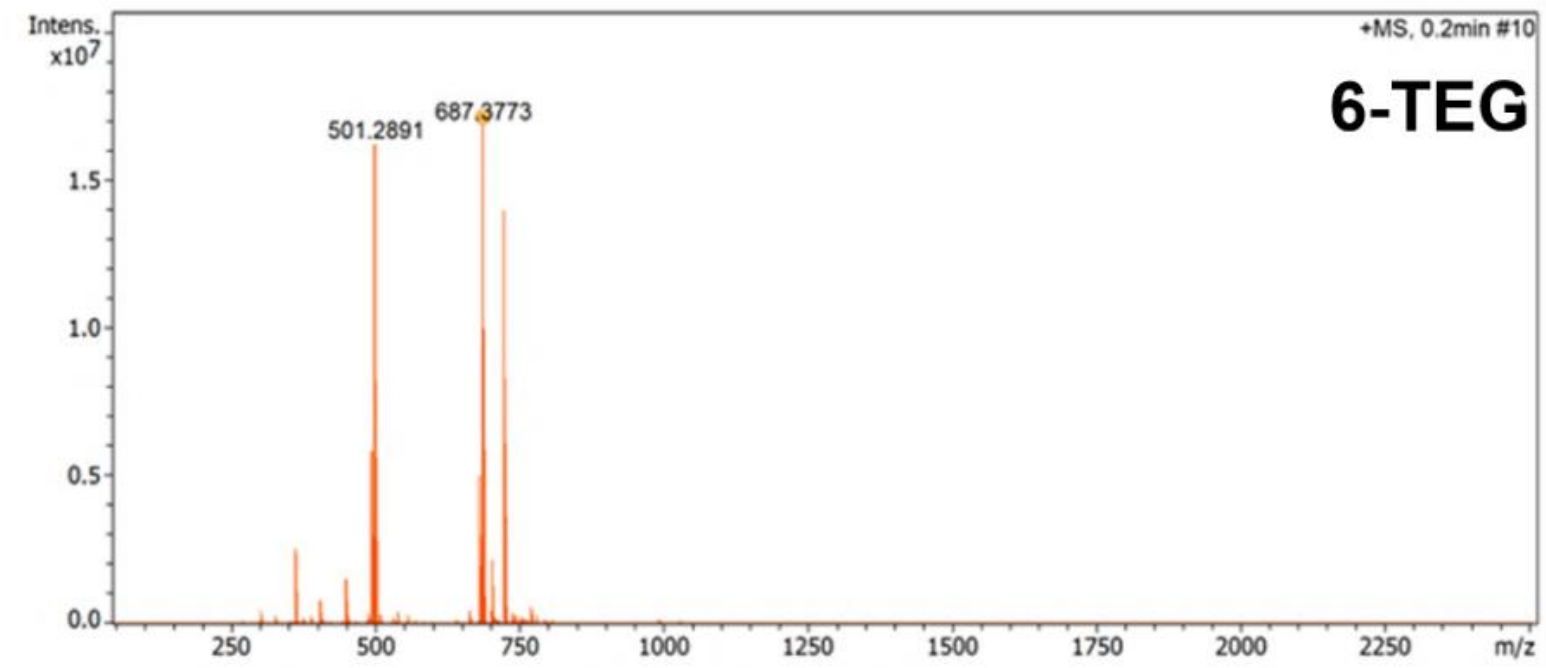

Meas. m/z	#	Ion Formula	m/z	err [ppm]	mSigma	# mSigma	Score	rdb	e⁻ Conf	N-Rule
687.3773	1	C37H53BN2NaO8	687.3787	3.0	119.9	1	100.00	13.0	even	ok

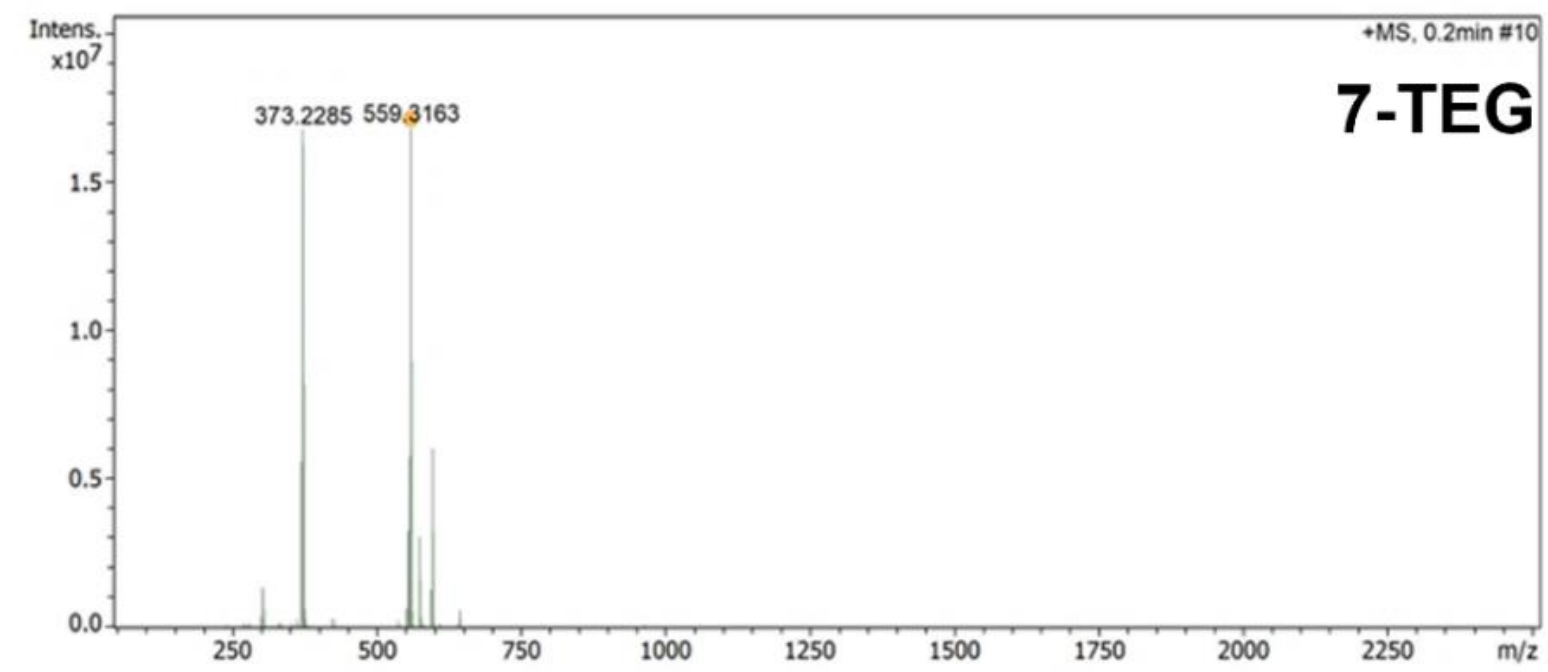

Meas. m/z	#	Ion Formula	m/z	err [ppm]	mSigma	# mSigma	Score	rdb	e⁻ Conf	N-Rule
559.3163	1	C27H45BN2NaO8	559.3161	0.6	86.0	1	100.00	7.0	even	ok

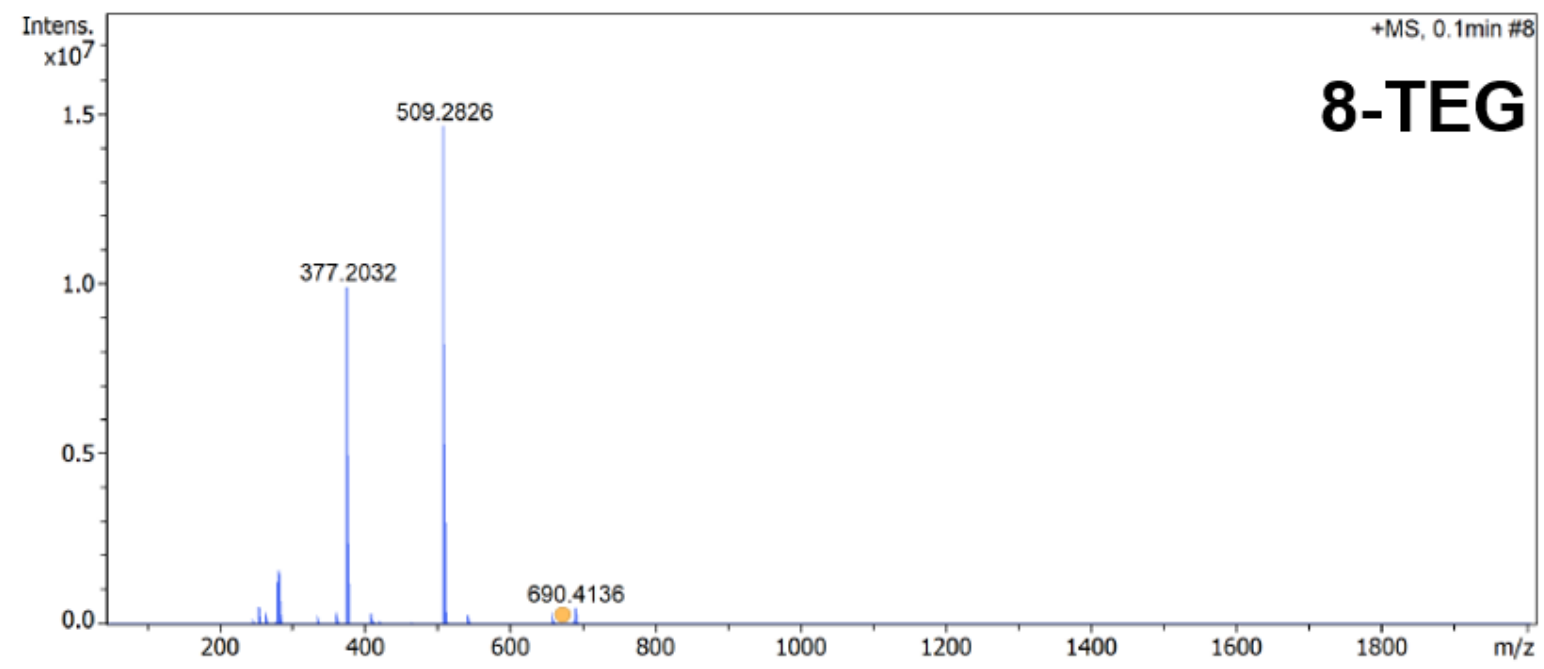

Meas. m/z	#	Ion Formula	m/z	err [ppm]	mSigma	# mSigma	Score	rdb	e⁻ Conf	N-Rule
673.3877	1	C35H54BN2O10	673.3866	-0.7	307.2	1	100.00	11.0	even	ok

Figure S46. High-resolution mass spectra of compounds **5-TEG**, **6-TEG, 7-TEG** and **8-TEG**.

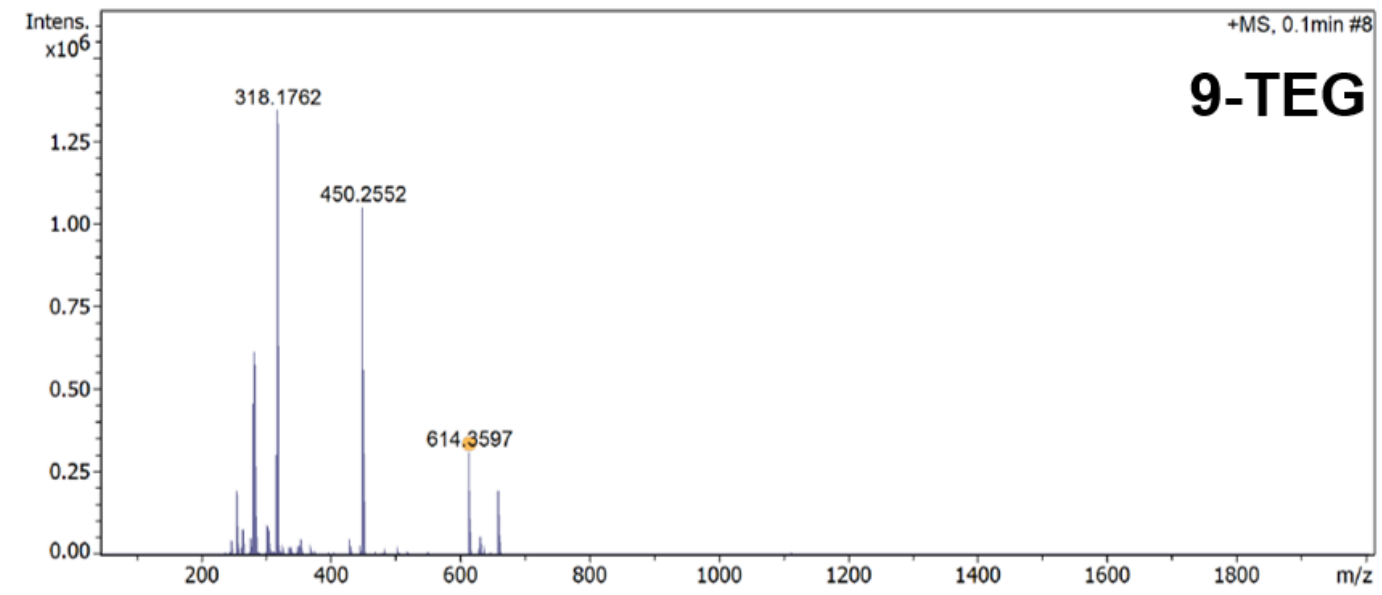

Meas. m/z	#	Ion Formula	m/z	err [ppm]	mSigma	# mSigma	Score	rdb	e⁻ Conf	N-Rule
614.3597	1	C32H49BN3O8	614.3607	2.6	2.1	1	100.00	11.0	even	ok

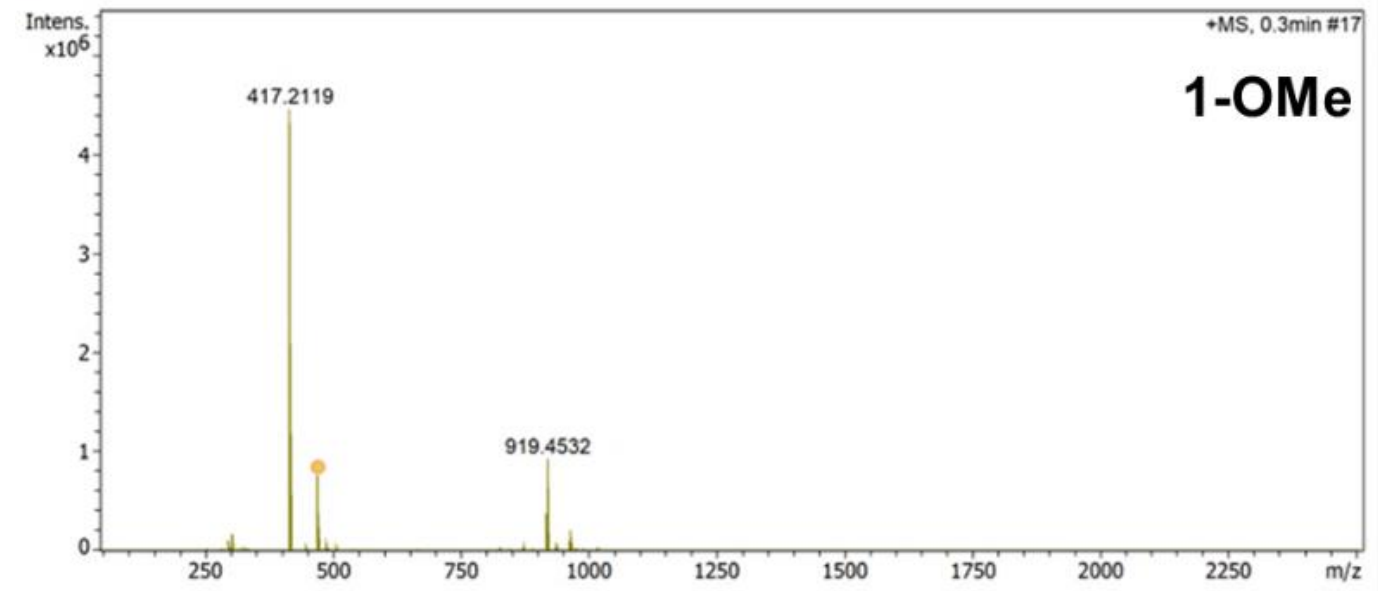

Meas. m/z	#	Ion Formula	m/z	err [ppm]	mSigma	# mSigma	Score	rdb	e⁻ Conf	N-Rule
471.2210	1	C29H29BN2NaO2	471.2214	2.0	4.6	1	100.00	17.0	even	ok

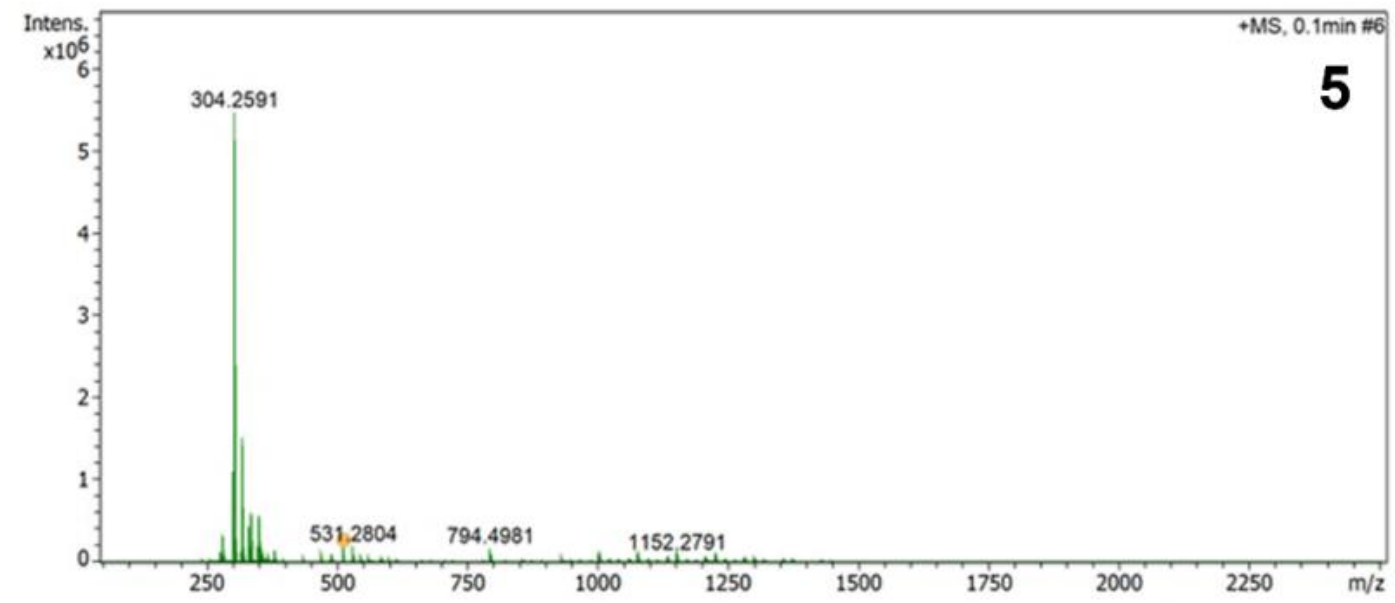

Meas. m/z	#	Ion Formula	m/z	err [ppm]	mSigma	# mSigma	Score	rdb	e⁻ Conf	N-Rule
513.2278	1	C32H29BF2N2Na	513.2284	2.4	17.7	1	100.00	19.0	even	ok

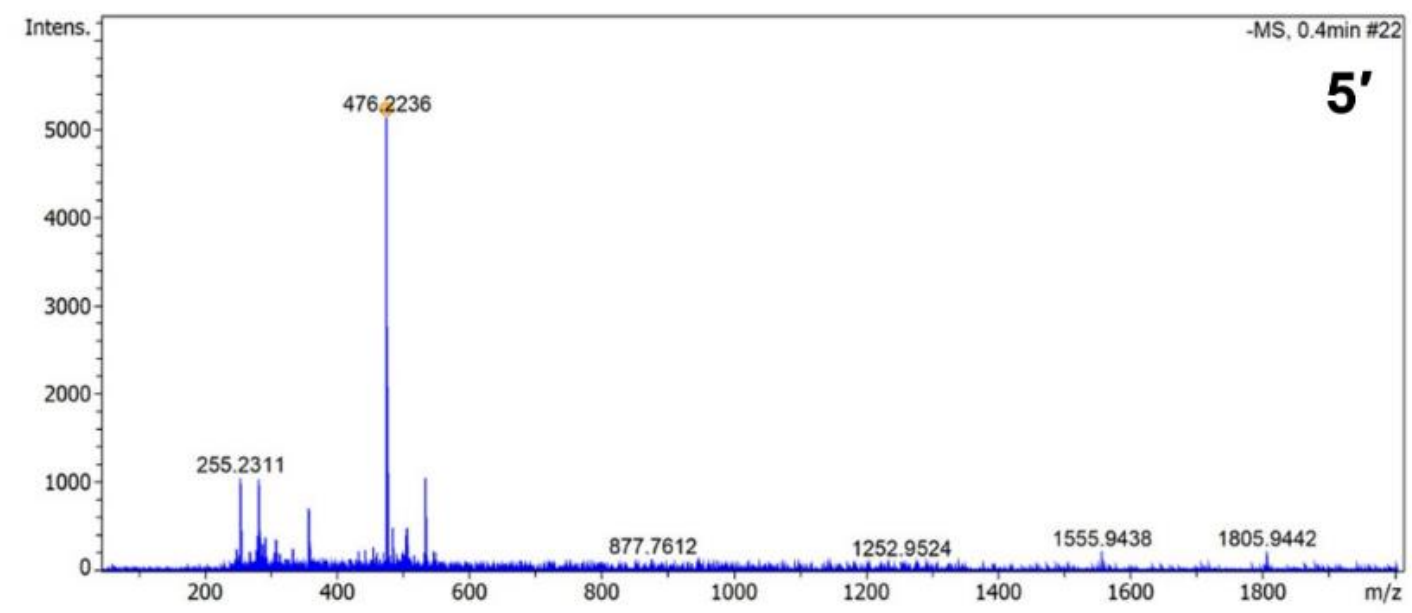

Meas. m/z	#	Ion Formula	m/z	err [ppm]	mSigma	# mSigma	Score	rdb	e⁻ Conf	N-Rule
476.2236	1	C31H27BF2N2	476.2241	2.3	87.8	1	100.00	18.5	odd	ok

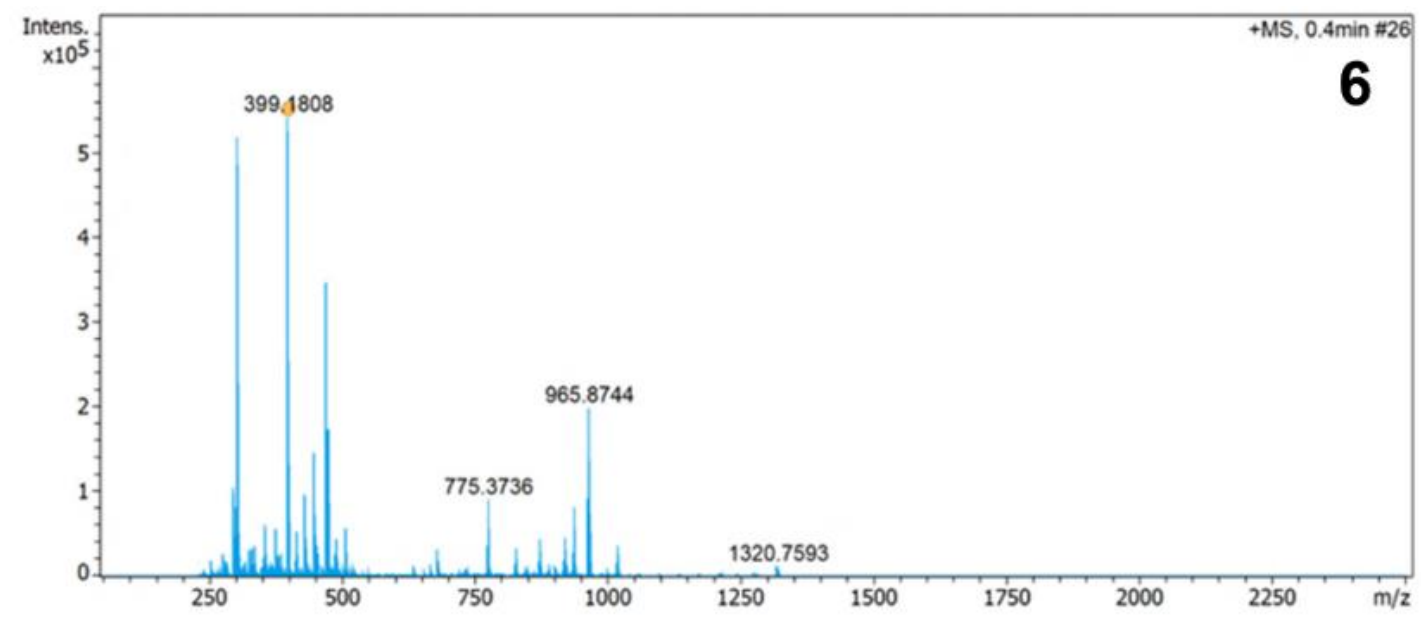

Meas. m/z	#	Ion Formula	m/z	err [ppm]	mSigma	# mSigma	Score	rdb	e⁻ Conf	N-Rule
399.1808	1	C23H23BF2N2Na	399.1815	2.6	6.0	1	100.00	13.0	even	ok

Figure S47. High-resolution mass spectra of compounds **9-TEG**, **1-OMe**, **5**, **5'** and **6**.

7. Computational Data

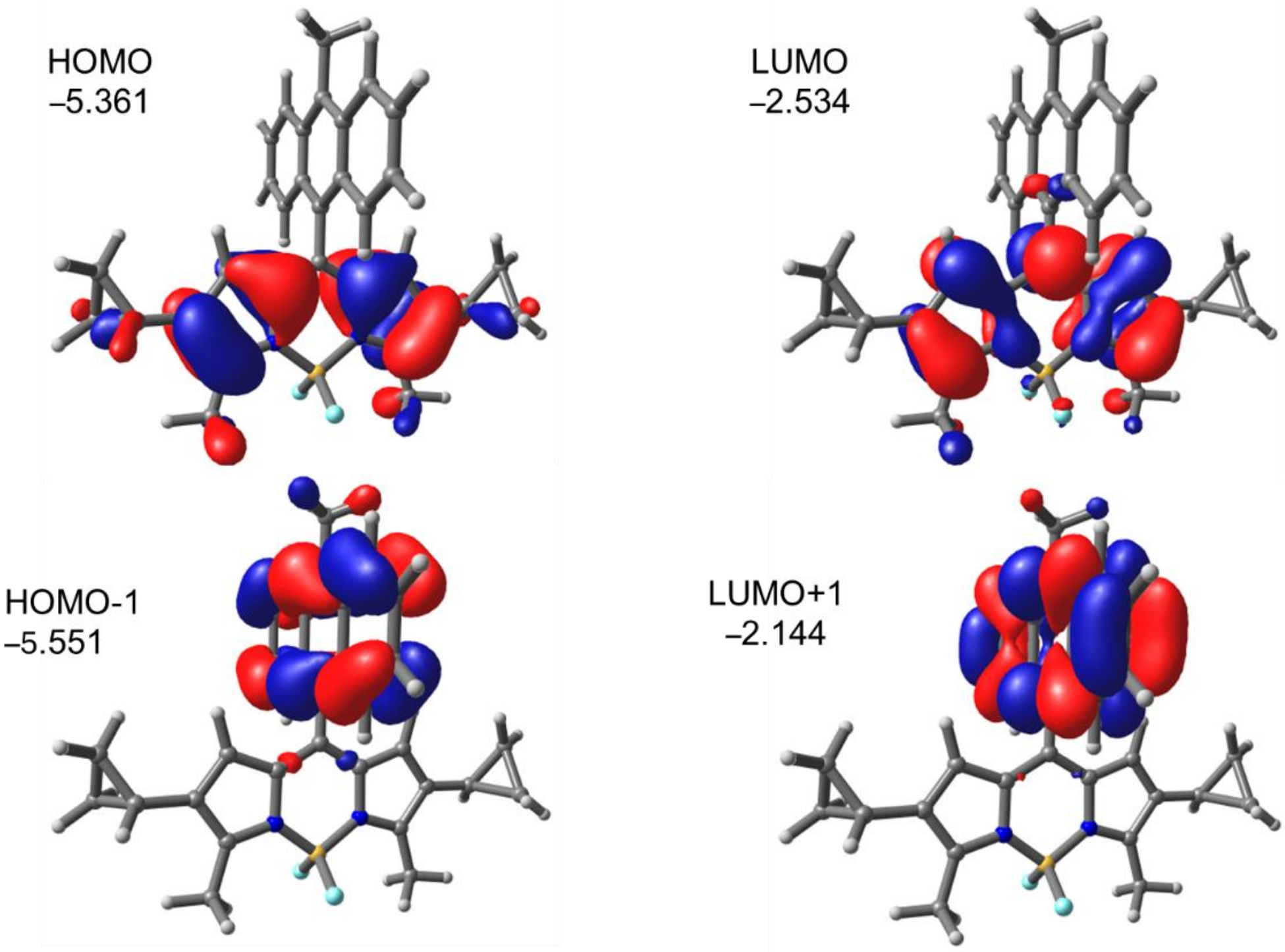

Figure S48. Frontier molecular orbitals of BODIPY **5** in vacuum (B3LYP/6-31+G(d,p)). Orbital energies are given in eV.

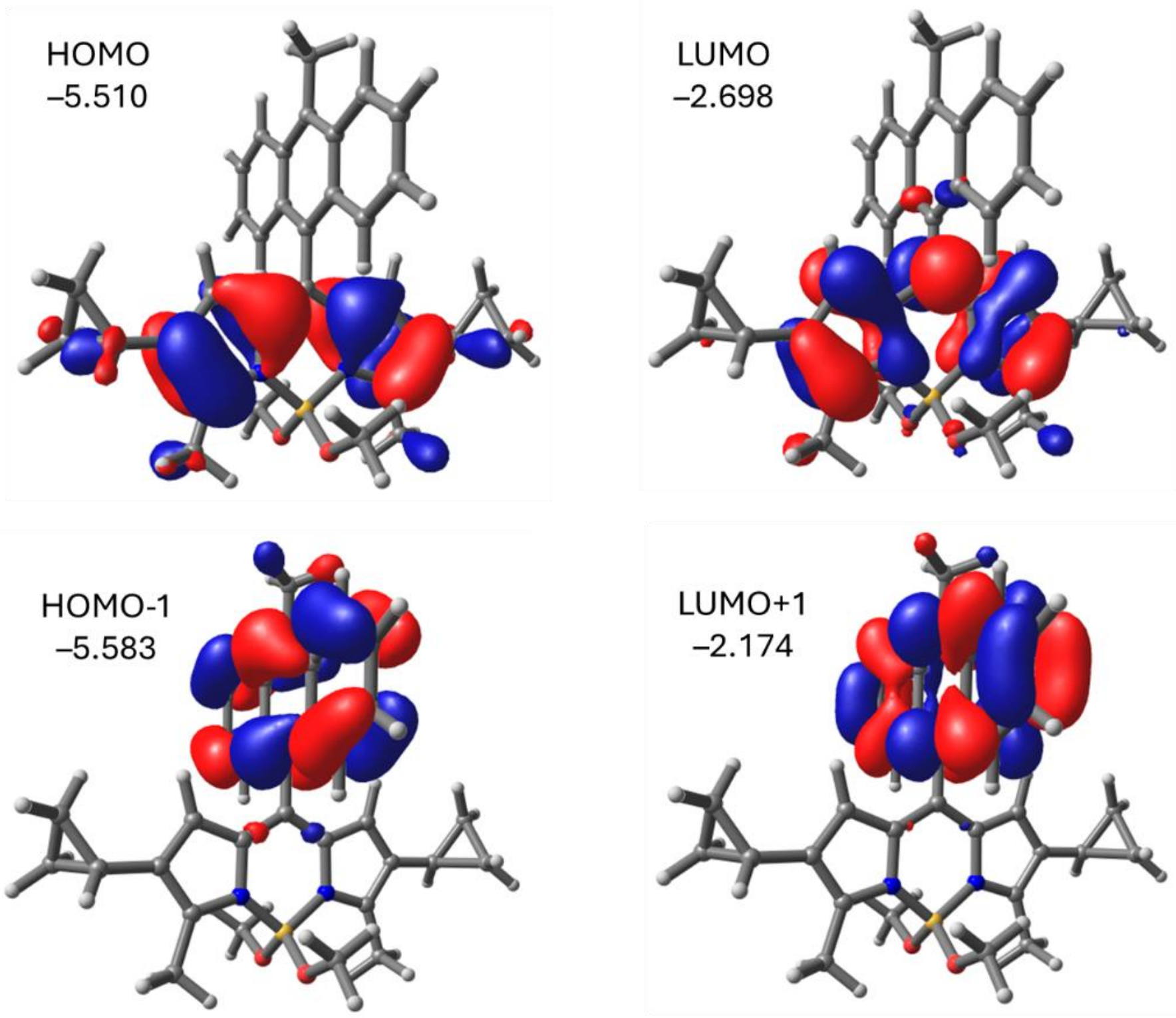

Figure S49. Frontier molecular orbitals of BODIPY **5-OMe** in vacuum (B3LYP/6-31+G(d,p)). Orbital energies are given in eV.

Table S17. Hirshfeld atomic charges for key atoms in dialkoxyboron BODIPY systems calculated at the B3LYP/6-31+G(d,p) level. Charges are given in elementary charge units (e). Charges are reported for boron (B), dipyrrin nitrogens (N, averaged over two symmetry-equivalent atoms), and alkoxy oxygens (O^1, O^2). In hydrated systems (**5-OMe·2H₂O**), the two alkoxy oxygens are non-equivalent due to different hydrogen-bonding geometries.

Compound	Conditions	Q(B)	Q(N)	Q(O^1)	Q(O^2)
**5-OMe**	Vacuum	+0.183	−0.054	−0.233	−0.233
**5-OMe**	PCM-MeCN	+0.179	−0.057	−0.250	−0.250
**5-OMe·2H_2O**	Vacuum	+0.191	−0.058	−0.194	−0.199
**5-OMe·2H_2O**	PCM-MeCN	+0.191	−0.058	−0.202	−0.201

Molecular docking

Molecular docking of anthracenyl BODIPY **5-OMe** with bovine serum albumin (BSA) was performed using AutoDock 4.2.[32] The DFT-optimized geometry of **5-OMe** was used for docking, with DFT-derived Hirshfeld atomic charges assigned in place of the automatically generated Gasteiger charges. The crystal structure of BSA was obtained from the Protein Data Bank (PDB ID: 4F5S).

A two-stage docking strategy comprising initial blind docking followed by focused docking was employed to identify potential binding sites of **5-OMe** within BSA. Docking conformations were generated using the Lamarckian genetic algorithm and ranked according to their predicted binding free energies. UCSF ChimeraX 1.12[33] was used for structural analysis, visualization, and preparation of molecular graphics.

Docking analysis

Docking analysis revealed two principal and spatially distinct binding regions for **5-OMe**, together with several less populated secondary sites (Figure S50).

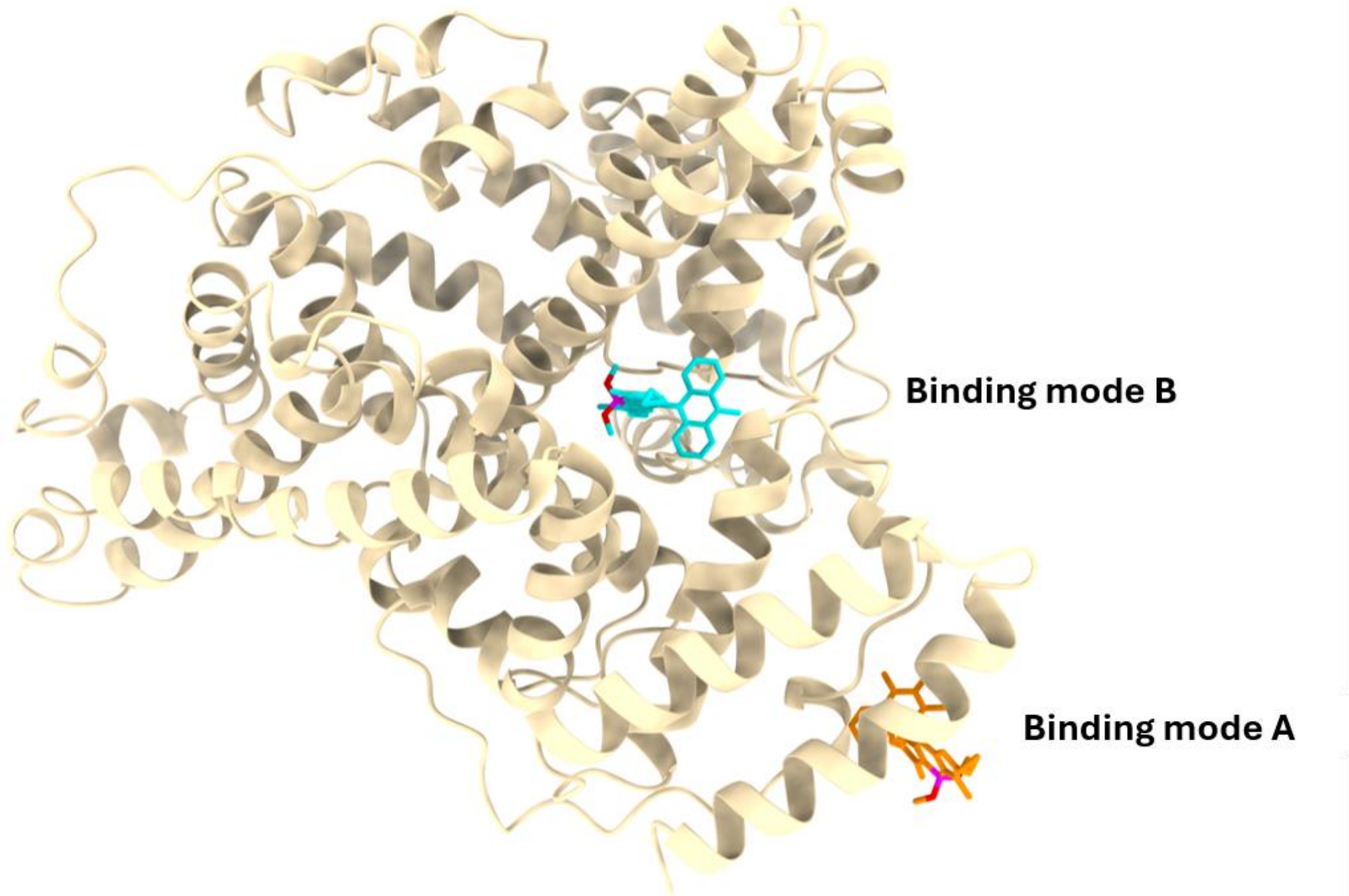

Figure S50. Principal binding modes of **5-OMe** predicted by molecular docking with BSA. Binding mode A is shown in orange and binding mode B in cyan.

Binding mode A is located in a predominantly hydrophobic region of domain III, where the anthracene moiety of **5-OMe** is accommodated within a hydrophobic/aromatic environment comprising Phe506, Phe508, Phe553, Phe567, Ala568, Val569, Gly571, and Val575. In this binding mode, the boron-bound oxygen atoms remain relatively distant from the surrounding protein residues (minimum distances of approximately 6–7 Å) and are therefore comparatively exposed to the solvent.

Binding mode B, located within an interdomain binding region, showed greater conformational heterogeneity but included the most favourable docking pose, with a predicted binding energy of −8.29 kcal mol^{-1} (Figure S51). The binding pocket contains both polar and hydrophobic residues, including Arg144, His145, Arg185, Leu189, Ser192, Ala193, Glu424, and Arg458. These residues surround the B–OR region and provide considerable steric enclosure of the boron-bound oxygen

atoms. Importantly, no strong conventional hydrogen bonds involving the B–OR oxygens were identified in this pose. This arrangement may therefore reduce their accessibility to bulk water and provide a locally less hydrated environment.

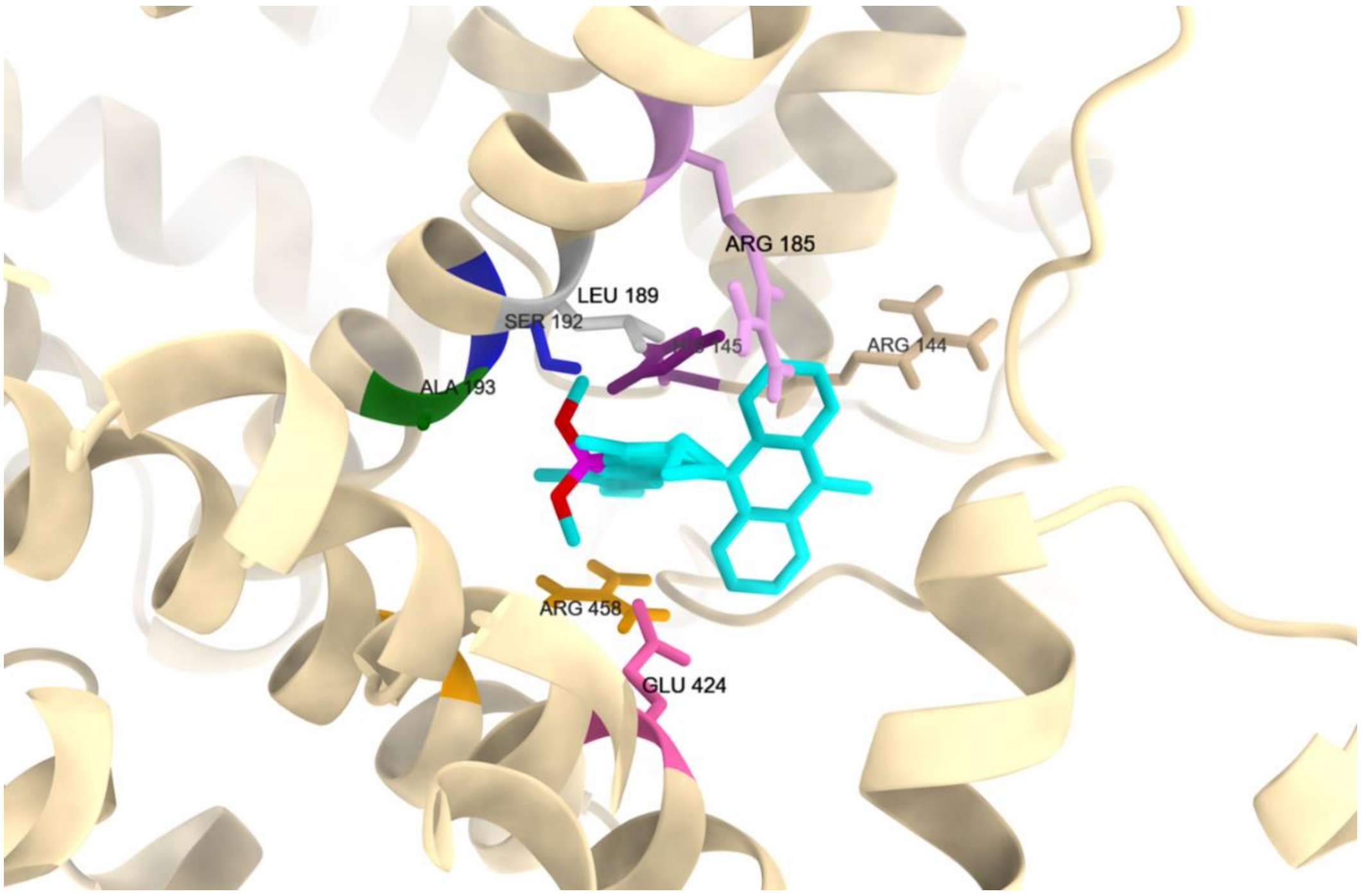

Figure S51. Local binding environment of **5-OMe** in the lowest-energy pose of binding mode B.

Table S18. Optimized geometry (Cartesian coordinates in Å) for compound **5** in vacuum (B3LYP/6-31+G(d,p) level).

Symbol	X	Y	Z	Symbol	X	Y	Z
C	1.45941	3.369197	-0.42174	C	-3.49038	0.455599	3.635235
C	2.611788	2.534928	-0.3082	C	-2.06805	0.458361	3.634892
C	0.354319	2.530765	-0.32211	H	-0.28338	-0.31178	-2.45858
C	0.831274	1.207666	-0.1475	H	-5.24849	-0.34207	-2.53912
N	2.227735	1.250482	-0.14535	H	-6.1139	-0.82037	0.675628
C	0.136602	0.000138	-0.00356	H	-6.19765	-0.13385	-0.9387
C	0.83779	-1.20308	0.144902	H	-6.11821	0.929878	0.456214
N	2.234477	-1.23733	0.151414	H	-5.26495	0.304244	2.485405
C	2.625377	-2.51941	0.316624	H	-0.29618	0.311606	2.464581
C	1.477457	-3.36075	0.422861	H	-3.99382	-0.60179	-4.59823
C	0.367886	-2.5291	0.316401	H	-1.48991	-0.58781	-4.57518
C	-1.36153	-0.00421	-0.00573	H	-4.02963	0.573725	4.570626
C	-2.06297	-0.16157	-1.21876	H	-1.52714	0.577939	4.568949
C	-3.51402	-0.16653	-1.22278	C	0.341598	-5.70316	0.241071
C	-4.22523	-0.0125	-0.00874	C	1.619571	-5.78646	-0.5562
C	-3.50913	0.14473	1.203034	C	0.307696	5.704477	-0.24838
C	-2.06391	0.149232	1.208611	C	1.579387	5.795824	0.558002
B	3.162007	0.009508	0.006484	H	2.03424	5.165509	-1.51003
F	3.971007	-0.11728	-1.13225	H	2.055688	-5.1536	1.515065
F	3.961599	0.141412	1.151317	H	-0.51357	-5.22698	-0.22893
C	1.488354	4.841696	-0.62472	H	0.085679	-6.53023	0.896357
C	4.044233	2.951535	-0.37236	H	1.62408	-5.3481	-1.54981
H	-0.68827	2.811136	-0.37466	H	2.24377	-6.66871	-0.44676
C	4.059923	-2.92721	0.389803	H	-0.54783	5.222991	0.215525
C	1.514138	-4.83308	0.6259	H	0.051301	6.529869	-0.9056
H	-0.67328	-2.8159	0.362092	H	1.579548	5.357569	1.551664
C	-1.36758	-0.31774	-2.4638	H	2.198829	6.681946	0.452905
C	-4.16718	-0.33197	-2.4918	H	4.635523	2.443633	0.393871
C	-5.73853	-0.01027	0.03961	H	4.128976	4.031716	-0.23572
C	-4.18194	0.303844	2.460085	H	4.481499	2.687973	-1.34308
C	-1.3805	0.309673	2.458451	H	4.489741	-2.66006	1.36286
C	-3.46309	-0.47919	-3.65858	H	4.652682	-2.41639	-0.37336
C	-2.0405	-0.47178	-3.64638	H	4.152077	-4.00697	0.254688

Table S19. Optimized geometry (Cartesian coordinates in Å) for compound **5** with PCM solvation in MeCN (B3LYP/6-31+G(d,p) level).

Symbol	X	Y	Z	Symbol	X	Y	Z
C	1.500752	3.366303	-0.39068	C	-3.46859	0.455664	3.638468
C	2.649691	2.527639	-0.28652	C	-2.04538	0.451655	3.63884
C	0.391913	2.529327	-0.30176	H	-0.26276	-0.29377	-2.46519
C	0.861743	1.203162	-0.14273	H	-5.22928	-0.29285	-2.54081
N	2.260093	1.237718	-0.14411	H	-6.09251	-0.79132	0.681491
C	0.158997	-0.00015	-0.00346	H	-6.17746	-0.11625	-0.93741
C	0.853202	-1.20777	0.140596	H	-6.09489	0.956216	0.451349
N	2.251334	-1.25133	0.150645	H	-5.24283	0.318616	2.485472
C	2.631633	-2.54358	0.296059	H	-0.27288	0.303568	2.470442
C	1.476653	-3.37486	0.393391	H	-3.977	-0.54419	-4.60381
C	0.373868	-2.53092	0.297249	H	-1.47304	-0.54684	-4.58177
C	-1.33903	0.004449	-0.00588	H	-4.00753	0.572522	4.573951
C	-2.04088	-0.14075	-1.22033	H	-1.50505	0.564949	4.573967
C	-3.49232	-0.13883	-1.22409	C	0.256354	-5.68594	0.425255
C	-4.20464	0.008429	-0.00894	C	1.321213	-5.77719	-0.63927
C	-3.48623	0.156144	1.203696	C	0.294362	5.684593	-0.43
C	-2.0406	0.154033	1.209578	C	1.352237	5.769128	0.642016
B	3.172662	-0.0097	0.005437	H	2.267952	5.193414	-1.2831
F	3.998047	-0.15334	-1.13235	H	2.226946	-5.20591	1.291889
F	3.993658	0.128543	1.147007	H	-0.67139	-5.18343	0.168863
C	1.541607	4.841911	-0.553	H	0.136831	-6.52156	1.108184
C	4.080381	2.955688	-0.29046	H	1.116627	-5.3117	-1.59907
H	-0.65014	2.81247	-0.34986	H	1.935003	-6.67199	-0.68677
C	4.059087	-2.98203	0.30926	H	-0.63812	5.187493	-0.18036
C	1.507626	-4.85055	0.556675	H	0.184568	6.52117	-1.1134
H	-0.67025	-2.80738	0.338949	H	1.138166	5.304408	1.600128
C	-1.34719	-0.29231	-2.46754	H	1.970944	6.660268	0.694286
C	-4.14809	-0.29022	-2.49427	H	4.428278	3.143065	0.733393
C	-5.71755	0.013463	0.039333	H	4.198703	3.886054	-0.85069
C	-4.15999	0.312442	2.46131	H	4.728288	2.194163	-0.72746
C	-1.35737	0.305313	2.461355	H	4.713355	-2.21557	0.727245
C	-3.44546	-0.43235	-3.66352	H	4.403975	-3.19761	-0.7101
C	-2.022	-0.4338	-3.65169	H	4.170798	-3.89977	0.891537

Table S20. Optimized geometry (Cartesian coordinates in Å) for compound **5-OMe** in vacuum (B3LYP/6-31+G(d,p) level).

Symbol	X	Y	Z	Symbol	X	Y	Z
C	1.257654	3.368262	-0.44728	H	-6.30242	-0.80765	0.677764
C	2.416299	2.538251	-0.33132	H	-6.38444	-0.12731	-0.93926
C	0.158259	2.525622	-0.34141	H	-6.30357	0.941741	0.451428
C	0.646171	1.205978	-0.15984	H	-5.45125	0.32403	2.482807
N	2.042929	1.251586	-0.16338	H	-0.4831	0.323384	2.461075
C	-0.04765	-0.00028	-0.00372	H	-4.18087	-0.61735	-4.59584
C	0.648602	-1.20454	0.156923	H	-1.67711	-0.60714	-4.57263
N	2.045434	-1.24621	0.169058	H	-4.21599	0.601455	4.567011
C	2.421395	-2.53181	0.339311	H	-1.71367	0.601863	4.565712
C	1.264404	-3.3651	0.448063	C	0.077661	-5.69168	0.390865
C	0.163311	-2.52557	0.335439	C	1.259695	-5.79113	-0.5415
C	-1.54668	-0.0022	-0.00604	C	0.063668	5.691348	-0.39903
C	-2.24949	-0.16376	-1.21785	C	1.238549	5.794372	0.541951
C	-3.70057	-0.16664	-1.22234	H	1.924329	5.172901	-1.46213
C	-4.41198	-0.00579	-0.00939	H	1.929004	-5.1679	1.467576
C	-3.69549	0.155745	1.201418	H	-0.81859	-5.20597	0.017128
C	-2.25021	0.157685	1.206874	H	-0.11553	-6.51578	1.071071
B	3.03132	0.004108	0.006042	H	1.161242	-5.35055	-1.52928
O	3.885084	-0.15155	-1.13534	H	1.881399	-6.68104	-0.5013
O	3.877125	0.16225	1.153032	H	-0.83389	5.203067	-0.03184
C	1.286111	4.840429	-0.64436	H	-0.12694	6.514799	-1.08075
C	3.843258	2.980902	-0.35914	H	1.134203	5.353548	1.529016
H	-0.88664	2.797054	-0.39625	H	1.857913	6.6861	0.506209
C	3.849378	-2.97049	0.375991	C	3.321775	-0.32086	-2.4154
C	1.295785	-4.8372	0.645204	H	2.721431	0.549702	-2.72366
H	-0.88113	-2.80002	0.383551	H	2.678751	-1.21351	-2.47183
C	-1.55472	-0.3272	-2.46219	H	4.139632	-0.44301	-3.13299
C	-4.35384	-0.33715	-2.49069	H	4.168132	3.292097	0.642071
C	-5.92535	-0.00071	0.038695	H	3.960857	3.84415	-1.02107
C	-4.36822	0.321843	2.457695	H	4.500057	2.173133	-0.684
C	-1.56726	0.323247	2.456204	H	4.502015	-2.16072	0.704218
C	-3.65001	-0.49104	-3.65674	H	4.181054	-3.28153	-0.62303
C	-2.2274	-0.48578	-3.64427	H	3.965367	-3.83297	1.039225
C	-3.67676	0.478062	3.632263	C	3.304918	0.329674	2.429364
C	-2.25441	0.478615	3.631964	H	2.705828	-0.54308	2.733883
H	-0.4707	-0.32284	-2.45546	H	2.658226	1.21994	2.481472
H	-5.43517	-0.34598	-2.53805	H	4.117634	0.454959	3.152241

Table S21. Optimized geometry (Cartesian coordinates in Å) for compound **5-OMe** with PCM solvation in MeCN (B3LYP/6-31+G(d,p) level).

Symbol	X	Y	Z	Symbol	X	Y	Z
C	1.2769	3.37117	-0.41148	H	-6.2865	-0.80063	0.679819
C	2.434808	2.539614	-0.30662	H	-6.37207	-0.1231	-0.93804
C	0.177181	2.526542	-0.31193	H	-6.29134	0.947206	0.452423
C	0.664771	1.205509	-0.14593	H	-5.43765	0.309379	2.485465
N	2.06222	1.248461	-0.15124	H	-0.46781	0.302856	2.468444
C	-0.03323	0.000438	-0.00372	H	-4.17013	-0.54698	-4.60444
C	0.659614	-1.20709	0.143157	H	-1.66609	-0.5465	-4.58203
N	2.056827	-1.25512	0.156996	H	-4.20251	0.564145	4.573916
C	2.423801	-2.54759	0.314868	H	-1.69998	0.561216	4.573597
C	1.262268	-3.3749	0.412849	C	0.029994	-5.67983	0.463097
C	0.16625	-2.52631	0.306445	C	1.092726	-5.78465	-0.60277
C	-1.53207	0.002958	-0.00618	C	0.053322	5.680541	-0.47037
C	-2.23497	-0.14266	-1.22008	C	1.108293	5.781197	0.603571
C	-3.68652	-0.14278	-1.22427	H	2.032838	5.198716	-1.31411
C	-4.39937	0.002839	-0.0093	H	2.004777	-5.20493	1.321708
C	-3.6809	0.150842	1.20316	H	-0.89544	-5.17402	0.204654
C	-2.23515	0.150957	1.208676	H	-0.09318	-6.51051	1.15152
B	3.03962	-0.00508	0.00602	H	0.888683	-5.32521	-1.56568
O	3.901568	-0.15244	-1.13806	H	1.70065	-6.68381	-0.64513
O	3.894692	0.139364	1.155671	H	-0.87588	5.178013	-0.21915
C	1.306914	4.845964	-0.58393	H	-0.0616	6.511864	-1.15945
C	3.858755	2.991014	-0.33307	H	0.895299	5.322192	1.564749
H	-0.86813	2.798447	-0.35849	H	1.719142	6.678134	0.650839
C	3.845907	-3.00421	0.349882	C	3.327174	-0.304	-2.42225
C	1.285715	-4.84977	0.585907	H	2.730438	0.573585	-2.71124
H	-0.88029	-2.79448	0.34659	H	2.680405	-1.19164	-2.48094
C	-1.54107	-0.29284	-2.46742	H	4.138461	-0.42154	-3.14736
C	-4.34185	-0.29439	-2.49477	H	4.19265	3.272699	0.673938
C	-5.9124	0.005675	0.038987	H	3.96661	3.874478	-0.96829
C	-4.35477	0.305183	2.461094	H	4.516154	2.200451	-0.69537
C	-1.55223	0.302912	2.460621	H	4.503903	-2.2168	0.717969
C	-3.63883	-0.43498	-3.66399	H	4.185464	-3.28514	-0.65544
C	-2.21528	-0.43462	-3.65189	H	3.94629	-3.88927	0.984075
C	-3.66346	0.448869	3.638258	C	3.31235	0.291822	2.436173
C	-2.24016	0.447432	3.638387	H	2.713185	-0.58524	2.7217
H	-0.45673	-0.29266	-2.46376	H	2.66581	1.179913	2.490498
H	-5.42307	-0.29845	-2.5416	H	4.119119	0.409044	3.166359

Table S22. Optimized geometry (Cartesian coordinates in Å) for compound **5-OMe·2H$_2$O** (with two explicit water molecules hydrogen-bonded to the methoxy oxygens) in vacuum (B3LYP/6-31+G(d,p) level).

Symbol	X	Y	Z	Symbol	X	Y	Z
C	-0.55339	3.462833	0.479394	H	5.878138	-0.05175	-2.53329
C	-1.76913	2.725626	0.36759	H	0.924015	0.308838	-2.45273
C	0.480257	2.538716	0.365986	H	4.627437	-0.94384	4.554632
C	-0.10444	1.261708	0.185129	H	2.131133	-0.75282	4.56282
N	-1.4939	1.410939	0.196369	H	4.64222	0.326649	-4.60118
C	0.495966	0.006071	0.017404	H	2.147003	0.508799	-4.56974
C	-0.29271	-1.13821	-0.13473	C	-0.06336	-5.65453	-0.29967
N	-1.69152	-1.07674	-0.12079	C	-1.29056	-5.66751	0.57737
C	-2.16462	-2.33577	-0.27781	C	0.793029	5.696123	0.354853
C	-1.07125	-3.24862	-0.40526	C	-0.44091	5.894875	-0.48944
C	0.089164	-2.49148	-0.31667	H	-1.0104	5.297332	1.559408
C	1.99036	-0.10813	0.001195	H	-1.82033	-4.99841	-1.45975
C	2.693233	-0.32866	1.203618	H	0.847301	-5.23384	0.115889
C	4.140195	-0.43681	1.190096	H	0.100562	-6.49072	-0.97257
C	4.846974	-0.32022	-0.03064	H	-1.20606	-5.23513	1.57004
C	4.12991	-0.09933	-1.23183	H	-1.97289	-6.50958	0.506369
C	2.688696	0.008091	-1.21971	H	1.620507	5.148299	-0.08569
B	-2.55794	0.24741	0.010224	H	1.095668	6.495222	1.024731
O	-3.45664	0.184342	1.146239	H	-0.44592	5.461584	-1.48528
O	-3.37535	0.465226	-1.15681	H	-0.9885	6.828518	-0.3982
C	-0.46517	4.931798	0.690152	C	-2.92045	-0.04299	2.439551
C	-3.15672	3.275874	0.437937	H	-2.23038	0.75922	2.736215
H	1.542661	2.732551	0.412125	H	-2.38409	-1.00045	2.492996
C	-3.61684	-2.67937	-0.29841	H	-3.75187	-0.07186	3.149411
C	-1.2049	-4.7152	-0.60715	H	-3.83419	2.722518	-0.21498
H	1.109526	-2.84256	-0.37945	H	-3.1539	4.331222	0.153555
C	2.003859	-0.44982	2.455724	H	-3.55428	3.204718	1.458702
C	4.794604	-0.66259	2.44912	H	-4.13959	-2.21148	-1.13943
C	6.355886	-0.42497	-0.09722	H	-4.11964	-2.32298	0.605816
C	4.798255	0.024869	-2.49517	H	-3.74334	-3.76144	-0.36393
C	2.005297	0.230057	-2.45994	C	-2.75444	0.600623	-2.42436
C	4.095889	-0.77297	3.623099	H	-2.17172	-0.29324	-2.68861
C	2.677422	-0.66476	3.62837	H	-2.08816	1.4743	-2.4559
C	4.106523	0.237695	-3.66061	H	-3.54112	0.734801	-3.17183
C	2.688168	0.341711	-3.64313	H	-5.25416	0.081324	1.160956
H	0.92291	-0.36773	2.463078	O	-6.22206	-0.07449	1.231086
H	5.872887	-0.7501	2.482738	H	-6.61563	0.778817	1.448366
H	6.665316	-1.2528	-0.7457	H	-5.18399	-0.08472	-1.5196
H	6.816245	-0.59136	0.874088	O	-6.07362	-0.44887	-1.67497
H	6.796666	0.490289	-0.50894	H	-6.44976	-0.47281	-0.77874

Table S23. Optimized geometry (Cartesian coordinates in Å) for compound **5-OMe·2H$_2$O** (with two explicit water molecules hydrogen-bonded to the methoxy oxygens) with PCM solvation in MeCN (B3LYP/6-31+G(d,p) level).

Symbol	X	Y	Z	Symbol	X	Y	Z
C	-0.79879	3.377483	0.462987	H	5.901291	0.321289	-2.45326
C	-1.96398	2.562035	0.334733	H	0.931606	0.366068	-2.46308
C	0.293149	2.522655	0.356425	H	4.586632	-0.73121	4.601415
C	-0.20472	1.210488	0.163209	H	2.083013	-0.70081	4.566835
N	-1.60228	1.268002	0.160043	H	4.680342	0.646145	-4.54037
C	0.481452	0.001645	-0.00114	H	2.178025	0.669195	-4.5532
C	-0.22638	-1.19327	-0.17666	C	0.345812	-5.66526	-0.57075
N	-1.62458	-1.22248	-0.19397	C	-0.73011	-5.77818	0.480768
C	-2.00952	-2.50875	-0.37606	C	0.450757	5.671524	0.54883
C	-0.85942	-3.34737	-0.4887	C	-0.61631	5.803502	-0.50955
C	0.247895	-2.51482	-0.36477	H	-1.52291	5.196287	1.409384
C	1.979823	-0.0135	0.008637	H	-1.61272	-5.15002	-1.4427
C	2.673963	-0.20277	1.221511	H	1.274102	-5.17588	-0.29188
C	4.125334	-0.21838	1.232941	H	0.466938	-6.48336	-1.27437
C	4.846149	-0.04319	0.026572	H	-0.53157	-5.34022	1.454751
C	4.13598	0.146216	-1.18506	H	-1.34941	-6.67032	0.498555
C	2.690408	0.161471	-1.1982	H	1.371011	5.163604	0.27653
B	-2.57784	0.032682	-0.02269	H	0.583479	6.488403	1.251775
O	-3.44621	-0.12982	1.131953	H	-0.42046	5.360007	-1.48157
O	-3.4385	0.209154	-1.17257	H	-1.21804	6.707445	-0.53264
C	-0.81097	4.849333	0.662968	C	-2.84976	-0.277	2.416874
C	-3.38361	3.025429	0.355062	H	-2.28361	0.619485	2.699025
H	1.340625	2.78341	0.414316	H	-2.17633	-1.14299	2.449724
C	-3.4377	-2.94423	-0.41685	H	-3.65029	-0.42812	3.145772
C	-0.89881	-4.81831	-0.69129	H	-3.74771	3.198789	-0.66535
H	1.290695	-2.79627	-0.40847	H	-3.46764	3.969322	0.898512
C	1.971814	-0.38231	2.460308	H	-4.03809	2.2838	0.815253
C	4.772236	-0.41618	2.50134	H	-4.07436	-2.17925	-0.86342
C	6.359328	-0.05202	-0.01344	H	-3.81064	-3.13815	0.59695
C	4.818321	0.328139	-2.43465	H	-3.5362	-3.8728	-0.98406
C	2.015914	0.354827	-2.44897	C	-2.84355	0.361326	-2.4554
C	4.061602	-0.58432	3.662275	H	-2.26714	-0.52874	-2.73925
C	2.638292	-0.56719	3.643211	H	-2.17913	1.234694	-2.49036
C	4.135029	0.511111	-3.61103	H	-3.64498	0.503959	-3.18549
C	2.711857	0.524407	-3.61865	H	-5.17059	-0.42782	1.238536
H	0.887532	-0.37048	2.451866	O	-6.15133	-0.50858	1.328683
H	5.853045	-0.43472	2.553152	H	-6.33245	-1.4514	1.436429
H	6.730497	-0.84973	-0.6667	H	-5.26174	0.502142	-1.29263
H	6.812585	-0.2015	0.963612	O	-6.23871	0.575744	-1.29728
H	6.747806	0.893661	-0.40784	H	-6.47718	0.204602	-0.42601

Table S24. Selected transitions, oscillator strength, symmetry calculated (H = HOMO, L = LUMO) from DFT analysis for **5** in vacuum.

No.	Energy (cm-1)	Wavelength (nm)	Osc. Strength	Symmetry	Major contribs	Minor contribs
1	18008.07	555.3067	0	Singlet-A	H-1->LUMO (100%)	
2	22123.13	452.0155	0.4911	Singlet-A	HOMO->LUMO (94%)	H-2->LUMO (5%)
3	24734.78	404.2891	0.133	Singlet-A	H-1->L+1 (98%)	
4	27774.7	360.0399	0.0843	Singlet-A	H-2->LUMO (93%)	HOMO->LUMO (5%)
5	29305.55	341.2323	0.0449	Singlet-A	H-4->LUMO (97%)	
6	32424.52	308.4086	0.0385	Singlet-A	H-5->LUMO (99%)	
7	36760.59	272.0305	0.074	Singlet-A	H-6->LUMO (78%)	H-7->LUMO (6%), HOMO->L+3 (7%)
8	37559.08	266.2472	0.0341	Singlet-A	HOMO->L+3 (90%)	H-7->LUMO (5%), H-6->LUMO (4%)

Table S25. Selected transitions, oscillator strength, symmetry calculated (H = HOMO, L = LUMO) from DFT analysis for **5** with PCM solvation in MeCN.

No.	Energy (cm-1)	Wavelength (nm)	Osc. Strength	Symmetry	Major contribs	Minor contribs
1	16737.73	597.4525	0	Singlet-A	HOMO->LUMO (100%)	
2	20868.93	479.1812	0.5746	Singlet-A	H-1->LUMO (97%)	H-2->LUMO (2%)
3	24489.58	408.3369	0.1708	Singlet-A	HOMO->L+1 (98%)	
4	27102.84	368.9651	0.0562	Singlet-A	H-2->LUMO (96%)	H-1->LUMO (2%)
5	28062.64	356.3456	0.0417	Singlet-A	H-3->LUMO (97%)	H-6->LUMO (2%)
6	28356.23	352.6562	0.0839	Singlet-A	H-4->LUMO (97%)	
7	31342.92	319.0513	0.0576	Singlet-A	H-5->LUMO (99%)	
8	36408.12	274.664	0.0814	Singlet-A	H-6->LUMO (83%)	H-12->LUMO (2%), H-8->LUMO (7%)
9	38801.99	257.7188	1.7265	Singlet-A	H-4->L+1 (38%), H-2->L+1 (11%), HOMO->L+2 (48%)	

Table S26. Selected transitions, oscillator strength, symmetry calculated (H = HOMO, L = LUMO) from DFT analysis for **5-OMe** in vacuum.

No.	Energy (cm-1)	Wavelength (nm)	Osc. Strength	Symmetry	Major contribs	Minor contribs
1	19059.82	524.6639	0	Singlet-A	H-1->LUMO (100%)	
2	21498.86	465.141	0.0002	Singlet-A	HOMO->L+1 (100%)	
3	21951.34	455.5531	0.4467	Singlet-A	HOMO->LUMO (92%)	H-2->LUMO (8%)
4	24717.84	404.5661	0.1357	Singlet-A	H-1->L+1 (98%)	
5	26282.56	380.4804	0.11	Singlet-A	H-2->LUMO (88%)	H-6->LUMO (3%), HOMO->LUMO (8%)
6	33520.63	298.3237	0.0256	Singlet-A	H-7->LUMO (99%)	
7	35976.61	277.9584	0.0433	Singlet-A	H-8->LUMO (79%), HOMO->L+3 (11%)	H-13->LUMO (3%)
8	36554.11	273.5671	0.0409	Singlet-A	HOMO->L+3 (89%)	H-8->LUMO (9%)

Table S27. Selected transitions, oscillator strength, symmetry calculated (H = HOMO, L = LUMO) from DFT analysis for **5-OMe** with PCM solvation in MeCN.

No.	Energy (cm-1)	Wavelength (nm)	Osc. Strength	Symmetry	Major contribs	Minor contribs
1	18133.08	551.4782	0	Singlet-A	H-1->LUMO (100%)	
2	21044.76	475.1776	0.5519	Singlet-A	HOMO->LUMO (96%)	H-2->LUMO (4%)
3	22223.15	449.9813	0.0001	Singlet-A	HOMO->L+1 (100%)	
4	24473.45	408.6061	0.1722	Singlet-A	H-1->L+1 (98%)	
5	26797.15	373.174	0.1153	Singlet-A	H-2->LUMO (94%)	HOMO->LUMO (4%)
6	27929.56	358.0436	0.0353	Singlet-A	H-3->LUMO (97%)	
7	32765.69	305.1973	0.0336	Singlet-A	H-7->LUMO (98%)	
8	35994.35	277.8214	0.0747	Singlet-A	H-8->LUMO (88%)	H-14->LUMO (2%)
9	37168.7	269.0435	0.0327	Singlet-A	HOMO->L+3 (97%)	

Table S28. Selected transitions, oscillator strength, symmetry calculated (H = HOMO, L = LUMO) from DFT analysis for **5-OMe·2H_2O** in vacuum.

No.	Energy (cm-1)	Wavelength (nm)	Osc. Strength	Symmetry	Major contribs	Minor contribs
1	18048.39	554.0659	0	Singlet-A	H-1->LUMO (100%)	
2	21789.22	458.9426	0.424	Singlet-A	HOMO->LUMO (94%)	H-2->LUMO (6%)
3	22416.72	446.0956	0.0002	Singlet-A	HOMO->L+1 (100%)	
4	24730.74	404.355	0.1392	Singlet-A	H-1->L+1 (98%)	
5	27274.63	366.641	0.0904	Singlet-A	H-2->LUMO (93%)	HOMO->LUMO (6%)
6	28404.62	352.0554	0.0289	Singlet-A	H-3->LUMO (96%)	
7	32656.81	306.2149	0.0267	Singlet-A	H-8->LUMO (27%), H-7->LUMO (55%), H-6->LUMO (17%)	
8	36154.86	276.588	0.0612	Singlet-A	H-9->LUMO (84%)	H-13->LUMO (3%), HOMO->L+3 (2%)
9	37471.97	266.8661	0.0308	Singlet-A	HOMO->L+3 (91%)	H-10->LUMO (4%), H-9->LUMO (2%)

Table S29. Selected transitions, oscillator strength, symmetry calculated (H = HOMO, L = LUMO) from DFT analysis for **5-OMe·2H_2O** with PCM solvation in MeCN.

No.	Energy (cm-1)	Wavelength (nm)	Osc. Strength	Symmetry	Major contribs	Minor contribs
1	17420.08	574.0501	0	Singlet-A	H-1->LUMO (99%)	
2	20891.52	478.6632	0.5248	Singlet-A	HOMO->LUMO (96%)	H-2->LUMO (4%)
3	22832.91	437.9644	0.0001	Singlet-A	HOMO->L+1 (100%)	
4	24477.48	408.5387	0.1757	Singlet-A	H-1->L+1 (98%)	
5	27143.16	368.4169	0.0933	Singlet-A	H-2->LUMO (95%)	HOMO->LUMO (3%)
6	27936.82	357.9506	0.0405	Singlet-A	H-3->LUMO (97%)	
7	28768.38	347.6038	0.0393	Singlet-A	H-4->LUMO (98%)	
16	36183.09	276.3722	0.0774	Singlet-A	H-8->LUMO (79%)	H-7->LUMO (9%)
17	37785.72	264.6502	0.0316	Singlet-A	HOMO->L+3 (97%)	

8. References

[1] J.J. Snellenburg, S. Laptenok, R. Seger, K.M. Mullen, I.H.M van Stokkum, *J. Stat. Softw.*, 2012, *49*, 3, 1-22.
[2] M. A. Filatov, S. Karuthedath, P. M. Polestshuk, S. Callaghan, K. J. Flanagan, M. Telitchko, T. Wiesner, F. Laquai, M. O. Senge, *Phys. Chem. Chem. Phys.*, **2018**, *20*, 8016–8031.
[3] M. A. Filatov, S. Karuthedath, P. M. Polestshuk, S. Callaghan, K. J. Flanagan, T. Wiesner, F. Laquai, M. O. Senge, *ChemPhotoChem* **2018**, *2*, 606–615.
[4] K. Coldrick, C. Newman, J. Doran, G. Amarandei, M. A. Filatov, *ACS Appl. Opt. Mater.* **2024**, *2*, 1985–1998.
[5] L. Wu, K. Burgess, *Chem. Commun.*, **2008**, 4933–4935.
[6] H. Sunahara, Y. Urano, H. Kojima, T. Nagano, *J. Am. Chem. Soc.* **2007**, *129*, 5597–5604.
[7] M. K. Raza, S. Gautam, P. Howlader, A. Bhattacharyya, P. Kondaiah, A. R. Chakravarty, *Inorg. Chem.* **2018**, *57*, 14374–14385.
[8] M. Gao, C. He, H. Chen, R. Bai, B. Cheng and Ana. Lei, *Angew. Chem. Int. Ed.* **2013**, *52*, 6958–6961
[9] C.-X. Zhuo, Y. Zhou and S.-L. You, *J. Am. Chem. Soc.* **2014**, *136*, 18, 6590–6593
[10] T. Sprenger, T. Schwarze, H. Holdt, A. Hentsch, M. Nazaré, *Chem. Eur. J.* **2024**, *30*, e202401928.
[11] A. M. Courtis, S. A. Santos, Y. Guan, J. A. Hendricks, B. Ghosh, M. Szantai-Kis, S. A. Reis, J. V. Shah, R. Mazitschek, *Bioconjugate Chem.* **2014**, *25*, 6, 1043–1051.
[12] A. L. Nguyen, P. Bobadova-Parvanova, M. Hopfinger, F. R. Fronczek, K. M. Smith, M. G. H. Vicente, *Inorg. Chem.* **2015**, *54*, 7, 3228–3236.
[13] Y. Gabe, T. Ueno, Y. Urano, H. Kojima, T. Nagano, *Anal. Bioanal. Chem.*, **2006**, *386*, 621–626.
[14] B. Brizet, C. Bernhard, Y. Volkova, Y. Rousselin, P. D. Harvey, C. Goze, F. Denat, *Org. Biomol. Chem.*, **2013**,*11,* 7729-7737.
[15] S. Zhu, J. Zhang, G. Vegesna, F. Luo, S. A. Green, H. Liu, *Org. Lett.* **2011**, 13, 438-441.
[16] Y. Jin, M. Lv, Y. Tao, S. Xu, J. He, J. Zhang, W. Zhao, *Spectrochim. Acta Part A*, **2019**, *219*, 569–575.
[17] C. Schad, C. Ray, C. Díaz-Norambuena, S. Serrano-Buitrago, F. Moreno, B. L. Maroto, I. García-Moreno, M. Muñoz-Úbeda, I. López-Montero, J. Bañuelos, S. de la Moya, *Chem. Sci.*, **2025**,*16*, 8030-8039.
[18] A. Blázquez-Moraleja, D. Álvarez-Fernández, R. P. Montero, I. García-Moreno, V. Martínez-Martínez, J. Bañuelos, I. Sáenz-de-Santa-María, M. D. Chiara, J. L. Chiara, *Dyes and Pigments* **2019**, *170*, 107545.
[19] N. Umeda, H. Takahashi, M. Kamiya, T. Ueno, T. Komatsu, T. Terai, K. Hanaoka, T. Nagano, Y. Urano, ACS Chem. Biol., **2014**, *9*, 10, 2242–2246.
[20] A. Sheehan, I. A. Okkelman, G. Groslambert, C. Bucher, R. I. Dmitriev, M. A. Filatov, *Chem. Eur. J.* **2025**, *31*, e202404188.
[21] S. Callaghan, M. A. Filatov, H. Savoie, R. W. Boyle, M. O. Senge, *Photochem. Photobiol. Sci.*, **2019**,*18*, 495-504
[22] K. Coldrick, C. Newman, J. Doran, G. Amarandei, M. A. Filatov, *ACS Appl. Opt. Mater.* **2024**, *2*, 1985−1998.
[23] R. F. Kubin, A. N. Fletcher, *J. Lumin.,* **1982**, *27*, 455–462.
[24] A. C. Debruyne, G. Ferrari, H. Zhou, N. Van Loon, N. Heymans, I. A. Okkelman, R. I. Dmitriev, *JoVE* **2024**, *210*, e66845.
[25] I. A. Okkelman, T. Foley, D. B. Papkovsky, R. I. Dmitriev, *Biomaterials* **2017**, *146*, 86–96.
[26] N. Barker, J. H. van Es, J. Kuipers, P. Kujala, M. van den Born, M. Cozijnsen, A. Haegebarth, J. Korving, H. Begthel, P. J. Peters, H. Clevers, *Nature* **2007**, *449*, 1003–1007.
[27] I. A. Okkelman, J. Puschhof, D. B. Papkovsky, R. I. Dmitriev, Visualization of Stem Cell Niche by Fluorescence Lifetime Imaging Microscopy, in *Intestinal Stem Cells: Methods and Protocols* (Ed.: P. Ordóñez-Morán), Springer US, New York, NY, **2020**, pp. 65–97.
[28] A. C. Debruyne, I. A. Okkelman, N. Heymans, C. Pinheiro, A. Hendrix, M. Nobis, S. M. Borisov, R. I. Dmitriev, *ACS Nano* **2024**, *18*, 12168–12186.
[29] A. Vallmitjana, B. Torrado, A.F. Durkin, A. Dvornikov, N. Rajil, S. Ranjit, M. Balu, *Bioinformatics* **2025**, *41*, btaf162.
[30] D. Rehm, A. Weller, *Isr. J. Chem.*, **1970**, *8*, 259–271.
[31] J. T. Buck, A. M. Boudreau, A. DeCarmine, R. W. Wilson, J. Hampsey, T. Mani, *Chem*, **2018**, *5*, 1
[32] G. M. Morris, R. Huey, W. Lindstrom, M. F. Sanner, R. K. Belew, D. S. Goodsell, A. J. Olson, *J. Comput. Chem.* **2009**, *30*, 2785–2791.
[33] E. C. Meng, T. D. Goddard, E. F. Pettersen, G. S. Couch, Z. J. Pearson, J. H. Morris, T. E. Ferrin, *Protein Sci.* **2023**, *32*, e4792.
